# Ruthenium(II)-enabled *para*-selective C–H difluoromethylation of anilides and their derivatives

**DOI:** 10.1038/s41467-018-03341-6

**Published:** 2018-03-22

**Authors:** Chunchen Yuan, Lei Zhu, Changpeng Chen, Xiaolan Chen, Yong Yang, Yu Lan, Yingsheng Zhao

**Affiliations:** 1 Key Laboratory of Organic Synthesis of Jiangsu Province College of Chemistry, Chemical Engineering, and Materials Science Soochow University, Suzhou, 215123 China; 2 School of Chemistry and Chemical Engineering Chongqing University, Chongqing, 40030 China; 3 Qingdao Institute of Bioenergy and Bioprocess Technology, Chinese Academy of Science, Qingdao, 266000 China

## Abstract

Transition-metal-catalyzed direct site-selective functionalization of arene C–H bonds has emerged as an innovative approach for building the core structure of pharmaceutical agents and other versatile complex compounds. However, *para*-selective C–H functionalization has seldom been explored, only a few examples, such as steric-hindered arenes, electron-rich arenes, and substrates with a directing group, have been reported to date. Here we describe the development of a ruthenium-enabled *para*-selective C–H difluoromethylation of anilides, indolines, and tetrahydroquinolines. This reaction tolerates various substituted arenes, affording *para*-difluoromethylation products in moderate to good yields. Results of a preliminary study of the mechanism indicate that chelation-assisted cycloruthenation might play a role in the selective activation of *para*-C_Ar_–H bonds. Furthermore, this method provides a direct approach for the synthesis of fluorinated drug derivatives, which has important application for drug discovery and development.

## Abstract (web summary)

Selective para-functionalization of substituted arenes is a formidable challenge in homogeneous catalysis. Here, the authors achieved the para-selective C-H difluoromethylation of anilides, indolines and tetrahydroquinolines with a ruthenium catalyst in good yields and apply it to the synthesis of bioactive compounds.

## Introduction

**Fig. 1:**
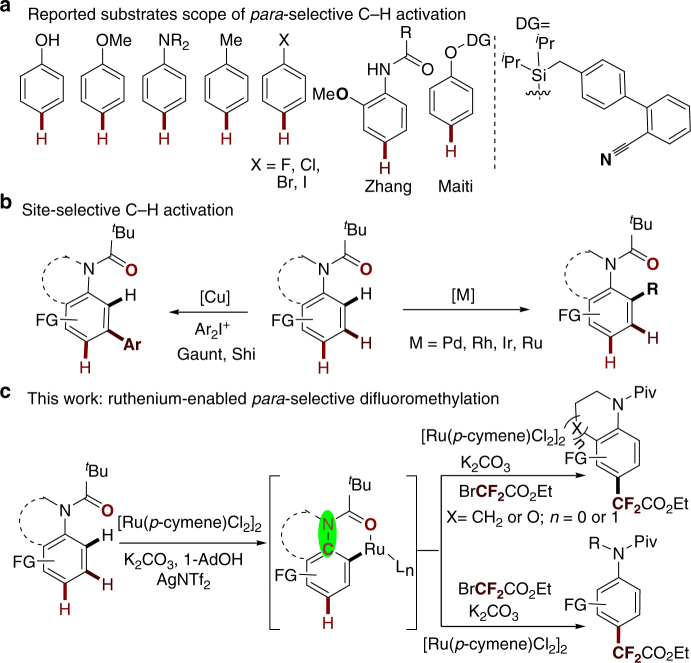
Site-selective C–H activation reactions. **a** Reported substrate scope of *para*-selective C–H activation. **b** Site-selective C–H activation. **c** Our work on ruthenium-enabled *para*-selective difluoromethylation

Over the past decade, transition-metal-catalyzed direct site-selective functionalization of arene C–H bonds has emerged as an innovative approach for building the core structure of pharmaceutical agents and other versatile complex compounds. In this context, various *ortho*-selective C–H functionalization reactions have been well-developed through the σ-chelation-directed cyclometalation strategy^1–9^. However, remote C–H functionalization still remains a great challenge. Compared with recently developed *meta*-selective C–H functionalization^10–26^, *para*-selective C–H functionalization is less explored;^27–34^ only a few examples, such as steric-hindered arenes^35,36^, electron-rich arenes^37–43^, and substrates with a directing group^44^, have been reported to date. For instance, Zhang and co-workers reported in 2011 a Pd(II)-catalyzed *para*-selective amination of *ortho*-methoxy-substituted anilide. Interestingly, substrate blockage of both *ortho*positions was also tolerated (Fig. 1a). In 2015, Maiti’s group independently developed a D-shaped template for the highly selective olefination of the *para*-C–H bond using a palladium catalyst (Fig. 1a)^45^. Very recently, a variety of *para*-selective functionalizations of less-activated arenes via nickel/aluminum^46^, gold^47^, and palladium^48^ catalyzes have been reported. Despite such major advances, most *para*-selective C–H functionalizations suffer from serious drawbacks such as limited substrate scope and relatively poor regioselectivity, which significantly restrict their applications (Fig. 1a)^27–34^. Therefore, a catalyst-controlled strategy for *para*-selective C–H functionalization of arenes is highly desired.

Fluorine-containing compounds are widely applied in pharmaceuticals, agrochemicals, and life sciences^49,50^. Specifically, the installation of a difluoromethylene (CF_2_) group into organic compounds is of particular value because it can cause significant changes in the chemical and physical properties of biologically active compounds^51,52^. As a consequence, there is a continued strong demand for methods that enable the selective synthesis of difluoromethylated molecules^53–56^.

Despite the development of various approaches to *ortho*- and *meta*-selective C–H functionalizations of anilides, indolines, and tetrahydroquinolines, which are significantly important molecular skeletons in medicinal chemistry (Fig. 1b)^57–62^, the general strategy for highly *para*-selective C–H functionalization is still elusive. Herein, we report a ruthenium(II)-enabled *para*-selective difluoromethylation of anilides, indolines, and tetrahydroquinolines. This reaction tolerates a wide variety of functional groups, affording the corresponding *para*-difluoromethylated products in moderate to good yields. Preliminary experimental results suggest that chelation-assisted cycloruthenation might play a role in the selective activation of *para*-C_Ar_–H bonds.

## Results

### Optimization of reaction conditions

At the outset, *N*-pivaloylaniline **1a** was reacted with bromodifluoroacetate **2** in the presence of [Ru(*p*-cymene)Cl_2_]_2_ (5 mol%), 1-Ad-OH (0.2 equiv.), and K_2_CO_3_ (4 equiv.) in DCE at 120 °C to investigate whether the *para*-selective C–H difluoromethylation could be performed. We were pleased to observe that we generated the *para*-difluoromethylated product **3a** in 45% yield (Table 1, entry 1). Encouraged by this result, we decided to further optimize the conditions to improve the efficiency. We investigated several additives, e.g., MesCO_2_H, Piv-Val-OH, Piv-OH, and KOAc, but none of them gave a noticeable enhancement (Table 1, entries 2–5). Subsequently, various silver salts that are known as facilitating to active the rhodium or iridium catalyst precursors were screened. To our great delight, dramatically improved yields of **3a** were obtained (53–65%; Table 1, entries 6–8). We could further enhance the reaction efficiency by an increase in AgNTf_2_ (20 mol%) loading, which provided product **3a** in 87% yield (Table 1, entry 9). In addition, the use of RuCl_3_, Pd(PPh_3_)_4_, or Cu_2_O, Ni(acac)_2_ or the obviation of [Ru(*p*-cymene)Cl_2_]_2_ failed to lead to *para*-selective transformation under otherwise identical conditions (Table 1, entries 10–14).

**Table 1:** Optimization of *para*-C–H difluoromethylation^a^


Entry	Catalyst	Silver salt	Additive	Yield (%)^b^
	(5 mol%)	(10 mol%)	(20 mol%)	
1	[Ru(*p*-cymene)Cl_2_]_2_	No	1-Ad-OH	45
2	[Ru(*p*-cymene)Cl_2_]_2_	No	KOAc	35
3	[Ru(*p*-cymene)Cl_2_]_2_	No	Piv-OH	39
4	[Ru(*p*-cymene)Cl_2_]_2_	No	MesCO_2_H	40
5	[Ru(*p*-cymene)Cl_2_]_2_	No	Piv-Val-OH	32
6	[Ru(*p*-cymene)Cl_2_]_2_	AgBF_4_	1-Ad-OH	51
7	[Ru(*p*-cymene)Cl_2_]_2_	AgSbF_6_	1-Ad-OH	48
8	[Ru(*p*-cymene)Cl_2_]_2_	AgNTf_2_	1-Ad-OH	65
9	[Ru(*p*-cymene)Cl_2_]_2_	AgNTf_2_	1-Ad-OH	87^c^
10	RuCl_3_	AgNTf_2_	1-Ad-OH	21^c^
11	Pd(PPh_3_)_4_	No	1-Ad-OH	Trace
12	Cu_2_O	No	1-Ad-OH	Trace
13	Ni(acac)_2_	No	1-Ad-OH	Trace
14	No	AgNTf_2_	1-Ad-OH	0

^a^ Reaction condition: 1a (0.20 mmol, 1.0 equiv.), 2 (3 equiv.), K2CO3 (4 equiv.), catalyst (5 mol%), additive (20 mol%), and Ag salt (10 mol%) in DCE (0.5 mL) for 48 h at 120 °C under argon in a sealed tube

^b^ GC yield using biphenyl as the internal standard

^c^ Ag salt (20 mol%)

### Scope of the anilide derivatives

Having identified the optimal reaction conditions, we next investigated the substrate scope to explore the versatility of this *para*-selective difluoromethylation protocol (Table 2). First, a series of *ortho*-substituted *N*-phenylpivalamides (**1a**–**1i**) performed well under the standard conditions; the corresponding *para*-difluoromethylated products **3a**–**3i** were exclusively obtained in yields of 38–84% along with the recovered starting material. Various functional groups, such as Me, Et, OMe, OPh, *t*-Bu, Cl, Br, and CO_2_Me, were fully tolerated. A sterically hindered functional group (^*t*^Bu, **1e**) and electron-withdrawing substituents (Cl, Br, CO_2_Me, **1g**–**1i**) gave slightly inferior yields. It is noteworthy that *o*-methoxy-substituted *N*-phenylpivalamides (**1d**) were exclusively transformed into *p*-difluoromethylated products (**3d**) without formation of the product difluoromethylated at the C5 position. Subsequently, *meta*-substituted *N*-phenylpivalamides (**1j**–**1p**) were used in the reaction. Overall, the reaction tolerated a wide range of functional groups at the *meta* position and afforded a decent yield with excellent regioselectivity. The difluoromethylation was found to prefer sterically bulkier positions (C4) over the less sterically hindered positions (C5). Of note are the exceptions, the *meta*-substituted substrates **1o** and **1p**, which afforded a mixture of *ortho*- and *para*-difluoromethylated products, respectively. It is probable that the electron-donating effect of the methoxy or methylthio groups greatly influenced the reactivity of the C–Ru bonds. Thus, the ruthenium(II) complex could successively trap electrophilic ·CF_2_CO_2_Et radicals and undergo reductive elimination, affording *ortho*-difluoromethylated products (**3o**^**(6)**^, **3p**^**(6)**^). Moreover, the steric effect of the thiomethyl group resulted in a low yield of *para*-difluorometylated product **3p**. Moreover, all of the disubstituted substrates **3q**–**3s** performed well, providing the *para*-difluoromethylated products in good yields.

**Table 2:** Substrate scope of anilides^a^

^a^ Reaction conditions: [Ru(*p*-cymene)Cl2]2 (5 mol%), **1** (0.20 mmol, 1.0 equiv.), K2CO3 (4 equiv.), 1-Ad-OH (20 mol%), AgNTf2 (20 mol%), and **2** (3 equiv.) in DCE (0.5 mL) at 120 °C for 48 h under argon; isolated yield after chromatography

These results might indicate that site selectivity is controlled by ruthenium catalysis rather than by functional groups on the aromatic ring. This *para-*selective difluoromethylation protocol is also amenable to *N*-alkylanilines protected with pivaloyl amide. All of the substrates reacted well under standard conditions, affording the *para*-difluoromethylated products in satisfactory yields (**3t**, **3u**). However, *ortho*- and *meta*-trifluoromethyl-substituted phenylpivalamides were less reactive, affording difluoromethylated products in only trace amounts (Supplementary Figs. 3-7). When acetyl- and benzoyl-protected aniline were subjected to standard reaction conditions, the difluoromethylated products were obtained in moderate to good yields (**3v**, **3w**). We were puzzled as to how the substrates with a small, strongly electronegative fluorine substituted pivaloyl amide either at the *ortho* or *meta* position, providing exclusively *ortho*-difluoromethylated products (Supplementary Figs. 2, 3sa, 3sb) in good yields. We may attribute this to the electron-withdrawing effect of fluorine, which suppressed the directing ability of *ortho* cycloruthenation and thereby caused selectivity toward different sites (Supplementary Figs. 3–10).

### Scope of the substrates indolines and tetrahydroquinolines

**Fig. 2:**
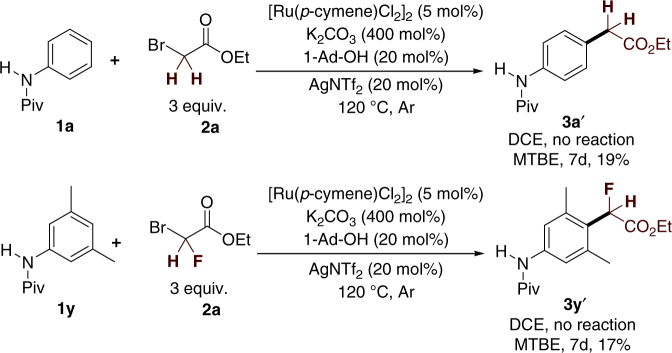
Monofluoromethylation and non-fluoromethylation. **a** Ruthenium(II)-enabled *para*-selective non-fluoromethylation. **b** Ruthenium(II)-enabled *para*-selective monofluoromethylation

The structural units indoline and tetrahydroquinolines are highly important molecular skeletons in medicinal chemistry; they are widely found in several well-known drugs, such as indapamide, ajmaline, oxamniquine, and argatroban. To explore the generality of this protocol, a number of indoline and tetrahydroquinoline derivatives protected with pivaloyl amide were used as substrates for *para*-selective difluoromethylation. Gratifyingly, all of these substrates were also compatible with the reaction, delivering the desired *para*-difluoromethylated products in moderate to good yields (Table 3). A set of functional groups, such Me, Cl, Br, and Ph, were all tolerated by this procedure. It is worth mentioning that the single-crystal structure of product **5c** confirms that the ruthenium-enabled difluoromethylation selectively occurs at the *para* position. In addition, we extended the reaction to monofluoromethylation and non-fluoromethylation, but we obtained only <20% yields of the corresponding products under harsh reaction conditions (Fig. 2). This result may be attributed to the stability of the corresponding free radicals.

**Table 3:** Substrate scope of indolines and tetrahydroquinolines^a^

^a^ Reaction conditions: [Ru(*p*-cymene)Cl2]2 (5 mol%), **1** (0.20 mmol, 1.0 equiv.), K2CO3 (4 equiv.), 1-Ad-OH (20 mol%), AgNTf2 (20 mol%), and 2 (3 equiv.) in DCE (0.5 mL) at 120 °C for 48 h under argon; isolated yield after chromatography

**Fig. 3:**
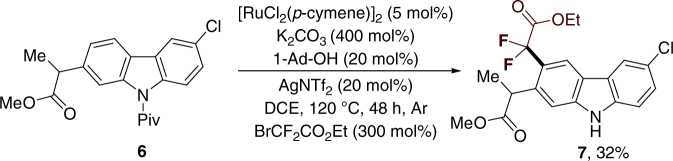
The application in organic synthesis. Synthesis of a carprofen derivative

**Fig. 4:**
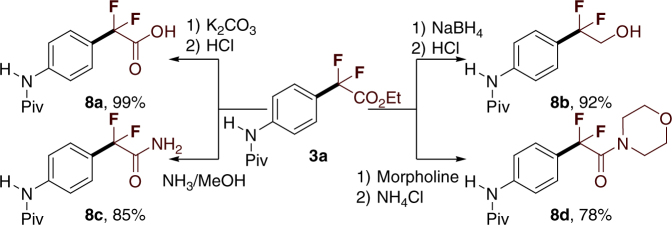
Transformations of **3a**. All the reactions were performed in 0.2 mmol scale. Isolated yields. **a** K_2_CO_3_, MeOH, 60 °C. **b** NaBH_4_, EtOH, r.t. **c** NH_3_, MeOH, 60 °C. **d** Morpholine, 60 °C

The importance and utility of this protocol can be highlighted by the synthesis of leflunomide (Supplementary Fig. 13), and a carprofen derivative (Fig. 3)^63,64^, which is a non-steroidal anti-inflammatory drug. Because of the possible decomposition of carprofen protected with pivaloyl amide in the reaction, a relatively low yield of **7** was afforded. Furthermore, the aryldifluoroacetates could be used as precursors to access a variety of difluoromethyl-containing organic molecules such as carboxylic acid (Fig. 4, **8a**), primary alcohol (Fig. 4, **8b**), and amides (Fig. 4, **8c**–**8d**) in high yields, through different known procedures, as illustrated in Fig. 3. As demonstrated earlier, this method may be a unique and highly efficient protocol for drug discovery and development (Fig. 4).

### Mechanistic investigations

**Fig. 5:**
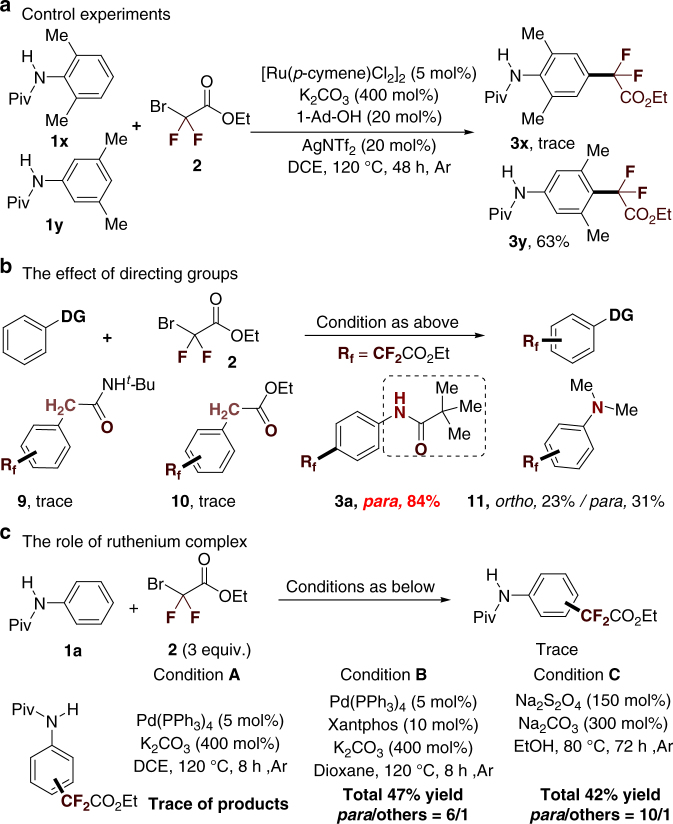
Preliminary studies on the mechanism. **a** Control experiments. **b** The effect of directing group. **c** The role of ruthenium complex

**Fig. 6:**
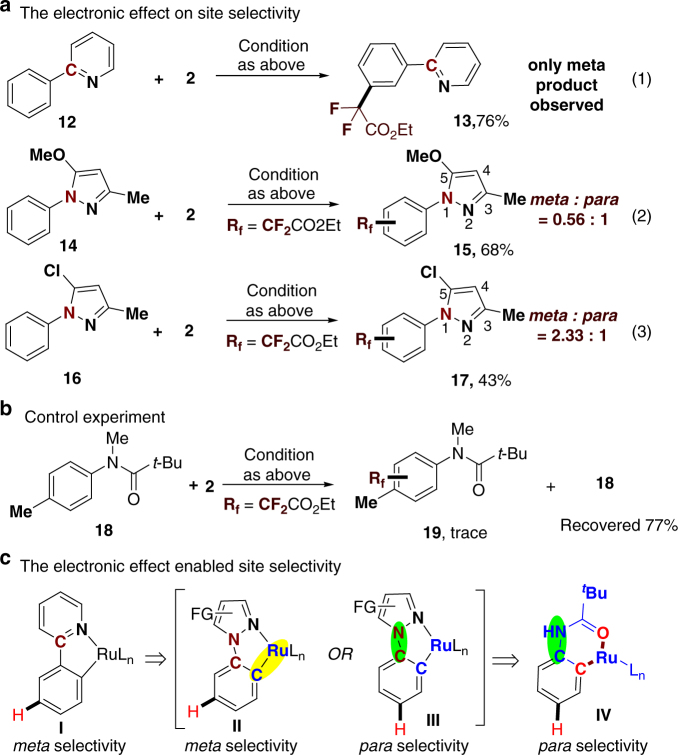
Preliminary studies on site selectivity. **a** The electronic effect on site selectivity. **b** Control experiment. **c** The electrionic enabled site selectivity

To understand the reaction pathway of this ruthenium-catalyzed site-selective difluoromethylation reaction, additional experiments were extensively performed. First, a control experiment was conducted by using 2,6-dimethyl-substituted aniline as a substrate under the standard conditions (Fig. 5, **1x**). In this case, no desired product was formed and the starting material was completely recovered. In sharp contrast, the pivaloyl amide protected 3,5-dimethylaniline (Fig. 5, **1y**) gave the *para*-difluoromethylated products **3y** in 63% yield (Fig. 5a). Second, no formation of difluoromethylated products were obtained when ethylphenyl acetate and *N*-(*tert*-butyl)-2-phenylacetamide were coupled with **2** under standard conditions (Fig. 5b). Interestingly, the reaction of *N*,*N*-dimethylaniline^37^ as a substrate proceeded well but afforded a mixture of *ortho*- and *para*-difluoromethylated products **11** (*ortho*/*para* ratio = 1:1.4). These results indicate that *ortho*-C_Ar_–H metalation plays an important role in accessing *para*-C–H functionalization reactions^17^. To further test this hypothesis, several other control experiments were performed (Fig. 5c). The ·CF_2_CO_2_Et radical can be generated from 2-bromo-2,2-difluoroacetates, as reported by the groups of Ackermann^25^, Wang^26^, and Kondratov^65^. Thus, we directly treated the substrate **1a** with **2** in the presence of the radical initiator. Interestingly, we found that the difluoromehyl radical could be trapped by the substrate **1a**. However, only a mixture of *ortho*-, *meta*-, and *para*-difluoromethylated products were obtained. These results suggest that the arylruthenium intermediate (Fig. 6c, IV) might play a role in realizing the *para* selectivity.

Third, a set of parallel experiments were performed in order to gain insights into the *para* selectivity (Fig. 6). We found that only the *meta*-difluoromethylated product **13** was generated in 76% yield when 2-phenylpyridine (**12**) and bromodifluoroacetate (**2**) were treated under standard conditions (Fig. 6**a**, Eq. (1)). This result is consistented with the reports of Ackermann and Wang on *meta*-selective difluoromethylation^25,26^. However, when the pyrazole derivative **14** was utilized in the reaction, a mixture of *meta*- and *para*-difluoromethylated products **15** (0.56:1) was obtained in 68% total yield (Fig. 6, Eq. (2)). Interestingly, a decrease in the electron-donating effect on **N**_**1**_ in the pyrazole ring significantly resulted in the regioselectivity shift from the *para*- to the *meta*-position (Fig. 6, Eqs. (2) and (3)). These results clearly indicate that the site selectivity could be elegantly tuned by modifying the electronic effect of **N**_**1**_. Finally, pivaloyl amide protected *N*-methyl-*p*-toluidine **18**, in which the *para* position was blocked with a –Me group, only afforded <5% yield of difluoromethylated products, along with 77% recovery of the starting material (Fig. 6b), indicating that the ruthenium-catalyzed C–H functionalizations tend to occur at the *para*-C_Ar_–H position of anilides.

**Fig. 7:**
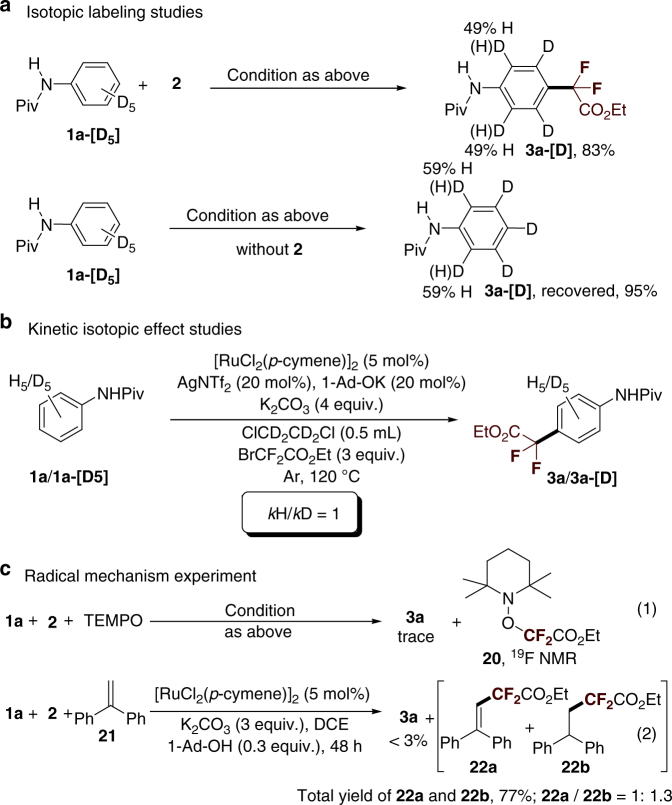
Deuteration and radical experiments. **a** Isotopic labeling studies. **b** Kinetic isotopic effect studies. **c** Radical mechanism experiment

The *para* difluoromethylation of the isotopically labeled substrate was investigated (Fig. 7). We found that treating **1a-[D**_**5**_**]** with **2** under standard reaction conditions afforded the product **3a-[D]** in 83% yield, with significant D/H scrambling at the *ortho*position. A similar D/H scrambling result was observed when **1a-[D**_**5**_**]** was subjected to standard reaction conditions without **2** (Fig. 7a). This result indicates that *ortho*cycloruthenation is reversible. The observed kinetic isotope effect value of 1.0 (Fig. 7b) suggests that C_Ar_–H activation is not a kinetically relevant step. Next, we tried to explore the nature of the *para*-carbon–carbon bond formation using **2**. The reaction conducted in the presence of the radical scavenger TEMPO resulted in complete inhibition of catalytic activity without formation of the desired product **3a**. A further ^19^F NMR study revealed that **20** may have been generated in the reaction, in good agreement with the results of Ackermann et al.^25,26^. We subsequently introduced the radical scavenger, 1,1-diphenylethylene (**21**)^66,67^, which could trap the ·CF_2_CO_2_Et radical generated in situ in the reaction (Fig. 7c). As expected, the products **22** formed by coupling **21** with ·CF_2_CO_2_Et were predominantly obtained in 77% total yield, and only a trace amount of **3a** was observed along with recovery of **1a** (Fig. 7c). We further treated **21** with **2** directly in the presence of ruthenium catalysts and K_2_CO_3_ in 1,4-dioxane for 12 h in order to obtain a mixture of **22a** and **22b** in yields >40%. Taken together, all of the foregoing observations suggest the involvement of a free radical pathway for this *para*-selective difluoromethylation, in which the ruthenium(II) complex can release a ·CF_2_CO_2_Et radical through single-electron transfer with **2** as the oxidant.

## Discussion

In conclusion, we demonstrated the *para*-selective C–H difluoromethylations of various anilides and indolines using a ruthenium catalyst. In addition, this method provides an efficient approach to directly access fluorinated bioactive compounds derivatives, which is significant for the development of new drugs. Preliminary experimental results show that the catalyst provides *para* selectivity and that it can release the electrophilic ·CF_2_CO_2_Et radical from ethylbromodifluoroacetate. Further applications and mechanistic studies including DFT calculations are now underway.

## Methods

### Procedure for Ru-catalyzed *para*-C−H difluoromethylation

A mixture of 1 or 4 (0.2 mmol, 1.0 equiv.), BrCF_2_CO_2_Et (80 μL, 121.2 mg, 3 equiv.), [Ru(*p*-cymene)Cl_2_]_2_ (6 mg, 5 mol%), K_2_CO_3_ (108.8 mg, 400 mol %), 1-Ad-OH (7.2 mg, 20 mol%), AgNTf_2_ (14.4 mg, 20 mol%), and DCE (0.5 mL) in a 15 mL glass vial sealed under argon atmosphere was heated at 120 °C for 48 h. The reaction mixture was cooled to room temperature and concentrated in vacuo. The resulting residue was purified by column chromatography (PE/EA = 10: 1) on silica gel to give the product 3 or 5. Full experimental details and characterization of new compounds can be found in the Supplementary Methods.

### Data availability

The authors declare that all relevant data supporting the findings of this study are available within the article and its Supplementary Information files. The X-ray crystallographic coordinates for structures reported in this study have been deposited at the Cambridge Crystallographic Data Centre (CCDC), under deposition numbers 1513022. These data can be obtained free of charge from The Cambridge Crystallographic Data Centre via www.ccdc.cam.ac.uk/data_request/cif.

## Electronic supplementary material

Supplementary Information(PDF 10712 kb)

Description of Additional Supplementary Files(PDF 49 kb)

Supplementary Data 1(CIF 1538 kb)

Supplementary Data 2(PDF 106 kb)
