# Supplementary material for: Ruthenium(II)-enabled para-selective C–H difluoromethylation of anilides and their derivatives
Source: Nat Commun. 2018 Mar 22;9:1189. doi: 10.1038/s41467-018-03341-6 (PMC5864885; doi:10.1038/s41467-018-03341-6)
Supplement: Supplementary file 1 — Supplementary Information(PDF 10712 kb) [file 41467_2018_3341_MOESM1_ESM.pdf]

Ruthenium(II)-enabled *para*-Selective C-H Difluoromethylation of Anilides, Indolines and Tetrahydroquinolines

Yuan et al.

Supplementary Methods

Reagents: Unless otherwise noted, all reagents purchased from commercial suppliers and used without further purification. Column chromatography purifications performed using 200–300 mesh silica gel.

Instruments: NMR spectra recorded on Varian Inova–400 MHz, Inova–300 MHz, Bruker DRX–400 or Bruker DRX–500 instruments and calibrated using residual solvent peaks as internal reference. Multiplicities are recorded as: s = singlet, d = doublet, t = triplet, q = quartet, dd = doublet of doublets, dt = doublet of triplets, brs = broad singlet, m = multiplet. HRMS analysis carried out using a Bruker micrOTOF–Q instrument or a TOF–MS instrument.

General procedures for the preparation of *N*-phenylpivalamide. All the derivatives of *N*-Phenylpivalamides synthesized according to the previous reports [1].

Supplementary Table 1: Optimization for *para*-C-H difluoromethylation[a]

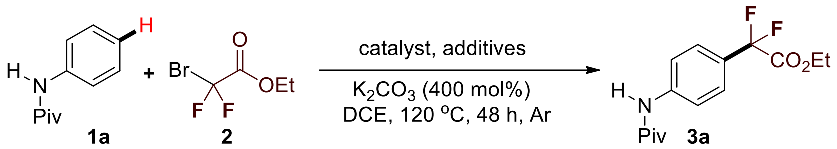

Entry	catalyst (5 mol%)	silver salt (10 mol%)	additive (20 mol%)	yield (%)[b]
1	$[RuCl_2(p\text{-cymene})]_2$	no	1-Ad-OH	45%
2	$[RuCl_2(p\text{-cymene})]_2$	no	KOAc	35%
3	$[RuCl_2(p\text{-cymene})]_2$	no	Piv-OH	39%
4	$[RuCl_2(p\text{-cymene})]_2$	no	MesCOOH	40%
5	$[RuCl_2(p\text{-cymene})]_2$	no	Piv-Val-OH	32%
6	$[RuCl_2(p\text{-cymene})]_2$	$AgBF_4$	1-Ad-OH	51%
7	$[RuCl_2(p\text{-cymene})]_2$	$AgSbF_6$	1-Ad-OH	48%
8	$[RuCl_2(p\text{-cymene})]_2$	$AgNTf_2$	1-Ad-OH	65%
9	$[RuCl_2(p\text{-cymene})]_2$	$AgNTf_2$	1-Ad-OH	87%[c]
10	$RuCl_3$	$AgNTf_2$	1-Ad-OH	21%[c]
11	$Pd(PPh_3)_4$	no	1-Ad-OH	trace
12	Cu_2O	no	1-Ad-OH	trace
13	$Ni(acac)_2$	no	1-Ad-OH	trace
14	no	$AgNTf_2$	1-Ad-OH	0%

[a]Reaction condition: **1a** (0.20 mmol, 1.0 equiv), **2** (3 equiv), K_2CO_3 (4 equiv), catalyst (5 mol %), additive (20 mol %), and Ag salt (10 mol %) in DCE (0.5 mL) for 48 h at 120 °C under argon in a sealed tube. [b]GC yield using biphenyl as the internal standard. [c]Ag salt (20 mol %).

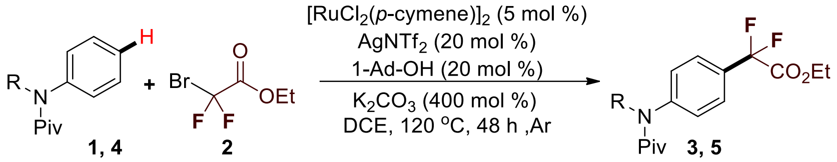

Supplementary Figure 1. Procedures for preparation of 3 and 5

General Procedure for *para*-Difluoromethylation of Anilides, Indolines and Quinolines: A mixture of **1 or 4** (0.2 mmol, 1.0 equiv), $BrCF_2CO_2Et$ (80 µL, 121.2 mg, 3 equiv), $[RuCl_2(p\text{-cymene})]_2$ (6 mg, 5 mol %), K_2CO_3 (108.8 mg, 400 mol %), 1-Ad-OH (7.2 mg, 20 mol %), $AgNTf_2$ (14.4 mg, 20 mol %) and DCE (0.5 mL) in a 15 mL glass vial sealed under argon atmosphere heated at 120 °C for 48 hours. The reaction mixture cooled to room temperature and concentrated *in vacuo*. The resulting residue was purified by column chromatography (PE / EA = 10 : 1) on silica gel to give the product **3 or 5**.

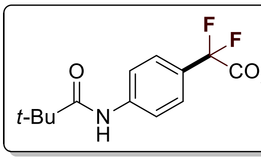

3a

White solid. Isolated yield: 50.2 mg, 84%.

^{1}H NMR (400 MHz, $CDCl_3$) δ 7.61 (d, *J* = 8.6 Hz, 3H), 7.50 (d, *J* = 8.7 Hz, 2H), 4.25 (q, *J* = 7.1 Hz, 2H), 1.31 – 1.23 (m, 12H).

^{13}C NMR (101 MHz, $CDCl_3$) δ 177.11, 164.30 (t, $J_{C\text{-}F}$ = 35.7 Hz), 140.68, 128.10 (t, $J_{C\text{-}F}$ = 26.1 Hz), 126.34 (t, $J_{C\text{-}F}$ = 6.1 Hz), 119.88, 113.36 (t, $J_{C\text{-}F}$ = 251.9 Hz), 63.20, 39.80, 27.54, 13.90.

^{19}F NMR (376 MHz, $CDCl_3$) δ -103.34.

HRMS Calcd for $C_{15}H_{20}F_2NO_3$ $[M+H^+]$: 300.1411; Found: 300.1417.

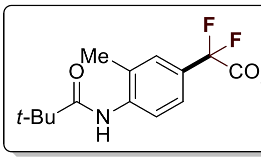

3b

White solid. Isolated yield: 48.8 mg, 78%.

^{1}H NMR (400 MHz, $CDCl_3$) δ 8.10 (d, *J* = 8.4 Hz, 1H), 7.45 (d, *J* = 8.6 Hz, 1H), 7.42 (s, 1H), 7.33 (brs, 1H), 4.28 (q, *J* = 7.1 Hz, 2H), 2.30 (s, 3H), 1.34 (s, 9H), 1.29 (t, *J* = 7.1 Hz, 3H).

^{13}C NMR (101 MHz, $CDCl_3$) δ 176.70, 164.42 (t, $J_{C\text{-}F}$ = 35.8 Hz), 138.60, 128.61 (t, $J_{C\text{-}F}$ = 25.8 Hz), 128.07, 127.51 (t, $J_{C\text{-}F}$ = 6.1 Hz), 124.46 (t, $J_{C\text{-}F}$ = 6.2 Hz), 122.00, 113.42 (t, $J_{C\text{-}F}$ = 250.5 Hz), 63.24, 40.11, 27.79, 17.78, 14.02.

^{19}F NMR (376 MHz, $CDCl_3$) δ -162.94.

HRMS Calcd for $C_{16}H_{22}F_2NO_3$ $[M+H^+]$: 314.1568; Found: 314.1556.

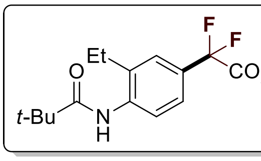

3c

White solid. Isolated yield: 48.2 mg, 76%.

^{1}H NMR (400 MHz, $CDCl_3$) δ 8.12 (d, *J* = 8.4 Hz, 1H), 7.61 – 7.34 (m, 3H), 4.28 (q, *J* = 7.1 Hz, 2H), 2.63 (q, *J* = 7.6 Hz, 2H), 1.34 (s, 9H), 1.29 (dd, *J* = 14.3, 7.3 Hz, 6H).

^{13}**C NMR** (101 MHz, $CDCl_3$) δ 176.70, 164.41(t, $J_{C\text{-}F}$ = 36.0 Hz), 138.00, 133.76, 128.83 (t, $J_{C\text{-}F}$ = 25.8 Hz), 125.68 (t, $J_{C\text{-}F}$ = 6.1 Hz), 124.38 (t, $J_{C\text{-}F}$ = 6.1 Hz), 122.42, 113.50 (t, $J_{C\text{-}F}$ = 252.0 Hz), 63.22, 40.11, 27.77, 24.55, 14.02, 13.63.

^{19}**F NMR** (376 MHz, $CDCl_3$) δ -103.51.

HRMS Calcd for $C_{17}H_{24}F_2NO_3$ [M+H$^+$]: 328.1724; Found: 328.1719.

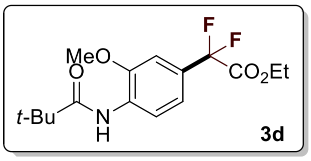

White solid. Isolated yield: 48.0 mg, 73%.

^{1}**H NMR** (400 MHz, $CDCl_3$) δ 8.50 (d, J = 8.5 Hz, 1H), 8.18 (brs, 1H), 7.20 (dd, J = 8.5, 1.4 Hz, 1H), 7.09 (d, J = 1.8 Hz, 1H), 4.29 (q, J = 7.1 Hz, 2H), 3.94 (s, 3H), 1.34 – 1.27 (m, 12H).

^{13}**C NMR** (101 MHz, $CDCl_3$) δ 176.95, 164.40 (t, $J_{C\text{-}F}$ = 35.8 Hz), 147.93, 130.44, 127.50 (t, $J_{C\text{-}F}$ = 26.0 Hz), 119.26, 118.87 (t, $J_{C\text{-}F}$ = 6.4 Hz), 113.41 (t, $J_{C\text{-}F}$ = 252.2 Hz), 106.88 (t, $J_{C\text{-}F}$ = 6.3 Hz), 63.25, 56.25, 40.28, 27.71, 14.02.

^{19}**F NMR** (376 MHz, $CDCl_3$) δ -103.31.

HRMS Calcd for $C_{16}H_{22}F_2NO_4$ [M+H$^+$]: 330.1517; Found: 330.1524.

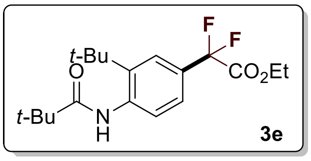

White solid. Isolated yield: 39.8 mg, 56%.

^{1}**H NMR** (400 MHz, $CDCl_3$) δ 7.91 (d, J = 8.4 Hz, 1H), 7.61 (d, J = 1.9 Hz, 1H), 7.56 (brs, 1H), 7.46 (dd, J = 8.5, 1.8 Hz, 1H), 4.29 (q, J = 7.1 Hz, 2H), 1.44 (s, 9H), 1.35 (s, 9H), 1.31 (t, J = 7.1 Hz, 3H).

^{13}**C NMR** (101 MHz, $CDCl_3$) δ 176.41, 164.38 (t, $J_{C\text{-}F}$ = 35.6 Hz), 141.34, 138.42, 129.26 (t, $J_{C\text{-}F}$ = 25.5 Hz), 126.52, 124.29 (t, $J_{C\text{-}F}$ = 6.0 Hz), 123.87 (t, $J_{C\text{-}F}$ = 6.3 Hz), 113.59 (t, $J_{C\text{-}F}$ = 252.0 Hz), 63.23, 39.84, 34.73, 30.54, 27.77, 14.03.

^{19}**F NMR** (376 MHz, $CDCl_3$) δ -103.35.

HRMS Calcd for $C_{19}H_{28}F_2NO_3$ [M+H$^+$]: 356.2037; Found: 356.2033.

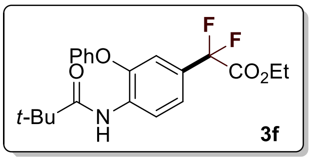

Green oil. Isolated yield: 62.7 mg, 80%.

^{1}**H NMR** (400 MHz, $CDCl_3$) δ 8.58 (d, J = 8.6 Hz, 1H), 8.11 (brs, 1H), 7.44 – 7.33 (m, 3H), 7.18 (t, J = 7.4 Hz, 1H), 7.13 (d, J = 1.9 Hz, 1H), 7.05 – 6.96 (m, 2H), 4.26 (q, J = 7.1 Hz, 2H), 1.29 – 1.21 (m, 12H).

^{13}**C NMR** (101 MHz, $CDCl_3$) δ 177.02, 164.06 (t, $J_{C\text{-}F}$ = 35.6 Hz), 155.95, 145.47, 132.54, 130.35, 128.04 (t, $J_{C\text{-}F}$ = 26.4 Hz), 124.57, 121.71 (t, $J_{C\text{-}F}$ = 6.2 Hz), 120.67, 118.39, 115.25 (t, $J_{C\text{-}F}$ = 6.3 Hz), 113.00 (t, $J_{C\text{-}F}$ = 250.5 Hz), 63.26, 40.21, 27.55, 13.96 .

^{19}**F NMR** (376 MHz, $CDCl_3$) δ -103.21.

HRMS Calcd for $C_{21}H_{24}F_2NO_4$ [M+H$^+$]: 392.1673; Found: 392.1662.

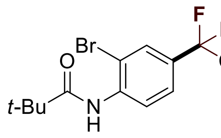

3g

White solid. Isolated yield: 28.7 mg, 38%.

^{1}H NMR (400 MHz, $CDCl_3$) δ 8.54 (d, *J* = 8.7 Hz, 1H), 8.13 (brs, 1H), 7.79 (d, *J* = 1.9 Hz, 1H), 7.55 (dd, *J* = 8.7, 1.7 Hz, 1H), 4.30 (dd, *J* = 14.3, 7.2 Hz, 2H), 1.36 (s, 9H), 1.31 (t, *J* = 7.1 Hz, 3H).

^{13}C NMR (101 MHz, $CDCl_3$) δ 177.00, 163.88 (t, $J_{C\text{-}F}$ = 35.4 Hz), 137.29, 128.64 (t, $J_{C\text{-}F}$ = 26.6 Hz), 126.34 (t, $J_{C\text{-}F}$ = 6.5 Hz), 125.38 (t, $J_{C\text{-}F}$ = 6.0 Hz), 122.92, 121.02, 112.64 (t, $J_{C\text{-}F}$ = 252.9 Hz), 63.47, 40.49, 27.63, 14.00.

^{19}F NMR (376 MHz, $CDCl_3$) δ -103.60.

HRMS Calcd for $C_{15}H_{19}BrF_2NO_3$ [M+H$^+$]: 378.0516; Found: 378.0521.

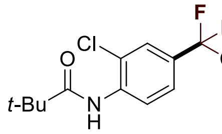

3h

White solid. Isolated yield: 32.0 mg, 48%.

^{1}H NMR (400 MHz, $CDCl_3$) δ 8.56 (d, *J* = 8.7 Hz, 1H), 8.12 (brs, 1H), 7.64 (d, *J* = 1.9 Hz, 1H), 7.51 (dd, *J* = 8.7, 1.9 Hz, 1H), 4.30 (q, *J* = 7.1 Hz, 2H), 1.35 (s, 9H), 1.31 (t, *J* = 7.1 Hz, 3H).

^{13}C NMR (101 MHz, $CDCl_3$) δ 177.05, 163.89 (t, $J_{C\text{-}F}$ = 35.4 Hz), 138.39, 129.48 (t, $J_{C\text{-}F}$ = 6.5 Hz), 129.03 (t, $J_{C\text{-}F}$ = 26.6 Hz), 126.04 (t, $J_{C\text{-}F}$ = 6.0 Hz), 121.14, 113.34, 112.52 (t, $J_{C\text{-}F}$ = 251.5 Hz), 63.48, 40.51, 27.66, 14.01.

^{19}F NMR (376 MHz, $CDCl_3$) δ -103.64.

HRMS Calcd for $C_{15}H_{19}ClF_2NO_3$ [M+H$^+$]: 334.1022; Found: 334.1027.

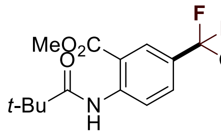

3i

White solid. Isolated yield: 40.7 mg, 57%.

^{1}H NMR (400 MHz, $CDCl_3$) δ 8.14 (d, *J* = 1.8 Hz, 1H), 7.90 (dd, *J* = 8.6, 2.2 Hz, 1H), 7.79 (d, *J* = 8.6 Hz, 1H), 7.52 (brs, 1H), 4.32 (q, *J* = 7.1 Hz, 2H), 3.86 (s, 3H), 1.33 (s, 9H), 1.29 (t, *J* = 7.1 Hz, 3H).

^{13}C NMR (101 MHz, $CDCl_3$) δ 177.10, 166.27, 140.33, 129.75 (d, $J_{C\text{-}F}$ = 3.6 Hz), 128.90, 128.60, 128.08 (t, $J_{C\text{-}F}$ = 10.3 Hz), 122.72, 121.96, 113.18, 62.87, 52.67, 39.98, 27.67, 14.02.

^{19}F NMR (376 MHz, $CDCl_3$) δ -98.62.

HRMS Calcd for $C_{17}H_{22}F_2NO_5$ [M+H$^+$]: 358.1466; Found: 358.1459.

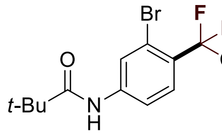

3j

White solid. Isolated yield: 36.2 mg, 48%.

^{1}H NMR (400 MHz, $CDCl_3$) δ 8.08 – 7.97 (m, 1H), 7.65 (d, *J* = 8.6 Hz, 1H), 7.53 (dd, *J* = 8.6, 2.1 Hz, 1H), 7.42 (brs, 1H), 4.34 (q, *J* = 7.1 Hz, 2H), 1.34 – 1.29 (m, 12H).

^{13}C NMR (101 MHz, $CDCl_3$) δ 177.03, 163.24 (t, $J_{C\text{-}F}$ = 34.4 Hz), 141.27, 128.29 (t, $J_{C\text{-}F}$ = 8.9 Hz), 124.76, 120.92 (t, $J_{C\text{-}F}$ = 4.3 Hz), 118.10, 115.33, 112.84, 63.51, 39.99, 27.64, 13.95.

^{19}F NMR (376 MHz, $CDCl_3$) δ -101.06.

HRMS Calcd for $C_{15}H_{19}BrF_2NO_3$ [M+H$^+$]: 378.0516; Found: 378.0520.

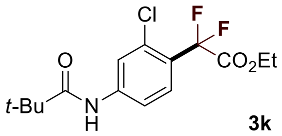

3k

White solid. Isolated yield: 41.9 mg, 63%.

^{1}H NMR (400 MHz, $CDCl_3$) δ 7.89 – 7.84 (m, 1H), 7.65 (d, *J* = 8.6 Hz, 1H), 7.44 (dd, *J* = 8.6, 2.0 Hz, 2H), 4.34 (q, *J* = 7.1 Hz, 2H), 1.36 – 1.28 (m, 12H).

^{13}C NMR (101 MHz, $CDCl_3$) δ 177.03, 163.33 (t, $J_{C\text{-}F}$ = 34.3 Hz), 141.36, 132.78, 127.99 (t, $J_{C\text{-}F}$ = 8.5 Hz), 126.55 (t, $J_{C\text{-}F}$ = 24.9 Hz), 121.47, 117.57, 112.29 (t, $J_{C\text{-}F}$ = 250.4 Hz), 63.47, 40.00, 27.63, 13.95.

^{19}F NMR (376 MHz, $CDCl_3$) δ -101.67.

HRMS Calcd for $C_{15}H_{19}ClF_2NO_3$ [M+H$^+$]: 334.1022; Found: 334.1028.

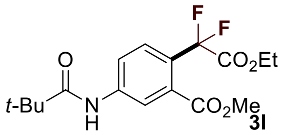

3l

White solid. Isolated yield: 36.4 mg, 51%.

^{1}H NMR (400 MHz, $CDCl_3$) δ 8.14 (s, 1H), 7.91 (dd, *J* = 8.6, 2.1 Hz, 1H), 7.80 (d, *J* = 8.6 Hz, 1H), 7.51 (brs, 1H), 4.32 (q, *J* = 7.1 Hz, 2H), 3.87 (s, 3H), 1.34 (s, 9H), 1.30 (t, *J* = 7.1 Hz, 3H).

^{13}C NMR (101 MHz, $CDCl_3$) δ 177.09, 166.26, 140.32, 129.77, 128.92 (t, $J_{C\text{-}F}$ = 5.4 Hz), 128.66 (t, $J_{C\text{-}F}$ = 5.0 Hz), 128.08 (t, $J_{C\text{-}F}$ = 10.2 Hz), 122.72, 121.95, 113.18 (t, $J_{C\text{-}F}$ = 269.8 Hz), 62.87, 52.68, 39.99, 27.67, 14.03.

^{19}F NMR (376 MHz, $CDCl_3$) δ -98.64.

HRMS Calcd for $C_{17}H_{22}F_2NO_5$ [M+H$^+$]: 358.1466; Found: 358.1465.

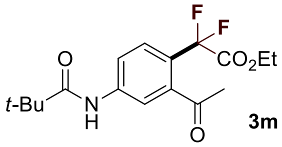

3m

White solid. Isolated yield: 28.7 mg, 42%.

^{1}H NMR (400 MHz, $CDCl_3$) δ 8.90 (s, 1H), 8.57 (brs, 1H), 7.77 (d, *J* = 8.2 Hz, 1H), 7.67 (d, *J* = 8.3 Hz, 1H), 4.32 (q, *J* = 7.1 Hz, 2H), 2.63 (s, 3H), 1.36 (s, 9H), 1.30 (t, *J* = 7.1 Hz, 3H).

^{13}C NMR (101 MHz, $CDCl_3$) δ 197.52, 177.45, 164.34 (t, $J_{C\text{-}F}$ = 34.4 Hz), 140.05, 136.96 (t, $J_{C\text{-}F}$ = 3.0 Hz), 126.74 (t, $J_{C\text{-}F}$ = 8.7 Hz), 125.37 (t, $J_{C\text{-}F}$ = 23.9 Hz), 124.18, 123.20, 113.23 (t, $J_{C\text{-}F}$ = 253.7 Hz), 64.32, 40.18, 27.39, 27.03, 13.93.

^{19}F NMR (376 MHz, $CDCl_3$) δ -104.19.

HRMS Calcd for $C_{17}H_{22}F_2NO_4$ [M+H$^+$]: 342.1517; Found: 342.1523.

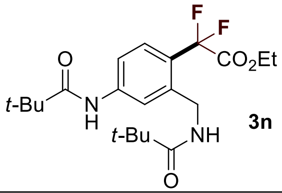

3n

White solid. Isolated yield: 49.4 mg, 60%.

^{1}H NMR (400 MHz, $CDCl_3$) δ 9.80 (brs, 1H), 8.06 (d, *J* = 8.6 Hz, 1H), 7.51 (d, *J* = 8.7 Hz, 1H), 7.45 (s, 1H), 6.34 (brs, 1H), 4.39 – 4.16 (m, 4H), 1.38 (s, 9H), 1.29 (t, *J* = 7.1 Hz, 3H), 1.17 (s, 9H).

^{13}C NMR (101 MHz, $CDCl_3$) δ 179.59, 178.49, 164.32 (t, J_{C-F} = 35.6 Hz), 139.67, 129.74, 128.43 (t, J_{C-F} = 26.1 Hz), 127.67 (t, J_{C-F} = 6.1 Hz), 125.88 (t, J_{C-F} = 5.9 Hz), 124.84, 113.32 (t, J_{C-F} = 252.0 Hz), 63.32, 40.42, 40.10, 38.70, 36.58, 27.57 (d, J_{C-F} = 4.4 Hz), 14.01.

^{19}F NMR (376 MHz, $CDCl_3$) δ -103.59.

HRMS Calcd for $C_{21}H_{32}F_2N_2O_4$ [M+H$^+$]: 413.2252; Found: 413.2257.

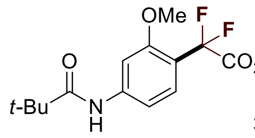

3o

White solid. Isolated yield: 44.7 mg, 68%.

^{1}H NMR (400 MHz, $CDCl_3$) δ 7.77 (s, 1H), 7.54 (d, *J* = 8.4 Hz, 1H), 7.45 (brs, 1H), 6.79 (dd, *J* = 8.4, 1.8 Hz, 1H), 4.31 (q, *J* = 7.1 Hz, 2H), 3.82 (s, 3H), 1.32 (s, 9H), 1.28 (t, *J* = 7.1 Hz, 3H).

^{13}C NMR (101 MHz, $CDCl_3$) δ 177.05, 163.99 (t, J_{C-F} = 34.4 Hz), 140.76, 138.38, 128.27 (t, J_{C-F} = 24.1 Hz), 127.13 (t, J_{C-F} = 8.9 Hz), 120.89, 116.77, 113.11 (t, J_{C-F} = 248.0 Hz), 63.19, 39.99, 27.72, 18.14, 13.99.

^{19}F NMR (376 MHz, $CDCl_3$) δ -102.05.

HRMS Calcd for $C_{16}H_{22}F_2NO_4$ [M+H$^+$]: 330.1517; Found:3 30.1516.

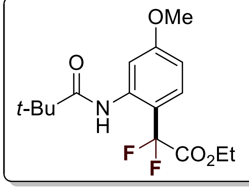

3o'

White solid. Isolated yield: 15.1 mg, 23%.

^{1}H NMR (400 MHz, $CDCl_3$) δ 8.57 (brs, 1H), 8.02 (d, *J* = 2.3 Hz, 1H), 7.43 (d, *J* = 8.8 Hz, 1H), 6.69 (dd, *J* = 8.8, 2.5 Hz, 1H), 4.30 (q, *J* = 7.1 Hz, 2H), 3.84 (s, 3H), 1.34 (s, 9H), 1.30 (t, *J* = 7.1 Hz, 3H).

^{13}C NMR (101 MHz, $CDCl_3$) δ 177.37, 165.07 (t, J_{C-F} = 35.9 Hz), 162.45, 138.11, 127.49 (t, J_{C-F} = 8.8 Hz), 113.93 (t, J_{C-F} = 251.5 Hz), 113.31 (t, J_{C-F} = 24.6 Hz), 110.87, 107.81, 63.87, 55.67, 40.27, 27.45, 14.01.

^{19}F NMR (376 MHz, $CDCl_3$) δ -102.29.

HRMS Calcd for $C_{16}H_{22}F_2NO_4$ [M+H$^+$]: 330.1517; Found: 330.1513.

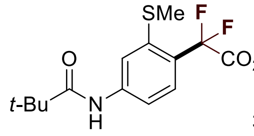

3p

White solid. Isolated yield: 35.2 mg, 51%.

^{1}H NMR (400 MHz, $CDCl_3$) δ 7.96 (d, *J* = 0.6 Hz, 1H), 7.60 (d, *J* = 8.5 Hz, 1H), 7.41 (brs, 1H), 7.26 – 7.22 (m, 1H), 4.33 (q, *J* = 7.1 Hz, 2H), 2.46 (s, 3H), 1.35 – 1.29 (m, 12H).

^{13}C NMR (101 MHz, $CDCl_3$) δ 177.05, 163.99, 140.76, 138.38, 128.27 (t, J_{C-F} = 24.1 Hz), 127.13 (t, J_{C-F} = 8.9 Hz), 120.89, 116.77, 113.11 (t, J_{C-F} = 248.0 Hz), 63.19, 39.99, 27.72, 18.14, 13.99.

^{19}F NMR (376 MHz, $CDCl_3$) δ -98.80.

HRMS Calcd for $C_{16}H_{22}F_2NO_3S$ [M+H$^+$]: 346.1288; Found: 346.1297.

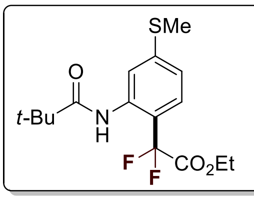

3p[6]

White solid. Isolated yield: 26.2 mg, 38%.

^{1}H NMR (400 MHz, $CDCl_3$) δ 8.53 (brs, 1H), 8.28 (s, 1H), 7.41 (d, *J* = 8.4 Hz, 1H), 7.00 (dd, *J* = 8.4, 1.5 Hz, 1H), 4.30 (q, *J* = 7.1 Hz, 2H), 2.50 (s, 3H), 1.33 (s, 9H), 1.30 (t, *J* = 7.1 Hz, 3H).

^{13}C NMR (101 MHz, $CDCl_3$) δ 177.30, 164.83 (t, J_{C-F} = 35.5 Hz), 144.59 (d, J_{C-F} = 1.4 Hz), 136.84, 128.55, 126.37 (t, J_{C-F} = 8.7 Hz), 121.09, 119.62, 117.32 (t, J_{C-F} = 24.4 Hz), 113.70 (t, J_{C-F} = 253.2 Hz), 64.00, 40.22, 27.43, 15.06, 13.98.

^{19}F NMR (376 MHz, $CDCl_3$) δ -103.12.

HRMS Calcd for $C_{16}H_{22}F_2NO_3S$ [M+H$^+$]: 346.1288; Found: 340.1298.

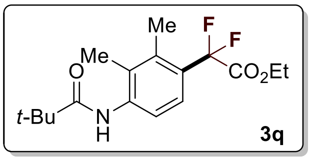

3q

White solid. Isolated yield: 37.3 mg, 57%.

^{1}H NMR (400 MHz, $CDCl_3$) δ 7.72 (d, *J* = 8.6 Hz, 1H), 7.47 (d, *J* = 8.6 Hz, 1H), 7.30 (brs, 1H), 4.32 – 4.27 (m, 2H), 2.30 (s, 3H), 2.16 (s, 3H), 1.35 (s, 9H), 1.29 (t, *J* = 7.1 Hz, 3H).

^{13}C NMR (101 MHz, $CDCl_3$) δ 176.79, 164.53 (t, J_{C-F} = 35.1 Hz), 137.99, 135.89 (t, J_{C-F} = 3.1 Hz), 129.92, 128.42 (t, J_{C-F} = 23.5 Hz), 124.40 (t, J_{C-F} = 9.5 Hz), 121.06, 114.30, 63.26, 39.92, 27.84, 16.67 (t, J_{C-F} = 2.8 Hz), 14.03, 13.90.

^{19}F NMR (376 MHz, $CDCl_3$) δ -99.47.

HRMS Calcd for $C_{17}H_{24}F_2NO_3$ [M+H$^+$]: 328.1724; Found: 328.1726.

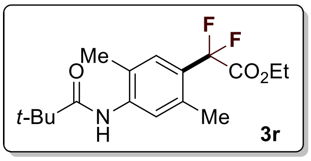

3r

White solid. Isolated yield: 34.7 mg, 53%.

^{1}H NMR (400 MHz, $CDCl_3$) δ 7.95 (s, 1H), 7.38 (s, 1H), 7.29 (brs, 1H), 4.29 (q, *J* = 7.1 Hz, 2H), 2.37 (s, 3H), 2.26 (s, 3H), 1.34 (s, 9H), 1.30 (t, *J* = 7.1 Hz, 3H).

^{13}C NMR (101 MHz, $CDCl_3$) δ 176.74, 164.38 (t, J_{C-F} = 35.4 Hz), 138.11, 135.52 (t, J_{C-F} = 3.2 Hz), 128.23 (t, J_{C-F} = 8.8 Hz), 126.94 (t, J_{C-F} = 23.6 Hz), 124.93, 124.84, 114.22 (t, J_{C-F} = 251.6 Hz), 63.22, 40.11, 27.80, 19.56 (t, J_{C-F} = 2.5 Hz), 17.25, 14.03.

^{19}F NMR (376 MHz, $CDCl_3$) δ -101.00.

HRMS Calcd for $C_{17}H_{24}F_2NO_3$ [M+H$^+$]: 328.1724; Found: 328.1725.

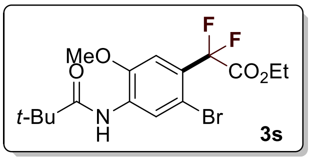

3s

White solid. Isolated yield: 39.1 mg, 48%.

^{1}H NMR (400 MHz, $CDCl_3$) δ 8.79 (s, 1H), 8.15 (brs, 1H), 7.20 (s, 1H), 4.34 (q, *J* = 7.1 Hz, 2H), 3.95 (s, 3H), 1.33 – 1.29 (m, 12H).

^{13}**C NMR** (101 MHz, $CDCl_3$) δ 177.00, 163.16 (t, J_{C-F} = 34.3 Hz), 146.84, 130.93, 127.01 (t, J_{C-F} = 24.4 Hz), 124.15, 112.82 (t, J_{C-F} = 249.5 Hz), 112.01 (t, J_{C-F} = 4.6 Hz), 108.83, 63.52, 56.41, 40.30, 27.62, 13.94.

^{19}**F NMR** (376 MHz, $CDCl_3$) δ -100.73.

HRMS Calcd for $C_{16}H_{21}BrF_2NO_4$ [M+H$^+$]: 408.0622; Found: 408.0626.

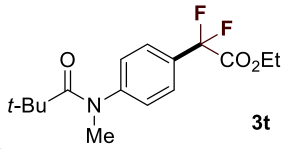

3t

White solid. Isolated yield: 44.4 mg, 71%.

^{1}**H NMR** (400 MHz, $CDCl_3$) δ 7.64 (d, *J* = 8.4 Hz, 2H), 7.31 (d, *J* = 8.4 Hz, 2H), 4.32 (q, *J* = 7.1 Hz, 2H), 3.23 (s, 3H), 1.31 (t, *J* = 7.1 Hz, 3H), 1.06 (s, 9H).

^{13}**C NMR** (101 MHz, $CDCl_3$) δ 178.23, 164.02, 148.07 (t, J_{C-F} = 1.9 Hz), 132.38 (t, J_{C-F} = 26.0 Hz), 129.09, 126.85, 113.15, 63.46, 41.33, 41.00, 29.55, 14.00.

^{19}**F NMR** (376 MHz, $CDCl_3$) δ -103.60.

HRMS Calcd for $C_{16}H_{22}F_2NO_3$ [M+H$^+$]: 314.1568; Found: 314.1577.

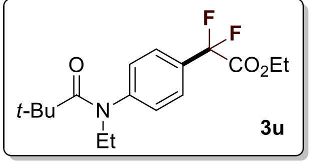

3u

White solid. Isolated yield: 43.8 mg, 67%.

^{1}**H NMR** (400 MHz, $CDCl_3$) δ 7.64 (d, *J* = 8.3 Hz, 2H), 7.28 (d, *J* = 8.3 Hz, 2H), 4.33 (dd, *J* = 14.3, 7.1 Hz, 2H), 3.66 (q, *J* = 7.1 Hz, 2H), 1.31 (t, *J* = 7.1 Hz, 3H), 1.10 (t, *J* = 7.0 Hz, 3H), 1.02 (s, 9H).

^{13}**C NMR** (101 MHz, $CDCl_3$) δ 177.34, 164.03, 146.22, 132.45 (t, J_{C-F} = 26.0 Hz), 130.17, 126.61 (t, J_{C-F} = 6.0 Hz), 113.18 (t, J_{C-F} = 253.6 Hz), 63.45, 47.85, 41.16, 29.63, 14.00, 12.88.

^{19}**F NMR** (376 MHz, $CDCl_3$) δ -103.52.

HRMS Calcd for $C_{17}H_{24}F_2NO_3$ [M+H$^+$]: 328.1724; Found: 328.1730.

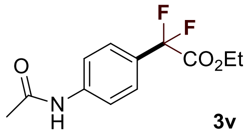

3v

Brown solid. Isolated yield: 38.1 mg, 74%.

^{1}**H NMR** (400 MHz, $CDCl_3$) δ 7.71 (s, 1H), 7.60 (d, *J* = 8.4 Hz, 2H), 7.53 (d, *J* = 8.6 Hz, 2H), 4.28 (q, *J* = 7.1 Hz, 2H), 2.18 (s, 3H), 1.29 (t, *J* = 7.1 Hz, 3H).

^{13}**C NMR** (101 MHz, $CDCl_3$) δ 168.90, 164.40 (t, J_{C-F} = 35.7 Hz), 140.53, 128.31 (t, J_{C-F} = 25.8 Hz), 126.56 (t, J_{C-F} = 6.1 Hz), 119.62, 113.38 (t, J_{C-F} = 252.0 Hz), 63.32, 24.72, 13.98.

^{19}**F NMR** (376 MHz, $CDCl_3$) δ -103.28.

HRMS Calcd for $C_{12}H_{14}F_2NO_3$ [M+H$^+$]: 258.0942; Found: 258.0951.

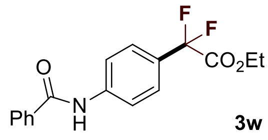

3w

White solid. Isolated yield: 19.8 mg, 31%.

^{1}H NMR (400 MHz, $CDCl_3$) δ 8.03 (s, 1H), 7.87 (d, *J* = 7.5 Hz, 2H), 7.75 (d, *J* = 8.4 Hz, 2H), 7.60 (d, *J* = 8.5 Hz, 2H), 7.56 (d, *J* = 7.2 Hz, 1H), 7.49 (t, *J* = 7.4 Hz, 2H), 4.30 (q, *J* = 7.1 Hz, 2H), 1.31 (t, *J* = 7.1 Hz, 3H).

^{13}C NMR (101 MHz, $CDCl_3$) δ 166.05, 164.35 (t, J_{C-F} = 35.7 Hz), 140.54, 134.62, 132.35, 129.03, 128.67 (t, J_{C-F} = 26.2 Hz), 127.20, 126.70 (t, J_{C-F} = 6.1 Hz), 120.02, 113.39 (t, J_{C-F} = 252.0 Hz), 63.32, 14.02.

^{19}F NMR (376 MHz, $CDCl_3$) δ -103.37.

HRMS Calcd for $C_{17}H_{16}F_2NO_3$ $[M+H^+]$: 320.1098; Found: 320.1102.

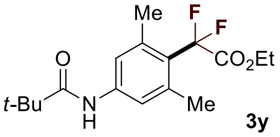

3y

White solid. Isolated yield: 41.2 mg, 63%.

^{1}H NMR (400 MHz, $CDCl_3$) δ 7.28 (s, 3H), 4.29 (q, *J* = 7.1 Hz, 2H), 2.43 (t, *J* = 4.2 Hz, 6H), 1.32 – 1.25 (m, 12H).

^{13}C NMR (101 MHz, $CDCl_3$) δ 176.98, 164.52 (t, J_{C-F} = 35.7 Hz), 139.24, 139.13 (t, J_{C-F} = 3.3 Hz), 125.48 (t, J_{C-F} = 22.8 Hz), 121.04, 116.08 (t, J_{C-F} = 253.1 Hz), 63.15, 39.87, 27.69, 21.94 (t, J_{C-F} = 5.9 Hz), 13.98.

^{19}F NMR (376 MHz, $CDCl_3$) δ -94.49 – -94.80 (m).

HRMS Calcd for $C_{17}H_{24}F_2NO_3$ $[M+H^+]$: 328.1724; Found: 328.1724.

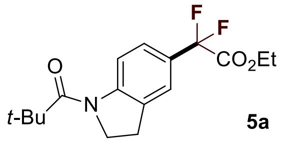

5a

White solid. Isolated yield: 53.3 mg, 82%.

^{1}H NMR (400 MHz, $CDCl_3$) δ 8.21 (d, *J* = 8.6 Hz, 1H), 7.56 (d, *J* = 8.6 Hz, 1H), 4.44 – 4.23 (m, 4H), 3.19 (t, *J* = 8.3 Hz, 2H), 1.37 (s, 9H), 1.31 (t, *J* = 7.1 Hz, 3H).

^{13}C NMR (101 MHz, $CDCl_3$) δ 177.36, 163.55 (t, J_{C-F} = 34.6 Hz), 148.22, 131.15, 127.80 (t, J_{C-F} = 4.1 Hz), 127.51 (t, J_{C-F} = 8.4 Hz), 125.71 (t, J_{C-F} = 24.4 Hz), 116.14, 112.56 (t, J_{C-F} = 250.6 Hz), 63.38, 49.57, 40.57, 28.81, 27.67, 13.97.

^{19}F NMR (376 MHz, $CDCl_3$) δ -100.88.

HRMS Calcd for $C_{17}H_{22}F_2NO_3$ $[M+H^+]$: 326.1568; Found: 326.1568.

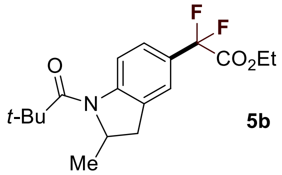

5b

White solid. Isolated yield: 50.2 mg, 74%.

^{1}H NMR (400 MHz, $CDCl_3$) δ 8.14 (d, *J* = 8.5 Hz, 1H), 7.44 (d, *J* = 8.2 Hz, 2H), 4.97 – 4.78 (m, 1H), 4.28 (q, *J* = 7.1 Hz, 2H), 3.32 (dd, *J* = 15.2, 7.6 Hz, 1H), 2.63 (d, *J* = 15.2 Hz, 1H), 1.37 (s, 9H), 1.30 (t, *J* = 7.1 Hz, 3H), 1.26 (d, *J* = 6.2 Hz, 3H).

^{13}C NMR (101 MHz, $CDCl_3$) δ 176.89, 164.57 (t, J_{C-F} = 35.9 Hz), 145.82, 131.49, 128.15 (t, J_{C-F} = 25.7 Hz), 125.26 (t, J_{C-F} = 6.2 Hz), 122.17 (t, J_{C-F} = 6.1 Hz), 119.39, 113.67 (t, J_{C-F} = 252.0 Hz), 63.19, 56.67, 40.93, 36.83, 28.58, 22.03, 14.03.

^{19}F NMR (376 MHz, $CDCl_3$) δ -102.75 (d, *J* = 11.9 Hz).

HRMS Calcd for $C_{18}H_{24}F_2NO_3$ [M+H$^+$]: 340.1724; Found: 340.1725.

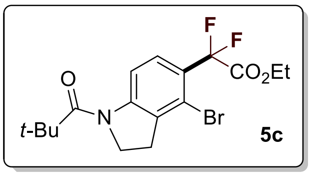

White solid. Isolated yield: 61.3 mg, 76%.

^{1}H NMR (400 MHz, $CDCl_3$) δ 8.25 (d, *J* = 8.6 Hz, 1H), 7.55 (d, *J* = 8.6 Hz, 1H), 4.33 (dt, *J* = 13.4, 7.7 Hz, 4H), 3.17 (t, *J* = 8.3 Hz, 2H), 1.37 (s, 9H), 1.31 (t, *J* = 7.1 Hz, 3H).

^{13}C NMR (101 MHz, $CDCl_3$) δ 177.44, 163.46 (t, J_{C-F} = 34.6 Hz), 147.74, 133.49, 127.71 (t, J_{C-F} = 8.8 Hz), 127.30 (t, J_{C-F} = 24.1 Hz), 116.97 (t, J_{C-F} = 4.1 Hz), 116.62, 113.08 (t, J_{C-F} = 250.7 Hz), 63.41, 49.20, 40.57, 31.21, 27.67, 13.97.

^{19}F NMR (376 MHz, $CDCl_3$) δ -100.28.

HRMS Calcd for $C_{17}H_{21}BrF_2NO_3$ [M+H$^+$]: 404.0673; Found: 404.0676.

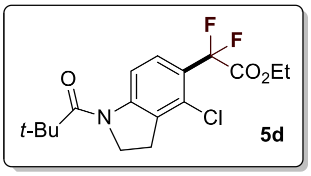

White solid. Isolated yield: 48.1 mg, 67%.

^{1}H NMR (400 MHz, $CDCl_3$) δ 8.21 (d, *J* = 8.6 Hz, 1H), 7.56 (d, *J* = 8.6 Hz, 1H), 4.44 – 4.23 (m, 4H), 3.19 (t, *J* = 8.3 Hz, 2H), 1.37 (s, 9H), 1.31 (t, *J* = 7.1 Hz, 3H).

^{13}C NMR (101 MHz, $CDCl_3$) δ 177.36, 163.55 (t, J_{C-F} = 34.6 Hz), 148.22, 131.15, 127.80 (t, J_{C-F} = 4.1 Hz), 127.51 (t, J_{C-F} = 8.4 Hz), 125.71 (t, J_{C-F} = 24.4 Hz), 116.14, 112.56 (t, J_{C-F} = 250.6 Hz), 63.38, 49.57, 40.57, 28.81, 27.67, 13.97.

^{19}F NMR (376 MHz, $CDCl_3$) δ -100.88.

HRMS Calcd for $C_{17}H_{21}ClF_2NO_3$ [M+H$^+$]: 360.1178; Found: 360.1177.

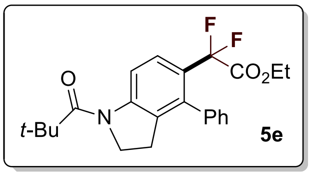

Green oil. Isolated yield: 51.3 mg, 64%.

^{1}H NMR (400 MHz, $CDCl_3$) δ 8.31 (d, *J* = 8.7 Hz, 1H), 3.92 (q, *J* = 7.1 Hz, 2H), 2.78 (t, *J* = 8.2 Hz, 2H), 1.37 (s, 9H), 1.13 (t, *J* = 7.1 Hz, 3H).

^{13}C NMR (101 MHz, CDCl$_3$) δ 177.26, 163.96, 146.66, 137.19 (t, $J_{C\text{-}F}$ = 3.5 Hz), 136.72, 131.58, 129.71, 128.18, 128.08, 126.54 (t, $J_{C\text{-}F}$ = 23.0 Hz), 126.07 (t, $J_{C\text{-}F}$ = 8.4 Hz), 117.07, 113.56 (t, $J_{C\text{-}F}$ = 245.5 Hz), 100.12, 62.86, 49.81, 40.51, 28.84, 27.78, 13.80.
^{19}F NMR (376 MHz, CDCl$_3$) δ -94.38.
HRMS Calcd for $C_{23}H_{26}F_2NO_3$ [M+H$^+$]: 402.1881; Found: 402.1883.

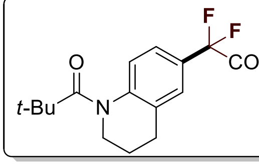

5f

White solid. Isolated yield: 48.1 mg, 71%.
^{1}H NMR (400 MHz, CDCl$_3$) δ 7.51 (d, J = 8.4 Hz, 1H), 7.35 (d, J = 9.4 Hz, 2H), 4.28 (q, J = 7.1 Hz, 2H), 3.92 – 3.70 (m, 2H), 2.84 (t, J = 7.1 Hz, 2H), 2.08 – 1.94 (m, 2H), 1.34 (s, 9H), 1.30 (t, J = 7.2 Hz, 3H).
^{13}C NMR (101 MHz, CDCl$_3$) δ 178.52, 164.38 (t, $J_{C\text{-}F}$ = 35.7 Hz), 143.12, 131.05, 128.75 (t, $J_{C\text{-}F}$ = 25.8 Hz), 126.77 – 125.77 (m), 122.72 (t, $J_{C\text{-}F}$ = 6.0 Hz), 113.43 (t, $J_{C\text{-}F}$ = 251.9 Hz), 63.21, 45.43, 40.35, 28.81, 26.30, 23.88, 13.99.
^{19}F NMR (376 MHz, CDCl$_3$) δ -103.57.
HRMS Calcd for $C_{18}H_{24}F_2NO_3$ [M+H$^+$]: 340.1724; Found: 340.1725.

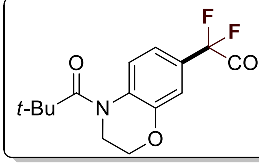

5g

White solid. Isolated yield: 23.1 mg, 34%.
^{1}H NMR (400 MHz, CDCl$_3$) δ 7.74 (d, J = 8.6 Hz, 1H), 7.13 (d, J = 2.0 Hz, 1H), 7.09 (dd, J = 8.6, 2.1 Hz, 1H), 4.37 – 4.32 (m, 2H), 4.28 (q, J = 7.1 Hz, 2H), 4.02 – 3.97 (m, 2H), 1.38 (s, 9H), 1.30 (t, J = 7.1 Hz, 3H).
^{13}C NMR (101 MHz, CDCl$_3$) δ 176.83, 164.23, 146.75, 129.80, 129.19, 125.94, 117.05 (t, $J_{C\text{-}F}$ = 6.0 Hz), 114.76 (t, $J_{C\text{-}F}$ = 6.5 Hz), 113.11 (t, $J_{C\text{-}F}$ = 251.0 Hz), 66.39, 63.26, 44.24, 40.02, 28.53, 14.02.
^{19}F NMR (376 MHz, CDCl$_3$) δ -103.76.
HRMS Calcd for $C_{17}H_{22}F_2NO_4$ [M+H$^+$]: 342.1517; Found: 342.1514.

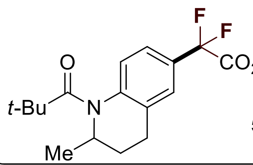

5h

White solid. Isolated yield: 48.7 mg, 69%.
^{1}H NMR (400 MHz, CDCl$_3$) δ 7.38 (d, J = 5.5 Hz, 3H), 4.71 – 4.67 (m, 1H), 4.30 (q, J = 7.1 Hz, 2H), 2.85 – 2.59 (m, 2H), 2.28 – 2.19 (m, 1H), 1.67 – 1.57 (m, 1H), 1.30 (t, J = 7.1 Hz, 3H), 1.27 (s, 9H), 1.10 (d, J = 6.6 Hz, 3H).
^{13}C NMR (101 MHz, CDCl$_3$) δ 178.69, 164.41, 164.06, 140.70, 132.76, 129.31, 127.70, 125.41 (t, $J_{C\text{-}F}$ = 6.2 Hz), 123.00 (t, $J_{C\text{-}F}$ = 6.0 Hz), 63.26, 49.35, 41.01, 30.35, 29.14, 24.39, 18.97, 14.02.
^{19}F NMR (376 MHz, CDCl$_3$) δ -103.36 (d, J = 8.5 Hz).

HRMS Calcd for $C_{19}H_{26}F_2NO_3$ [M+H$^+$]: 354.1881; Found: 354.1890.

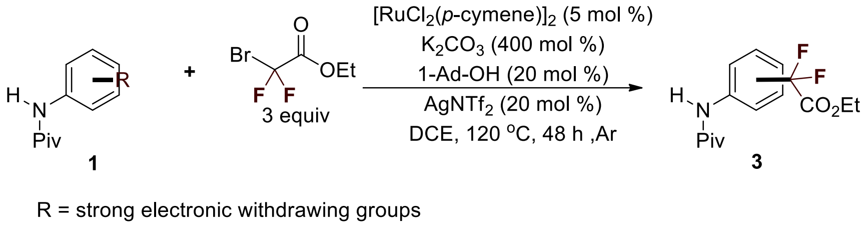

Supplementary Figure 2. More examples with electron-withdrawing groups substituted pivaloyl amide

Note: When too strong electronic withdrawing groups (F, CF_3, NO_2) were substituted on the aromatic ring, the directing ability of *ortho*-cycloruthenation was suppressed, which caused a different site selectivity.

General procedure as above.

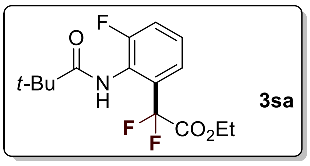

White solid. Isolated yield: 32.3 mg, 51%.

^{1}H NMR (400 MHz, $CDCl_3$) δ 8.52 (t, *J* = 8.3 Hz, 1H), 7.73 (brs, 1H), 7.44 – 7.35 (m, 2H), 4.32 (q, *J* = 7.1 Hz, 2H), 1.36 (s, 9H), 1.32 (t, *J* = 7.2 Hz, 3H).

^{13}C NMR (101 MHz, $CDCl_3$) δ 176.97, 163.90 (t, $J_{C\text{-}F}$ = 35.4 Hz), 153.14, 150.71, 132.10, 129.37 (dd, $J_{C\text{-}F}$ = 9.5, 1.5 Hz), 124.25 (d, $J_{C\text{-}F}$ = 3.8 Hz), 122.28 (td, $J_{C\text{-}F}$ = 6.2, 3.9 Hz), 121.40 (d, $J_{C\text{-}F}$ = 1.1 Hz), 112.60 (t, $J_{C\text{-}F}$ = 6.2 Hz), 112.38 (t, $J_{C\text{-}F}$ = 6.4 Hz), 63.45, 40.29, 27.63, 14.01.

^{19}F NMR (376 MHz, $CDCl_3$) δ -103.47, -125.70 – -133.69 (m).

HRMS Calcd for $C_{15}H_{19}F_3NO_3$ [M+H$^+$]: 318.1317; Found: 318.1318.

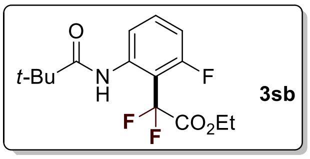

White solid. Isolated yield: 40.0 mg, 63%.

^{1}H NMR (400 MHz, $CDCl_3$) δ 7.76 – 7.68 (m, 1H), 7.54 (t, *J* = 8.3 Hz, 1H), 7.48 (brs, 1H), 7.18 (dd, *J* = 8.5, 1.8 Hz, 1H), 4.34 (q, *J* = 7.1 Hz, 2H), 1.33 – 1.29 (m, 12H).

^{13}C NMR (101 MHz, $CDCl_3$) δ 176.42, 162.83 (t, $J_{C\text{-}F}$ = 34.3 Hz), 160.85 (t, $J_{C\text{-}F}$ = 4.6 Hz), 158.36 (t, $J_{C\text{-}F}$ = 5.0 Hz), 141.81 (d, $J_{C\text{-}F}$ = 11.5 Hz), 126.91 (td, $J_{C\text{-}F}$ = 7.0, 3.6 Hz), 115.50 (q, $J_{C\text{-}F}$ = 13.2 Hz), 114.12 (d, $J_{C\text{-}F}$ = 3.2 Hz), 111.06 (t, $J_{C\text{-}F}$ = 250.8 Hz), 107.21 (d, $J_{C\text{-}F}$ = 26.2 Hz), 62.85, 39.38, 26.98, 13.33.

^{19}F NMR (376 MHz, $CDCl_3$) δ -101.42 (d, *J* = 7.9 Hz), -112.25 (dt, *J* = 21.8, 7.9 Hz).

HRMS Calcd for $C_{15}H_{19}F_3NO_3$ [M+H$^+$]: 318.1317; Found: 318.1319.

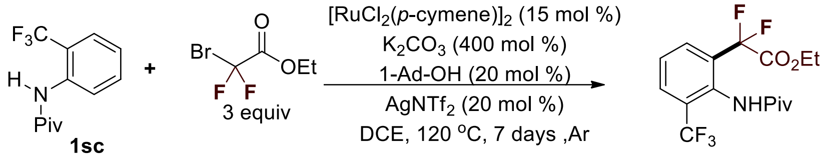

Supplementary Figure 3.

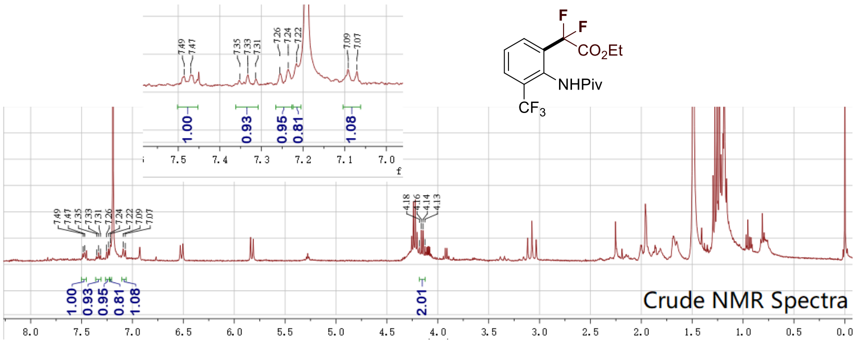

Supplementary Figure 4. ^{1}H NMR analysis of 1sc performed in standard condition

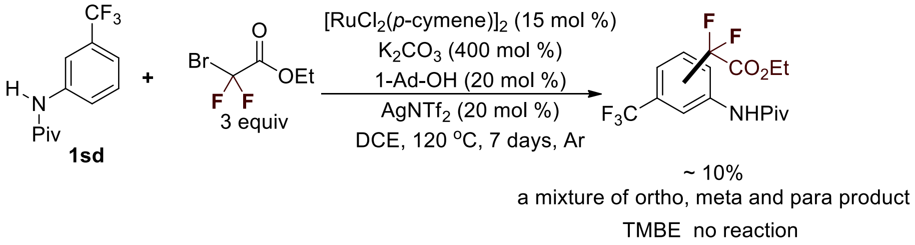

Supplementary Figure 5.

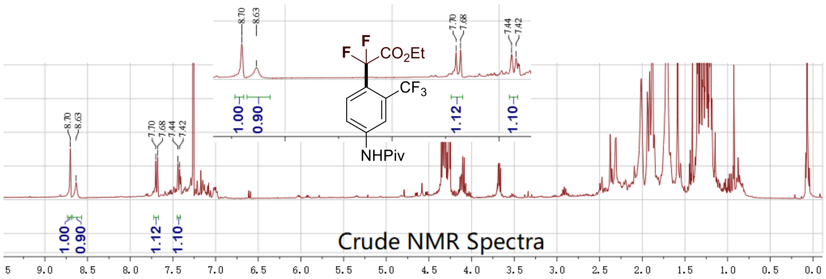

Supplementary Figure 6. ^{1}H NMR analysis of 1sd performed in standard condition

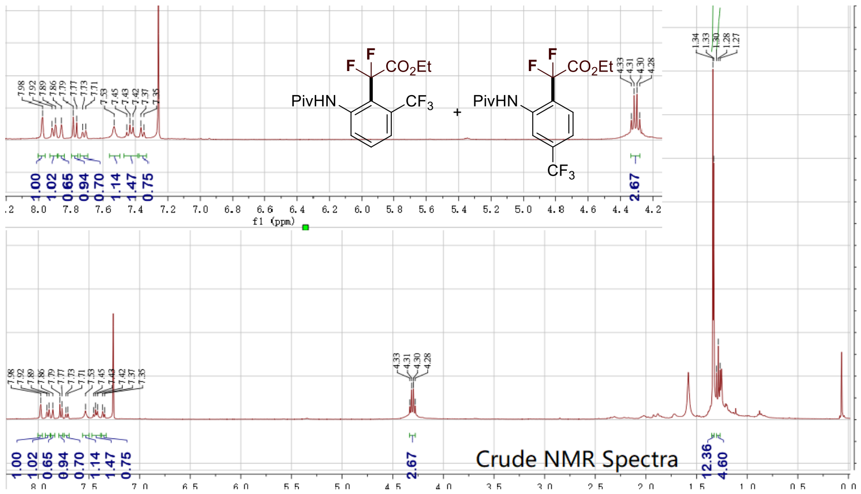

Supplementary Figure 7.

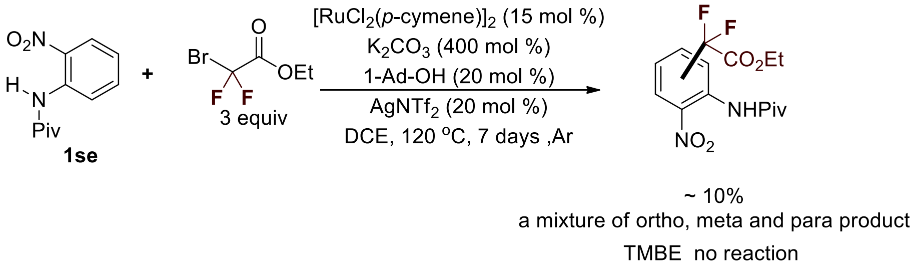

Supplementary Figure 8.

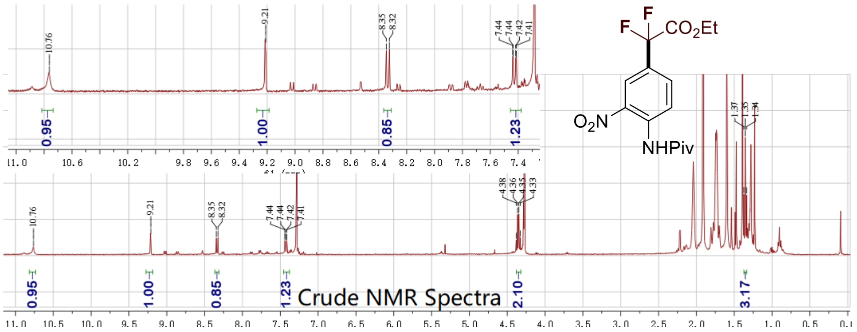

Supplementary Figure 9. ^{1}H NMR analysis of 1se performed in standard condition

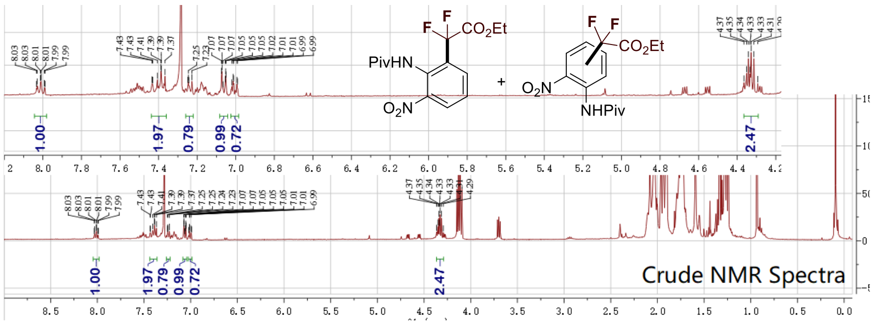

Supplementary Figure 10. ^{1}H NMR analysis of 1se performed in standard condition

Supplementary Table 2. Investigation on monofluoromethylation and non-fluoromethylation

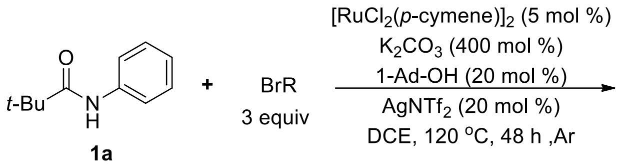

BrR	$BrCFHCO_2Et$	$BrCH_2CO_2Et$	$BrCMe_2CO_2Et$
**120 °C**	n.r.	trace	n.r.
**150 °C**	n.r.	trace	n.r.
**N-methyl-N-phenylpivalamide instead of 1a**	n.r.	n.r.	n.r.
**4a instead of 1a**	n.r.	n.r.	n.r.
**1,4-dioxane instead of DCE**	n.r.	n.r.	n.r.
**Toluene instead of DCE**	n.r.	n.r.	n.r.
**DMF instead of DCE**	n.r.	n.r.	n.r.
**HFIP instead of DCE**	n.r.	n.r.	n.r.
**PPh_3 (20 mol %) added**	n.r.	n.r.	n.r.
**PPh_3 (20 mol %) added / 1,4-dioxane instead of DCE**	n.r.	trace	n.r.
**$Pd(PPh_3)_4$ (10 mol %) added**	n.r.	trace	n.r.
**$Ba(OAc)_2$ (15 mol %) instead of 1-Ad-OH**	n.r.	trace	n.r.
**$Pd(PPh_3)_4$ (10 mol %) added $Ba(OAc)_2$ (15 mol %) instead of 1-Ad-OH 1,4-dioxane instead of DCE**	n.r.	trace	n.r.
**$Pd(OAc)_2$ (10 mol %) instead of $[RuCl_2(p\text{-cymene})]_2$**	n.r.	n.r.	n.r.
**$Ni(PPh_3)_2Cl_2$ (10 mol %) instead of $[RuCl_2(p\text{-cymene})]_2$**	n.r.	n.r.	n.r.

A variety of reactions carried out to explore the possibility for monofluoromethylation and non-fluoromethylation. However, the corresponding products only be observed in GC-Ms in less than 10% yields.

Note: When we replaced TMBE (methyl tert-butyl ether) of DCE as a solvent. A little para product obtained. We tried to perform this reaction under a harsh condition, the corresponding products could be provided.

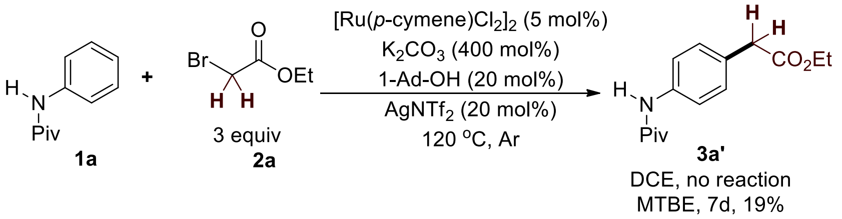

Supplementary Figure 11.

General procedure as above.

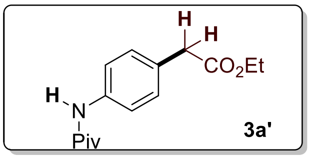

Colorless oil. Isolated yield: 10.1 mg, 19%.

¹H NMR (400 MHz, $CDCl_3$) δ 7.48 (d, *J* = 8.5 Hz, 2H), 7.30 (s, 1H), 7.24 (d, *J* = 8.5 Hz, 2H), 4.16 – 4.11 (m, 2H), 3.57 (s, 2H), 1.31 (s, 9H), 1.24 (s, 3H).

¹³C NMR (101 MHz, $CDCl_3$) δ 176.66, 171.73, 137.12, 130.08, 129.92, 120.19, 61.01, 40.99, 39.74, 27.77, 14.31.

HRMS Calcd for $C_{15}H_{22}NO_3$ [M+H⁺]: 264.1600; Found: 264.1603.

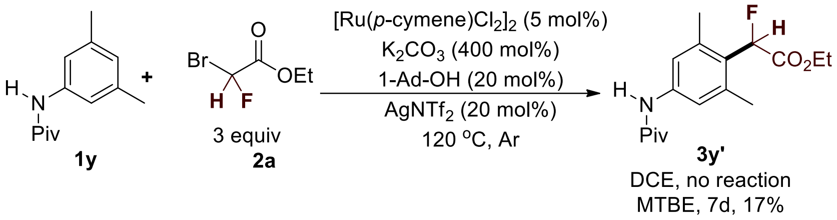

Supplementary Figure 12.

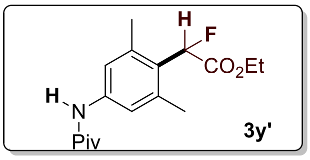

Colorless oil. Isolated yield: 10.5 mg, 17%.

¹H NMR (400 MHz, $CDCl_3$) δ 7.27 (s, 2H), 6.12 (d, *J* = 46.0 Hz, 1H), 4.30 – 4.17 (m, 2H), 2.38 (d, *J* = 2.2 Hz, 6H), 1.30 (s, 9H), 1.22 (d, *J* = 7.1 Hz, 3H).

¹³C NMR (101 MHz, $CDCl_3$) δ 176.89, 169.39 (d, J_{C-F} = 27.9 Hz), 138.97 (d, J_{C-F} = 3.1 Hz), 138.70 (d, J_{C-F} = 2.3 Hz), 127.25 (d, J_{C-F} = 17.9 Hz), 120.01 (d, J_{C-F} = 1.3 Hz), 85.91 (d, J_{C-F} = 183.6 Hz), 61.89, 39.82, 27.71, 20.22 (d, J_{C-F} = 1.7 Hz), 14.20.

¹⁹F NMR (376 MHz, $CDCl_3$) δ -182.67.

HRMS Calcd for $C_{17}H_{25}FNO_3$ [M+H⁺]: 310.1818; Found: 310.1823.

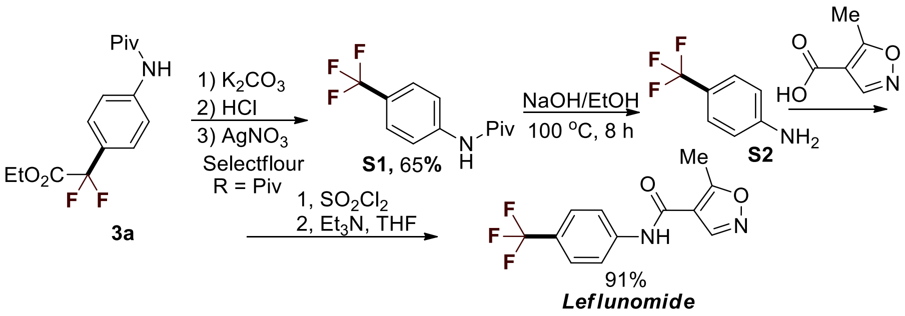

Supplementary Figure 13. Synthesis of Leflunomide

The procedure for synthesis of S1[2]**:** In a 15 mL glass vial, a mixture of **3a** (0.2 mmol, 1 equiv), $AgNO_3$ (6.8mg, 20 mol %), Selectflour (140 mg, 2 equiv), aceton (1 mL) and H_2O (1 mL) stirred for 1h at 55 °C. The reaction was concentrated in vacuo. Products were purified by flash chromatography on silica gel with PE / EA=10 / 1 as the eluent.

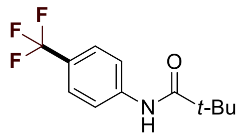

S1

White soild. Isolated yield: 40.2 mg, 82%.

^{1}H NMR (400 MHz, $CDCl_3$) δ 7.65 (d, J = 8.5 Hz, 2H), 7.54 (d, J = 8.5 Hz, 3H), 1.32 (s, 9H).

^{13}C NMR (101 MHz, $CDCl_3$) δ 177.11, 141.23, 126.27 (t, J_{C-F} = 6.0 Hz), 119.73, 39.91, 27.62.

^{19}F NMR (376 MHz, $CDCl_3$) δ -62.46.

Compound S2 was synthesized according to a reported procedures[3]**.**

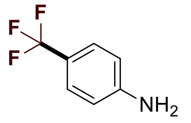

S2

White soild. Isolated yield: 31.9 mg, 99%.

^{1}H NMR (400 MHz, $CDCl_3$) δ 7.43 (d, J = 8.3 Hz, 2H), 6.68 (d, J = 8.4 Hz, 2H), 3.95 (s, 2H).

^{13}C NMR (101 MHz, $CDCl_3$) δ 149.63, 126.72 (q, J_{C-F} = 3.8 Hz), 114.24.

^{19}F NMR (376 MHz, $CDCl_3$) δ -60.98 (d, J = 5.0 Hz).

Compound Leflunomide was synthesized according to a reported procedures[4]**.**

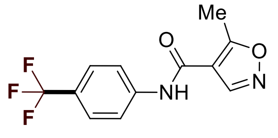

Leflunomide

White soild. Isolated yield: 49.1 mg, 91%.

^{1}H NMR (400 MHz, DMSO) δ 10.33 (s, 1H), 9.09 (s, 1H), 7.94 (t, J = 11.2 Hz, 2H), 7.70 (d, J = 8.6 Hz, 2H), 2.81 – 2.49 (m, 3H).

^{13}C NMR (101 MHz, DMSO) δ 173.41, 159.61, 149.01, 142.29, 125.97 (q, J_{C-F} = 3.6 Hz), 120.00, 111.78, 12.17.

^{19}F NMR (376 MHz, DMSO) δ -60.55.

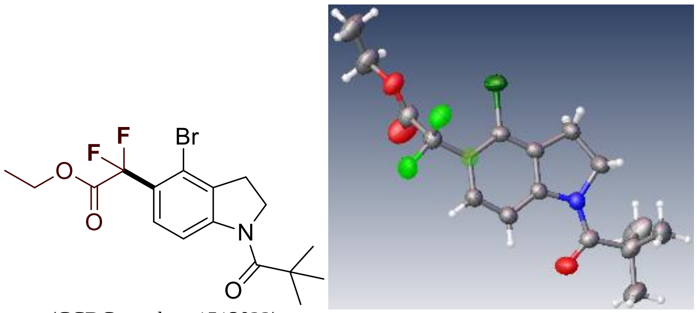

(CCDC number: 1513022)

Supplementary Figure 14. X–Ray Data for Complex 5c

Intensity data were collected with a Rigaku Mercury CCD area detector in ω scan mode using Mo Kαradiation (λ = 0.71070 Å). The diffracted intensities were corrected for Lorentz polarization effects and empirical absorption corrections. Details of the intensity data collection and crystal data are given in Table 2. The structures were solved by direct methods and refined by fullmatrix least–squares procedures based on |F|2 . All the non–hydrogen atoms were refined anisotropically. All the H atoms were held stationaryand included in the structure factor calculation in the final stage of full–matrix least–squares refinement. The structures were solved and refinedusing OLEX-2 programs.

Supplementary Table 3

Empirical formula	C17 H20 F2 N O3
Formula weight	359.79
Temperature	296(2) K
Wavelength	0.71073 A
Crystal system, space group	monoclinic
Unit cell dimensions	a = 8.1010(6) A alpha = 90 deg<br>b = 9.6695(8) A beta = 90.768(2) deg<br>c = 22.3350(19) A gamma = 90 deg
Volume	1749.4(2) A^3
Z, Calculated density	4 Mg/m^3
Absorption coefficient	0.253 mm^-1
F(000)	752
Crystal size	0.50 x 0.40 x 0.30 mm
Thata range for data collection	2.30 to 26.37 deg
Limiting indices	–10<=h<=10, –12<=k<=12, –28<=l<=28
Reflections collected / unique	4004 / 2974
Completeness to theta = 25.00	99.8 %
Absorption correction	Semi–empirical from equivalents
Max. and min. transmission	0.927 and 0.886
Refinement method	Full–matrix least–squares on F^2
Data / restraints / parameters	4004 / 0 / 217
Goodness–of–fit on F^2	0.742
Final R indices [I>2sigma(I)]	R1 = 0.0744, wR2 = 0.1965
R indices (all data)	R2 = 0.0493, wR2 = 0.1616

Largest diff. peak and hole	0.458 and -0.512 e.A^-3

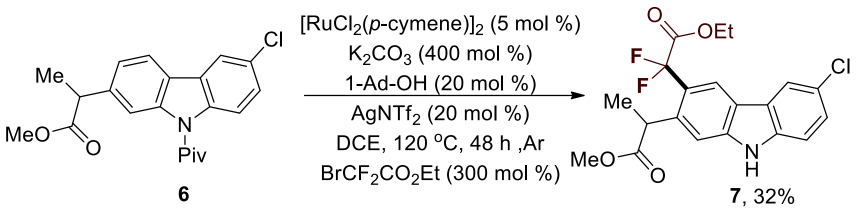

Supplementary Figure 15. Procedures for preparation of 7

General procedure for synthesis of 7: A mixture of **6** (0.2 mmol, 1.0 equiv), $BrCF_2CO_2Et$ (80 µL, 121.2 mg, 3 equiv), $[RuCl_2(p\text{-cymene})]_2$ (6 mg, 5 mol %), K_2CO_3 (108.8 mg, 400 mol %), 1-Ad-OH (7.2 mg, 20 mol %), $AgNTf_2$ (14.4 mg, 20 mol %) and DCE (0.5 mL) in a 15 mL glass vial sealed under argon atmosphere was heated at 120 °C for 48 hours. The reaction mixture was cooled to room temperature and concentrated *in vacuo*. The resulting residue was purified by column chromatography (PE / EA = 10 : 1) on silica gel to give the product **7**.

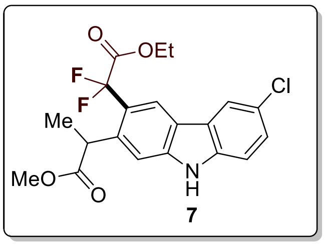

White solid. Isolated yield: 26.1 mg, 32%.
^{1}H NMR (400 MHz, $CDCl_3$) δ 8.84 (brs, 1H), 8.11 (s, 1H), 7.96 (d, J = 8.1 Hz, 1H), 7.59 (d, J = 1.6 Hz, 1H), 7.46 (s, 1H), 7.22 (dd, J = 8.1, 1.1 Hz, 1H), 4.31 (dd, J = 14.2, 7.1 Hz, 2H), 3.90 (dd, J = 14.3, 7.1 Hz, 1H), 3.68 (s, 3H), 1.59 (d, J = 7.1 Hz, 3H), 1.30 (t, J = 7.2 Hz, 3H).
^{13}C NMR (101 MHz, $CDCl_3$) δ 175.08, 154.73, 140.59, 140.45, 134.74 (t, J_{C-F} = 3.7 Hz), 126.85, 126.07, 124.78, 123.05, 122.76 (t, J_{C-F} = 7.5 Hz), 120.84, 120.39, 119.98, 110.20, 63.99, 52.32, 45.98, 19.08, 14.02.
^{19}F NMR (376 MHz, $CDCl_3$) δ -104.90.
HRMS Calcd for $C_{20}H_{19}ClF_2NO_4$ [M+H$^+$]: 410.0971; Found: 410.0980.

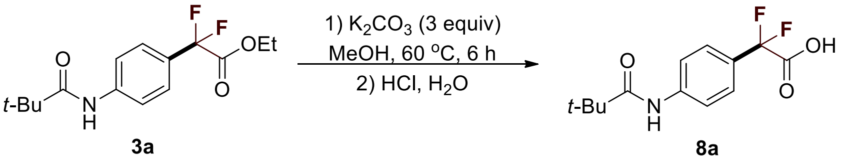

Supplementary Figure 16. Procedures for preparation of 8a[5].

General procedure for synthesis of 8a: In a 15 mL glass vial, a mixture of **3a** (0.2 mmol, 1 equiv), MeOH (30 mL) and 1 M K_2CO_3 aq. (30 mL) and stirred for 6h at 60 °C. The reaction was then poured into 1 M HCl aq. to acidify to pH 1, and the aqueous phase was extracted with EtOAc (100 mL),

washed with water (100 mL), dried over Na_2SO_4 and concentrated in vacuo. Products were purified by flash chromatography on silica gel with PE / EA = 2 / 1 as the eluent.

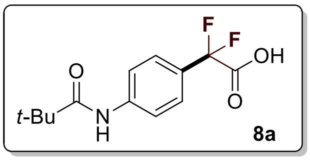

White solid. Isolated yield: 54.0 mg, 99%.

^{1}H NMR (400 MHz, DMSO) δ 9.49 (brs, 1H), 7.71 (d, J = 8.1 Hz, 2H), 7.46 (d, J = 8.3 Hz, 2H), 1.23 (s, 9H).

^{13}C NMR (101 MHz, DMSO) δ 177.13, 166.12 (t, $J_{C\text{-}F}$ = 29.8 Hz), 140.86, 131.77 (t, $J_{C\text{-}F}$ = 26.2 Hz), 126.04 (t, $J_{C\text{-}F}$ = 5.7 Hz), 119.91, 115.83 (t, $J_{C\text{-}F}$ = 251.2 Hz), 39.67, 27.63.

^{19}F NMR (376 MHz, DMSO) δ -97.58.

HRMS Calcd for $C_{13}H_{16}F_2NO_3$ [M+H$^+$]: 272.1098; Found: 272.1089.

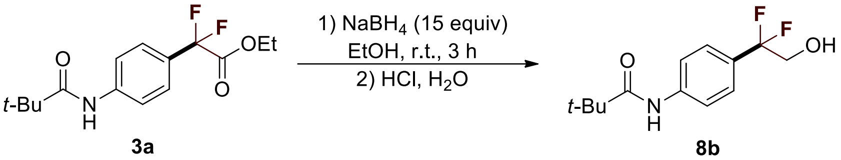

Supplementary Figure 17. Procedures for preparation of 8b. [6]

General procedure for synthesis of 8b: In a 15 mL glass vial, a mixture of **3a** (0.2 mmol, 1 equiv), $NaBH_4$ (15 equiv), EtOH (2 mL) and stirred for 3h at room temperature. The reaction was then poured into 1 M HCl aq. to acidify to pH 1, and the aqueous phase was extracted with EtOAc (100 mL), washed with water (100 mL), dried over Na_2SO_4 and concentrated in vacuo. Products were purified by flash chromatography on silica gel with PE / EA = 2 / 1 as the eluent.

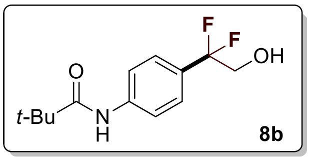

White solid. Isolated yield: 47.3 mg, 92%.

^{1}H NMR (400 MHz, $CDCl_3$) δ 7.62 (d, J = 7.8 Hz, 2H), 7.47 (d, J = 7.9 Hz, 2H), 7.40 (brs, 1H), 4.06 – 3.84 (m, 2H), 2.00 (s, 1H), 1.32 (s, 9H).

^{13}C NMR (101 MHz, $CDCl_3$) δ 176.94, 139.97, 130.07 (t, $J_{C\text{-}F}$ = 26.0 Hz), 126.53 (t, $J_{C\text{-}F}$ = 6.2 Hz), 120.64 (t, $J_{C\text{-}F}$ = 228.5 Hz), 119.80, 66.16 (t, $J_{C\text{-}F}$ = 32.9 Hz), 39.88, 27.73.

^{19}F NMR (376 MHz, $CDCl_3$) δ -106.48.

HRMS Calcd for $C_{13}H_{18}F_2NO_2$ [M+H$^+$]: 258.1306; Found: 258.1301.

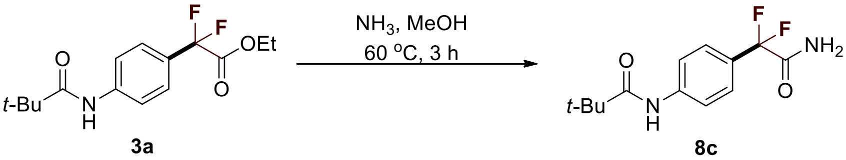

Supplementary Figure 18. Procedures for preparation of 8c. [7]

General procedure for synthesis of 8c: In a 15 mL glass vial, a mixture of **3a** (0.2 mmol, 1 equiv) and NH_3 (2M in MeOH, 2 mL) stirred for 3h at 60 °C. The reaction was then extracted with EtOAc (100mL), washed with water (100 mL), dried over Na_2SO_4 and concentrated in vacuo. Products were purified by flash chromatography on silica gel with PE / EA = 2 / 1 as the eluent.

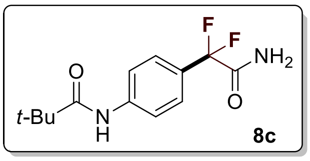

White solid. Isolated yield: 46.0 mg, 85%.

^{1}H NMR (400 MHz, DMSO) δ 9.22 (brs, 1H), 8.09 (brs, 1H), 7.73 (brs, 1H), 7.54 (d, J = 8.5 Hz, 2H), 7.27 (d, J = 8.6 Hz, 2H), 1.00 (s, 9H).

^{13}C NMR (101 MHz, DMSO) δ 177.45, 165.98 (t, $J_{C\text{-}F}$ = 31.6 Hz), 141.98, 128.18 (t, $J_{C\text{-}F}$ = 26.1 Hz), 126.05 (t, $J_{C\text{-}F}$ = 5.9 Hz), 120.32, 115.17 (t, $J_{C\text{-}F}$ = 251.7 Hz), 39.73, 27.51.

^{19}F NMR (376 MHz, DMSO) δ -101.00.

HRMS Calcd for $C_{13}H_{17}F_2N_2O_2$ [M+H$^+$]: 271.1258; Found: 271.1264.

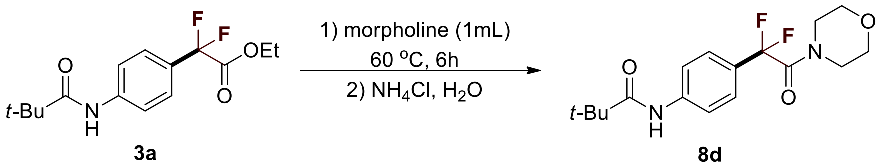

Supplementary Figure 19. Procedures for preparation of 8d.

General procedure for synthesis of 8d: In a 15 mL glass vial, a mixture of **3a** (0.2 mmol, 1 equiv) and morpholine (1 mL) stirred for 6h at 60 °C. The reaction was then poured into saturated NH_4Cl solution, and the aqueous phase was extracted with EtOAc (100 mL), washed with water (100 mL), dried over Na_2SO_4 and concentrated in vacuo. Products were purified by flash chromatography on silica gel with PE / EA = 2 / 1 as the eluent.

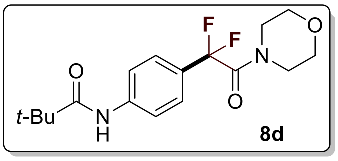

White solid. Isolated yield: 53.0 mg, 78%.

^{1}H NMR (400 MHz, $CDCl_3$) δ 7.65 (d, J = 8.5 Hz, 2H), 7.48 (d, J = 8.4 Hz, 3H), 3.69 (s, 4H), 3.46 (s, 4H), 1.31 (s, 9H).

^{13}C NMR (101 MHz, $CDCl_3$) δ 177.00, 162.27 (t, $J_{C\text{-}F}$ = 30.4 Hz), 140.66, 128.83 (t, $J_{C\text{-}F}$ = 25.3 Hz), 126.20 (t, $J_{C\text{-}F}$ = 5.6 Hz), 119.90, 115.50 (t, $J_{C\text{-}F}$ = 250.1 Hz), 66.79, 66.49, 46.79, 43.59, 39.91, 27.67.

^{19}F NMR (376 MHz, $CDCl_3$) δ -93.77.

HRMS Calcd for $C_{17}H_{23}F_2N_2O_3$ [M+H$^+$]: 341.1677; Found: 341.1671.

Preliminary mechanism studies

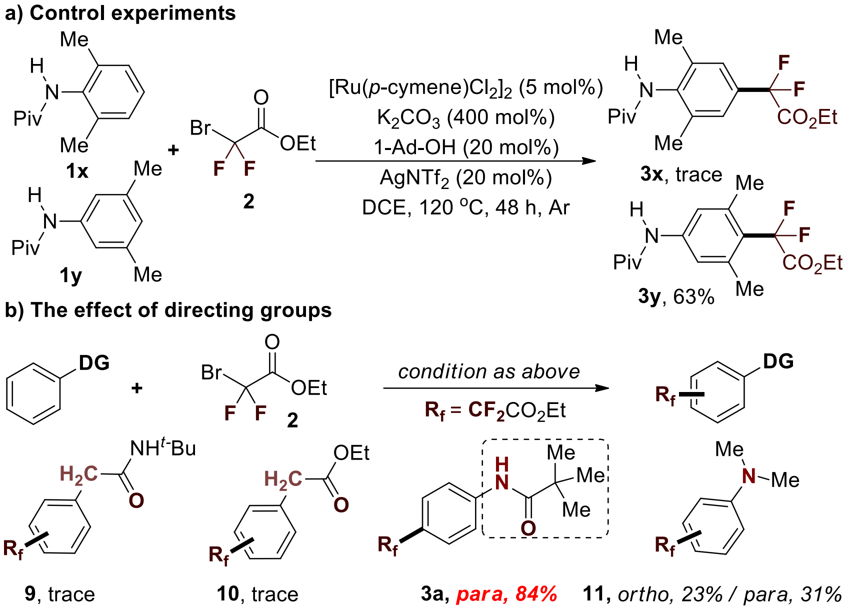

Supplementary Figure 20. Control experiments I

Procedures for preparation of 3z, 3aa, 9, and 10 were followed the above condition.

Procedures for preparation of 11: A mixture of N,N-dimethylaniline (0.2 mmol, 1.0 equiv), $BrCF_2CO_2Et$ (80 μL, 121.2 mg, 3 equiv), $[RuCl_2(p\text{-cymene})]_2$ (6 mg, 5 mol %), K_2CO_3 (108.8 mg, 400 mol %), 1-Ad-OH (7.2 mg, 20 mol %), $AgNTf_2$ (14.4 mg, 20 mol %) and DCE (0.5 mL) in a 15 mL glass vial sealed under argon atmosphere heated at 120 °C for 48 hours. The reaction mixture cooled to room temperature and concentrated *in vacuo*. The resulting residue was purified by column chromatography (PE / EA = 10 : 1) on silica gel to give the difluoroacetylated products. The ortho-difluoroacetylated product **11-ortho** was obtained in 11.2 mg (23% yield) as brown oil. The para-difluoroacetylated product **11-para** was obtained in 15.1 mg (31% yield) as brown oil.

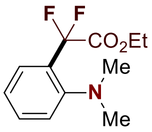

11 *ortho*

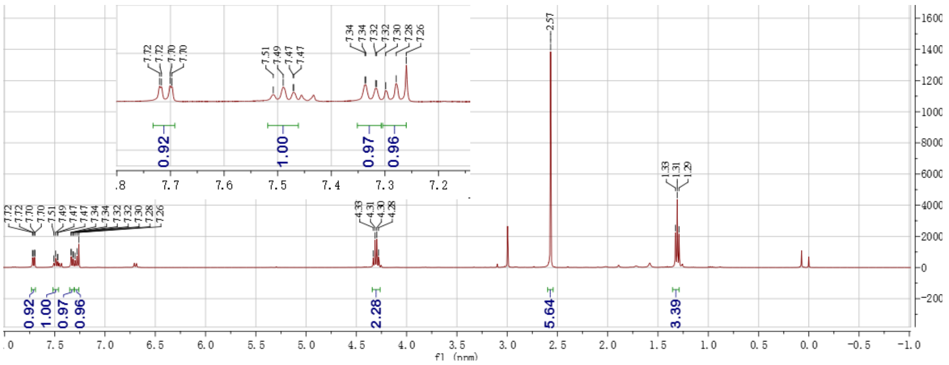

Supplementary Figure 21. ^{1}H NMR analysis of 11 *ortho*

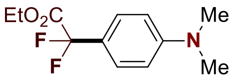

11 *para*

Brown oil. Isolated yield: 15.1 mg, 31%.

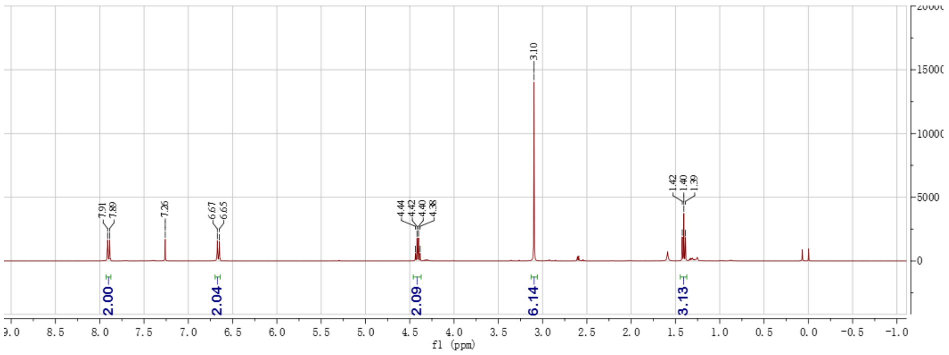

Supplementary Figure 22. ^{1}H NMR analysis of 11 *para*

When we treat this reaction without $[RuCl_2(p\text{-cymene})]_2$. It alomost no reaction.

To further get insight of this reaction, we directly subjected the N,N-dimethylaniline to the condition in which the $\cdot CF_2CO_2Et$ radical could be generated (*J. Org. Chem.* **2015**, *80*, 12258.). 31% ortho product and 42% para product were obtained.

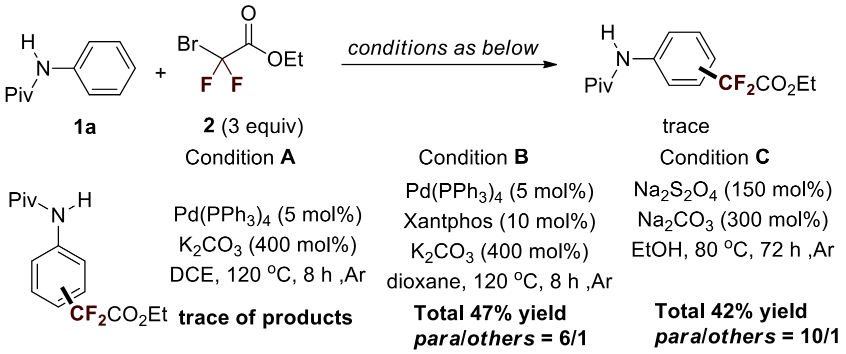

Supplementary Figure 23. Control experiments II.

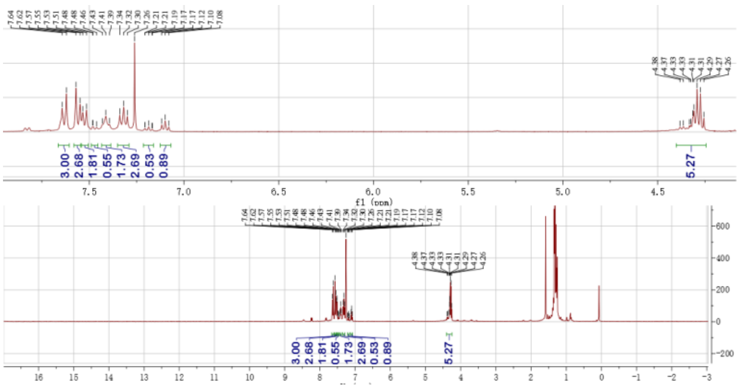

Supplementary Figure 24. ^{1}H NMR analysis of 1a performed in condition B

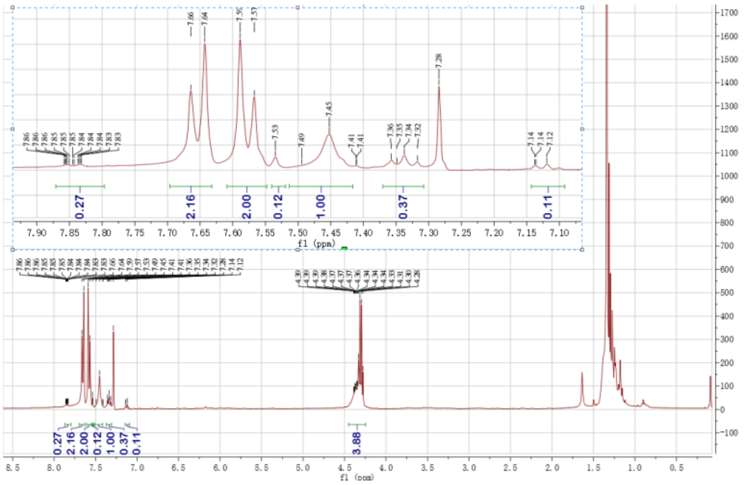

Supplementary Figure 25. ^{1}**H NMR analysis of 1a performed in condition C**

Control experiments

To further get insight of the reaction pathway, several other control experiments were performed.The •CF_2CO_2Et radical can be generated from 2-bromo-2,2-difluoroacetates, which were reported by the groups of Wang,[8] Ackermann,[9] Zhang[10] and Kondratov[11], respectively. Thus we directly treated the substrate **1a** with **2** in the presence of the radical initiator. Interestingly, we found that the difluorom-ehyl radical could be trapped by the substrate 1a. However, only a mixture of ortho, meta, and para-difluoromethylated products were obtained. These results clearly indicated that the arylruthenium intermediate (Scheme 5, c, IV) is the dominant effect in realizing the *para*-selectivity.

General procedures of the condition A[8]**:** A mixture of **1a** (0.2 mmol, 1.0 equiv), $BrCF_2CO_2Et$ (80 µL, 121.2 mg, 3 equiv), $Pd(PPh_3)_4$ (11.5 mg, 10 mol %), K_2CO_3 (108.8 mg, 400 mol %), and DCE (0.5 mL) in a 15 mL glass vial sealed was heated at 120 ºC for 8 hours. The reaction mixture cooled to room temperature and concentrated *in vacuo*. The resulting residue was purified by column chromatography (PE / EA = 10 : 1) on silica gel to give the product.

General procedures of the condition B[8,9,10]**:** A mixture of **1a** (0.2 mmol, 1.0 equiv), $BrCF_2CO_2Et$ (80 µL, 121.2 mg, 3 equiv), $Pd(PPh_3)_4$ (11.5 mg, 10 mol %), Xantphos (12 mg, 10 mol %), K_2CO_3 (57 mg, 2 equiv) and dioxane (0.5 mL) in a 15 mL glass vial sealed was heated at 120 ºC for 8 hours. The reaction mixture cooled to room temperature and concentrated *in vacuo*. The resulting residue was purified by column chromatography (PE / EA = 10 : 1) on silica gel to give the product. (28 mg, 47%, a mixture of para and others product.)

General procedures of the condition C[11]**:** A mixture of 1a (0.2 mmol, 1.0 equiv), $BrCF_2CO_2Et$ (80 µL, 121.2 mg, 3 equiv), sodium bicarbonate (63 mg g, 0.6 mmol), sodium dithionite (53 mg, 0.3 mmol), and ethanol (0.5 mL) was put into a 15 mL glass vial sealed and the mixture was vigorously stirred at 80 °C for 72 h. Then the reaction was cooled to room temperature, carefully opened (liberation of gases CO_2/SO_2), and treated with water (10 mL). The mixture extracted with MTBE (3 × 15 mL). The combined organic layers washed with water (3 × 10 mL), brine (15 mL), and dried with Na_2SO_4. The solvent removed under reduced pressure, and the residue purified by distillation under reduced pressure

using a Claisen condenser setup or by column chromatography giving product. (25 mg, 42%, a mixture of para and meta product.)

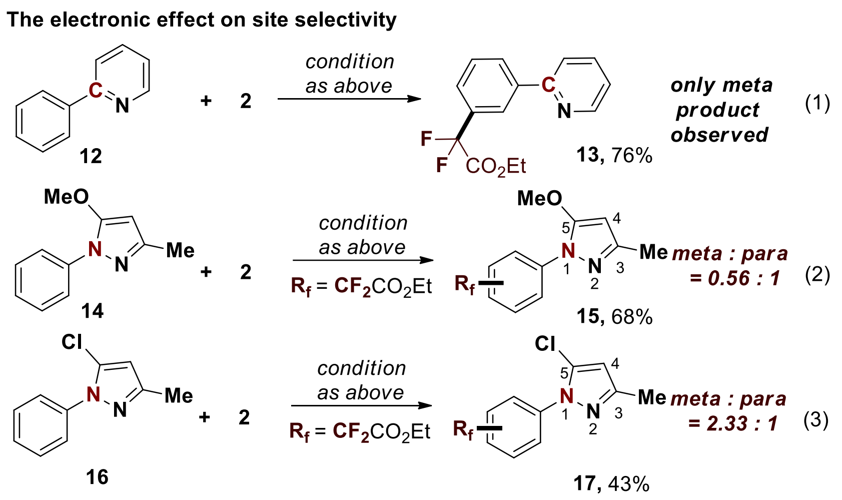

Supplementary Figure 26. Preliminary studies on site selectivity

Compound 14 was synthesized according to a reported procedures[12].

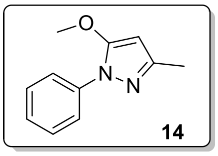

^{1}H NMR (400 MHz, CDCl3) δ 7.62 (d, *J* = 7.7 Hz, 2H), 7.32 (t, *J* = 7.1 Hz, 2H), 7.18 – 7.10 (m, 1H), 5.42 (s, 1H), 3.81 (s, 3H), 2.21 (s, 3H).

^{13}C NMR (101 MHz, CDCl3) δ 155.82, 148.72, 138.73, 128.78, 125.86, 121.82, 85.81, 58.74, 14.59.

Compound 3-methyl-5-trifluoromethylphenylpyrazole was synthesized according to a reported procedures[13].

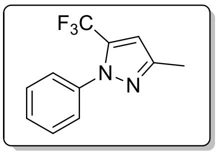

^{1}H NMR (400 MHz, $CDCl_3$) δ 7.56 – 7.36 (m, 5H), 6.46 (s, 1H), 2.34 (s, 3H).

^{13}C NMR (101 MHz, $CDCl_3$) δ 142.80 (q, *J* = 38.1 Hz), 140.81, 139.00, 129.38, 128.84, 125.41, 121.55 (q, *J* = 268.7 Hz), 104.93, 12.38.

Procedures for preparation of 13, 15 and 17 were followed Supplementary Figure 1 condition.

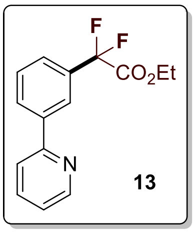

Yellow oil. Isolated yield: 42.1 mg 76%. This is a known compound.

^{1}H NMR (400 MHz, CDCl3) δ 8.71 (d, *J* = 3.4, 1.2 Hz, 1H), 8.23 (s, 1H), 8.16 (d, *J* = 7.8 Hz, 1H), 7.81 – 7.74 (m, 2H), 7.66 (d, *J* = 7.9 Hz, 1H), 7.57 (t, *J* = 7.8 Hz, 1H), 7.30 – 7.26 (m, 1H), 4.31 (q, *J* = 7.1 Hz, 2H), 1.31 (t, *J* = 7.1 Hz, 3H).

^{13}C NMR (101 MHz, CDCl3) δ 164.30 (t, $J_{C\text{-}F}$ = 35.2 Hz), 156.29, 149.92, 140.08, 137.13, 133.55 (t, $J_{C\text{-}F}$ = 25.5 Hz), 129.63, 129.31, 126.07 (t, $J_{C\text{-}F}$ = 6.1 Hz), 124.10 (t, $J_{C\text{-}F}$ = 6.3 Hz), 122.84, 120.85, 113.50 (t, $J_{C\text{-}F}$ = 252.4 Hz), 63.35, 14.03.

^{19}F NMR (376 MHz, CDCl3) δ -103.74.

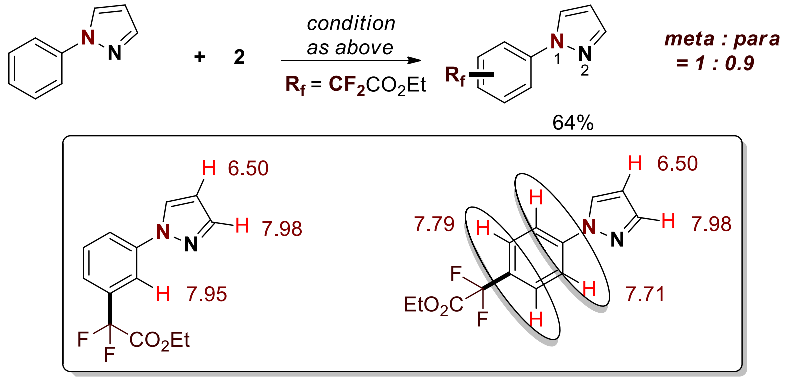

Supplementary Figure 27.

Note: These two compounds could not be separated.

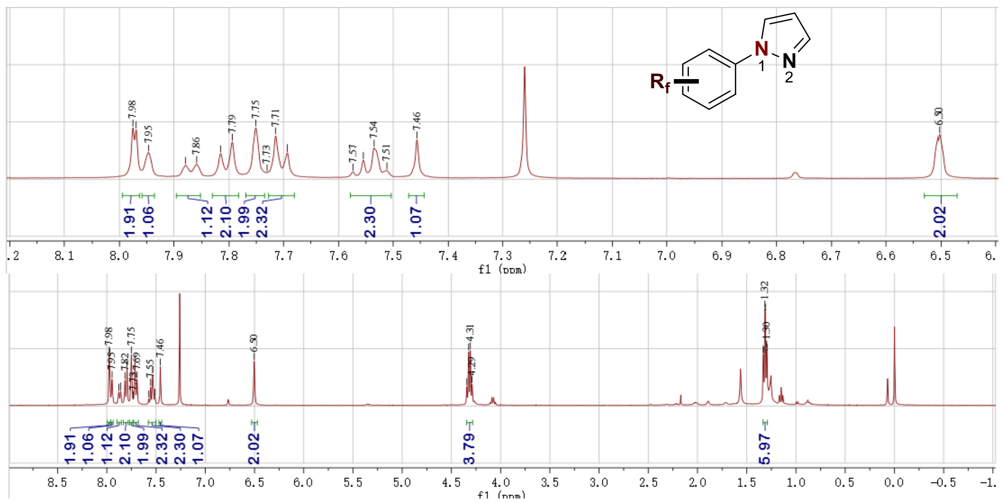

Supplementary Figure 28. ^{1}H NMR analysis of 1-phenyl-1H-pyrazole

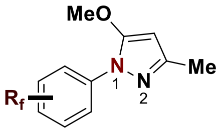

15

Note: These two compounds could not be separated.

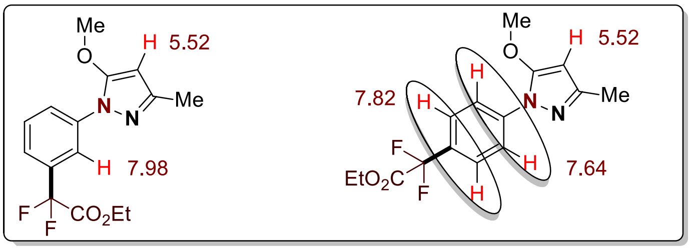

Supplementary Figure 29.

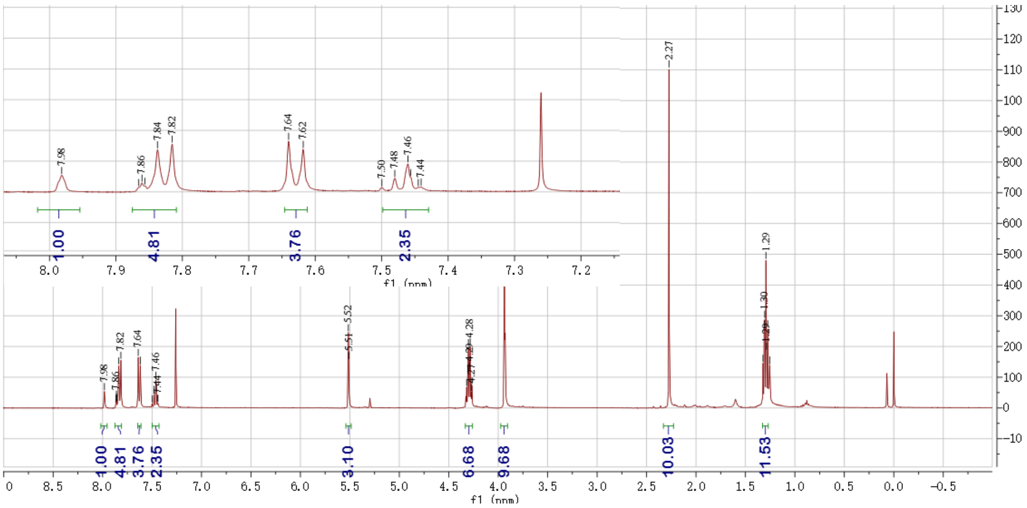

Supplementary Figure 30. ^{1}**H NMR analysis of 15**

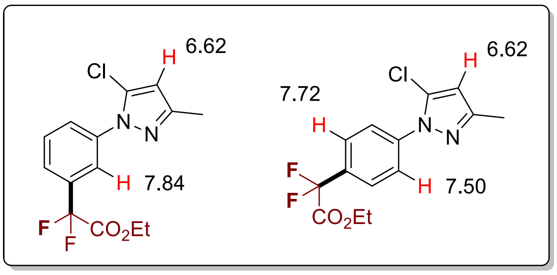

Supplementary Figure 31.

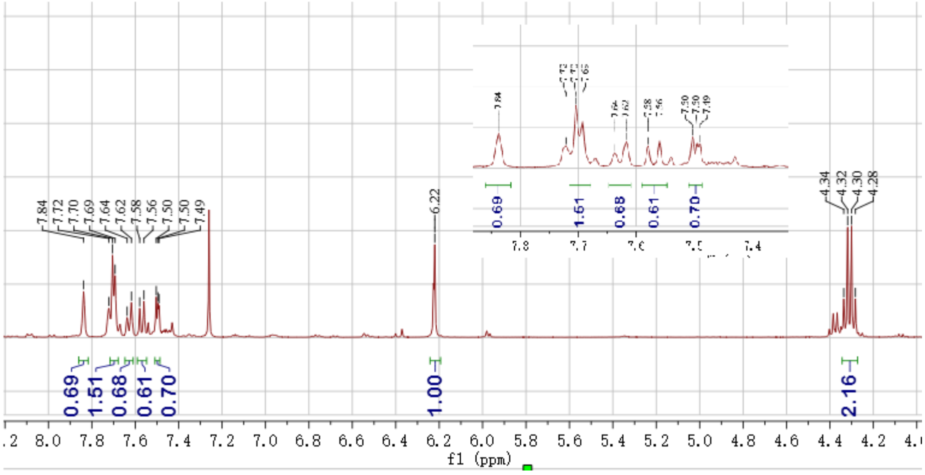

Supplementary Figure 32. ^{1}**H NMR analysis of 17**

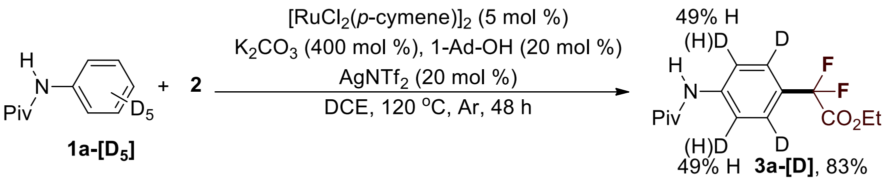

Supplementary Figure 33. Isotopic and free radical studies

General procedure for synthesis of 3a-[D]: A mixture of **1a-[D**$_5$**]** (0.2 mmol, 1.0 equiv), $BrCF_2CO_2Et$ (80 µL, 121.2 mg, 3 equiv), $[RuCl_2(p\text{-cymene})]_2$ (6 mg, 5 mol %), K_2CO_3 (108.8 mg, 400 mol %), 1-Ad-OH (7.2 mg, 20 mol %), $AgNTf_2$ (14.4 mg, 20 mol %) and DCE (0.5 mL) in a 15 mL glass vial sealed under argon atmosphere heated at 120 °C for 48 hours. The reaction mixture cooled to room temperature and concentrated *in vacuo*. The resulting residue was purified by column chromatography (PE / EA = 10 : 1) on silica gel to give the product **3a-[D]**.

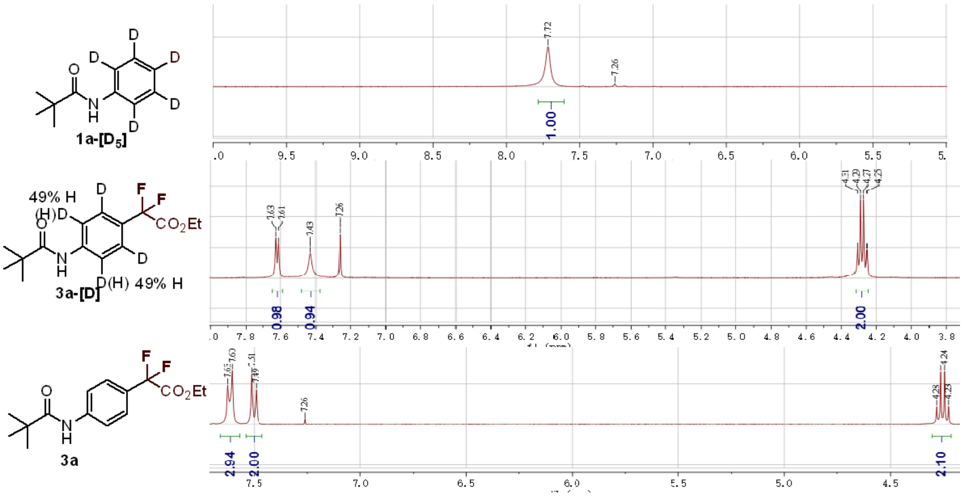

Supplementary Figure 34. ^{1}**H NMR analysis of Isotopic Labeling Studies**

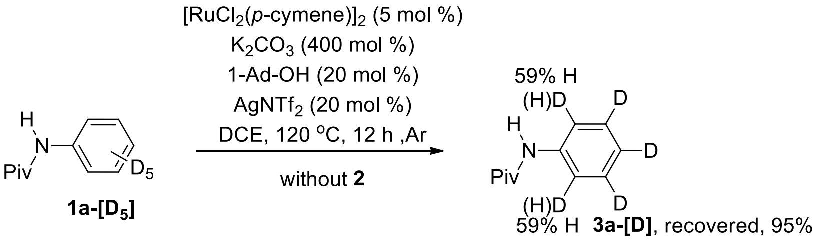

Supplementary Figure 35.

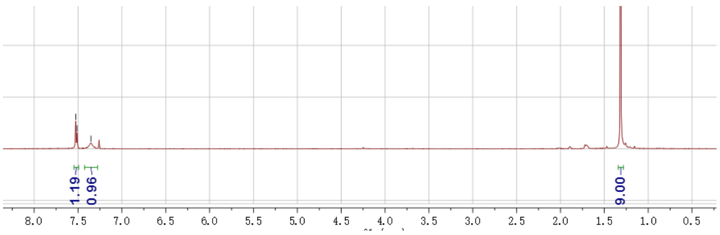

Supplementary Figure 36. ^{1}**H NMR analysis of 3a-[D]**

General procedure: A mixture of **1a-[D5]** (0.2 mmol, 1.0 equiv), $[RuCl_2(p\text{-cymene})]_2$ (6 mg, 5 mol %), K_2CO_3 (108.8 mg, 400 mol %), 1-Ad-OH (7.2 mg, 20 mol %), $AgNTf_2$ (14.4 mg, 20 mol %) and DCE (0.5 mL) in a 15 mL glass vial sealed under argon atmosphere was heated at 120 °C for 48 hours. The reaction mixture cooled to room temperature and concentrated *in vacuo*. The resulting residue was purified by column chromatography (PE / EA = 10 : 1) on silica gel to recovered **1a-[D]**. Analysis as followed.

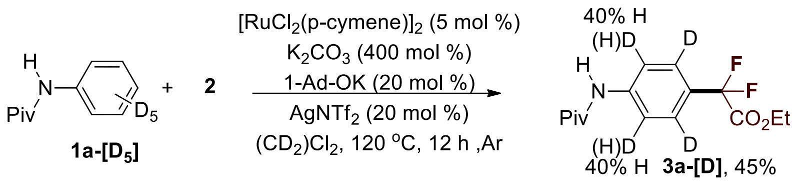

Supplementary Figure 37.

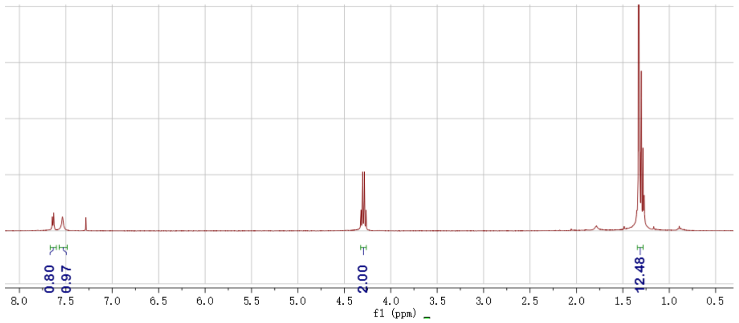

Supplementary Figure 38. ^{1}H NMR analysis of 3a-[D]

The reaction performed with 1-Ad-OK and deuterated DCE instead of 1-Ad-OH and DCE under standard condition (generally regents and tubes, not completely dried). The result clearly show that a relevant D/H scrambling was still present (H/D = 2/3), which is analogues to the result when the 1-Ad-OH and DCE were employed in the reaction.

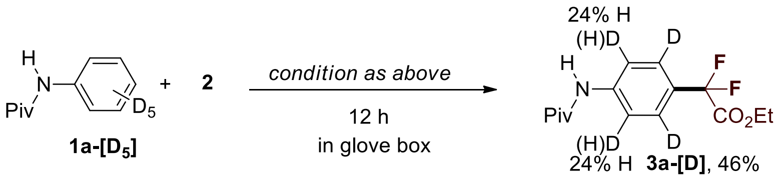

Supplementary Figure 39.

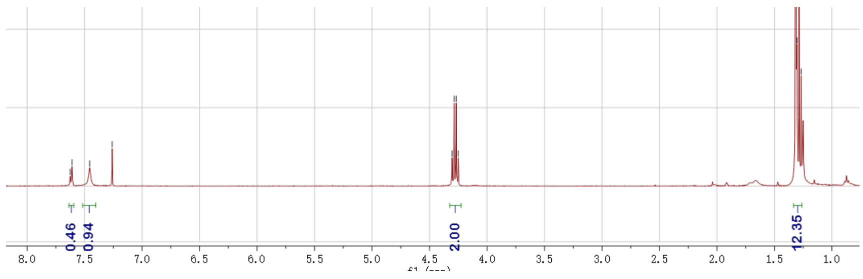

Supplementary Figure 40. ^{1}H NMR analysis of 3a-[D]

When we performed the reaction in the glove box, the relevant D/H scrambled product still obtained

(1/4). Given these result we can speculate that the proton is came from H_2O which is hard to completely avoid in the reaction.

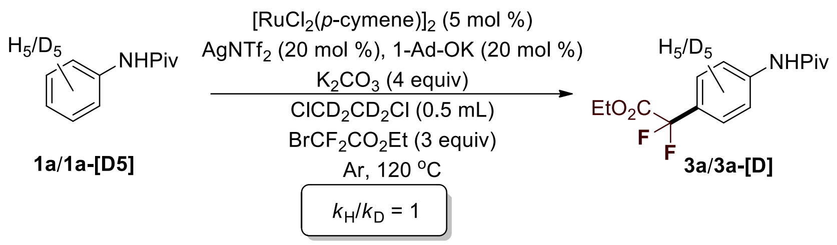

Supplementary Figure 41. Kinetic Isotope Effect Studies

General procedure: A mixture of **1a-[D5] or 1a** (0.2 mmol, 1.0 equiv), $BrCF_2CO_2Et$ (80 μL, 121.2 mg, 3 equiv), $[RuCl_2(p\text{-cymene})]_2$ (6 mg, 5 mol %), K_2CO_3 (108.8 mg, 400 mol %), 1-Ad-OK (10 mg, 20 mol %), $AgNTf_2$ (14.4 mg, 20 mol %) and $(CD_2Cl)_2$ (0.5 mL) in a 15 mL glass vial sealed under argon atmosphere heated at 120 °C for 30-90 mins. The reaction mixture cooled to room temperature and concentrated *in vacuo*. The resulting residue was purified by column chromatography (PE / EA = 100 : 10) on silica gel to give the product **3a-[D] or 3a**. GC analysis using *n*-Tridecane as an internal standard to provide the following conversions.

Mins	30	45	60	75	90
3a	15%	18%	20%	23%	28%
3a-[D]	13%	15%	18%	22%	24%

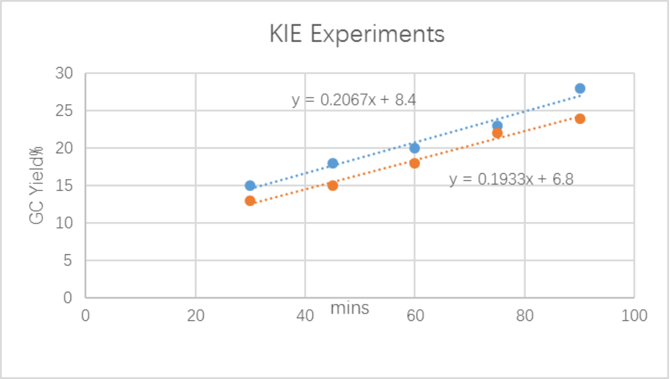

$$KIE = k_H / k_D = 0.2067 / 0.1933 = 1.07$$

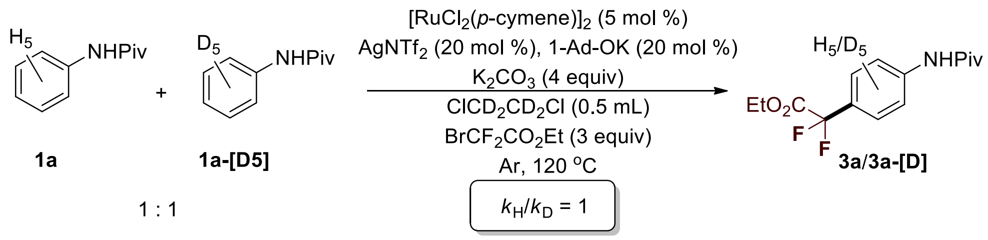

Supplementary Figure 42.

General procedure: A mixture of **1a-[D$_5$] and 1a** (0.1 + 0.1 mmol, 1.0 equiv), $BrCF_2CO_2Et$ (80 µL, 121.2 mg, 3 equiv), $[RuCl_2(p\text{-cymene})]_2$ (6 mg, 5 mol %), K_2CO_3 (108.8 mg, 400 mol %), 1-Ad-OK (10 mg, 20 mol %), $AgNTf_2$ (14.4 mg, 20 mol %) and $(CD_2Cl)_2$ (0.5 mL) in a 15 mL glass vial sealed under argon atmosphere heated at 120 °C for 12 hours. The reaction mixture cooled to room temperature and concentrated *in vacuo*. The resulting residue was purified by column chromatography (PE / EA = 100 : 10) on silica gel to give the product. NMR analysis as followed..

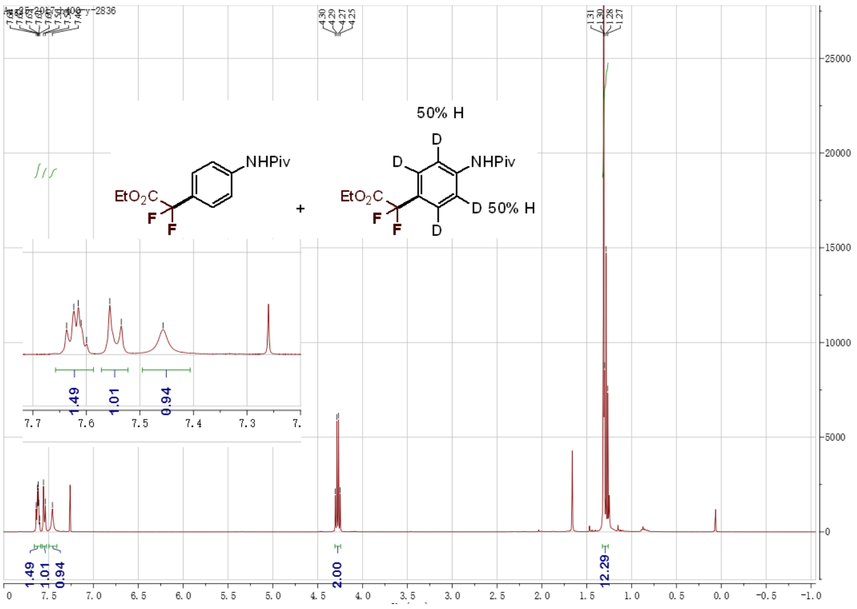

Supplementary Figure 43.

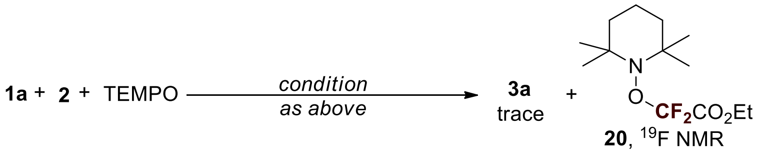

Supplementary Figure 44. Radical mechanism experiment

General procedure for synthesis of 20: A mixture of **1a** (0.2 mmol, 1.0 equiv), $BrCF_2CO_2Et$ (80 µL, 121.2 mg, 3 equiv), $[RuCl_2(p\text{-cymene})]_2$ (6 mg, 5 mol %), K_2CO_3 (108.8 mg, 400 mol %), 1-Ad-OH (7.2 mg, 20 mol %), $AgNTf_2$ (14.4 mg, 20 mol %) and DCE (0.5 mL) in a 15 mL glass vial sealed under argon atmosphere heated at 120 °C for 48 hours. The reaction mixture cooled to room temperature and concentrated *in vacuo*. The resulting residue was purified by column chromatography (PE : EA / 20 : 1) on silica gel to give the product **20**. The structure was determined according to the ^{19}F NMR analysis and HRMS analysis as following.

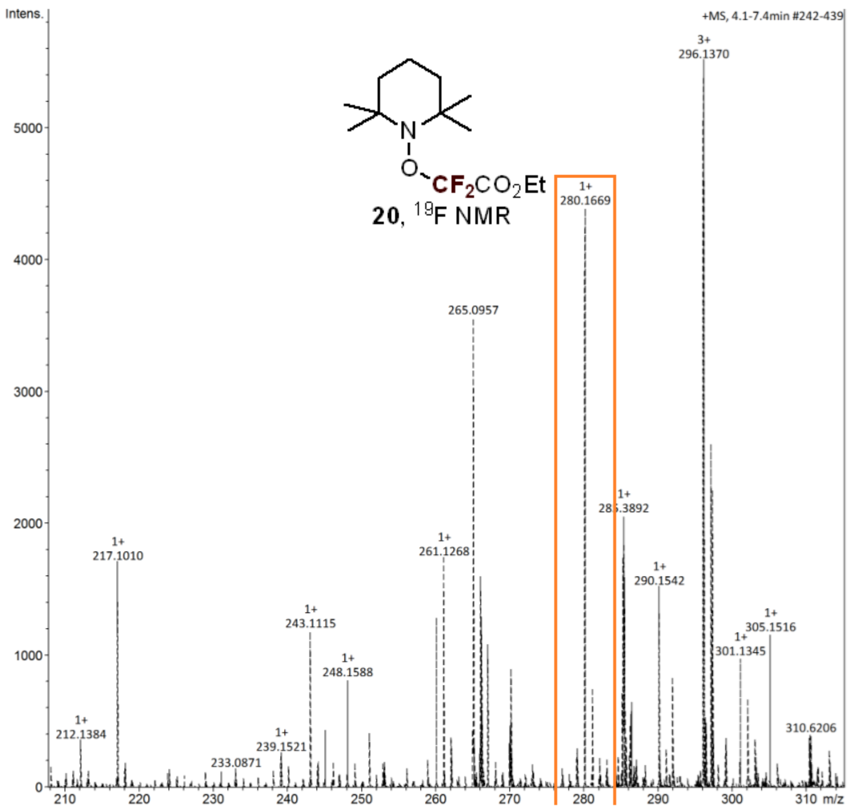

Supplementary Figure 45. HRMS analysis of 20

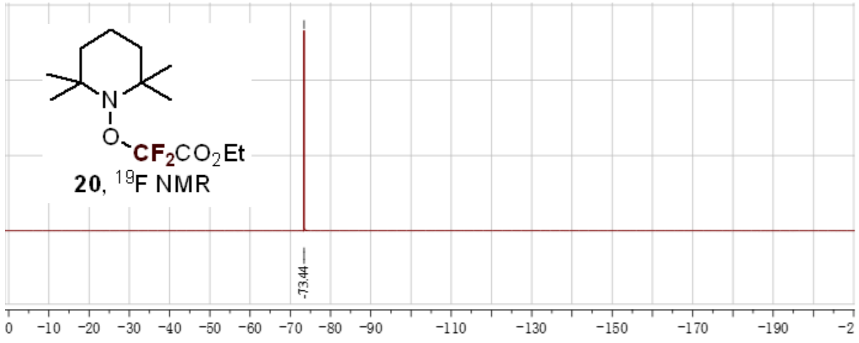

Supplementary Figure 46. ^{19}F NMR analysis of 20

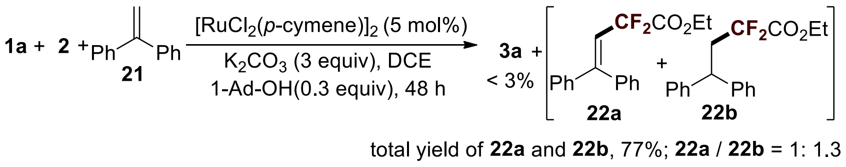

Supplementary Figure 47.

General procedure for synthesis of 22: A mixture of **1a** (0.2 mmol, 1.0 equiv), $BrCF_2CO_2Et$ (80μL, 121.2 mg, 3 equiv), $[RuCl_2(p\text{-cymene})]_2$ (6 mg, 5 mol %), K_2CO_3 (108.8 mg, 400 mol %), 1-Ad-OH (7.2 mg, 20 mol %), ethene-1,1-diyldibenzene (36.0 mg, 1.0 equiv) and DCE (0.5 mL) in a 15 mL glass vial sealed under argon atmosphere heated at 120 °C for 12 hours. The reaction mixture cooled to room temperature and concentrated *in vacuo*. The resulting residue was purified by column chromatography (PE : EA / 20 : 1) on silica gel to give the product **22a** and **22b** (colorless oil, 46.5 mg). The structure was determined according to the ^{1}H NMR analysis as following.

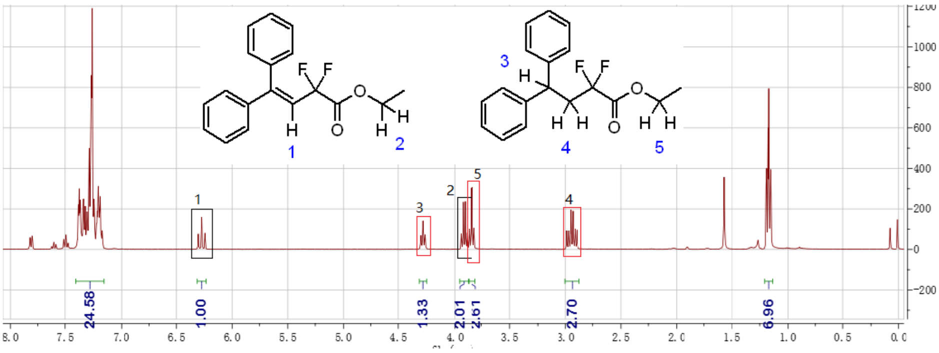

Supplementary Figure 48. ^{1}**H NMR analysis of 22**

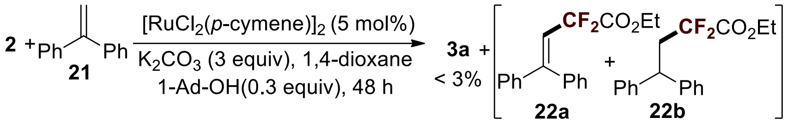

total yield of **22a** and **22b**, 40%

Supplementary Figure 49.

General procedure: A mixture of $BrCF_2CO_2Et$ (80μL, 121.2 mg, 3 equiv), $[RuCl_2(p\text{-cymene})]_2$ (6 mg, 5 mol %), K_2CO_3 (108.8 mg, 400 mol %), 1-Ad-OH (7.2 mg, 20 mol %), ethene-1,1-diyldibenzene (36.0 mg, 1.0 equiv) and 1,4-dioxane (1 mL) in a 15 mL glass vial sealed under argon atmosphere was heated at 120 °C for 12 hours. The reaction mixture cooled to room temperature and concentrated *in vacuo*. The resulting residue was purified by column chromatography (PE:EA / 20:1) on silica gel to give the product **22a** and **22b** (colorless oil, 27.2 mg).

Note: 22a and **22b** could not be separated.

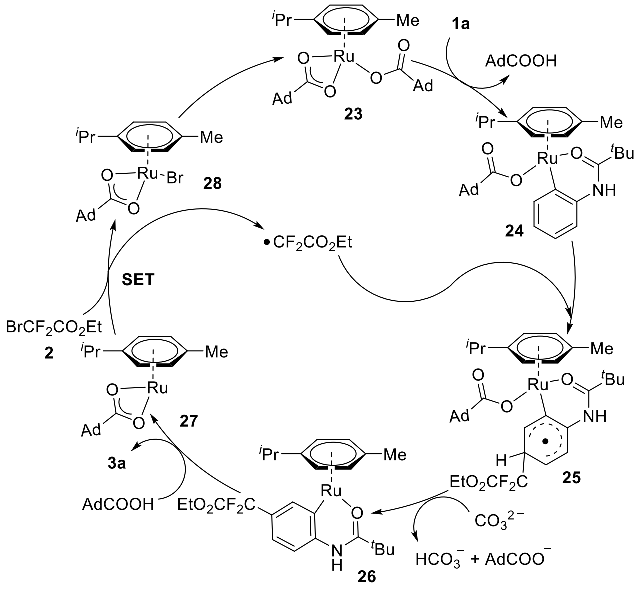

Figure 50. Proposed reaction mechanism

At the same time, we cannot completely rule out the pathway that this reaction is radical addition induced by a ruthenium catalyst.

On the basis of the foregoing results in combination with previously reported findings,[11–20] we propose a plausible reaction pathway (illustrated in Figure **8**). The catalytic cycle starts with Ru catalyst **23**, which can afford a six-membered aryl Ru species **24** through *ortho*-C–H cleavage along with the chelation of the amide group in substrate **1a**. Subsequently, the $\cdot CF_2CO_2Et$ radical can be trapped by radical addition to give complex **25**, in which deprotonation–aromatization assisted by K_2CO_3 generates aryl ruthenium(I) species **26**. Difluoromethylated product **3a** is then released with concomitant generation of intermediate **27** after the protonation. Meanwhile, the radical species $\cdot CF_2CO_2Et$ can be generated via single-electron-transfer-type transformation of reactant **2** with the aid of a Ru(I) complex **27**. Here, the active Ru(II) catalyst **23** is regenerated after ligand exchange to complete the reaction cycle.[23-25]

Supplementary Figures

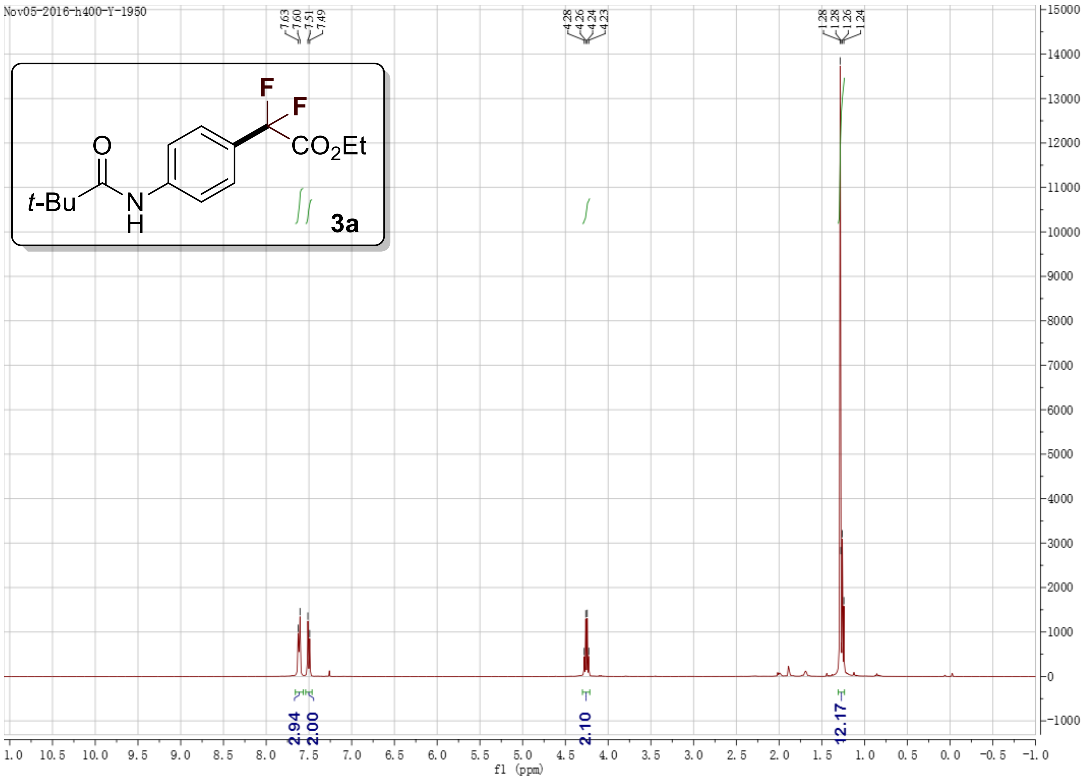

Supplementary Figure 51. ^{1}**H NMR spectra for 3a**

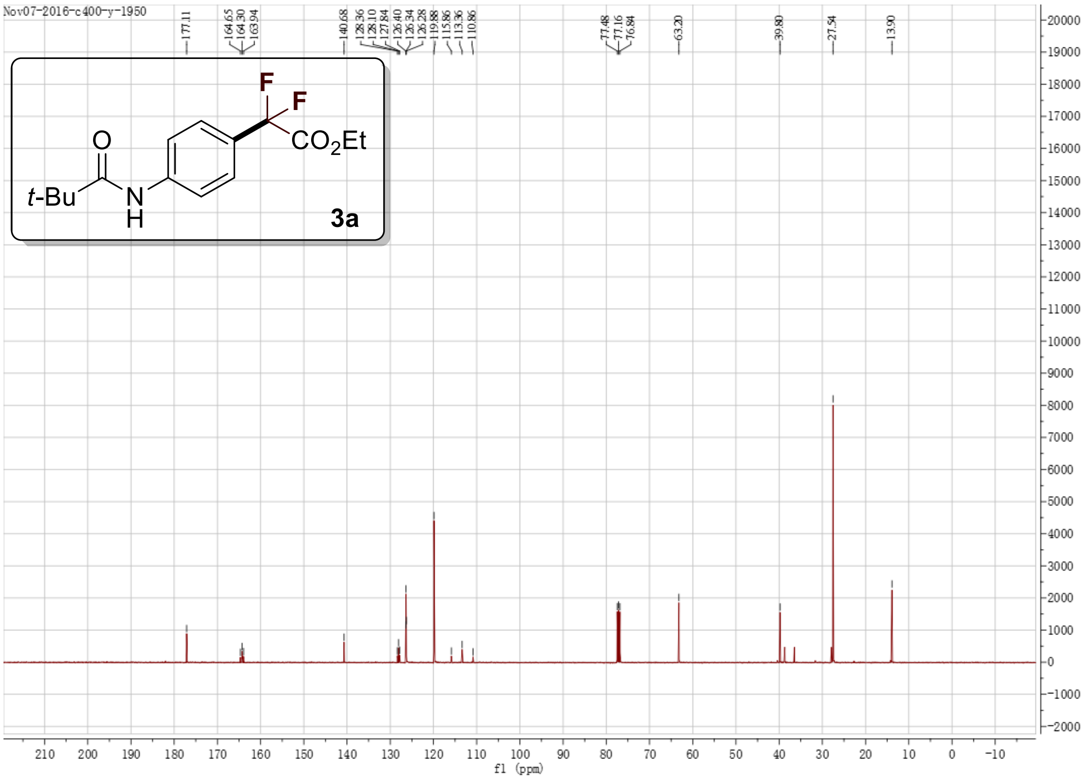

Supplementary Figure 52. ^{13}**C NMR spectra for 3a**

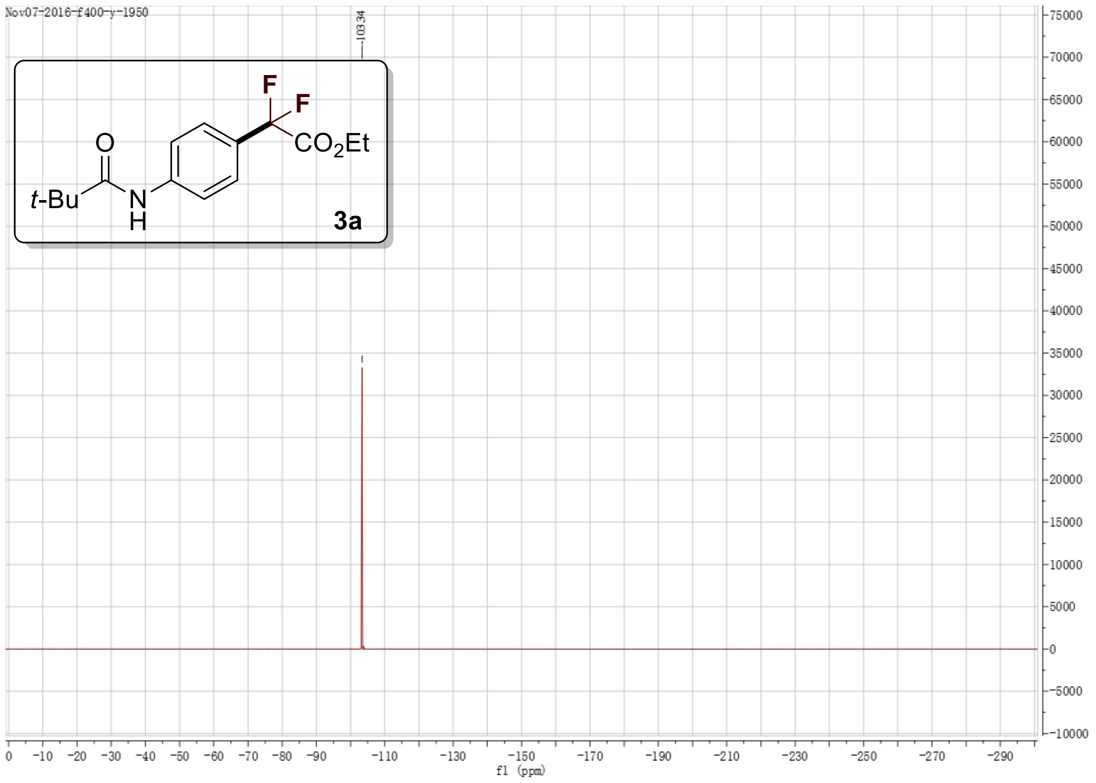

Supplementary Figure 53. ^{19}F NMR spectra for 3a

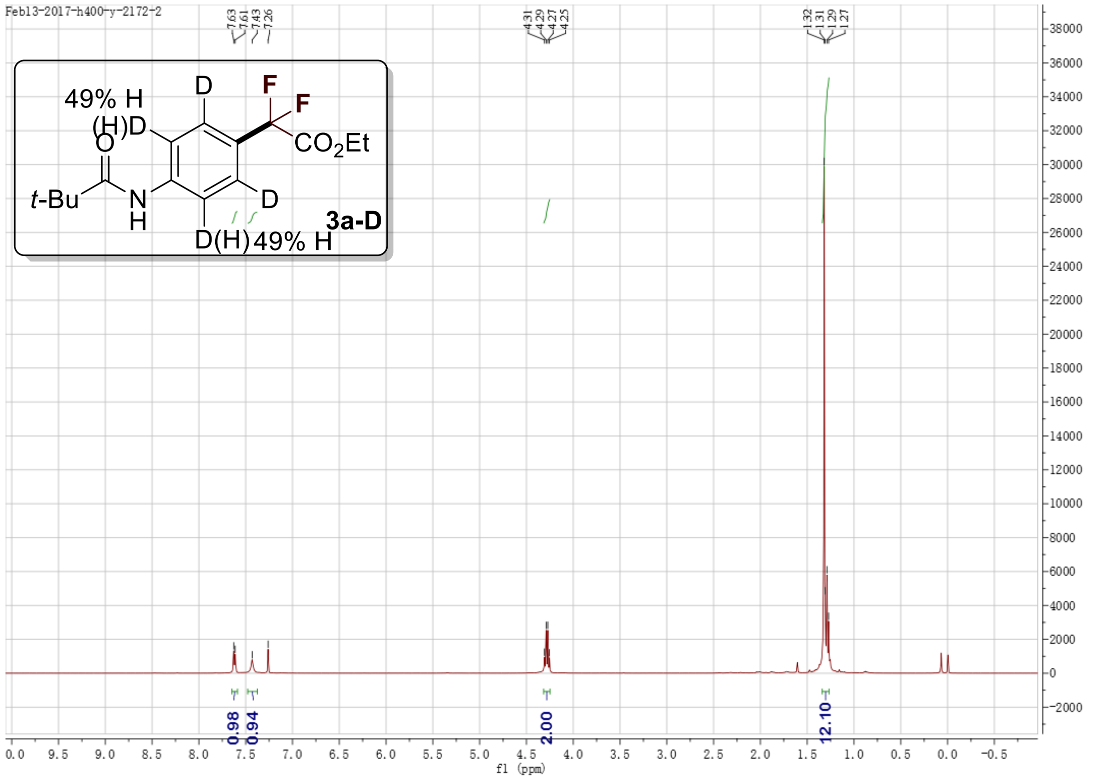

Supplementary Figure 54. ^{1}H NMR spectra for 3a-[D]

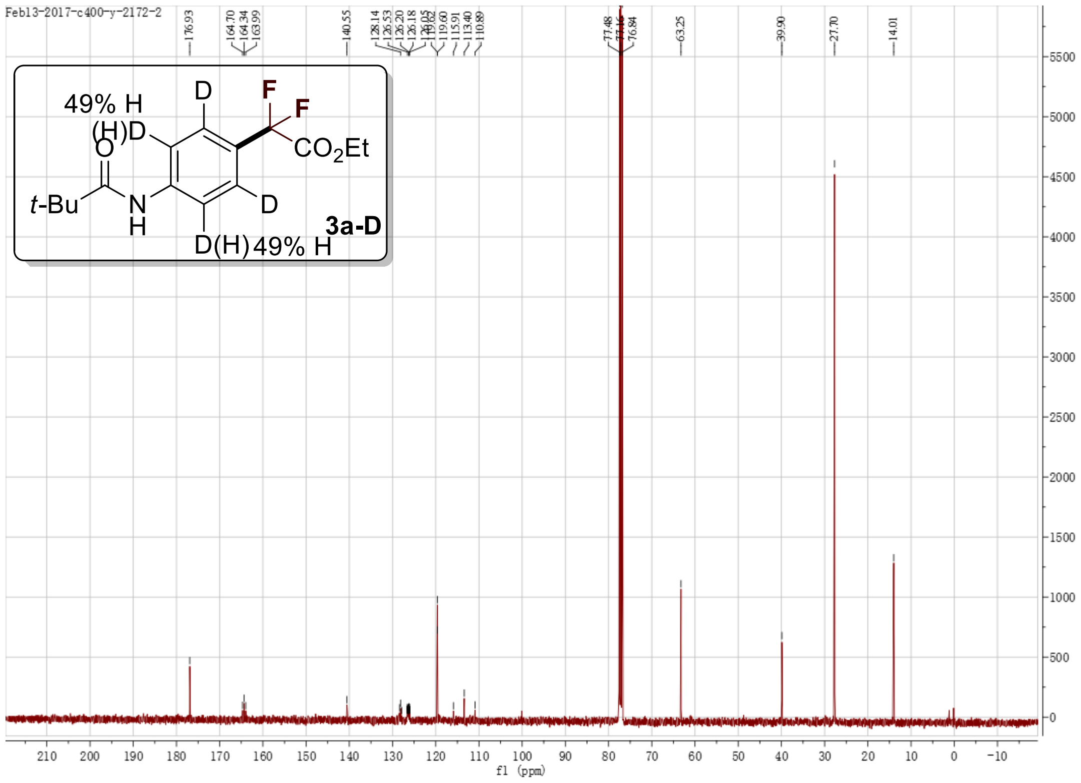

Supplementary Figure 55. ^{13}**C NMR spectra for 3a-[D]**

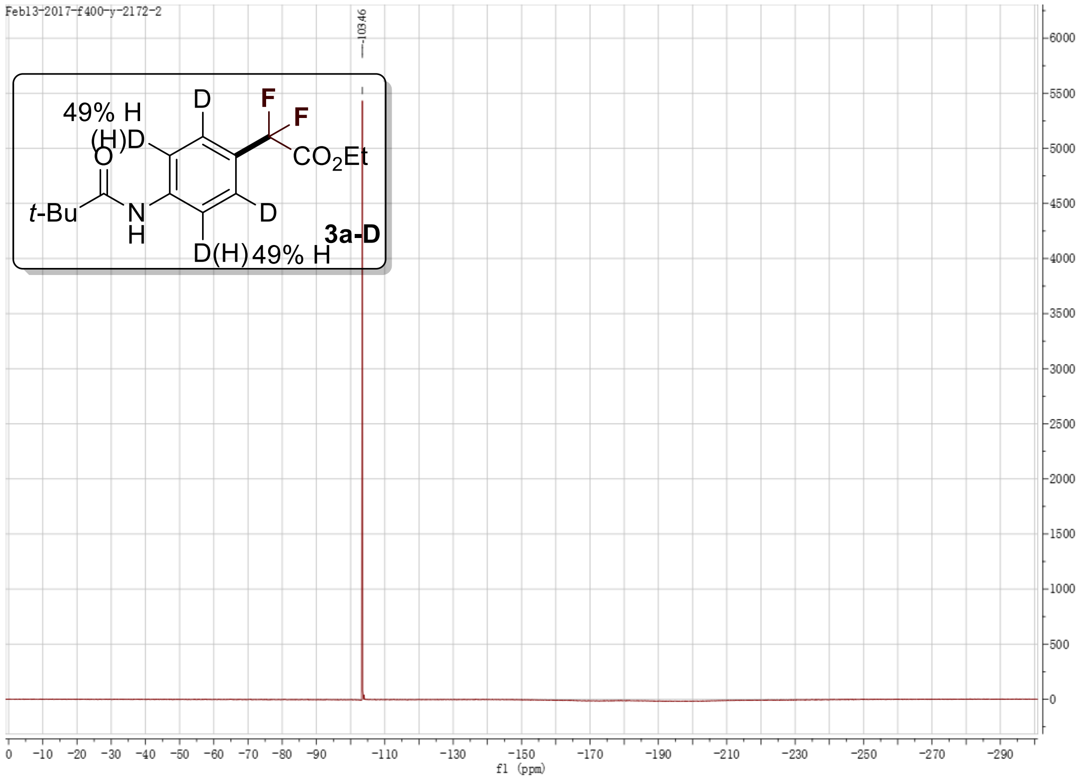

Supplementary Figure 56. ^{19}**F NMR spectra for 3a-[D]**

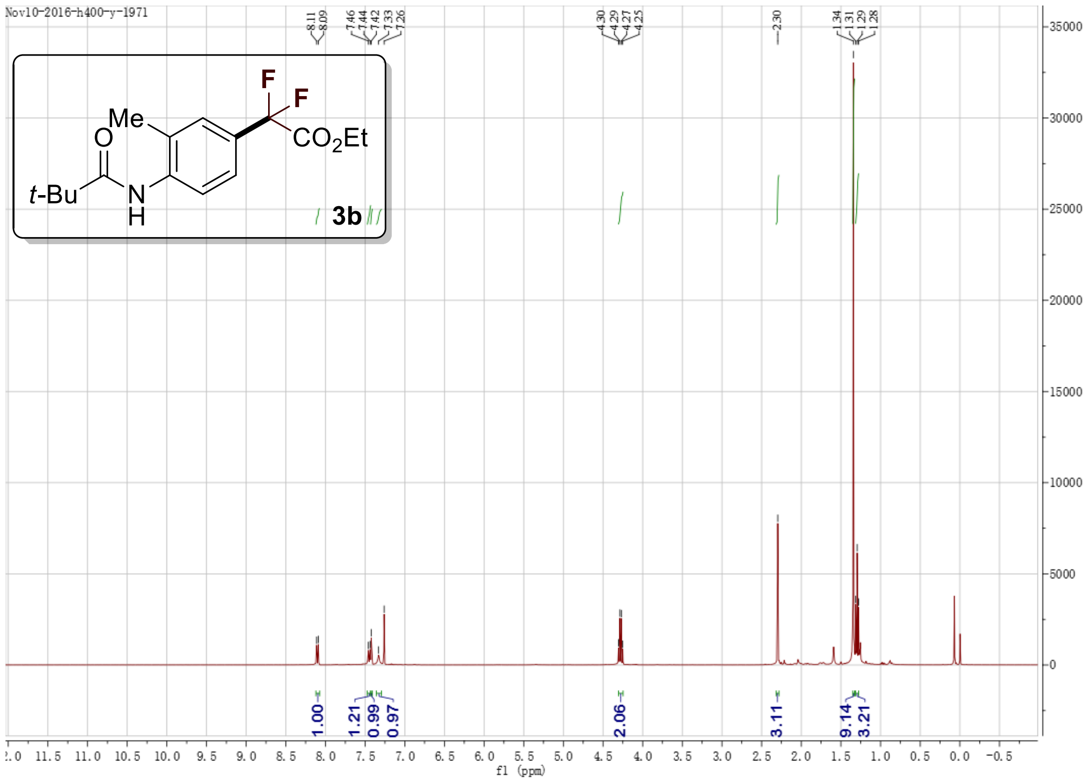

Supplementary Figure 57. ^{1}H NMR spectra for 3b

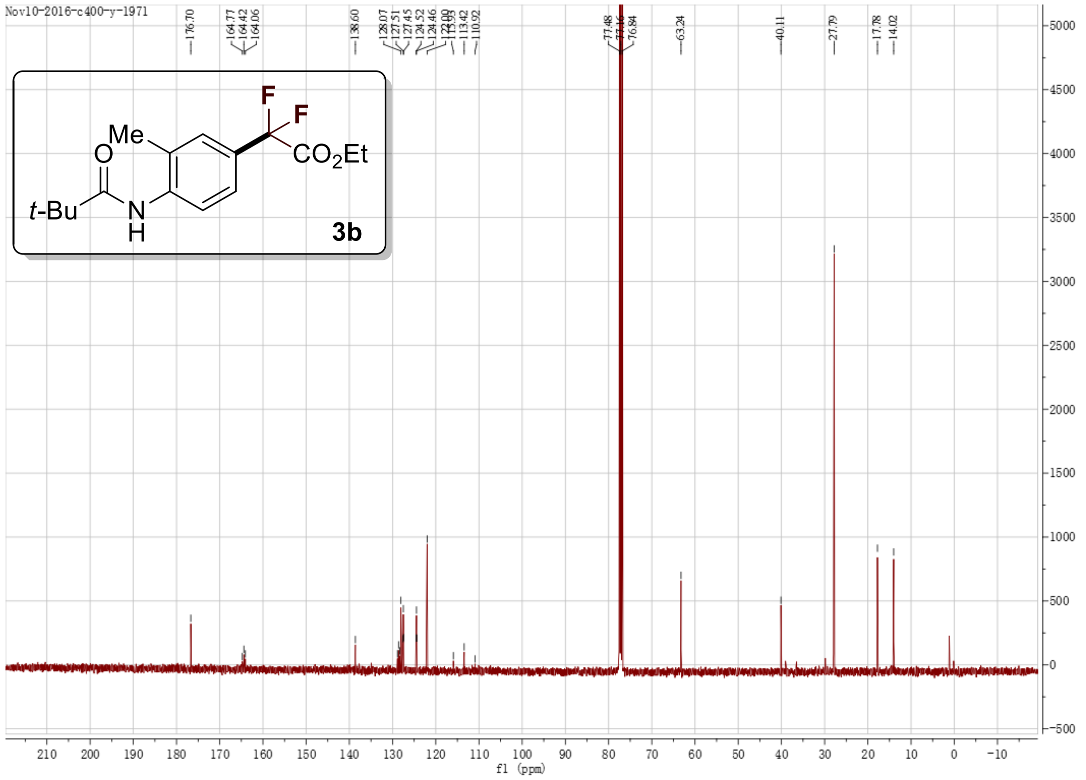

Supplementary Figure 58. ^{13}C NMR spectra for 3b

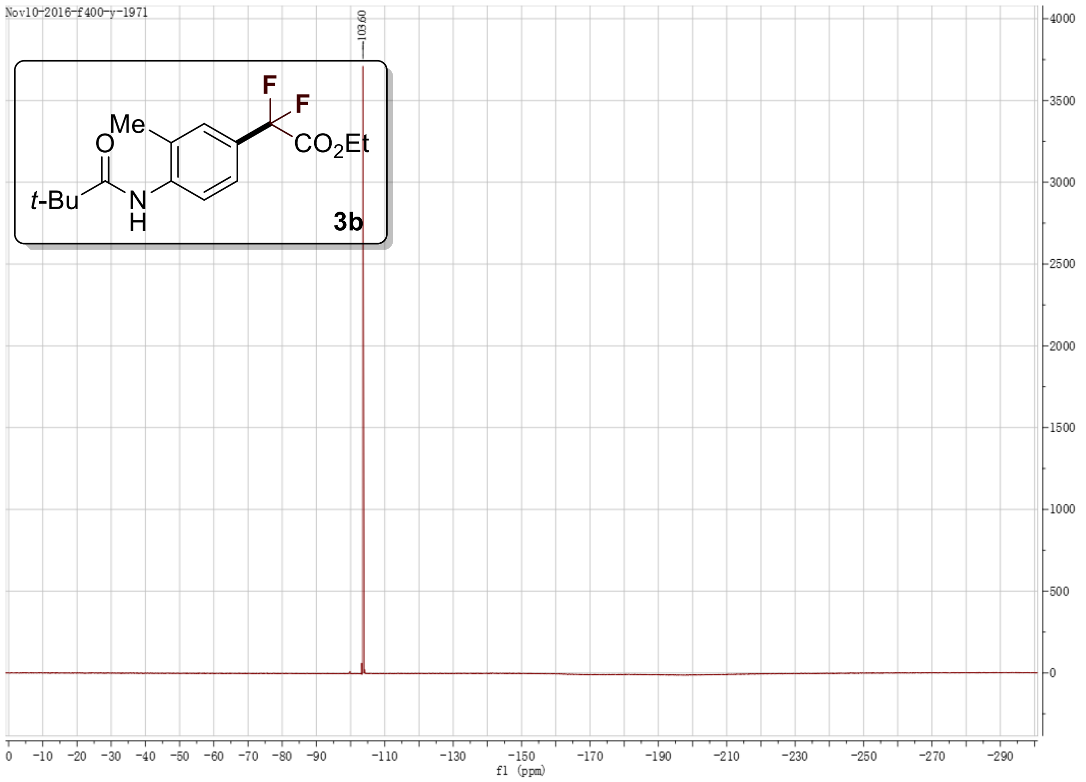

Supplementary Figure 59. ^{19}F NMR spectra for 3b

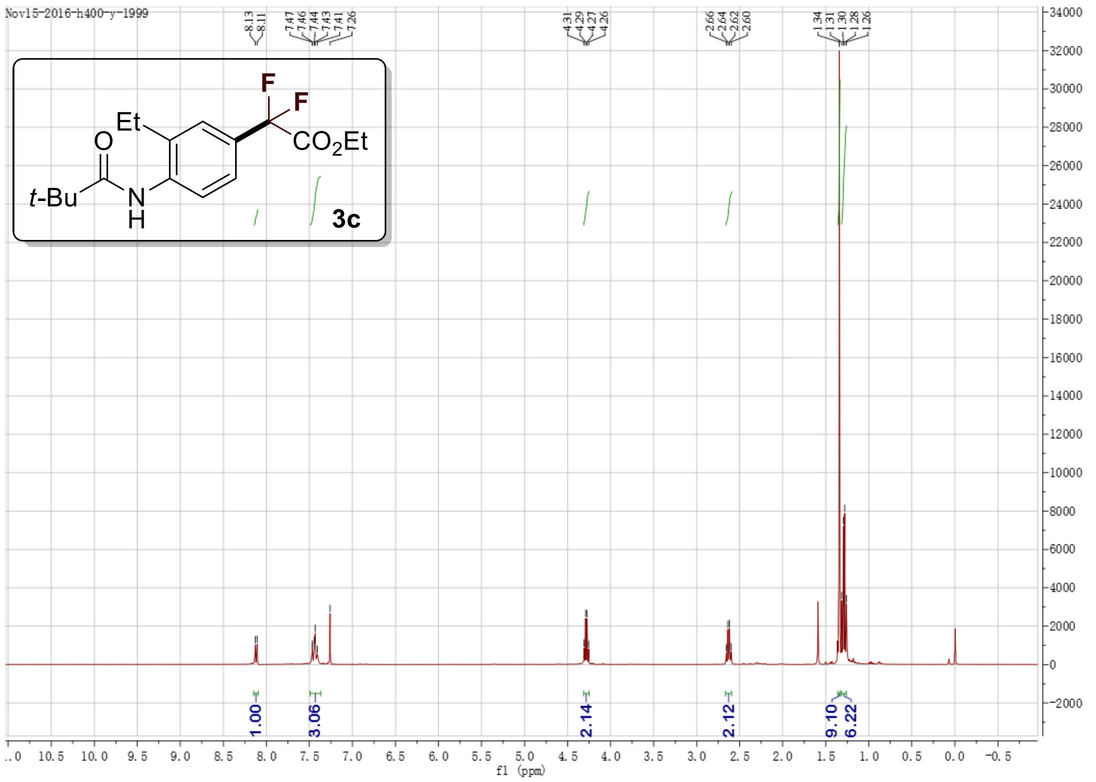

Supplementary Figure 60. ^{1}H NMR spectra for 3c

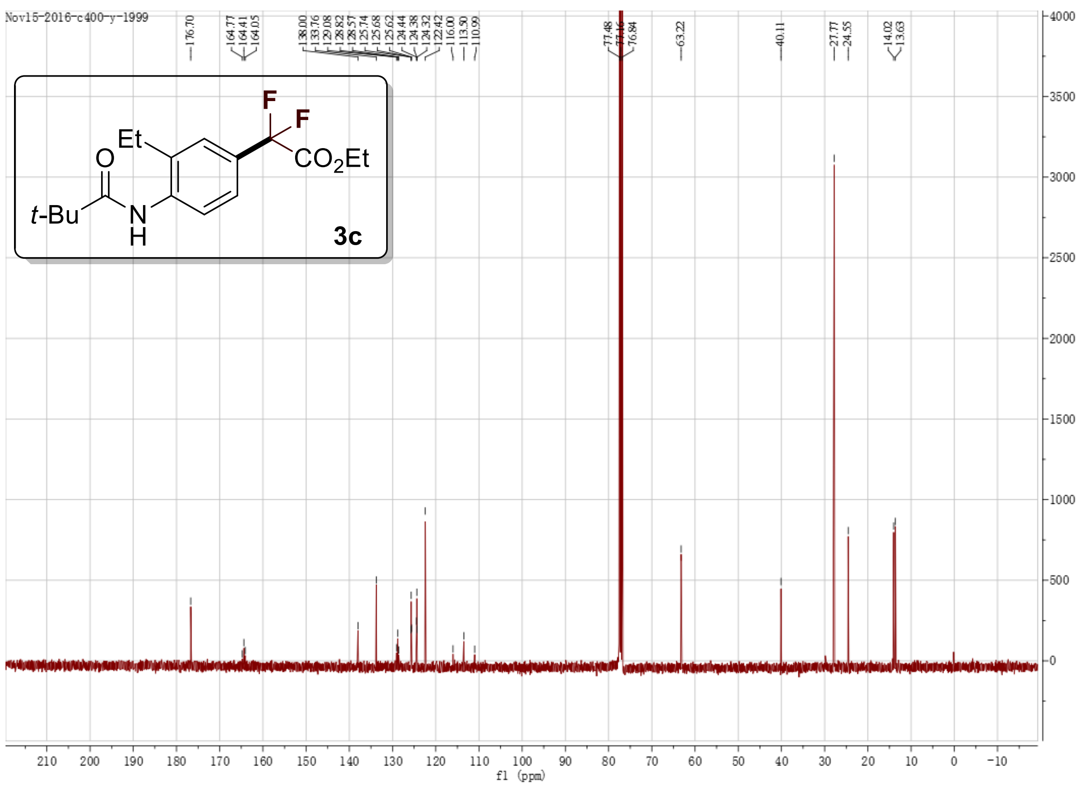

Supplementary Figure 61. **^{13}C NMR spectra for 3c**

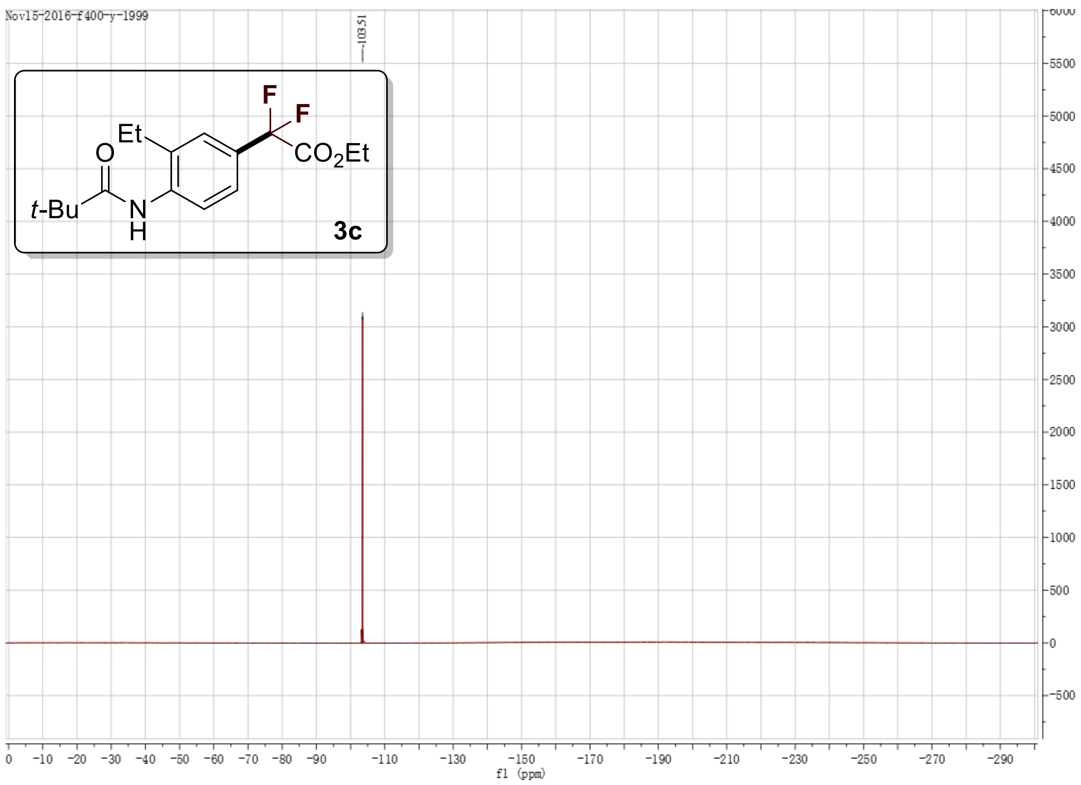

Supplementary Figure 62. **^{19}F NMR spectra for 3c**

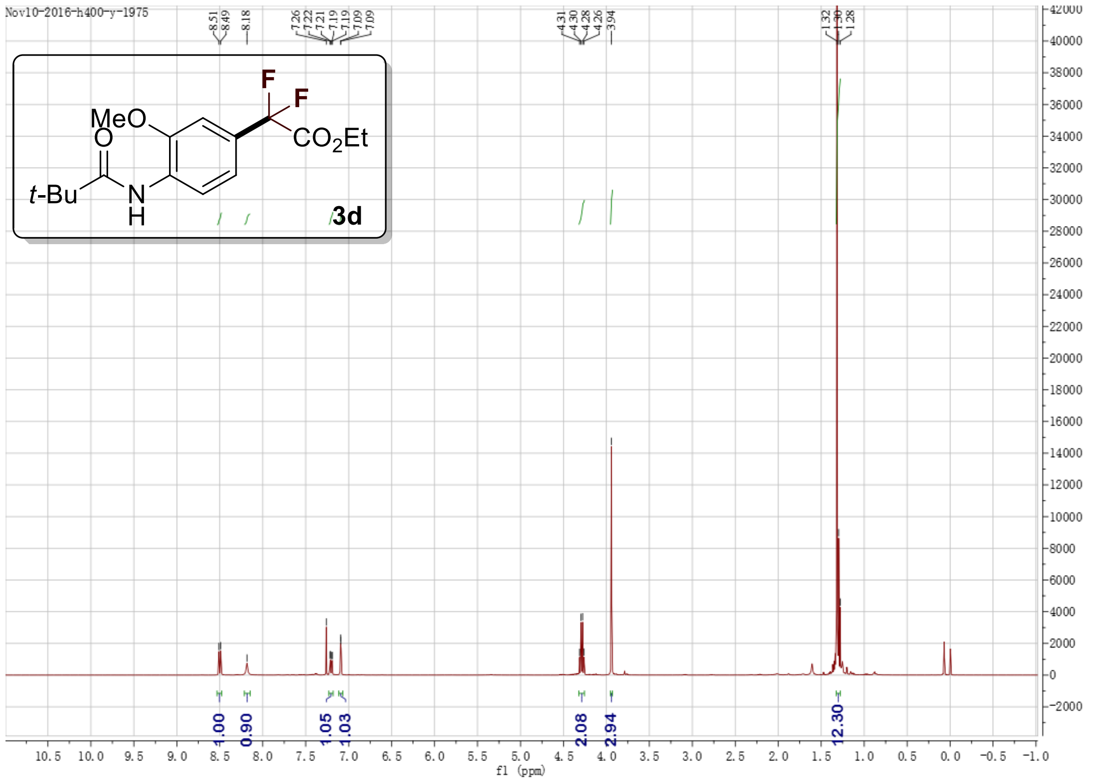

Supplementary Figure 63. ^{1}**H NMR spectra for 3d**

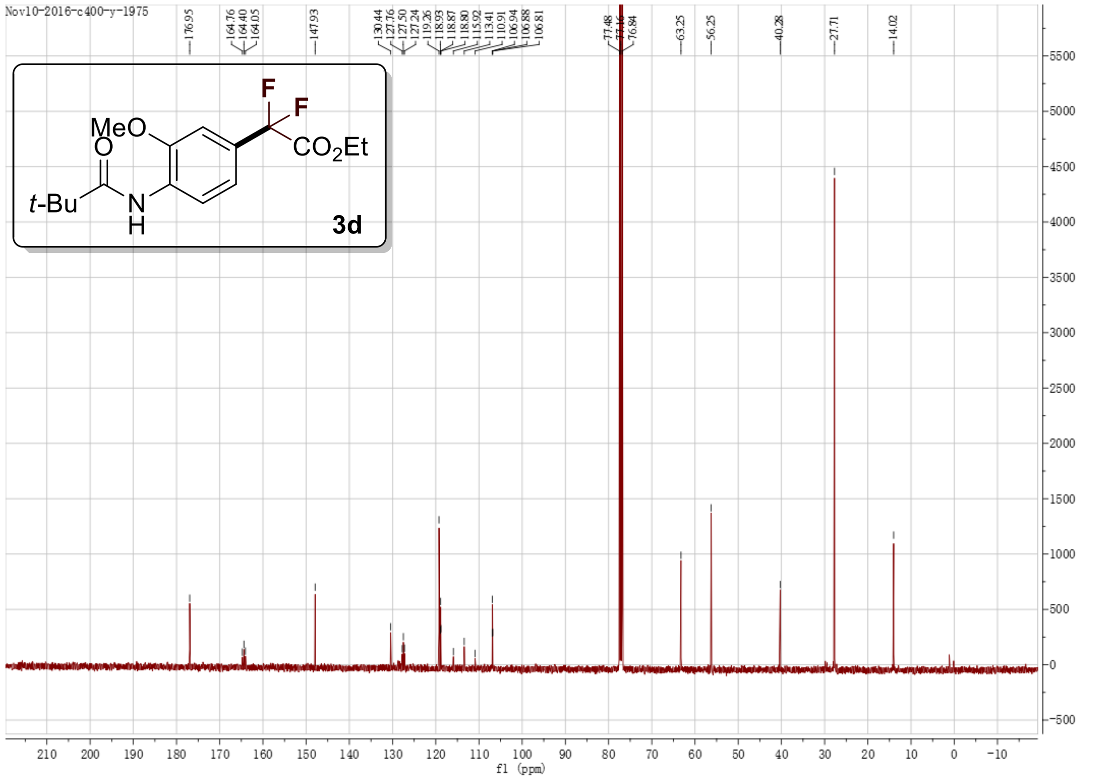

Supplementary Figure 64. ^{13}**C NMR spectra for 3d**

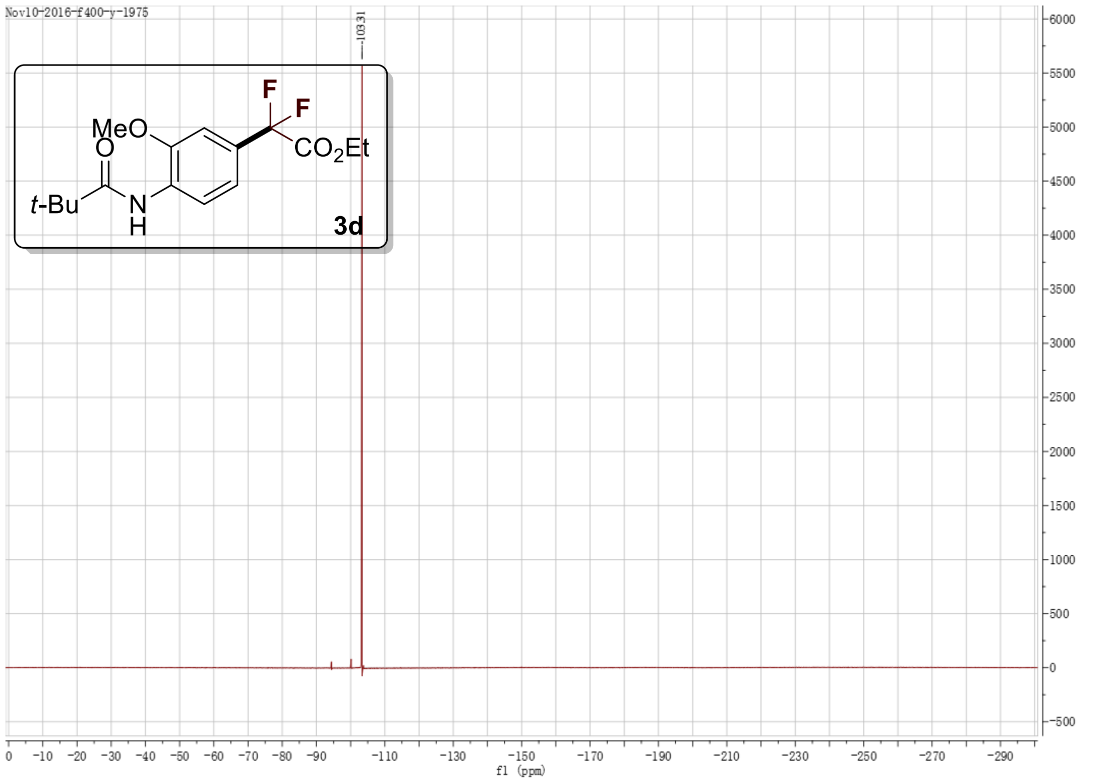

Supplementary Figure 65. ^{19}F NMR spectra for 3d

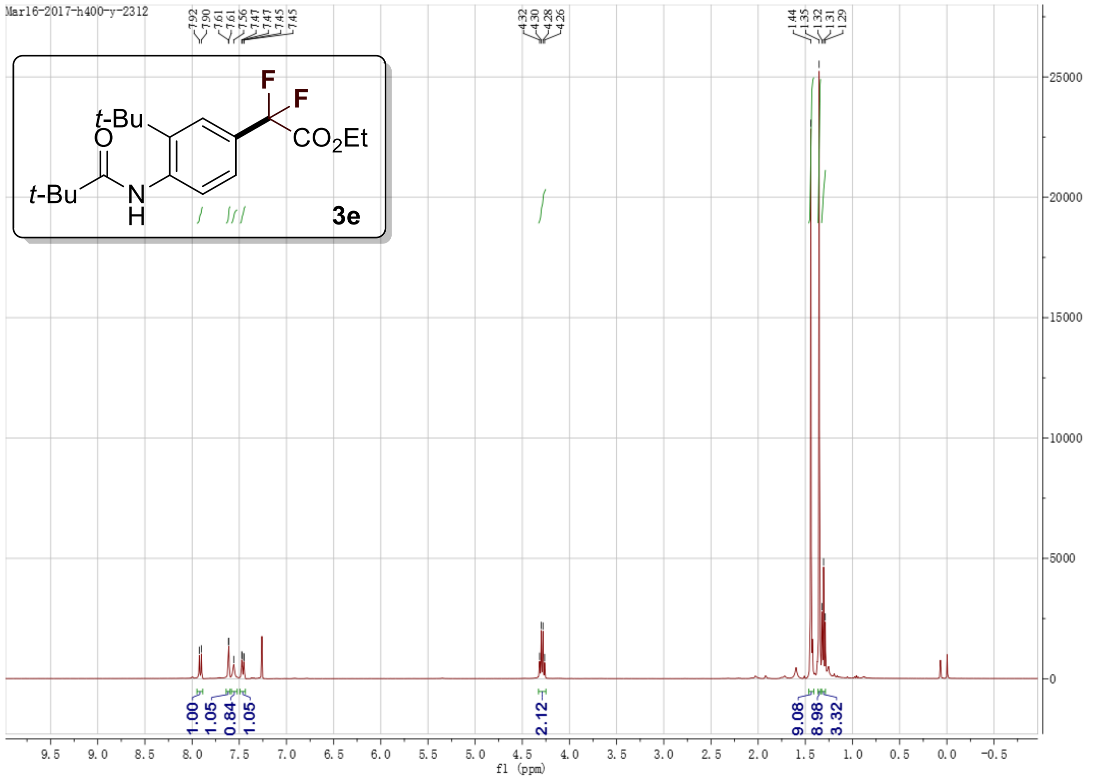

Supplementary Figure 66. ^{1}H NMR spectra for 3e

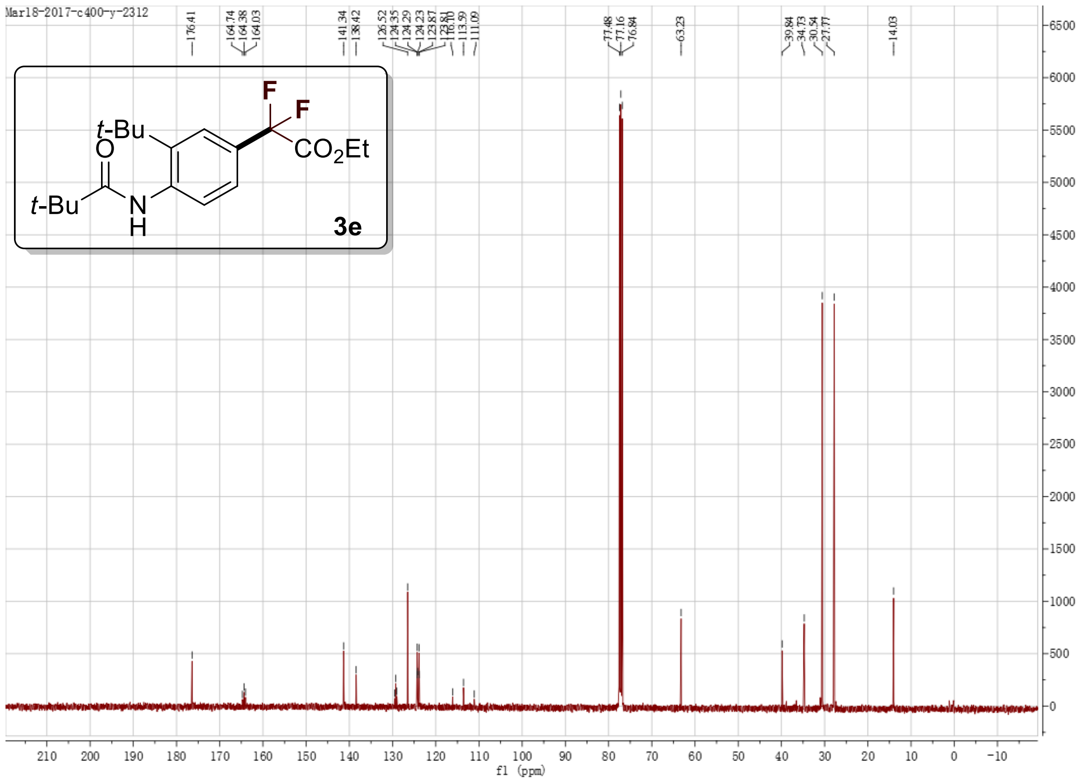

Supplementary Figure 67. ^{13}C NMR spectra for 3e

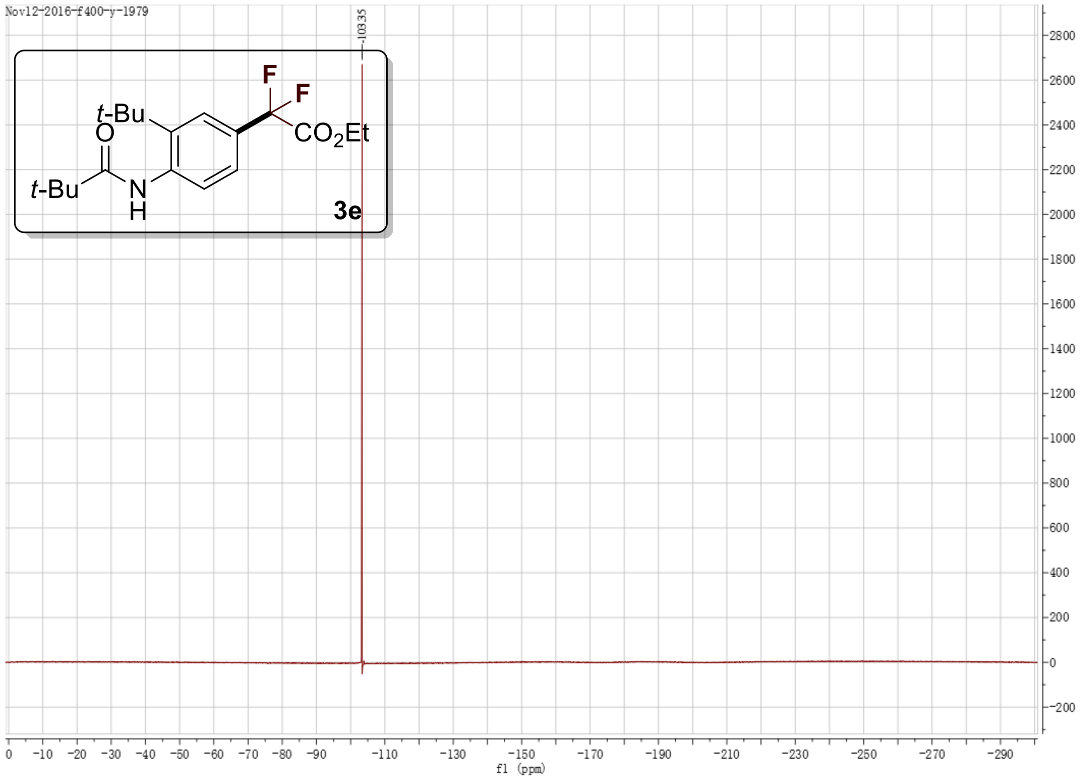

Supplementary Figure 68. ^{19}F NMR spectra for 3e

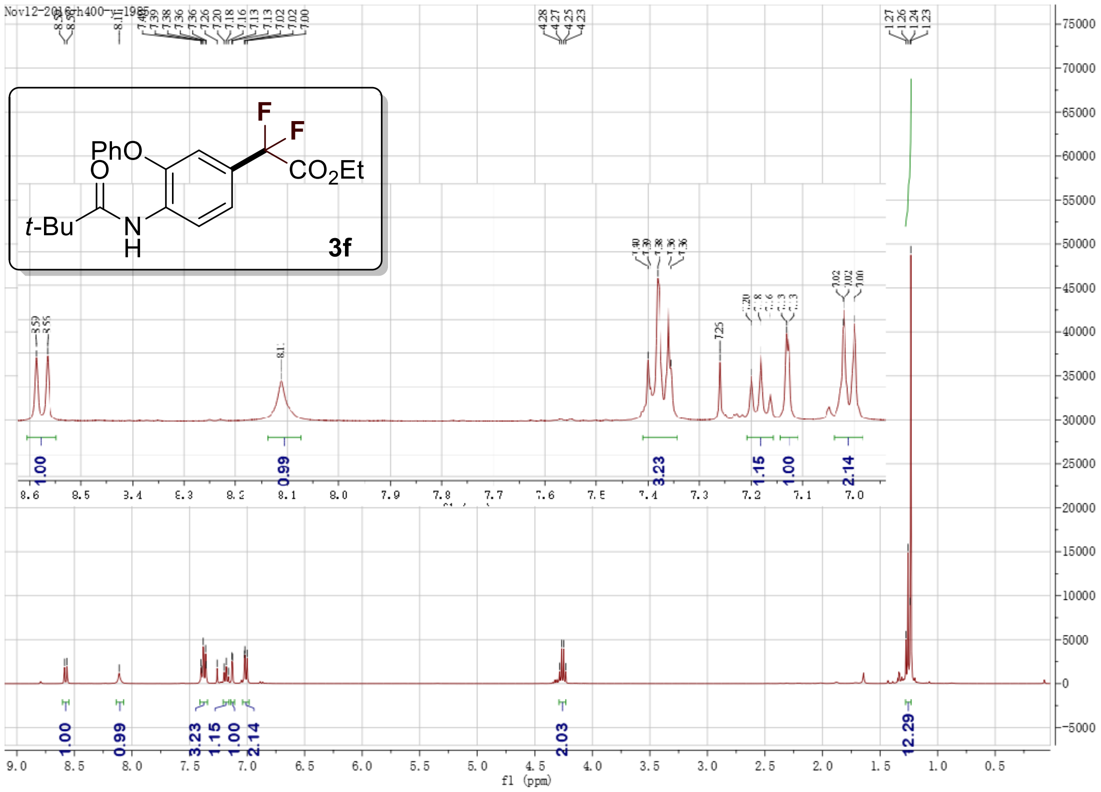

Supplementary Figure 69. ^{1}H NMR spectra for 3f

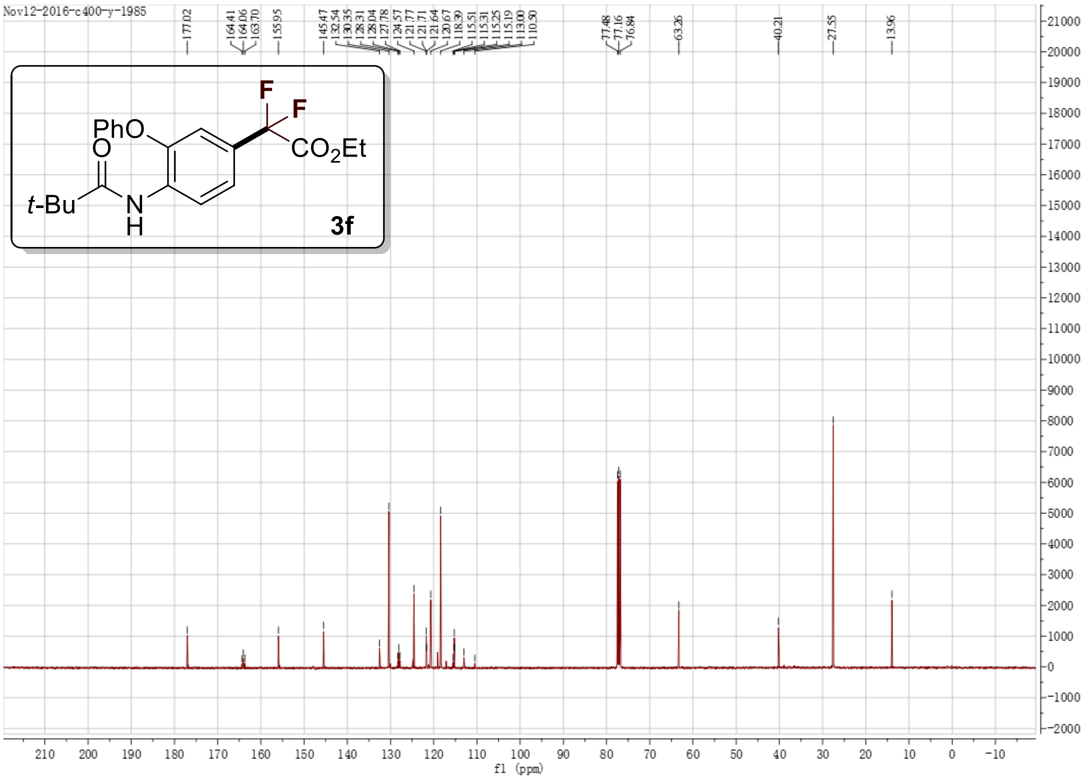

Supplementary Figure 70. ^{13}C NMR spectra for 3f

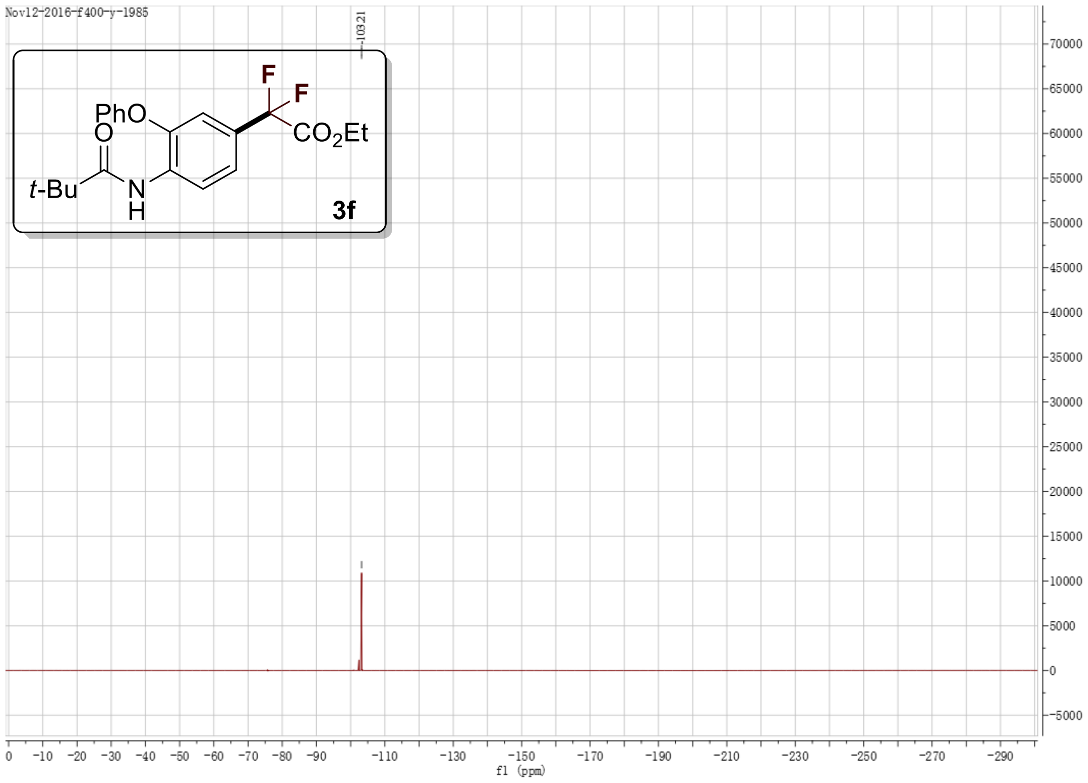

Supplementary Figure 71. ^{19}F NMR spectra for 3f

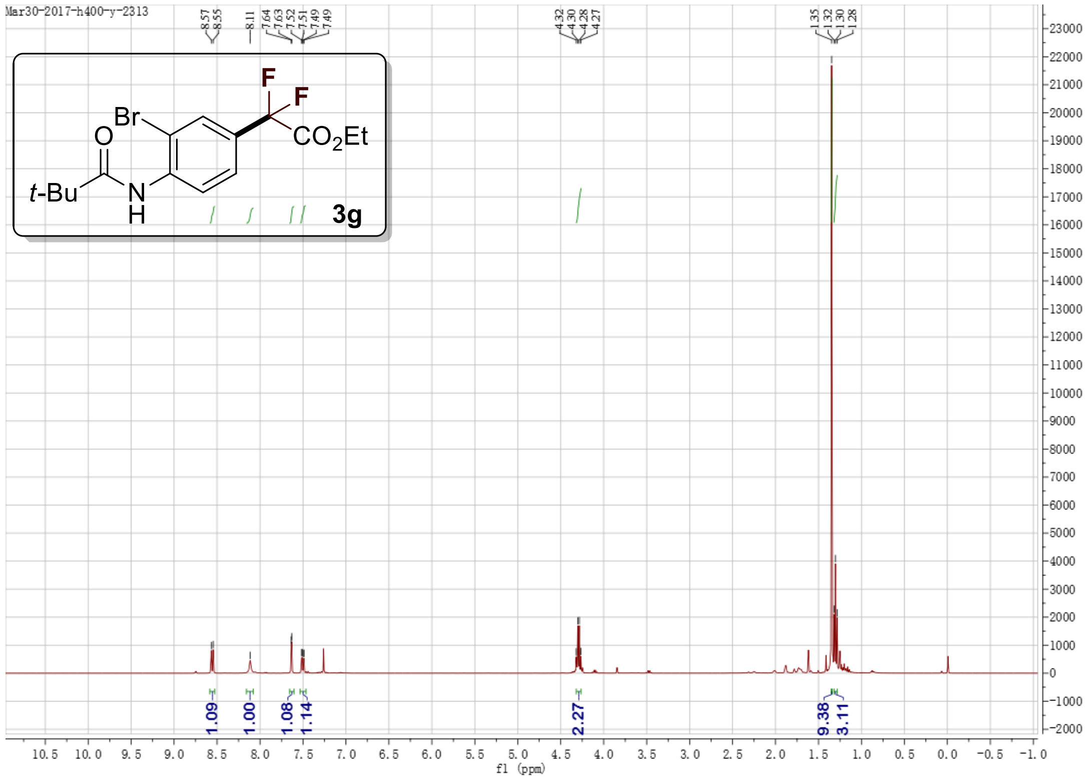

Supplementary Figure 72. ^{1}H NMR spectra for 3g

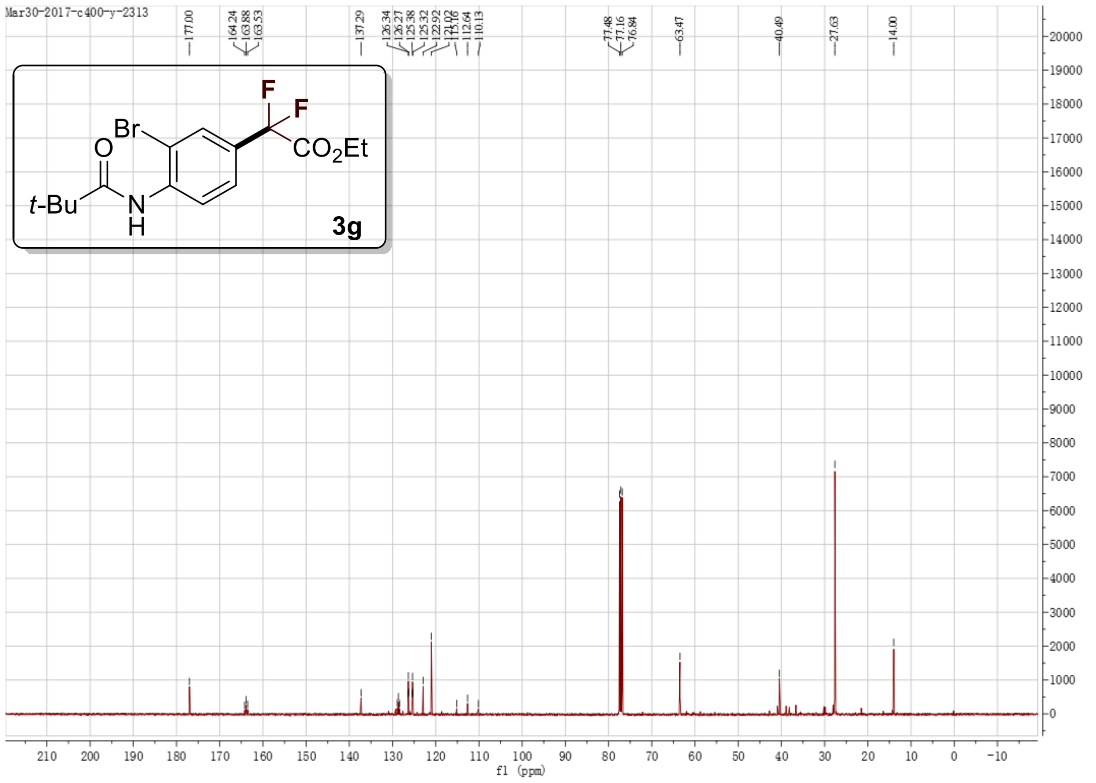

Supplementary Figure 73. ^{13}C NMR spectra for 3g

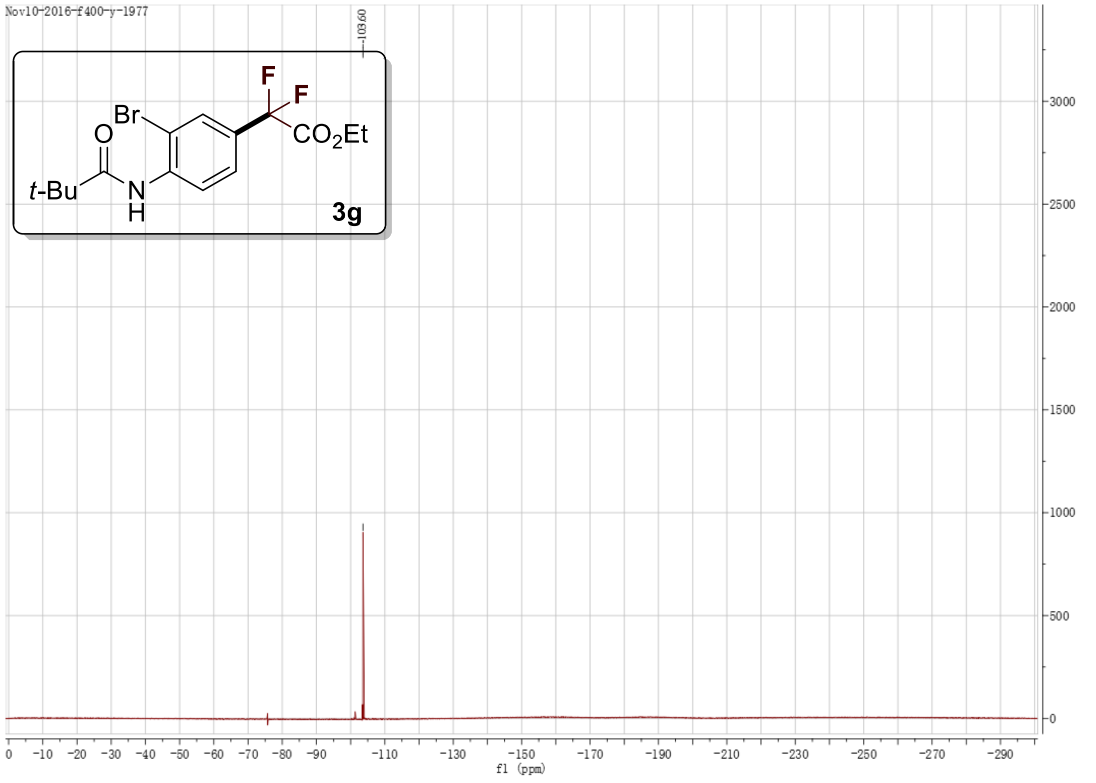

Supplementary Figure 74. ^{19}F NMR spectra for 3g

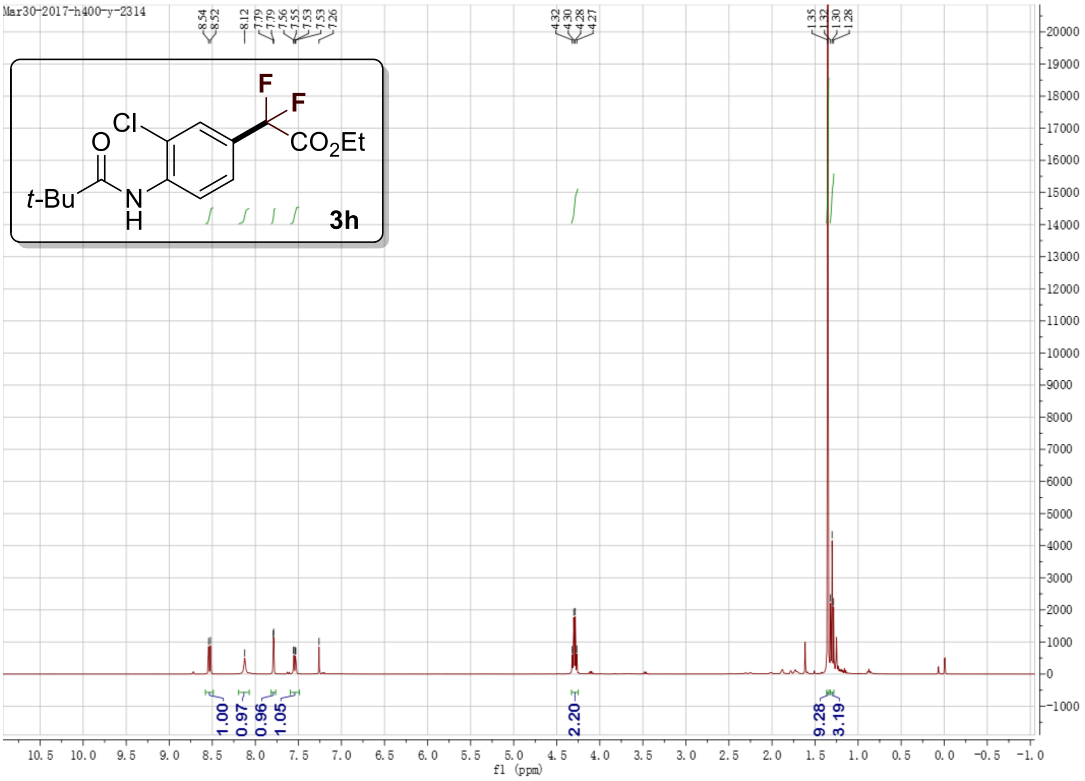

Supplementary Figure 75. ^{1}H NMR spectra for 3h

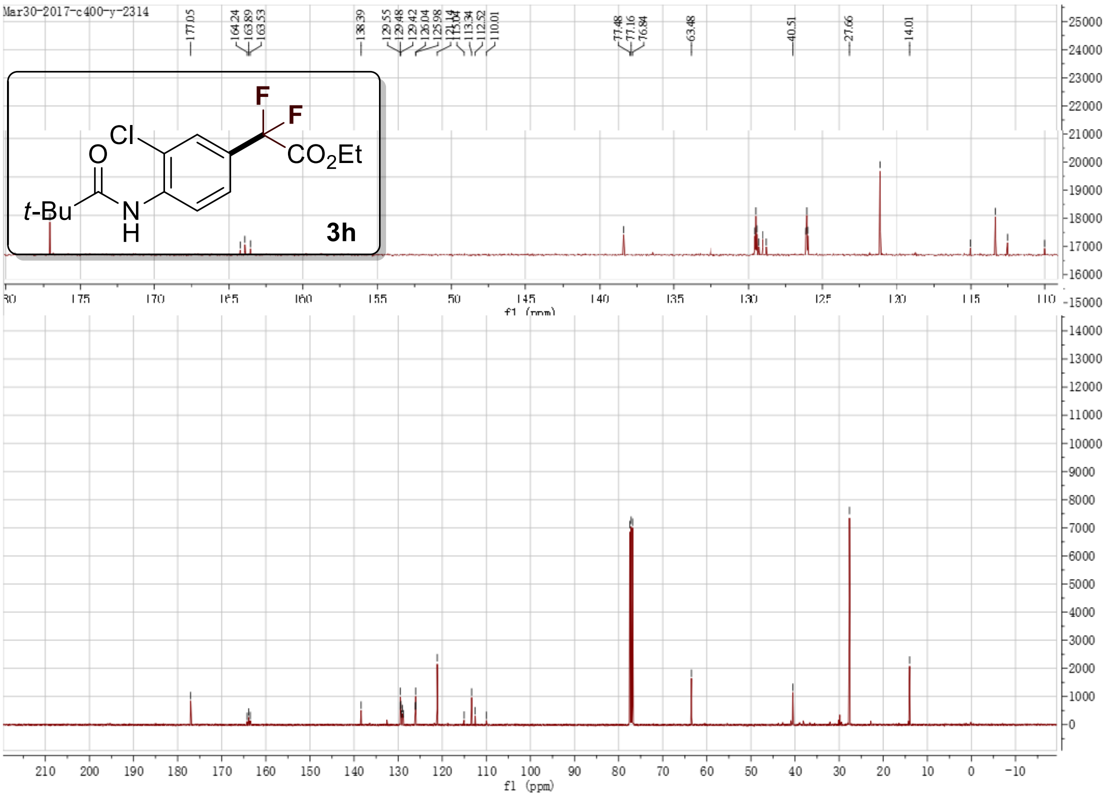

Supplementary Figure 76. ^{13}C NMR spectra for 3h

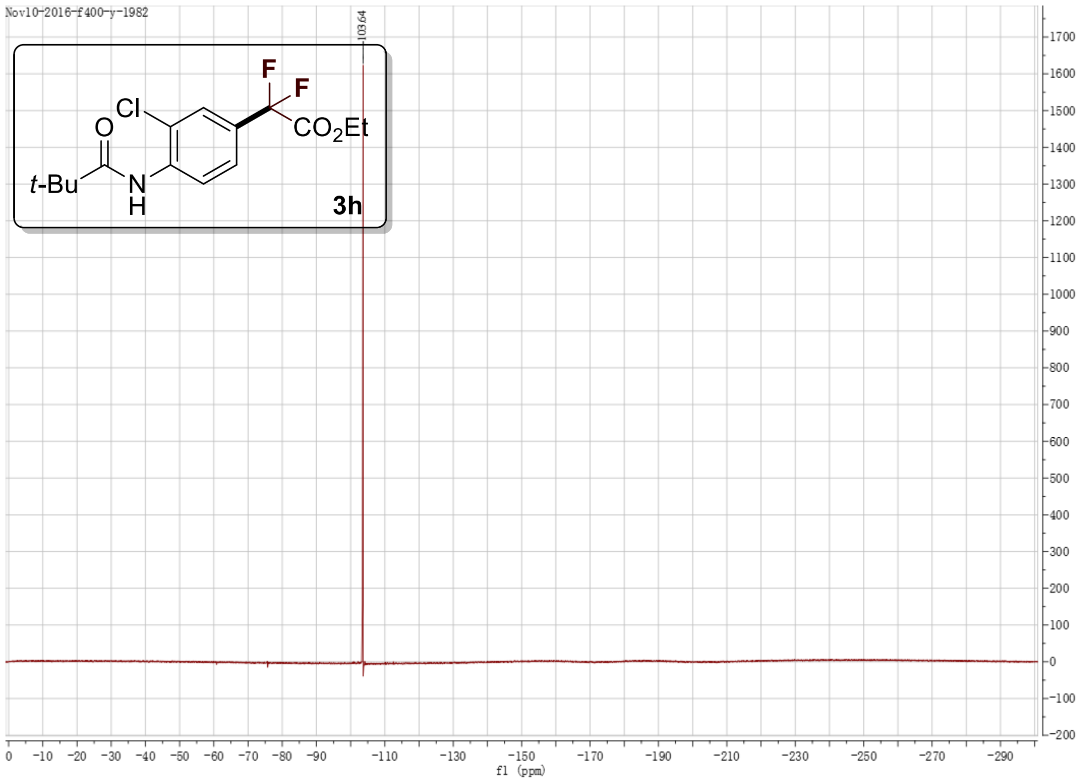

Supplementary Figure 77. ^{19}F NMR spectra for 3h

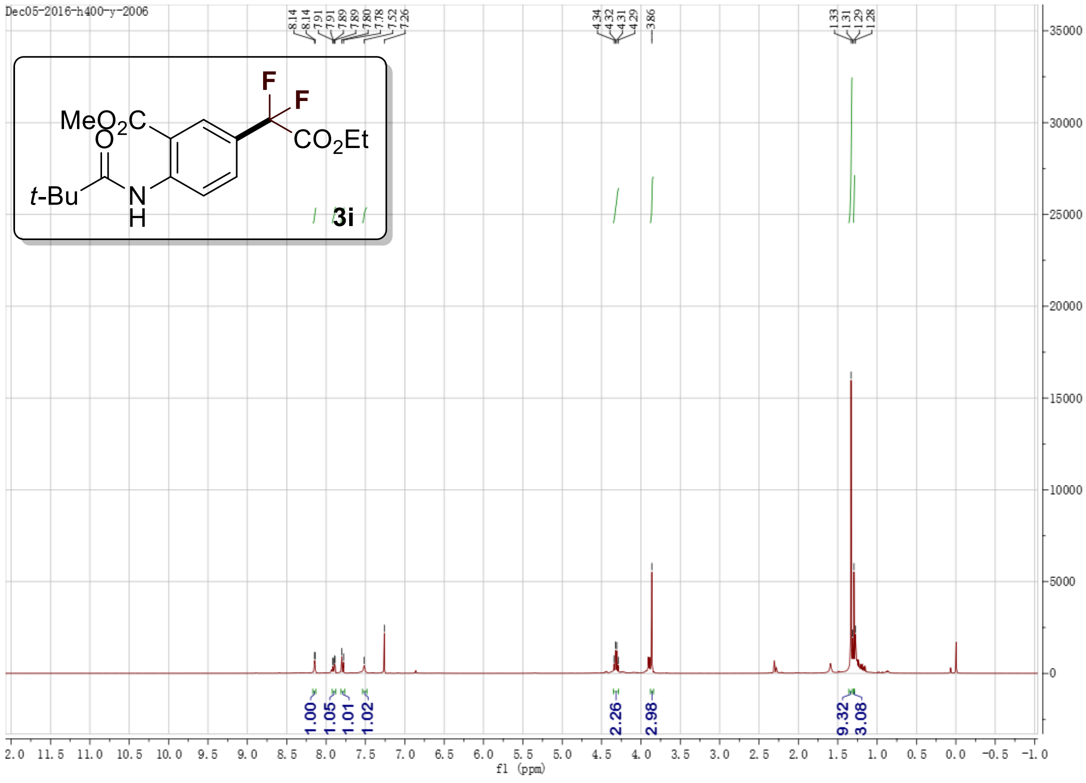

Supplementary Figure 78. ^{1}H NMR spectra for 3i

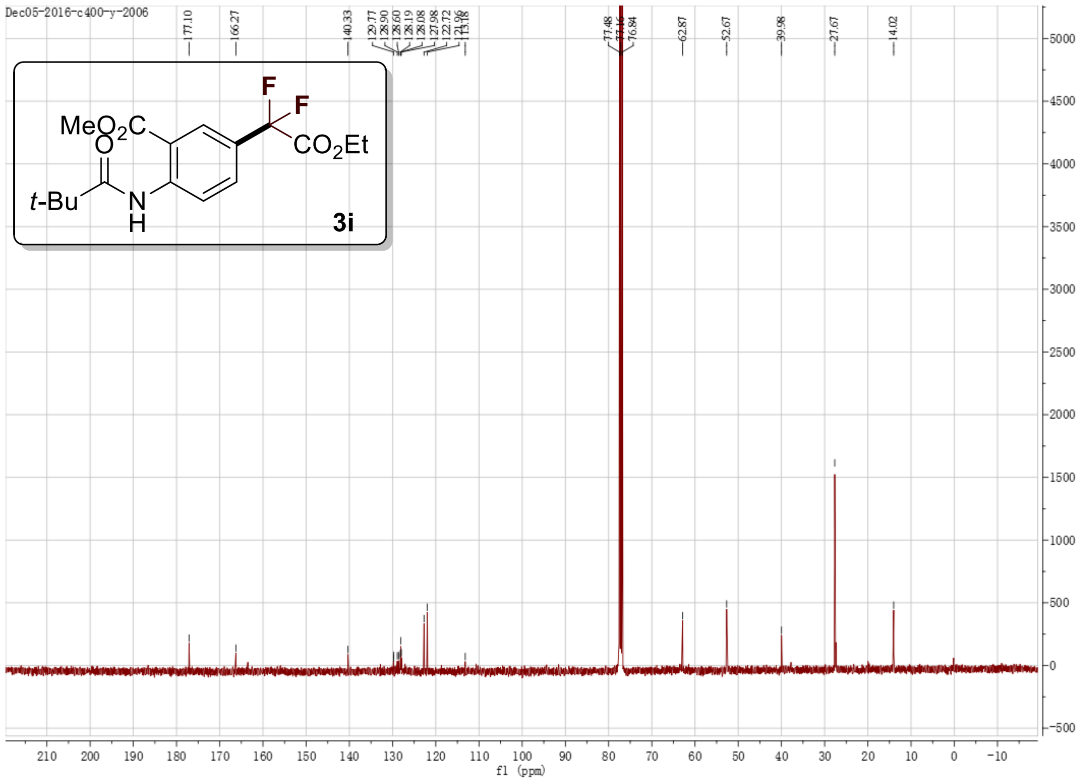

Supplementary Figure 79. ^{13}**C NMR spectra for 3i**

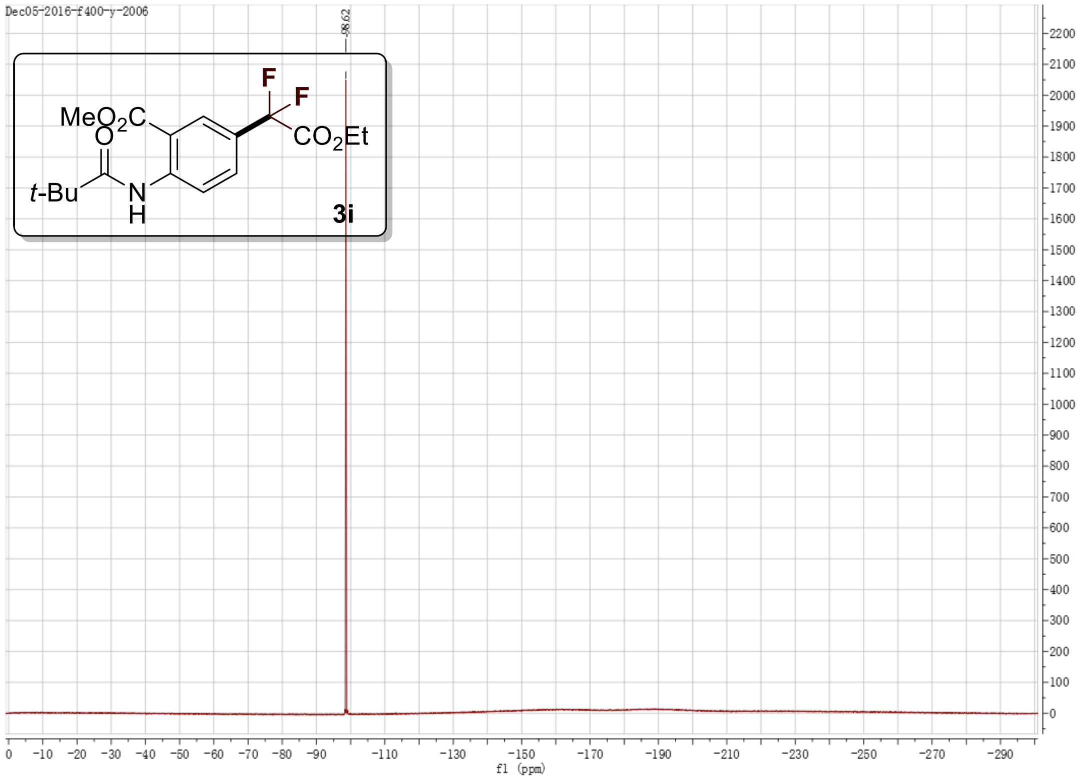

Supplementary Figure 80. ^{19}**F NMR spectra for 3i**

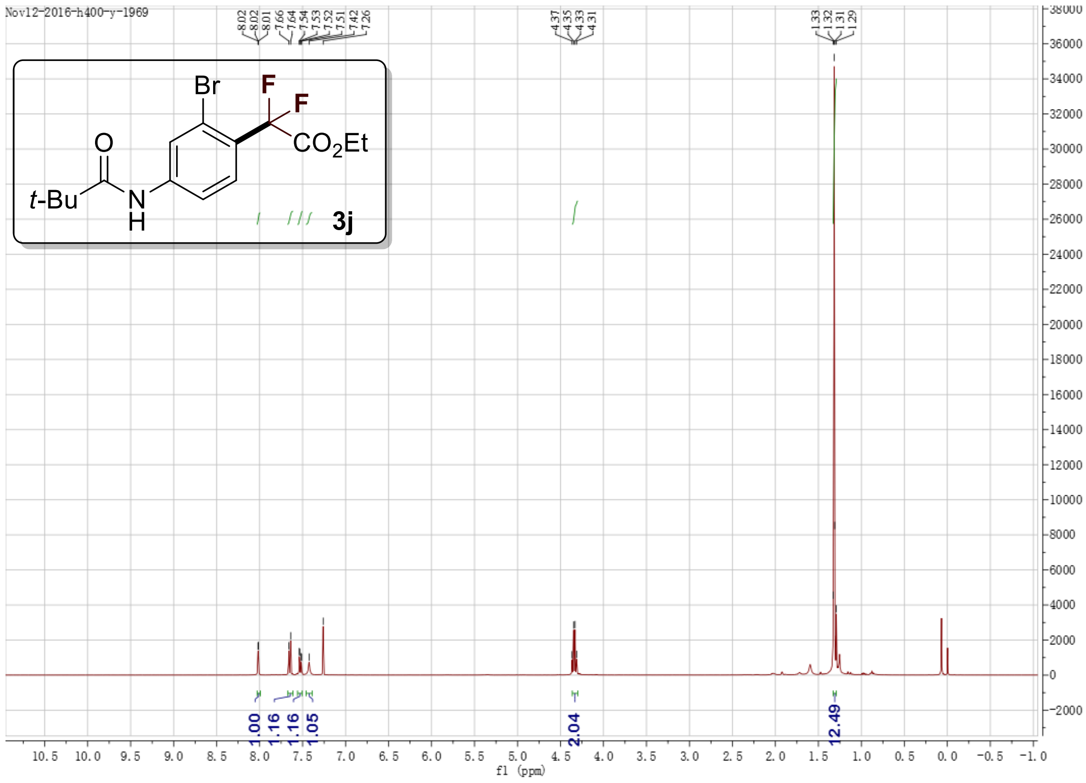

Supplementary Figure 81. ^{1}H NMR spectra for 3j

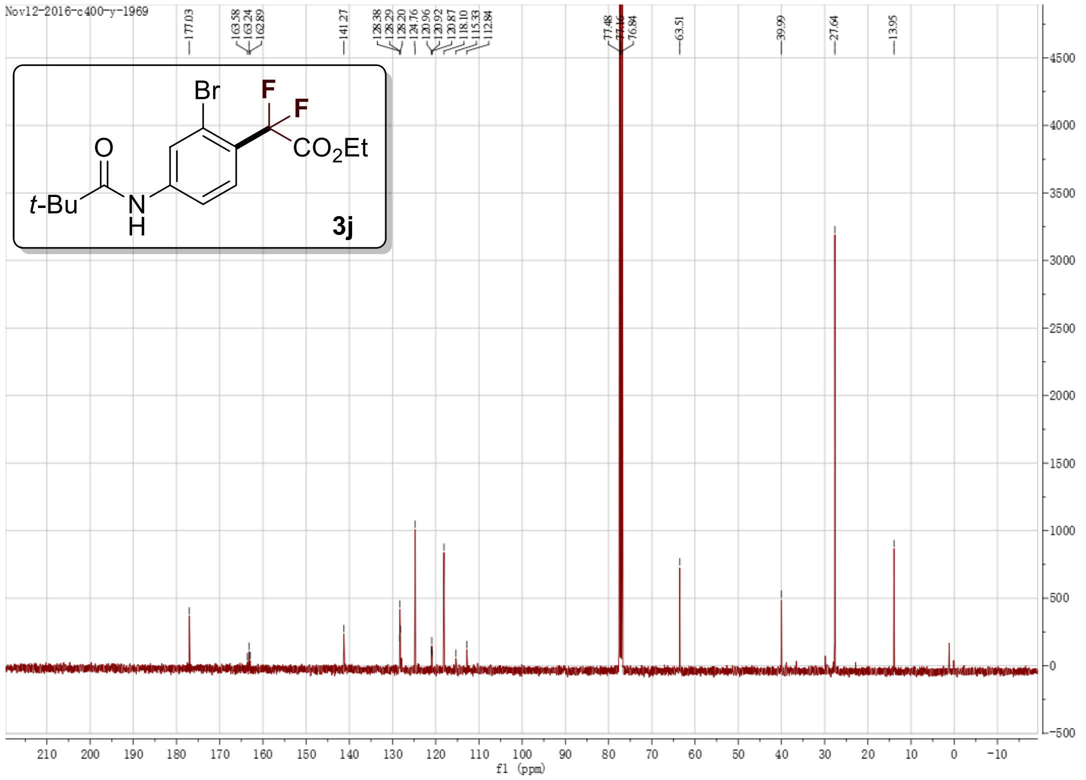

Supplementary Figure 82. ^{13}C NMR spectra for 3j

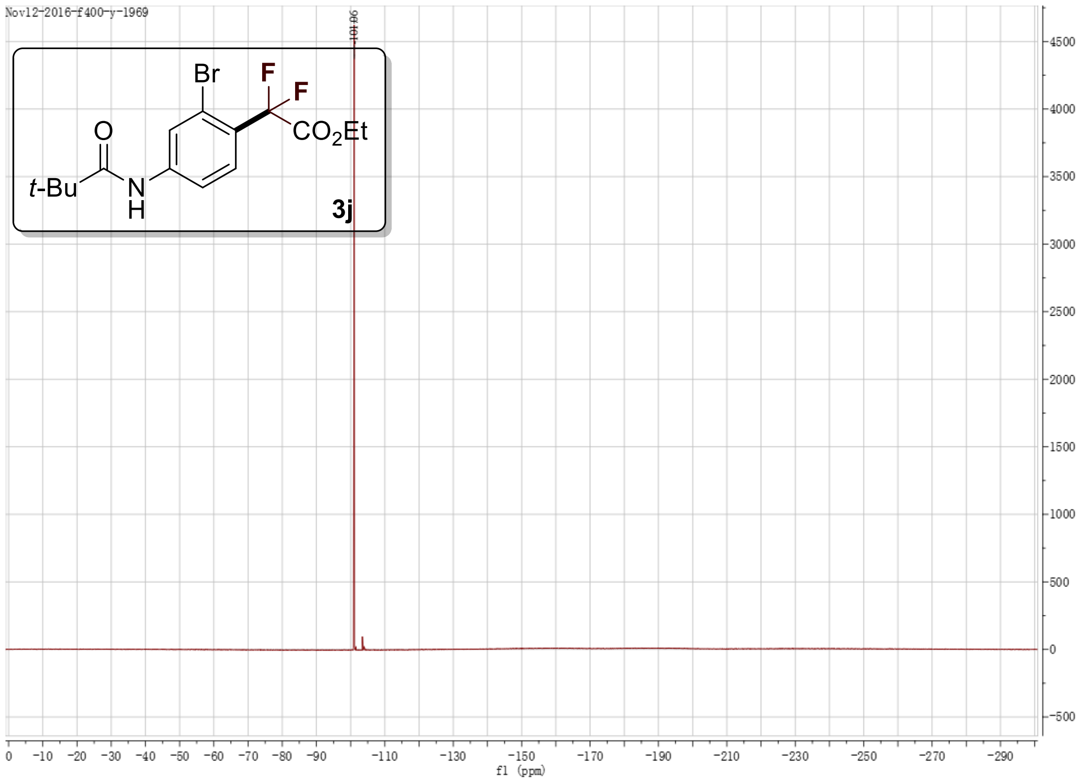

Supplementary Figure 83. ^{19}F NMR spectra for 3j

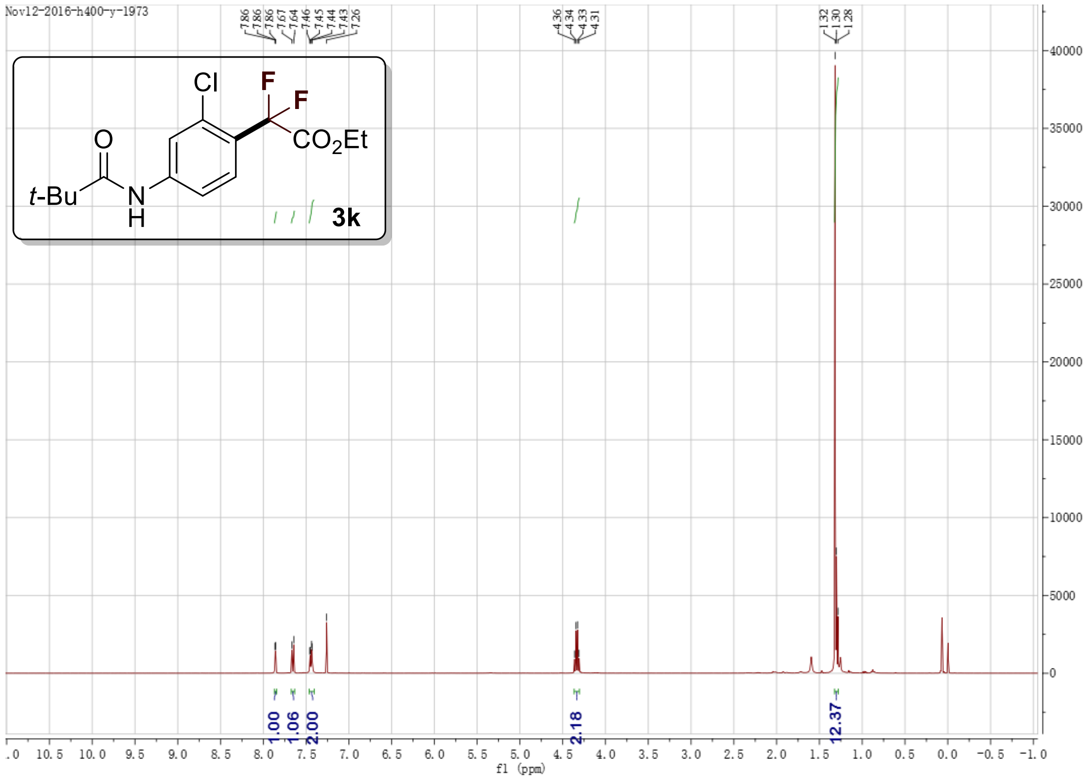

Supplementary Figure 84. ^{1}H NMR spectra for 3k

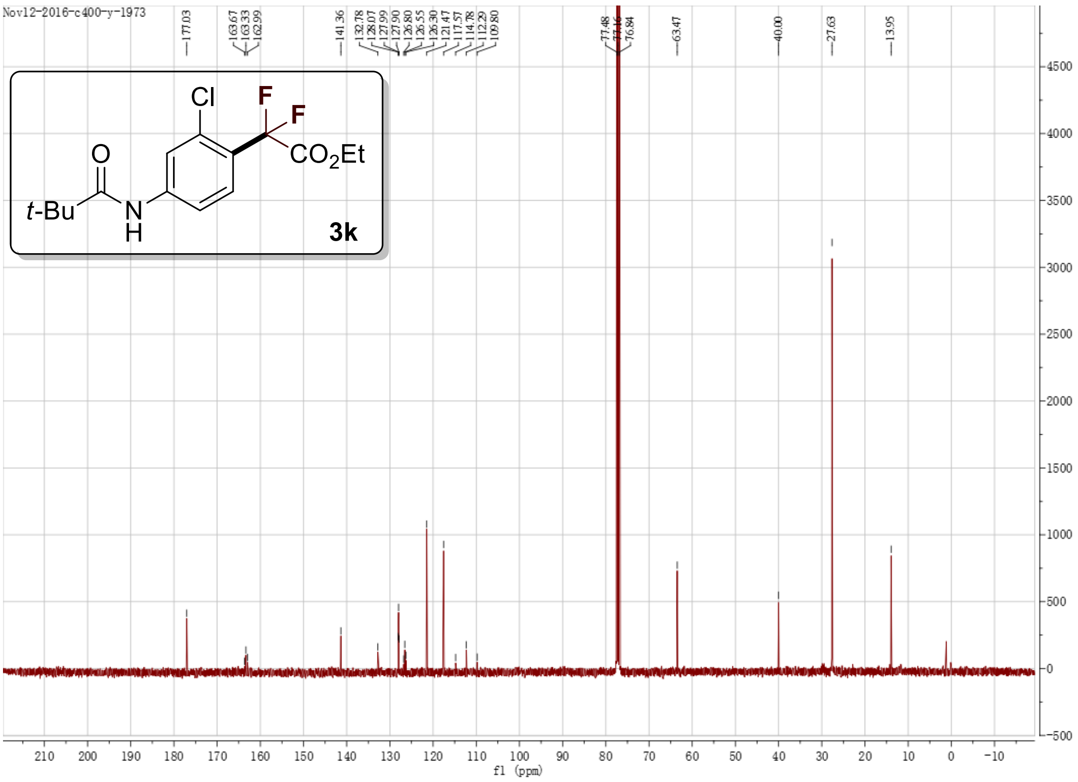

Supplementary Figure 85. ^{13}C NMR spectra for 3k

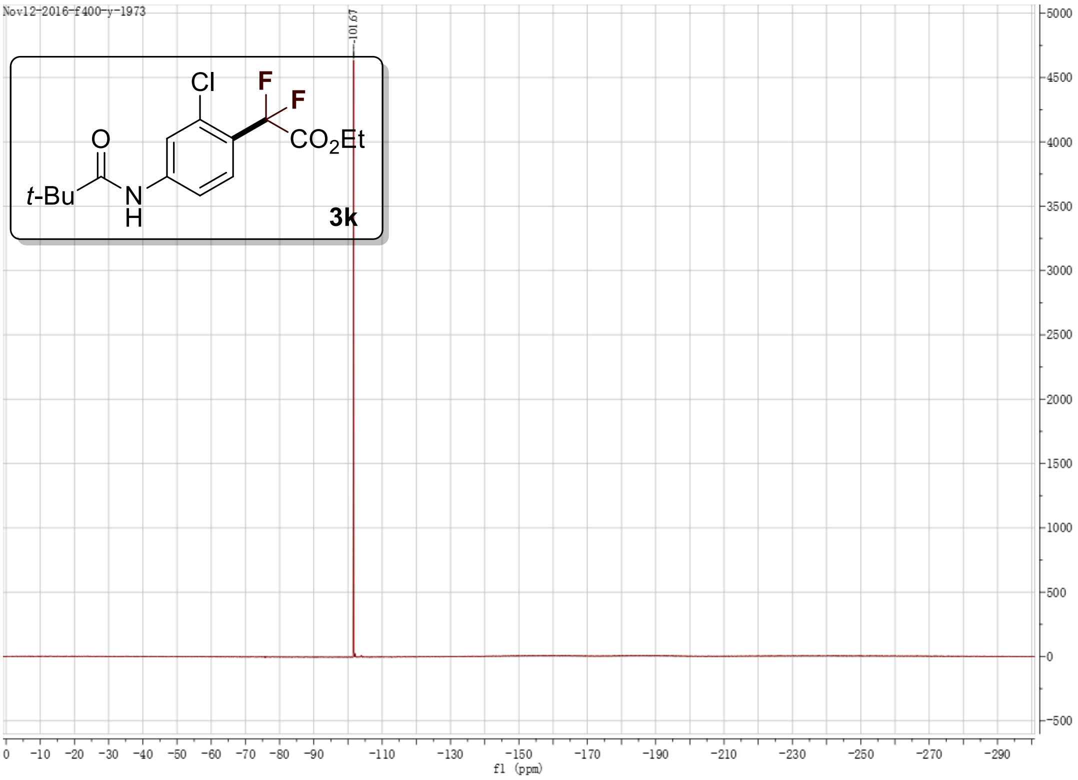

Supplementary Figure 86. ^{19}F NMR spectra for 3k

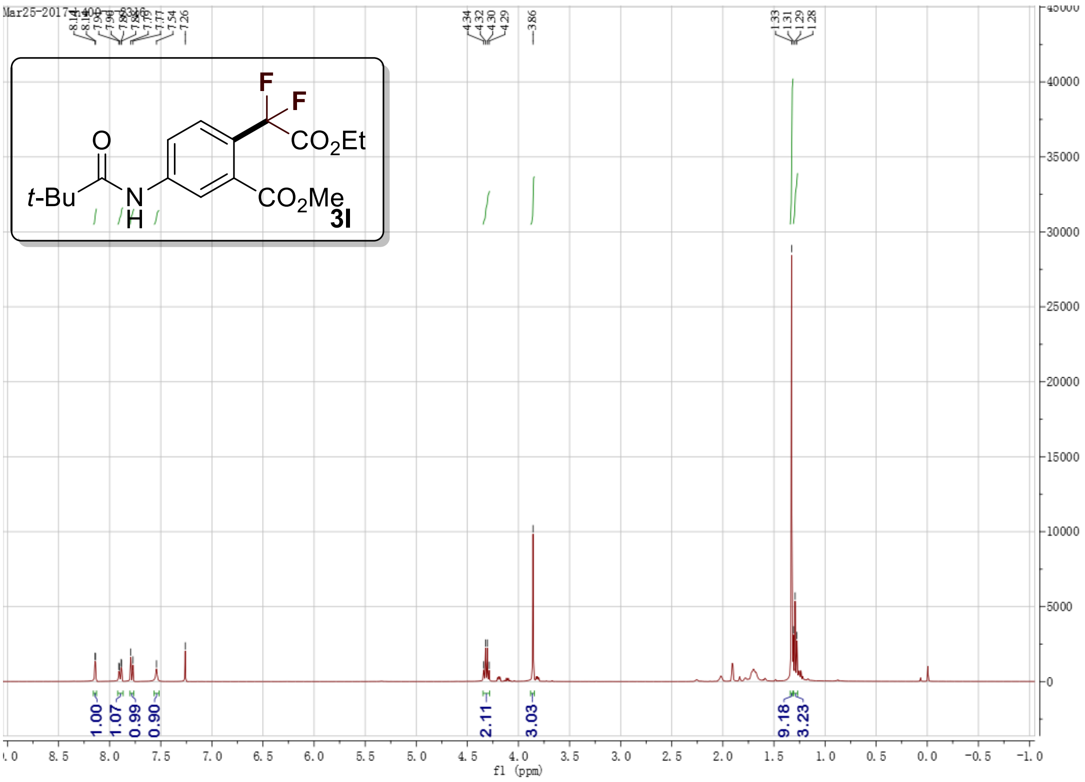

Supplementary Figure 87. ^{1}H NMR spectra for 3l

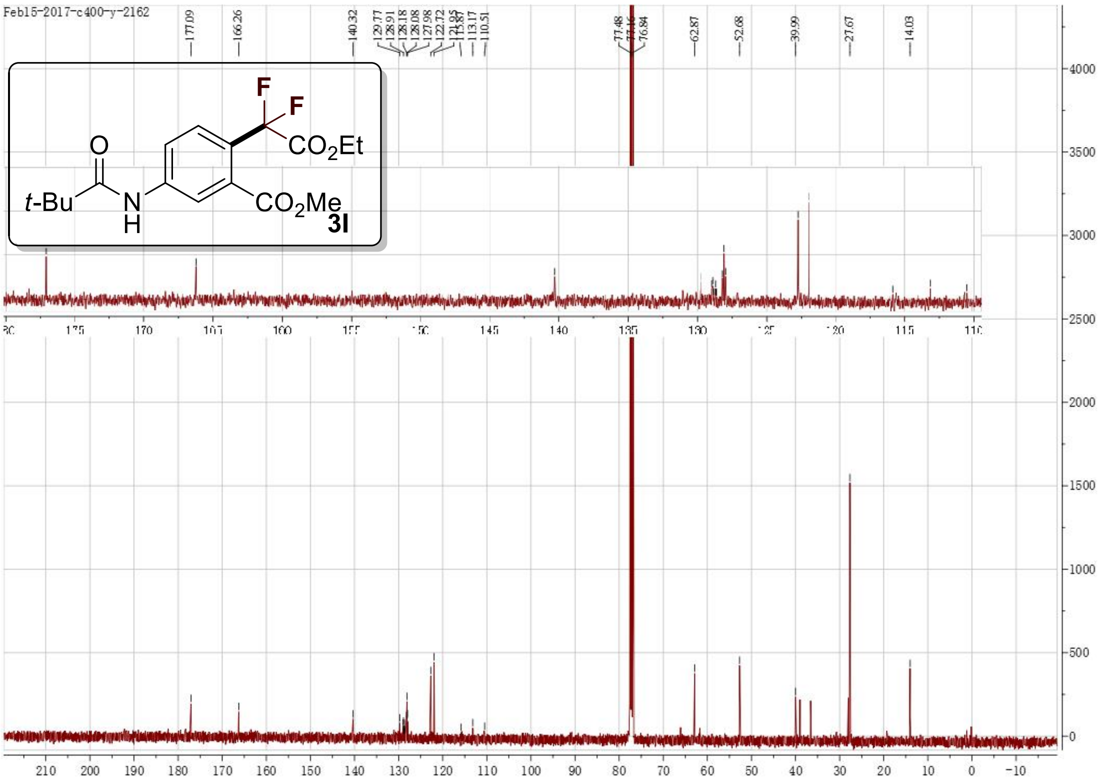

Supplementary Figure 88. ^{13}C NMR spectra for 3l

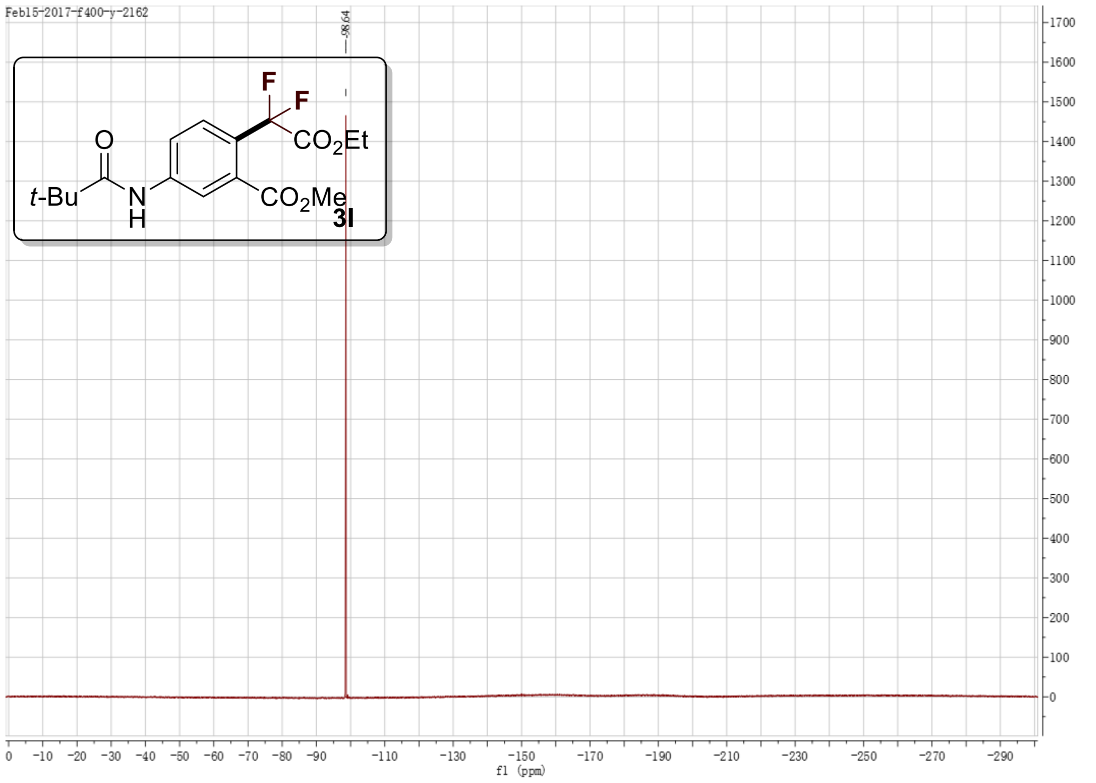

Supplementary Figure89. ^{19}F NMR spectra for 3l

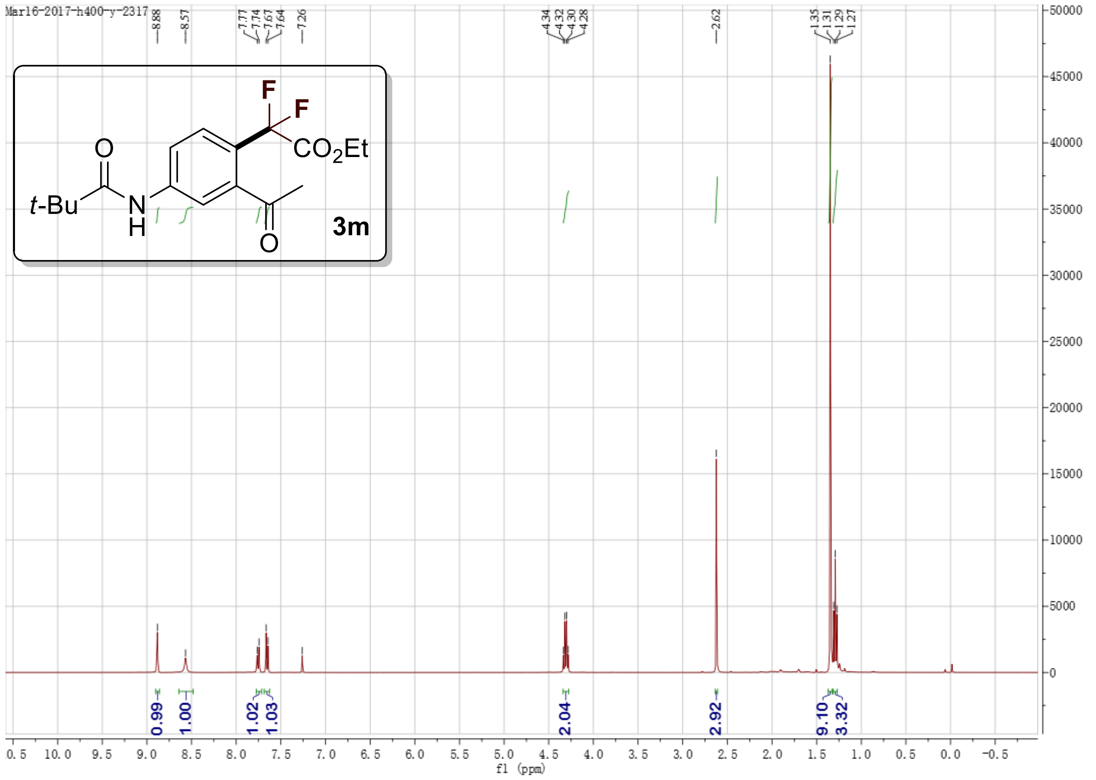

Supplementary Figure 90. ^{1}H NMR spectra for 3m

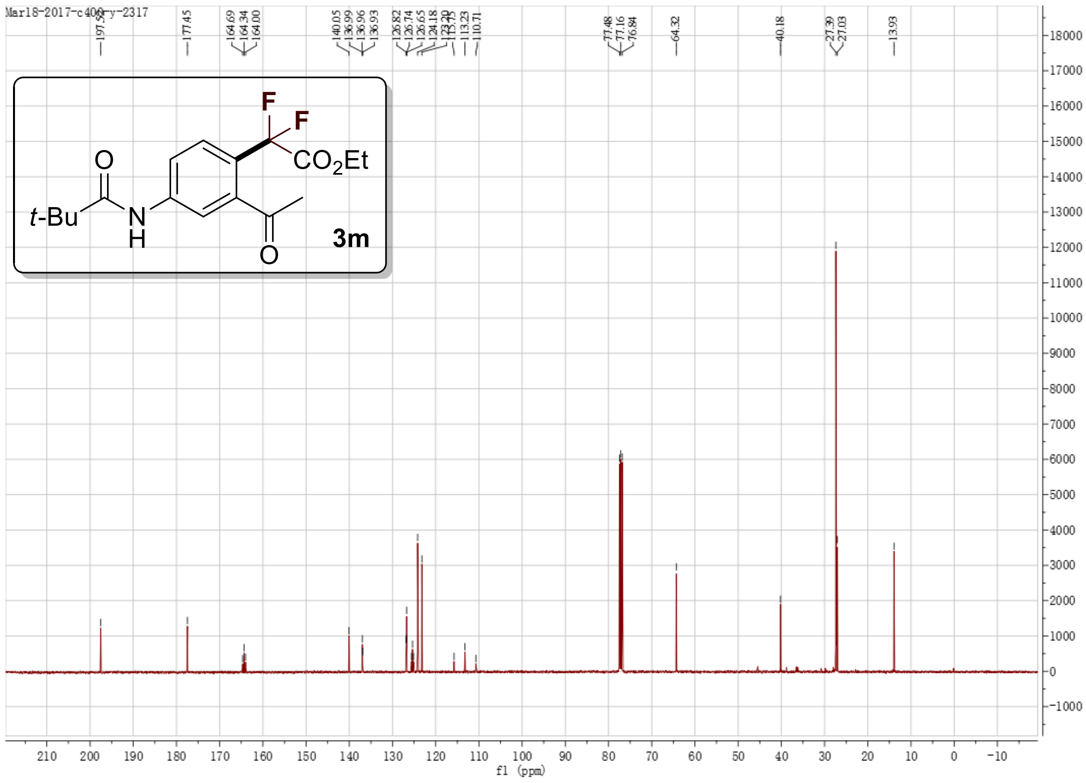

Supplementary Figure 91. ^{13}**C NMR spectra for 3m**

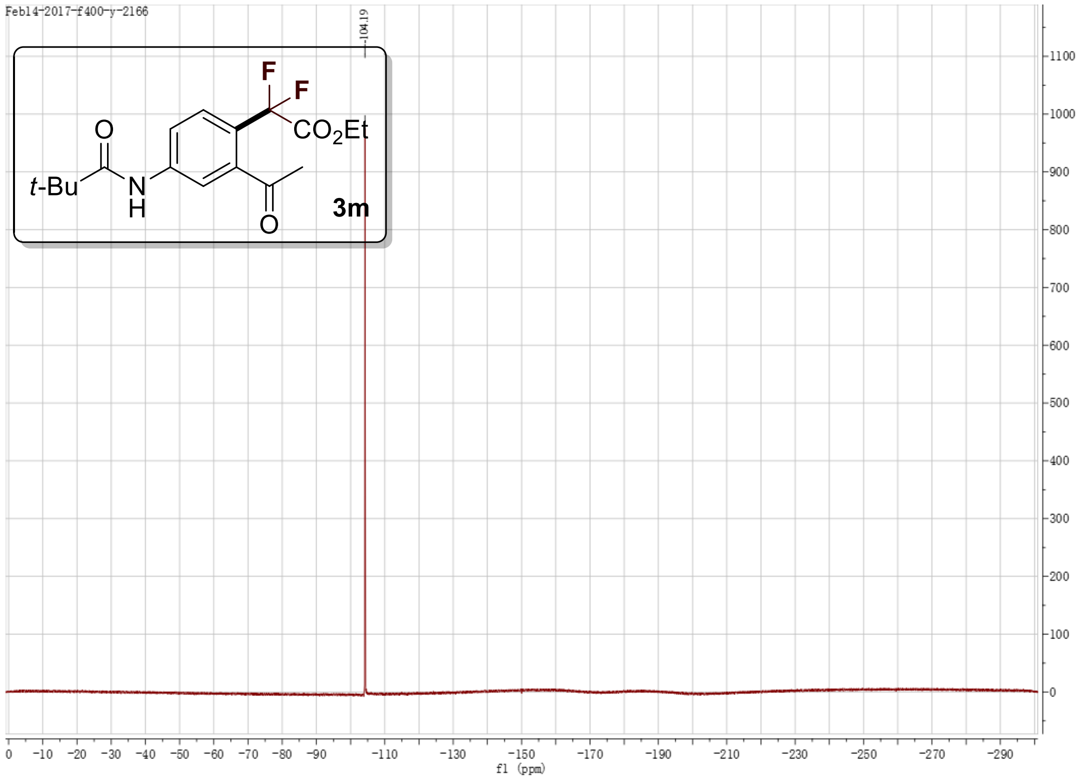

Supplementary Figure 92. ^{19}**F NMR spectra for 3m**

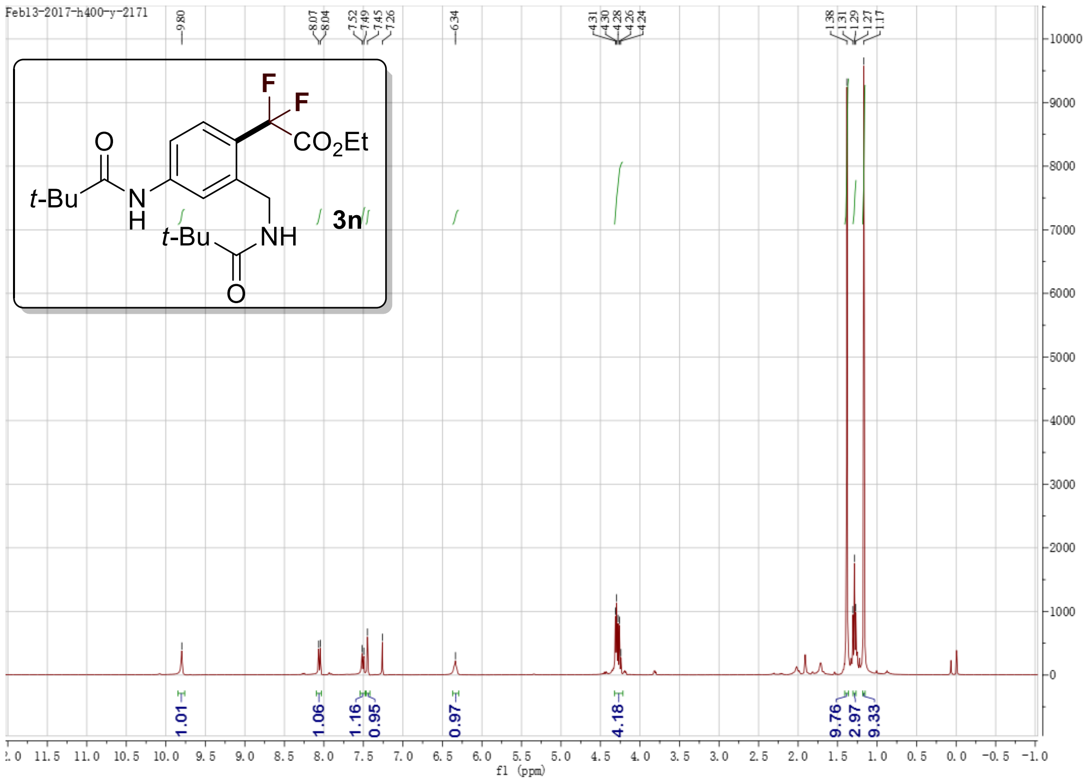

Supplementary Figure 93. ^{1}H NMR spectra for 3n

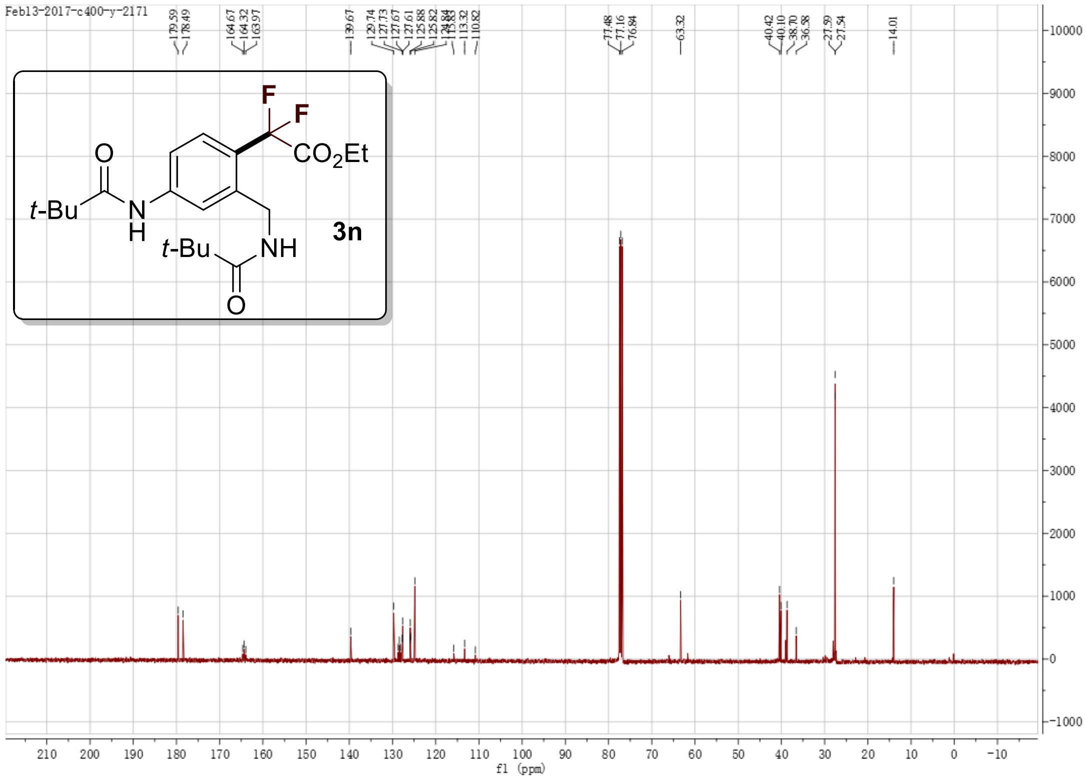

Supplementary Figure 94. ^{13}C NMR spectra for 3n

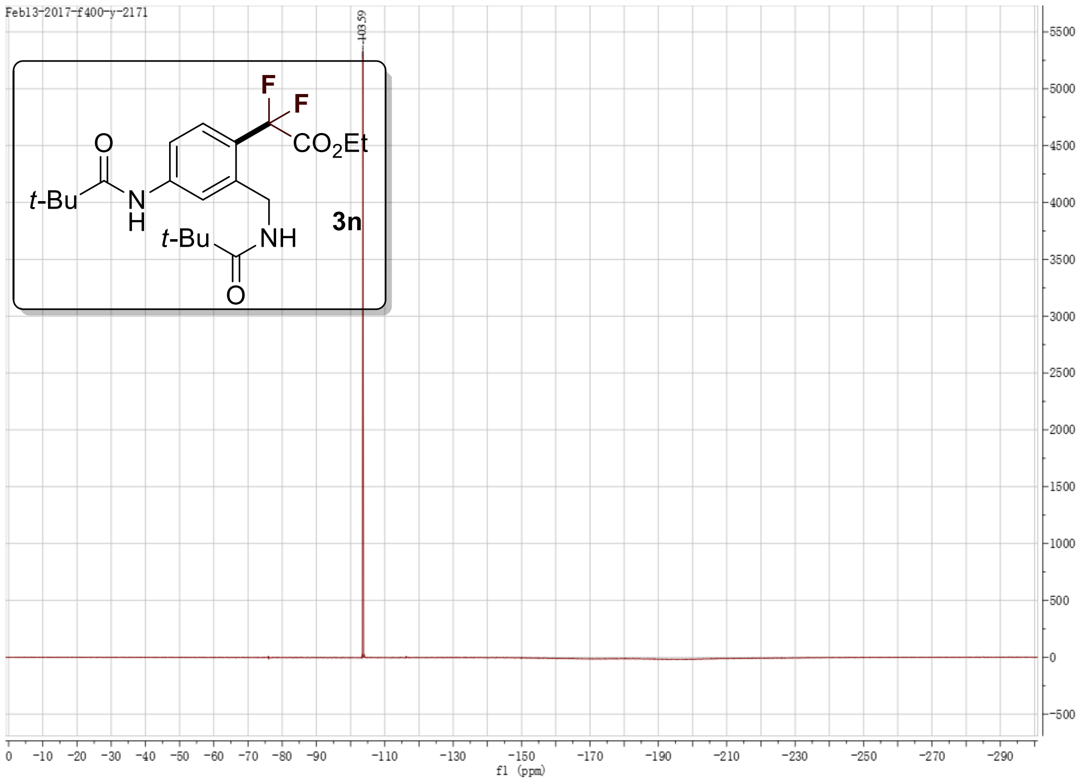

Supplementary Figure 95. ^{19}F NMR spectra for 3n

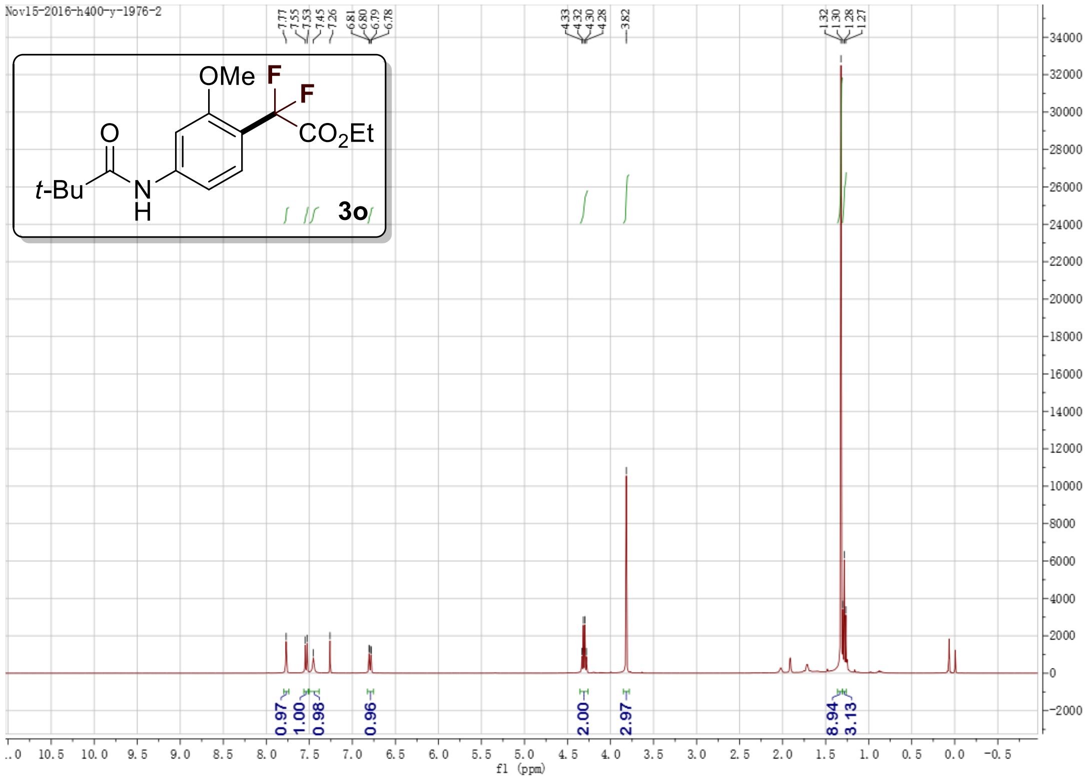

Supplementary Figure 96. ^{1}H NMR spectra for 3o

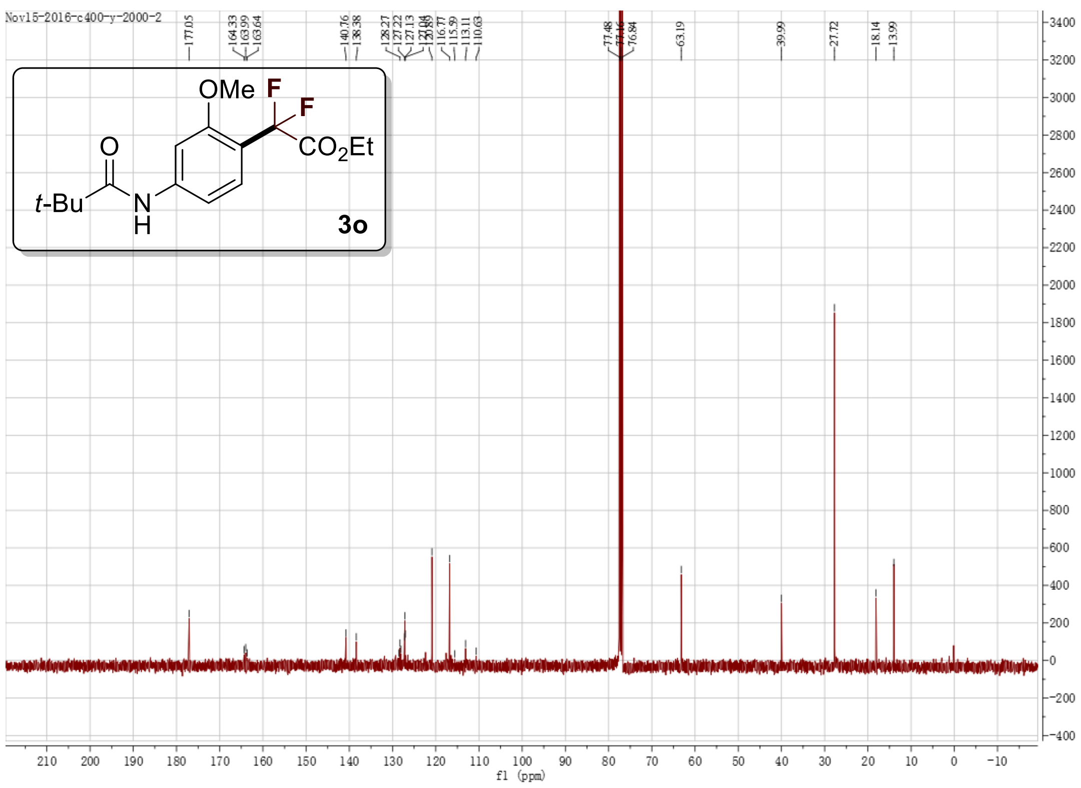

Supplementary Figure 97. ^{13}C NMR spectra for 3o

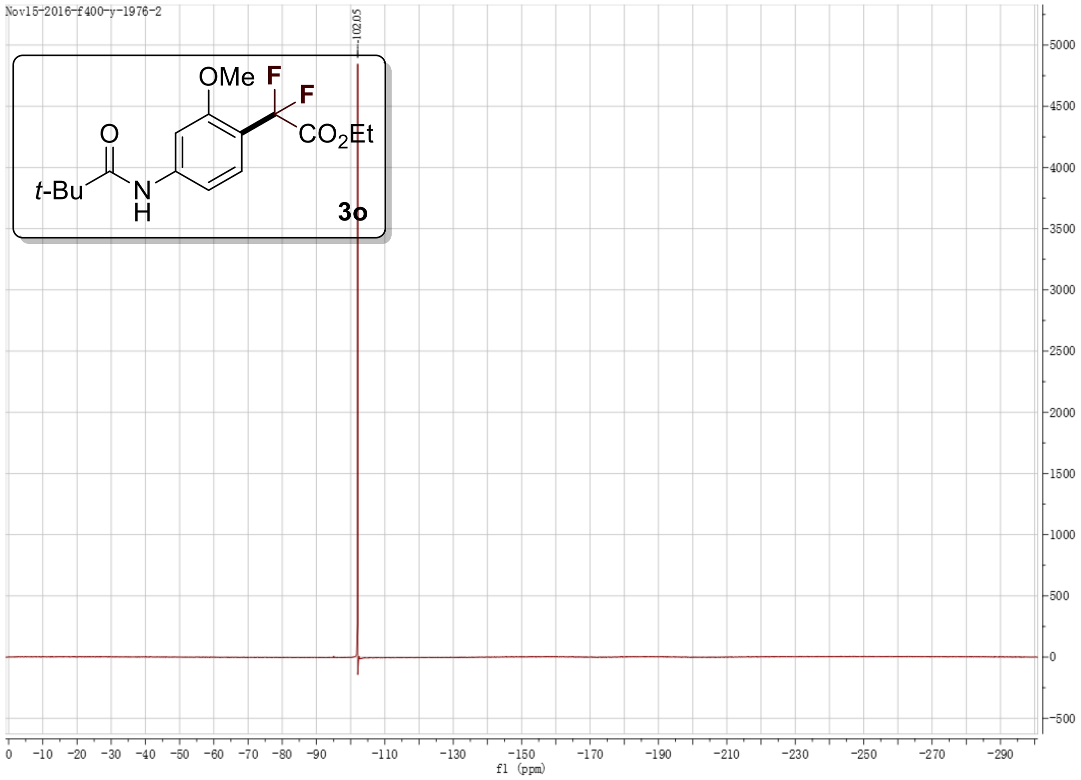

Supplementary Figure 98. ^{19}F NMR spectra for 3o

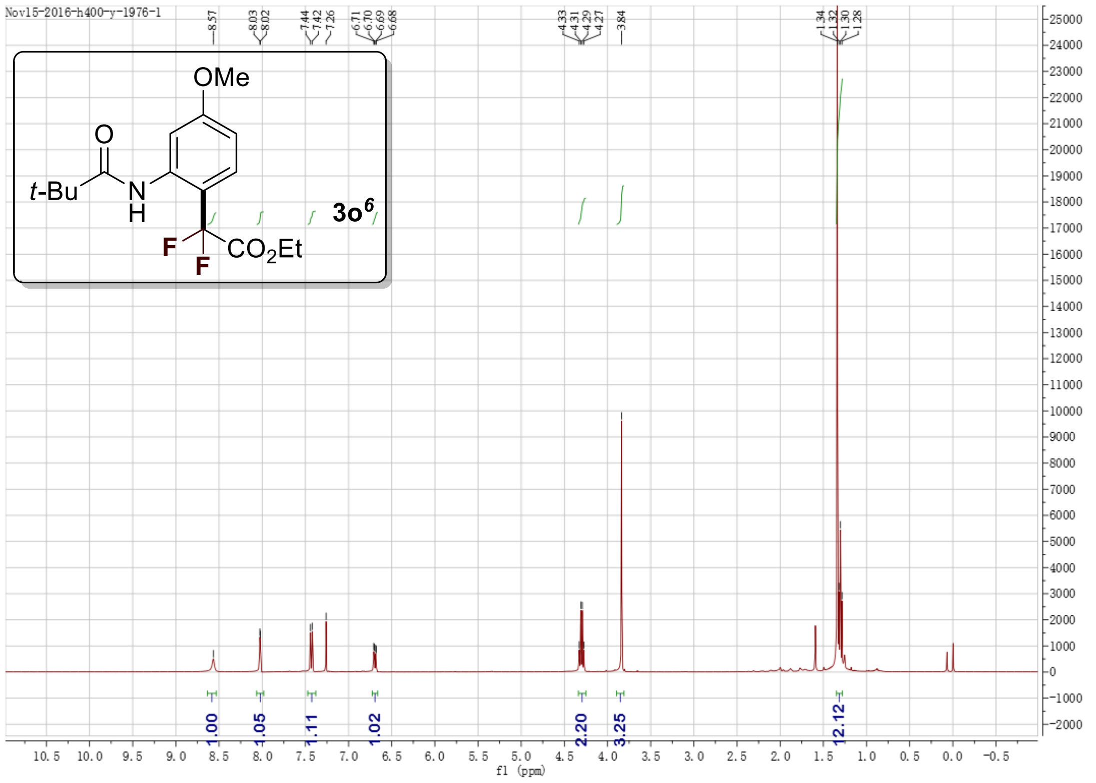

Supplementary Figure 99. **^{1}H NMR spectra for 3o**$^{(6)}$

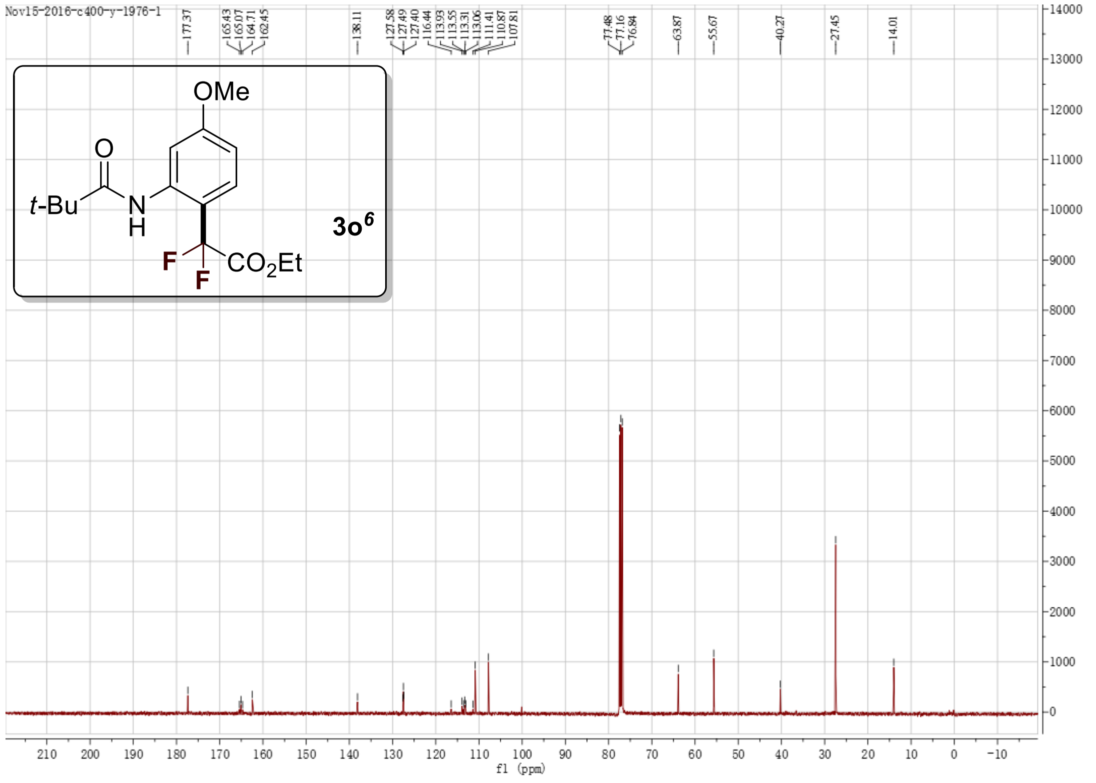

Supplementary Figure 100. **^{13}C NMR spectra for 3o**$^{(6)}$

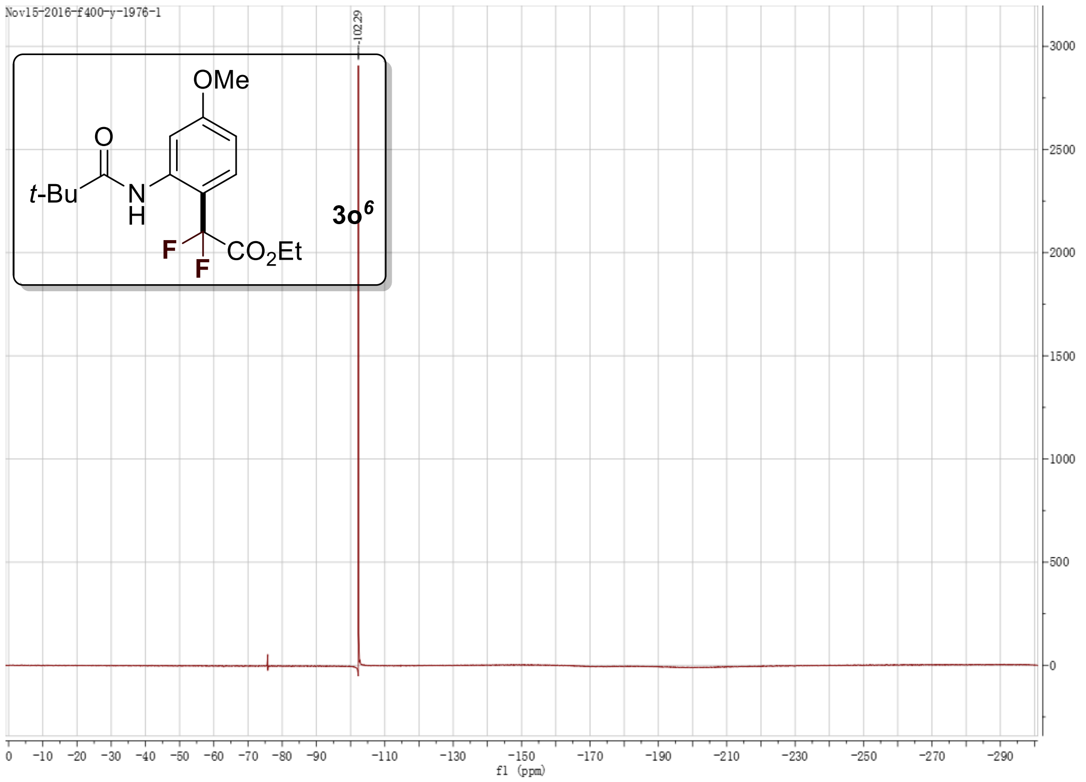

Supplementary Figure 101. **^{19}F NMR spectra for 3o[(6)]**

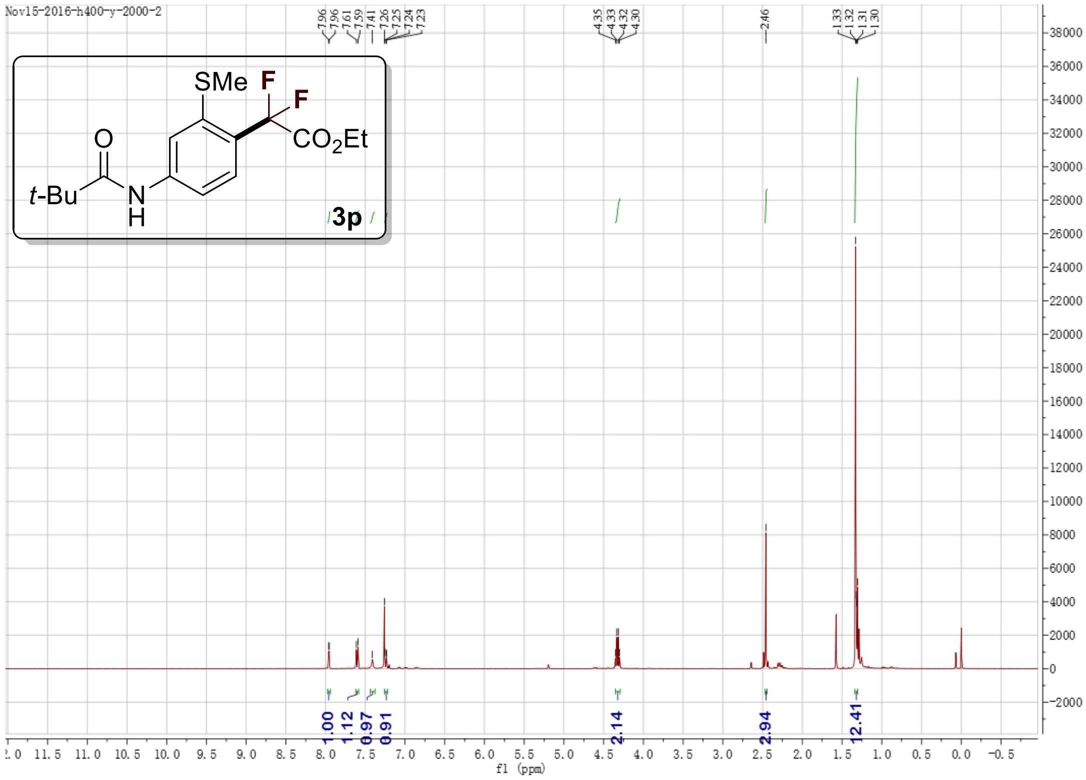

Supplementary Figure 102. **^{1}H NMR spectra for 3p**

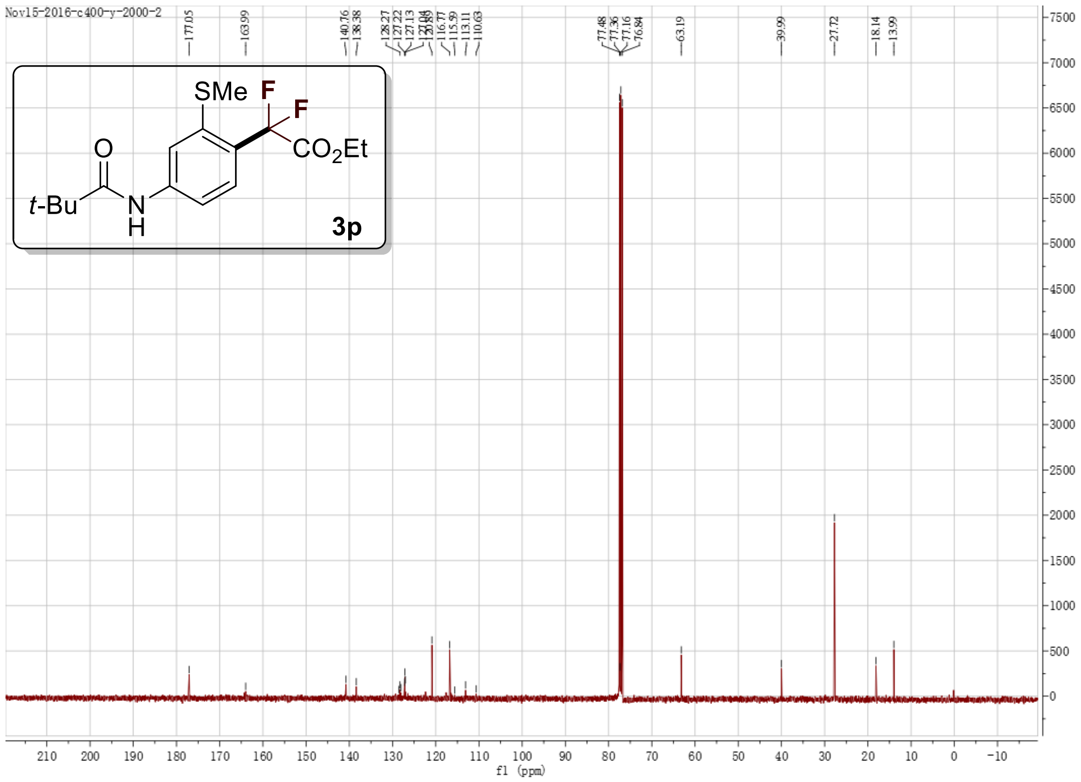

Supplementary Figure 103. ^{13}**C NMR spectra for 3p**

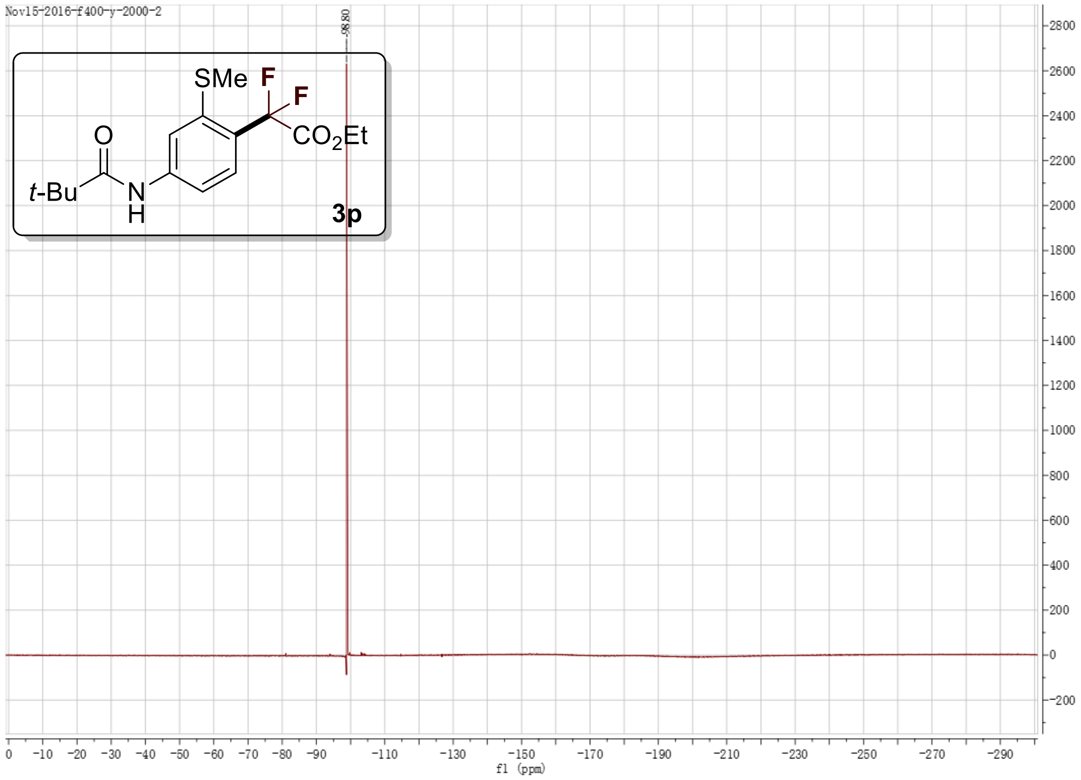

Supplementary Figure 104. ^{19}**F NMR spectra for 3p**

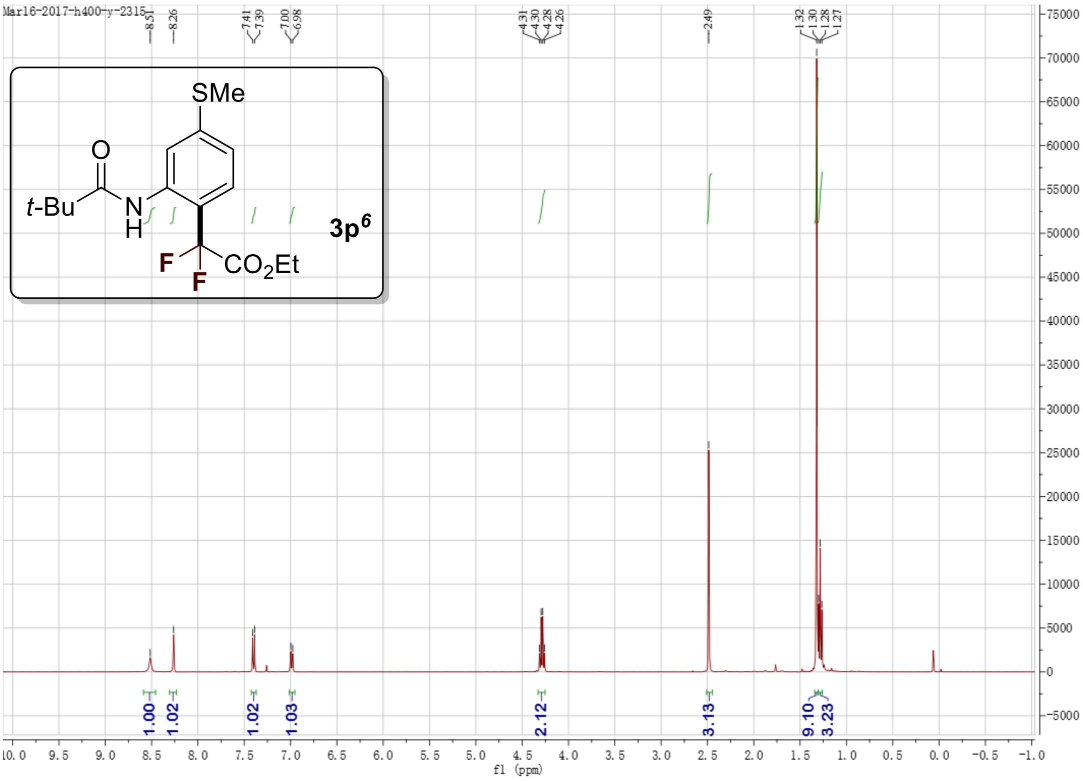

Supplementary Figure 105. ^{1}**H NMR spectra for 3p**$^{(6)}$

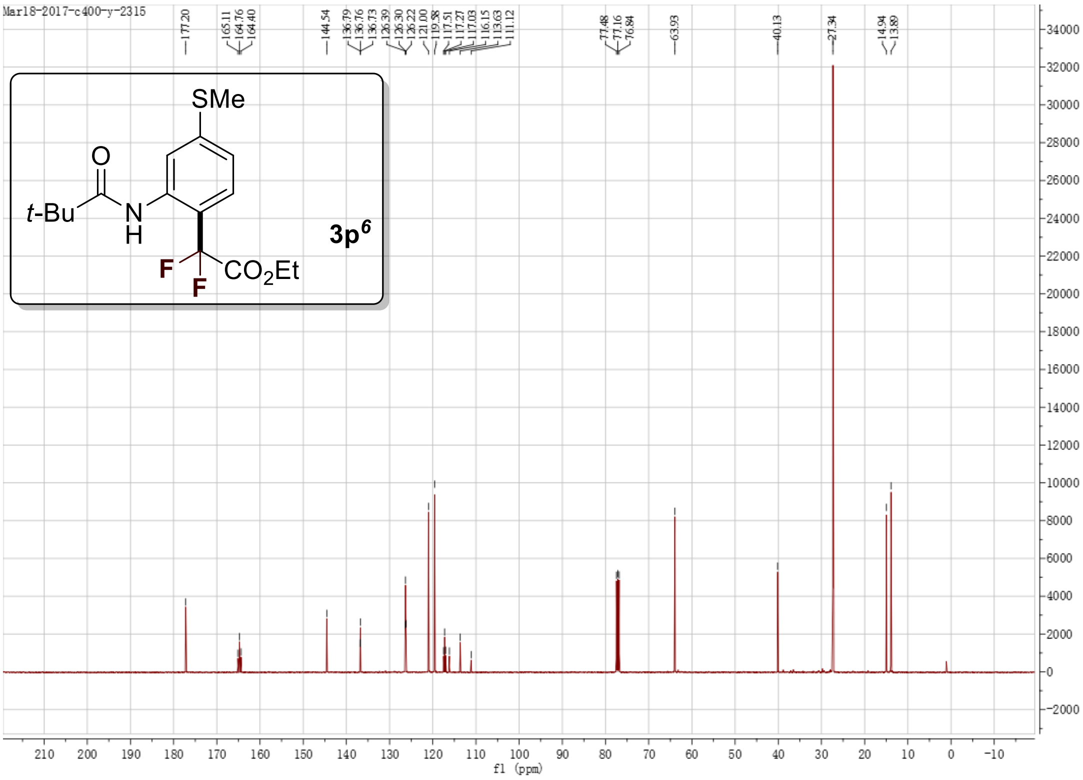

Supplementary Figure 106. ^{13}**C NMR spectra for 3p**$^{(6)}$

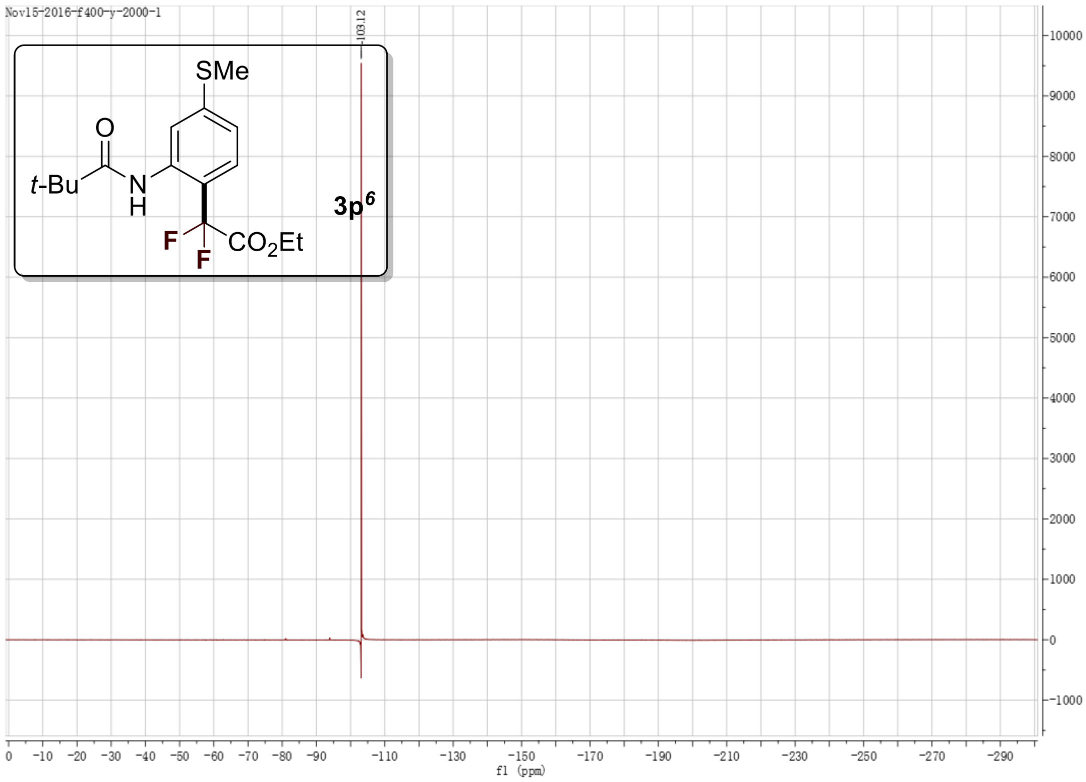

Supplementary Figure 107. ^{19}**F NMR spectra for 3p**$^{(6)}$

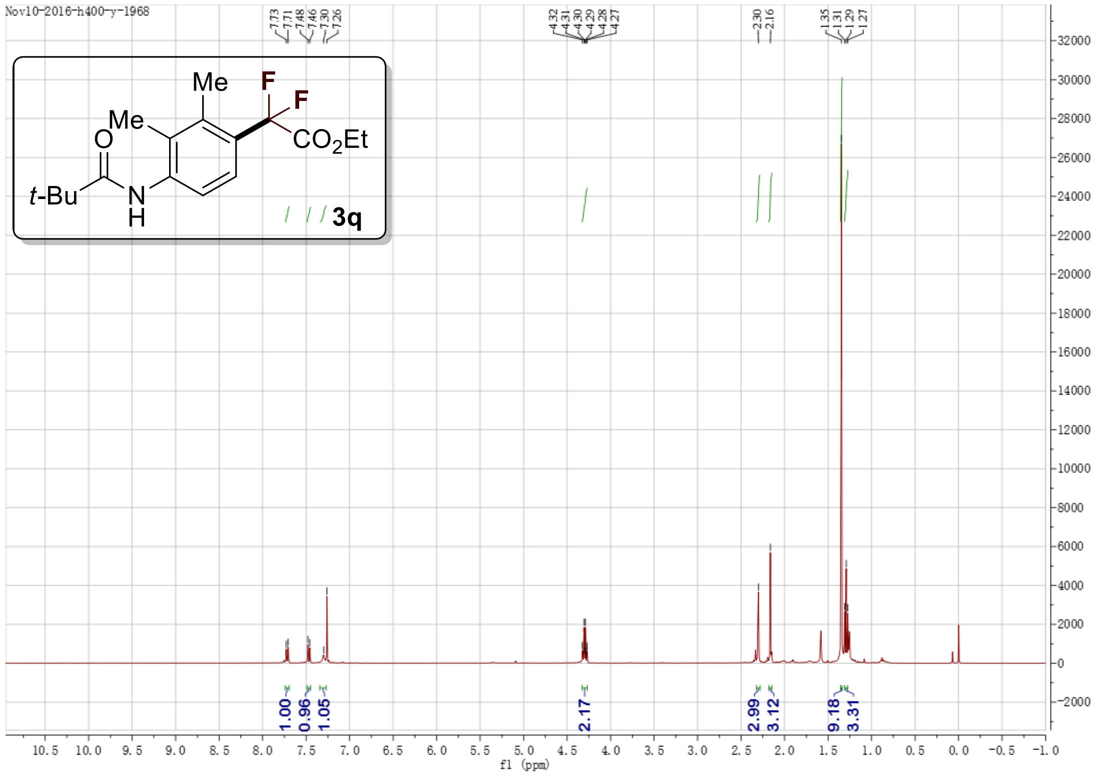

Supplementary Figure 108. ^{1}**H NMR spectra for 3q**

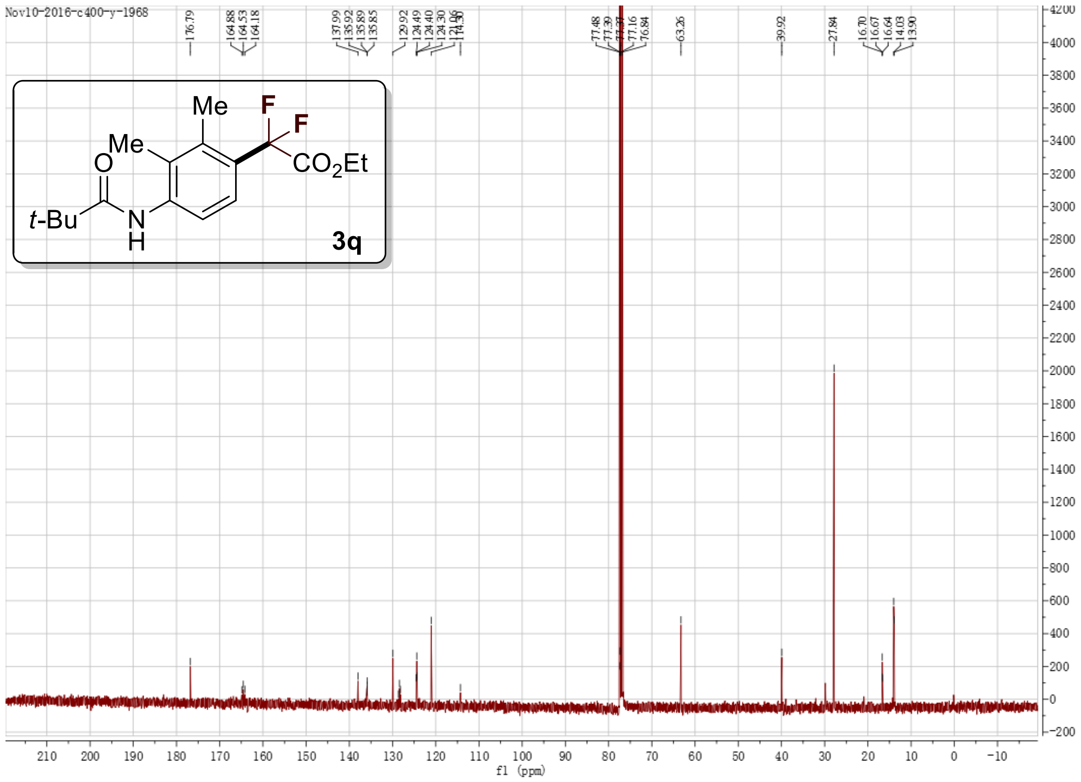

Supplementary Figure 109. ^{13}**C NMR spectra for 3q**

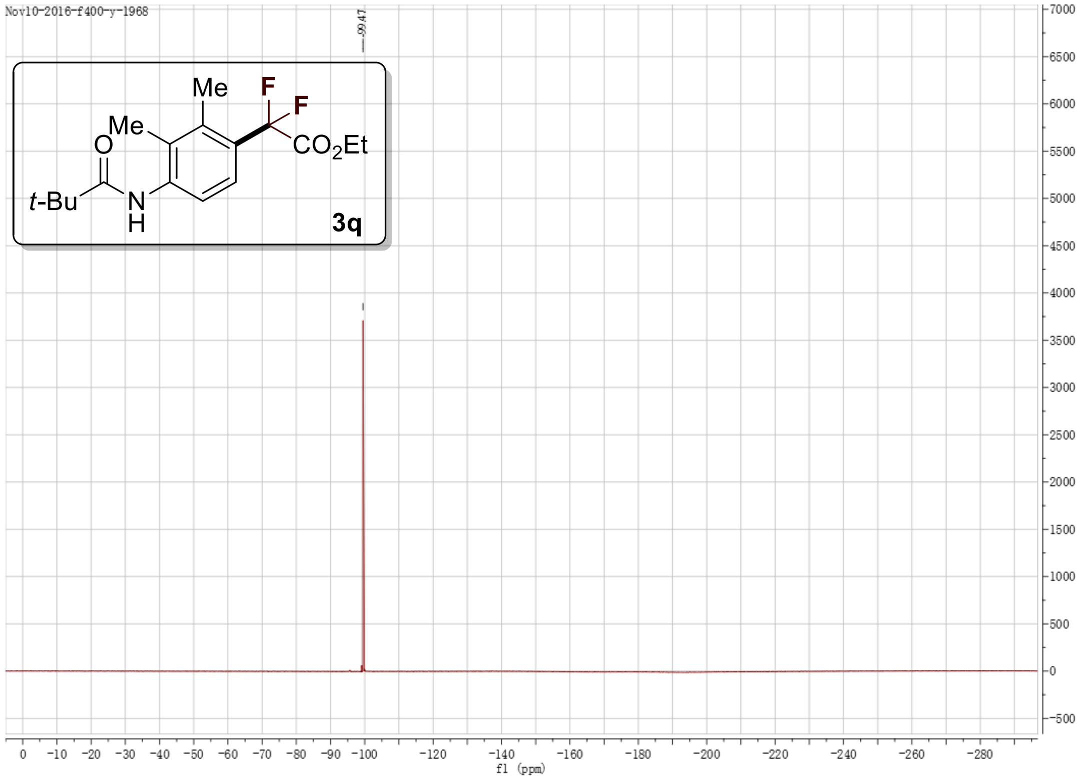

Supplementary Figure 110. ^{19}**F NMR spectra for 3q**

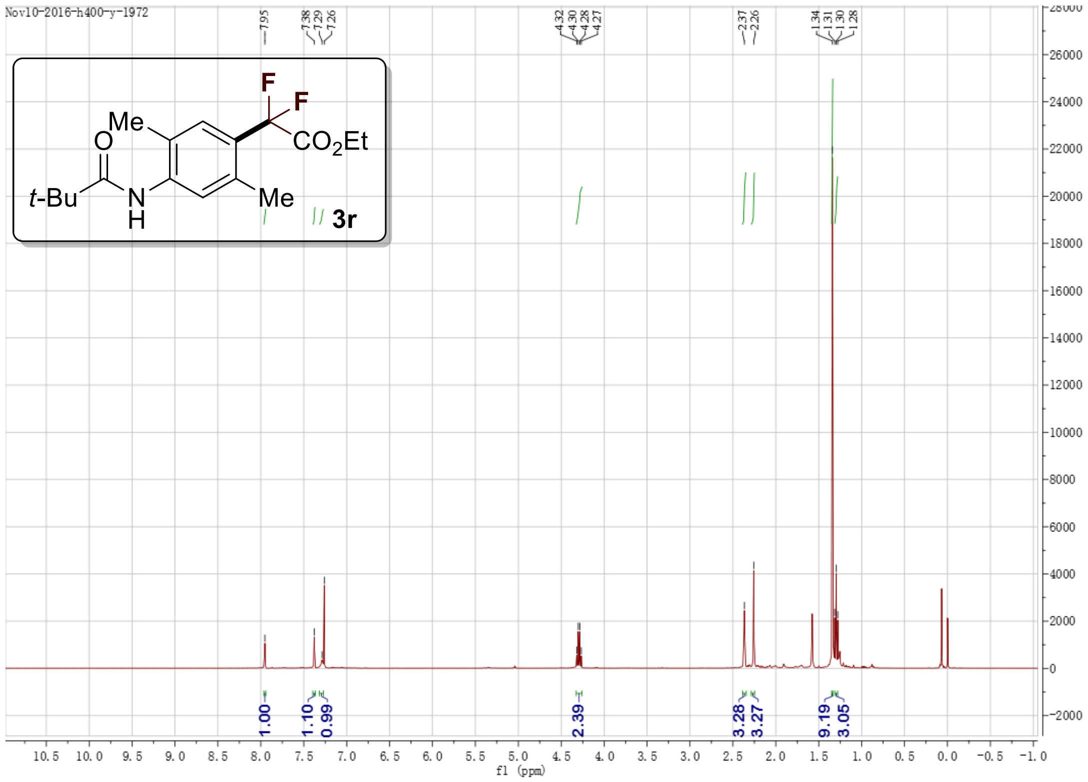

Supplementary Figure 111. ^{1}**H NMR spectra for 3r**

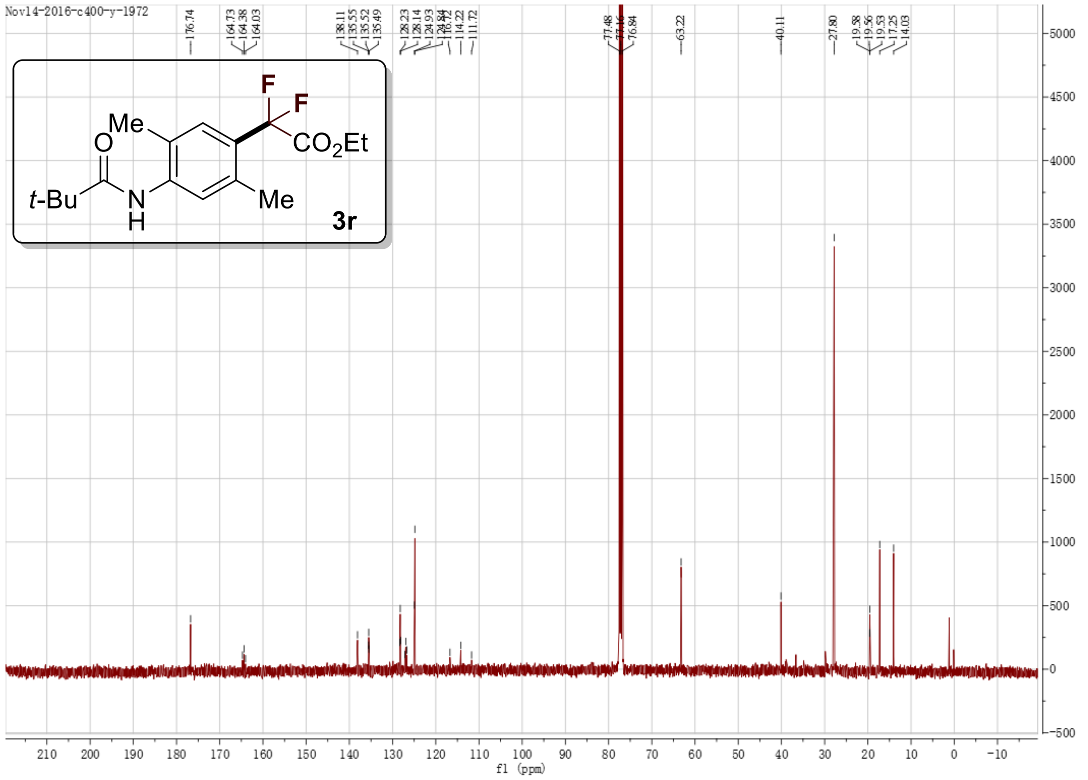

Supplementary Figure 112. ^{13}**C NMR spectra for 3r**

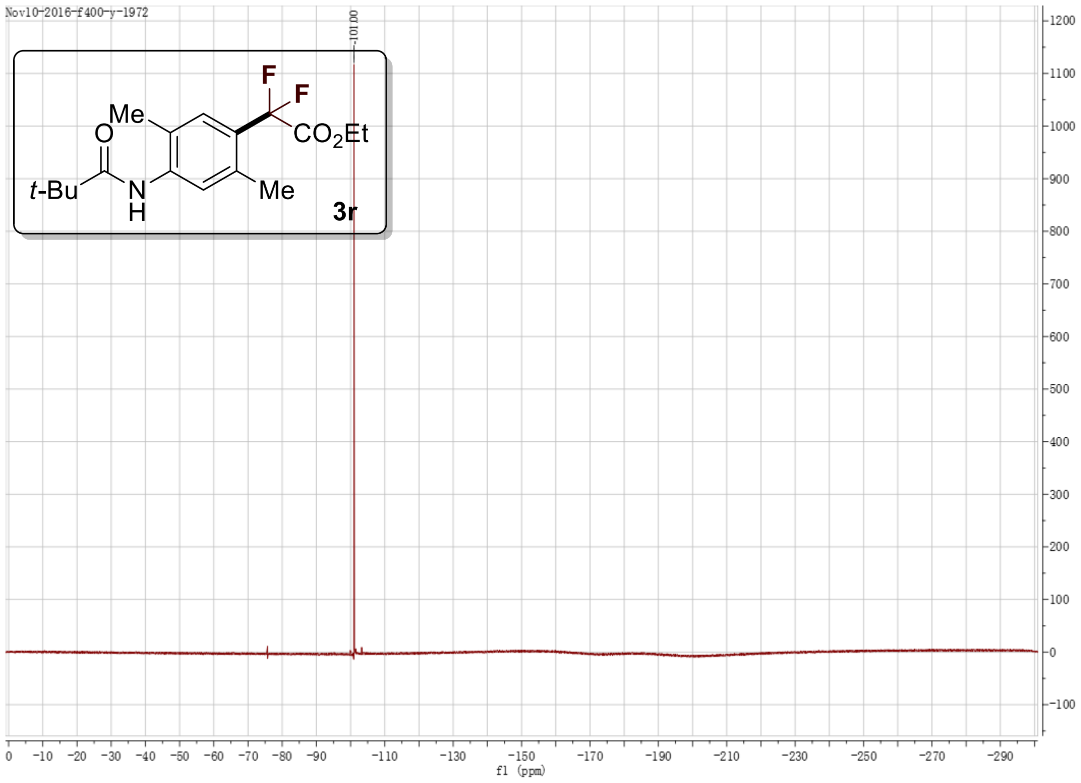

Supplementary Figure 113. ^{19}F NMR spectra for 3r

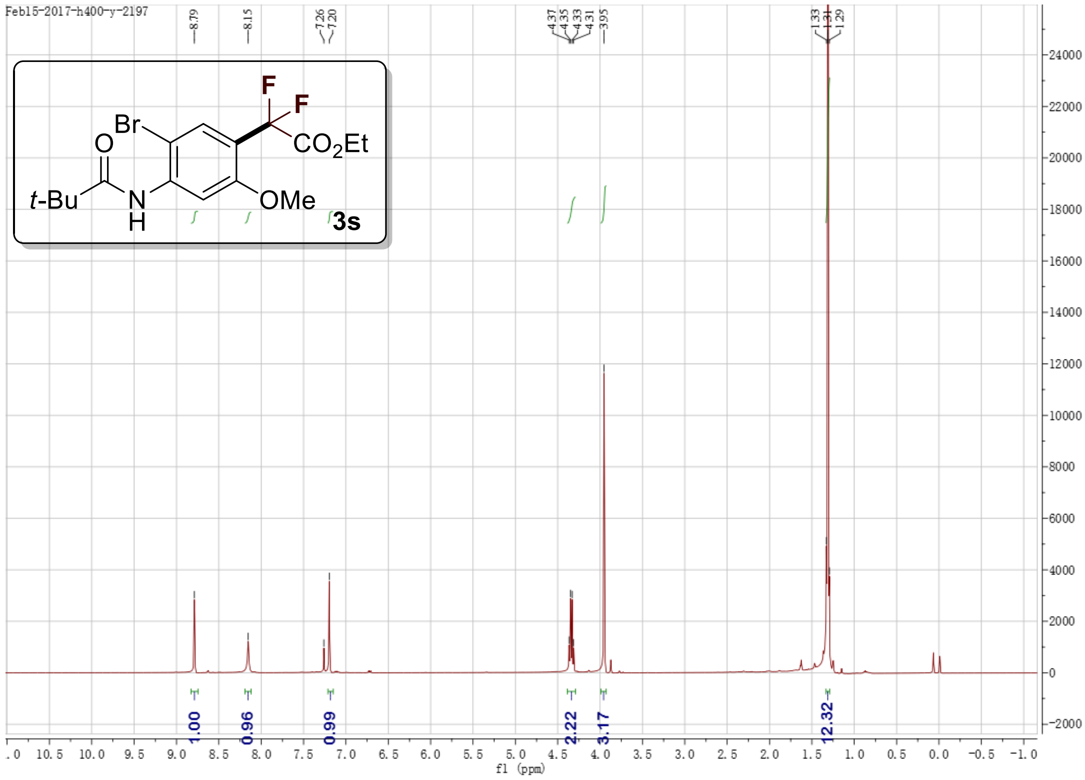

Supplementary Figure 114. ^{1}H NMR spectra for 3s

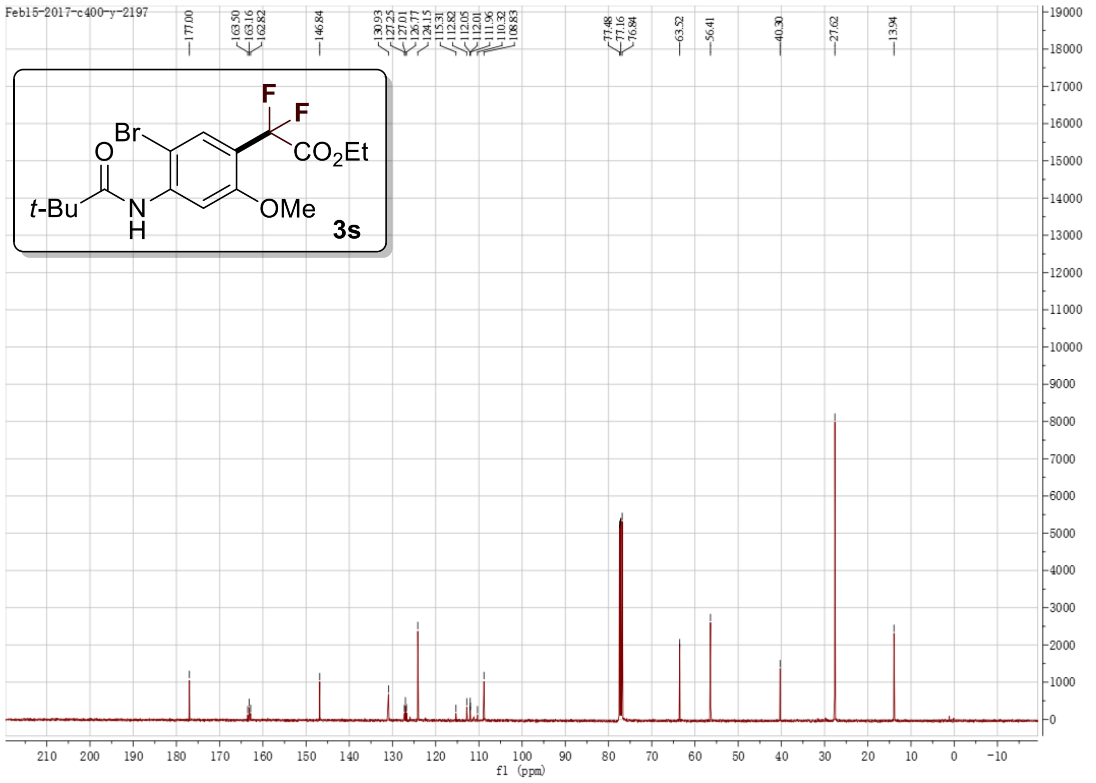

Supplementary Figure 115. ^{13}C NMR spectra for 3s

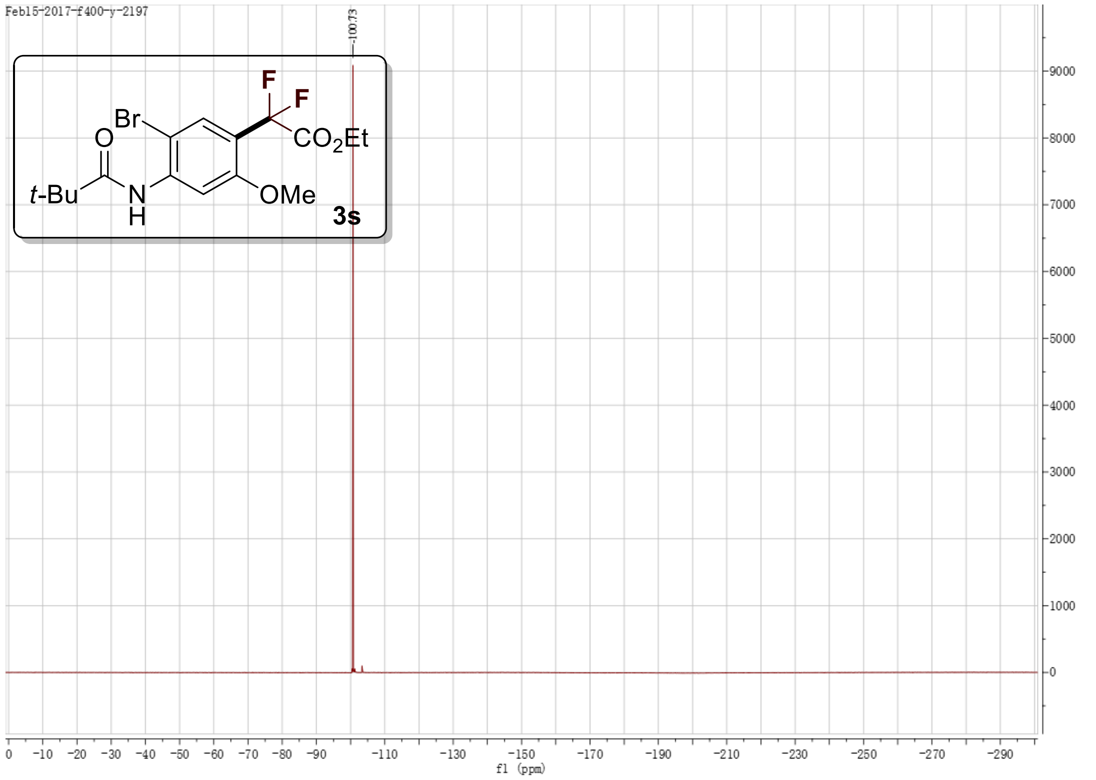

Supplementary Figure 116. ^{19}F NMR spectra for 3s

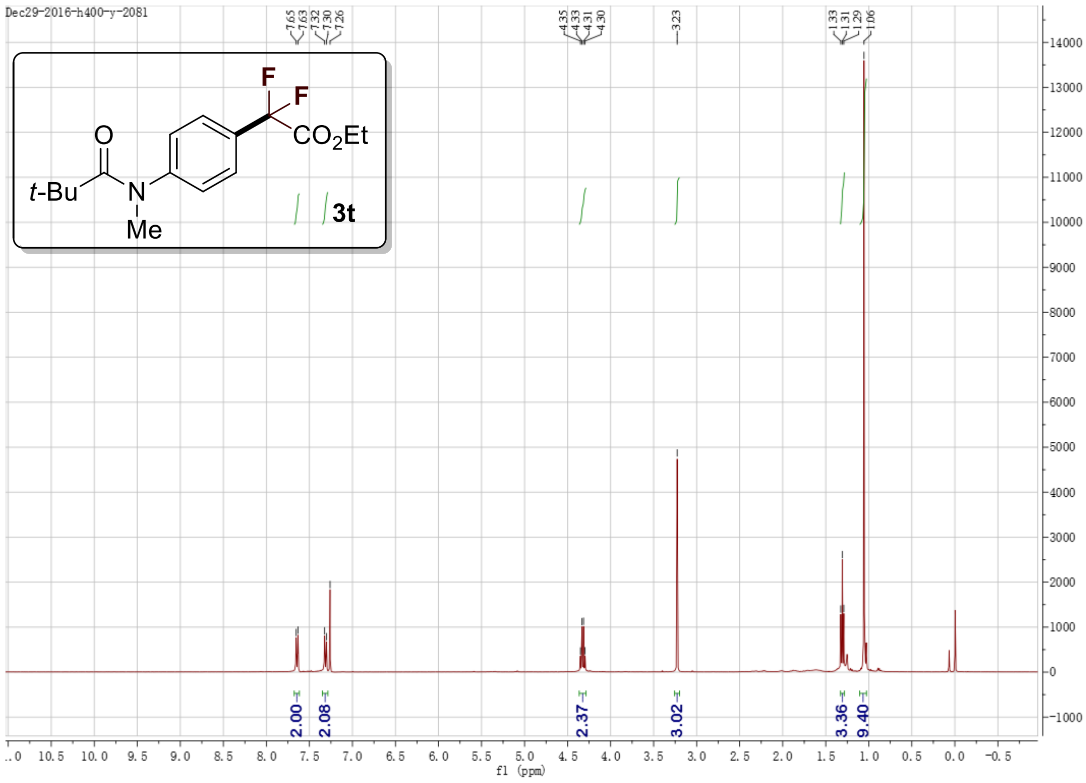

Supplementary Figure 117. ^{1}**H NMR spectra for 3t**

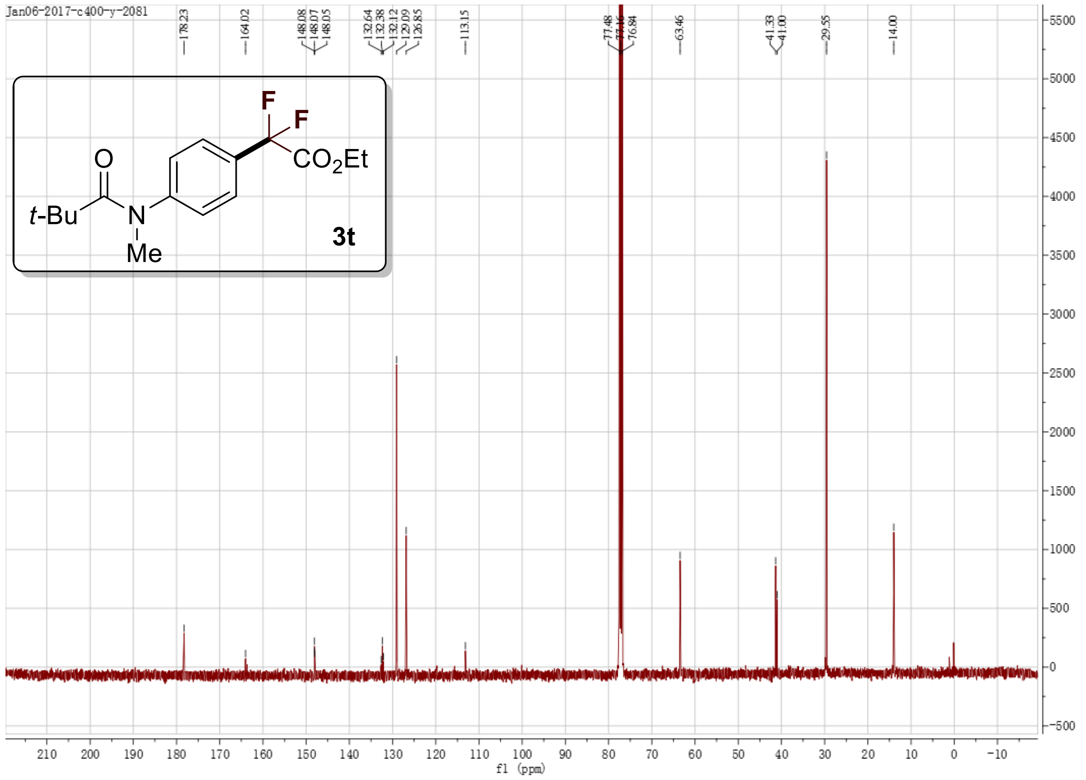

Supplementary Figure 118. ^{13}**C NMR spectra for 3t**

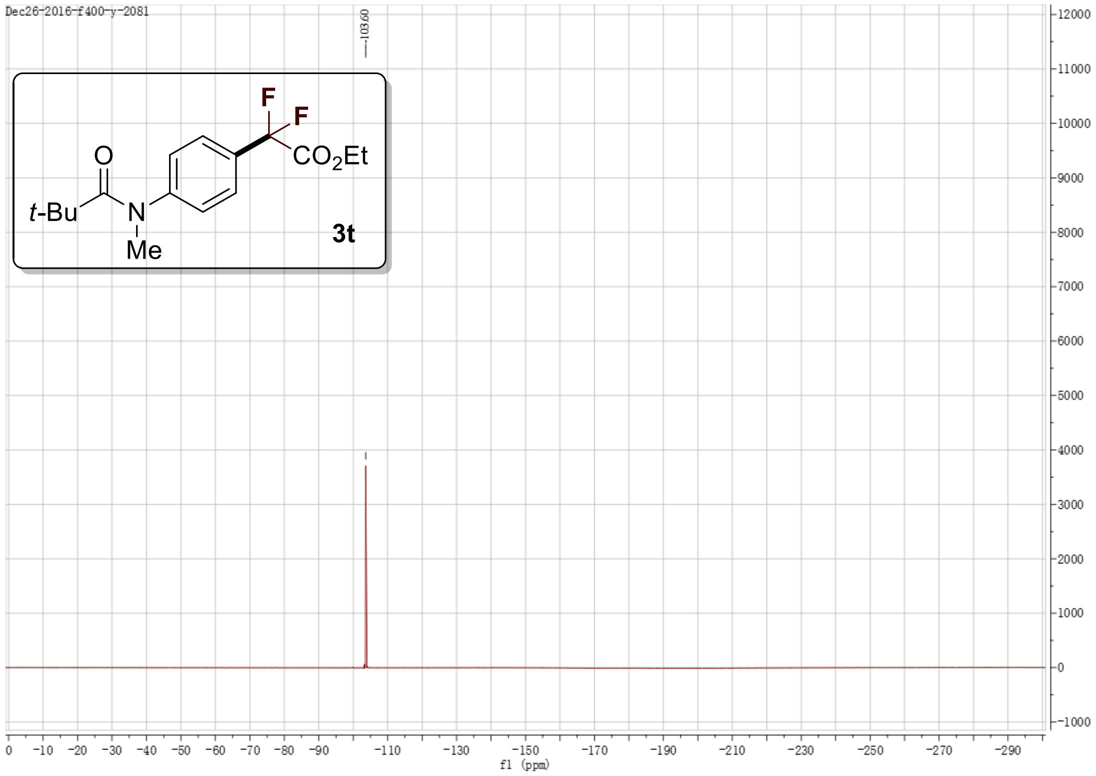

Supplementary Figure 119. ^{19}F NMR spectra for 3t

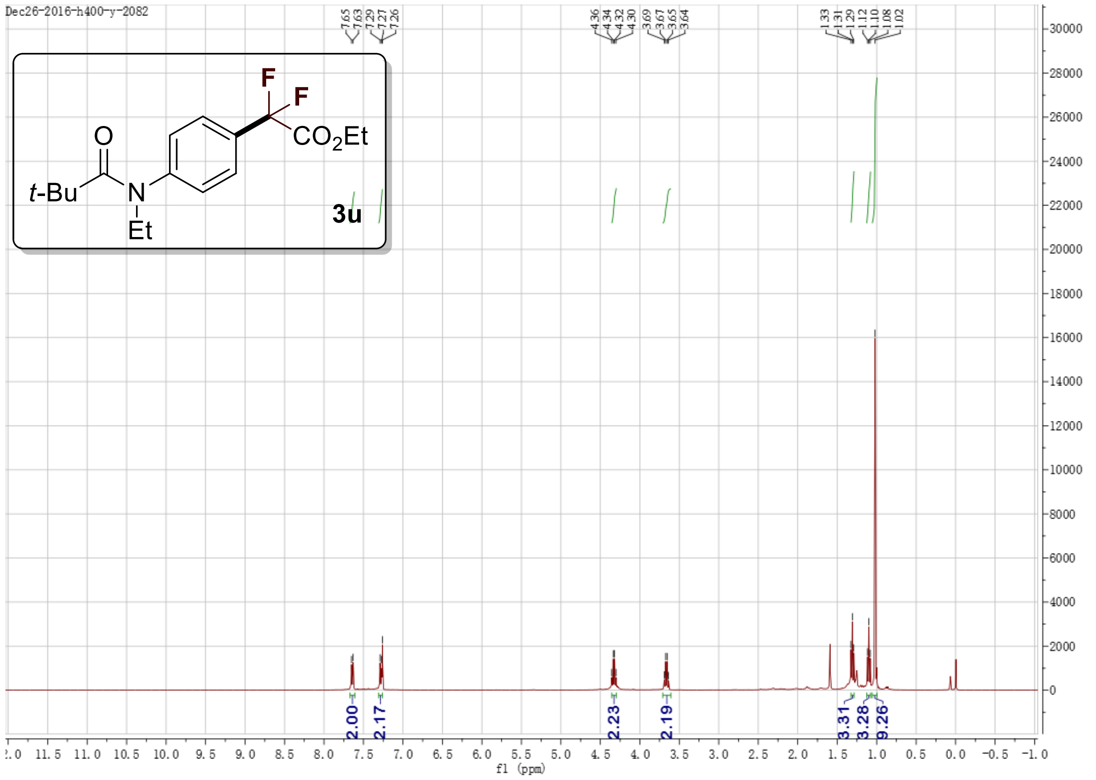

Supplementary Figure 120. ^{1}H NMR spectra for 3u

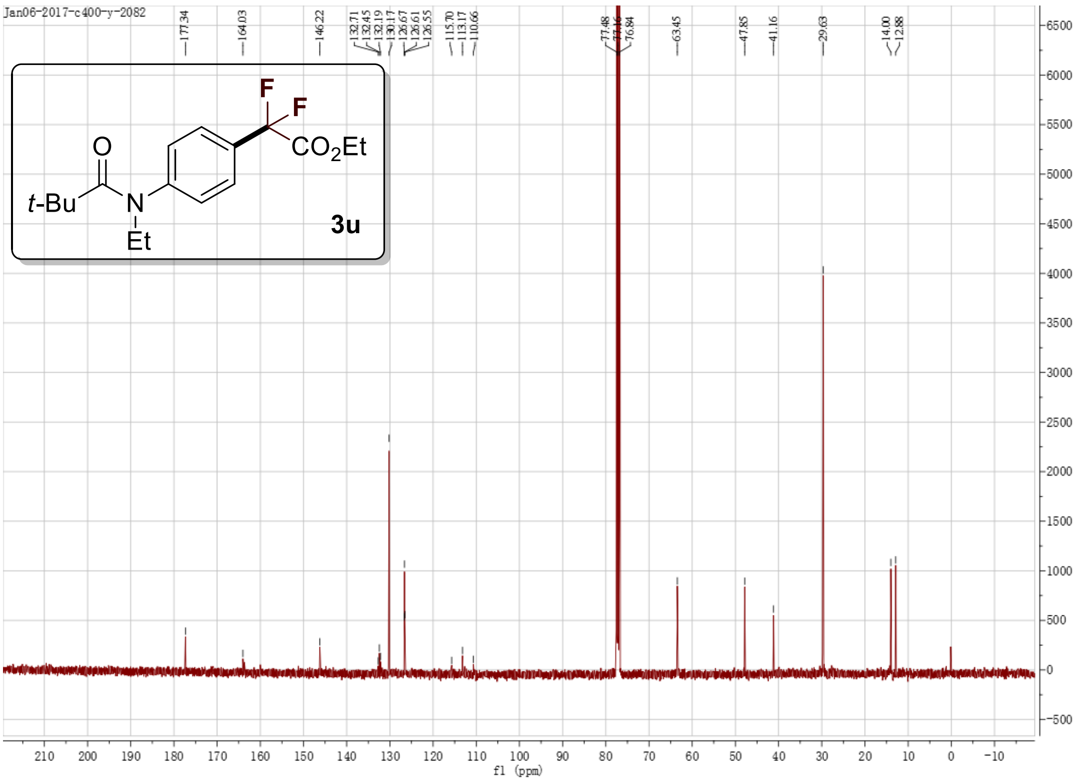

Supplementary Figure 121. ^{13}**C NMR spectra for 3u**

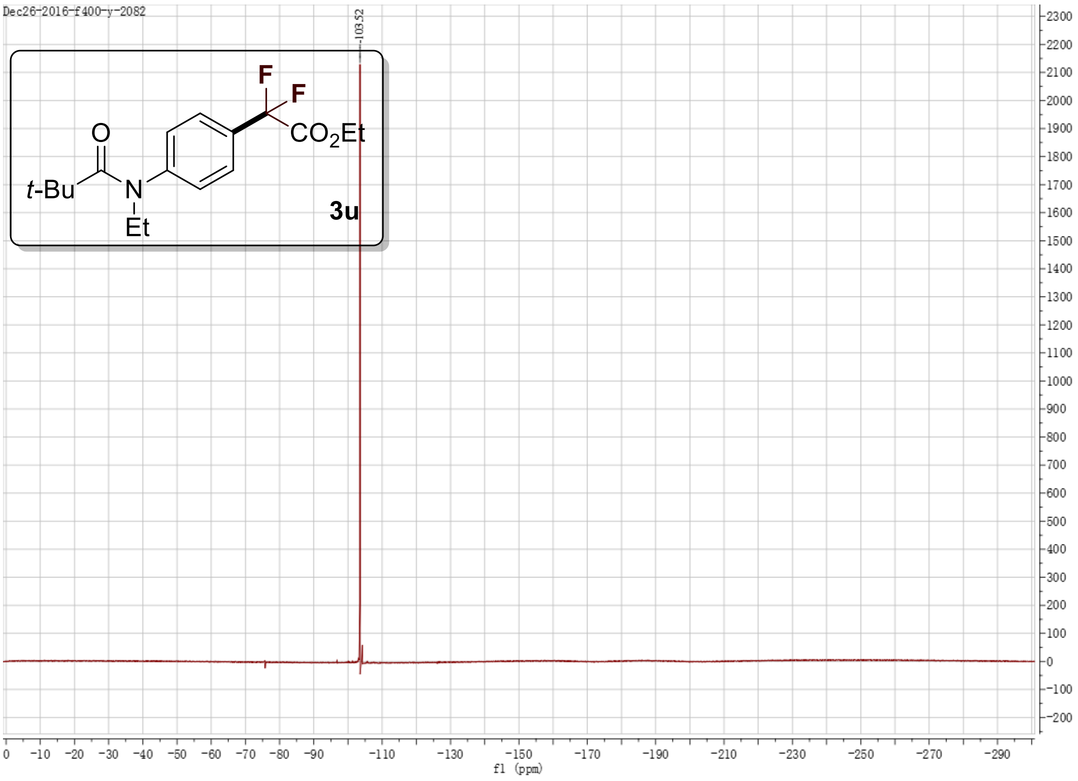

Supplementary Figure 122. ^{19}**F NMR spectra for 3u**

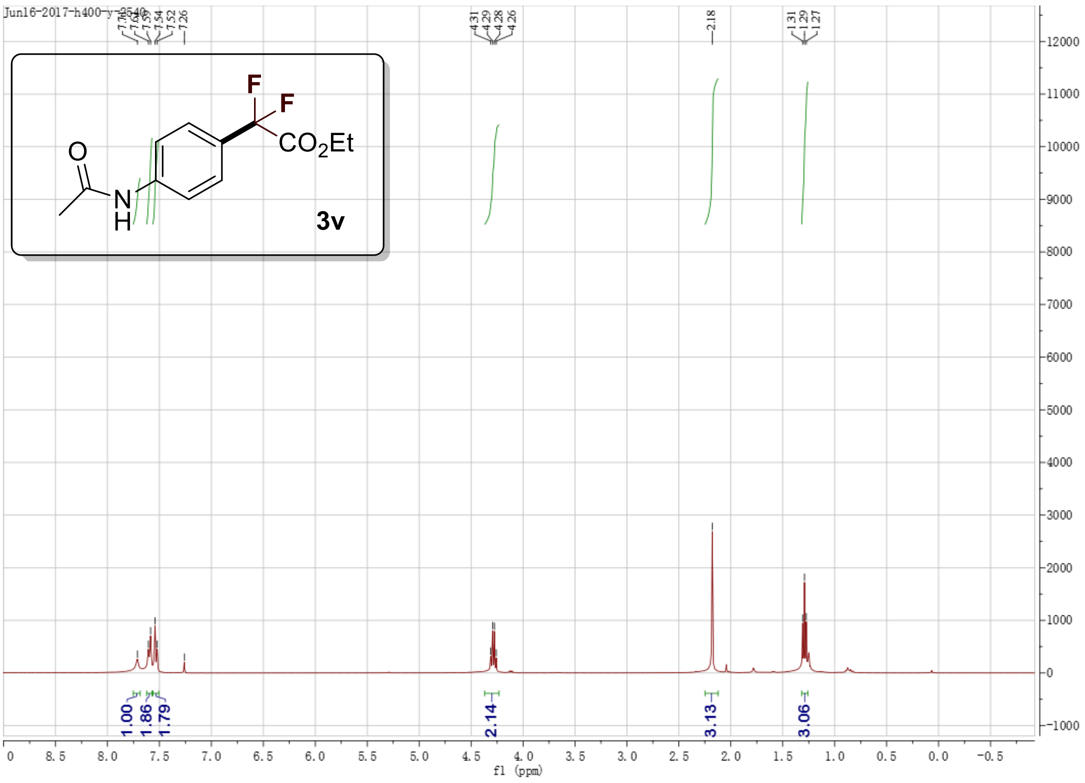

Supplementary Figure 123. ^{1}**H NMR spectra for 3v**

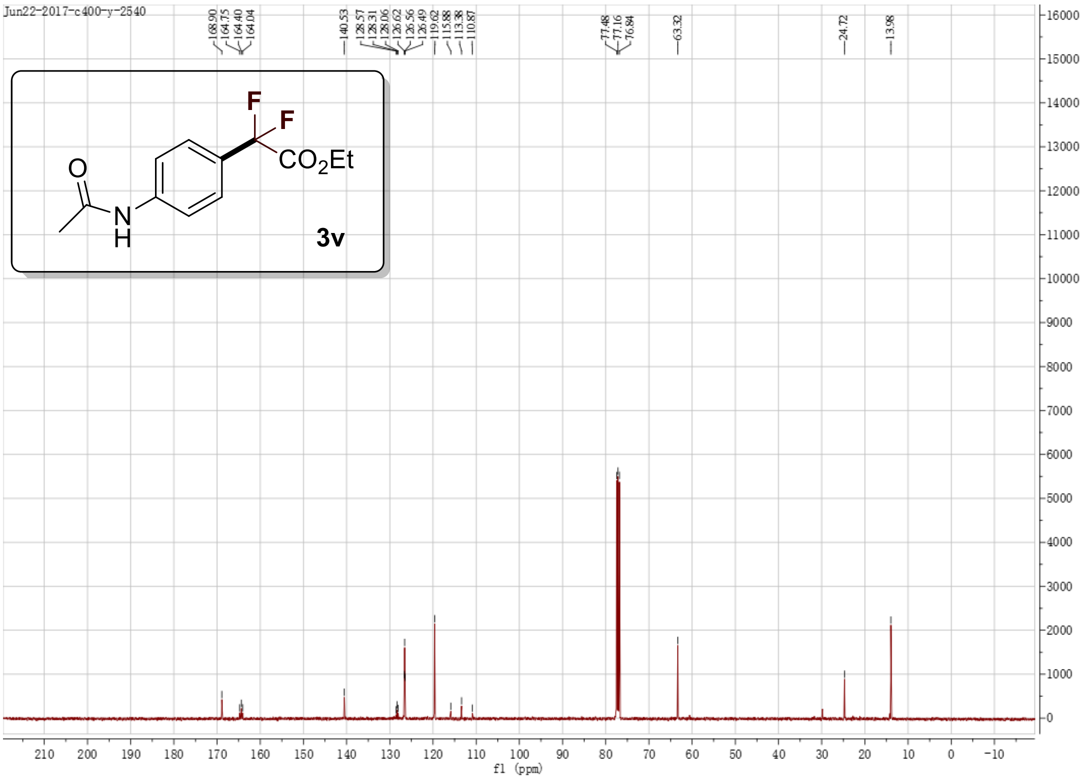

Supplementary Figure 124. ^{13}**C NMR spectra for 3v**

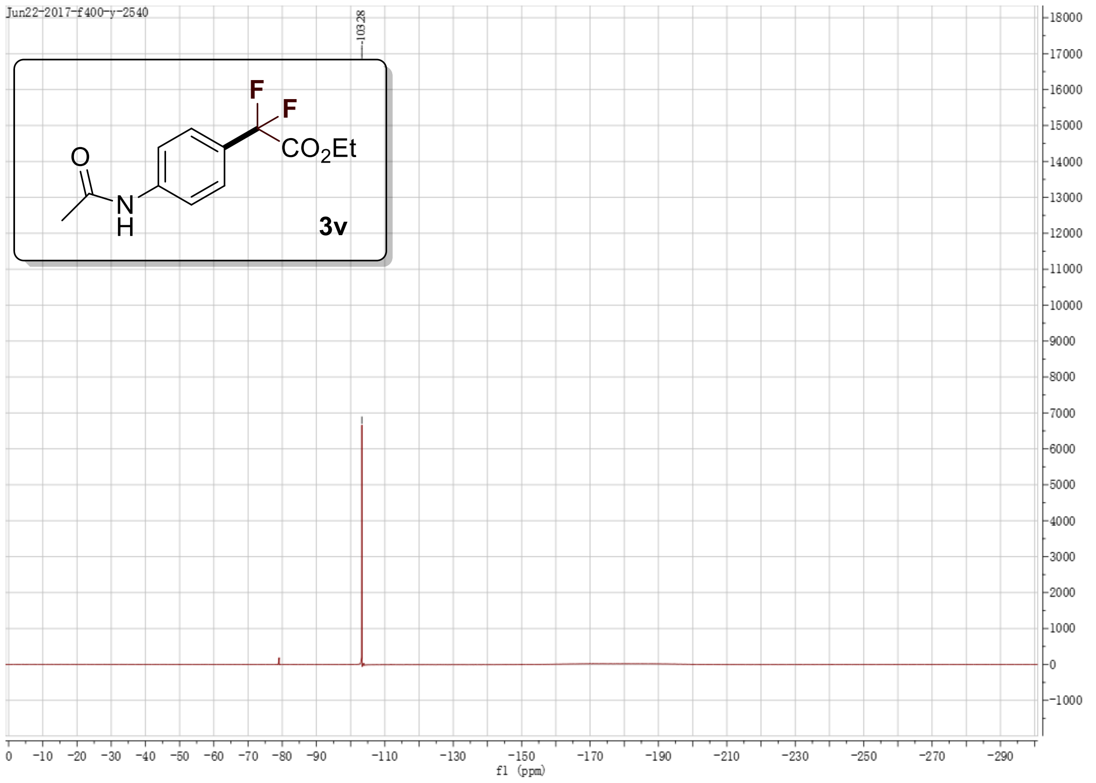

Supplementary Figure 125. ^{19}**F NMR spectra for 3v**

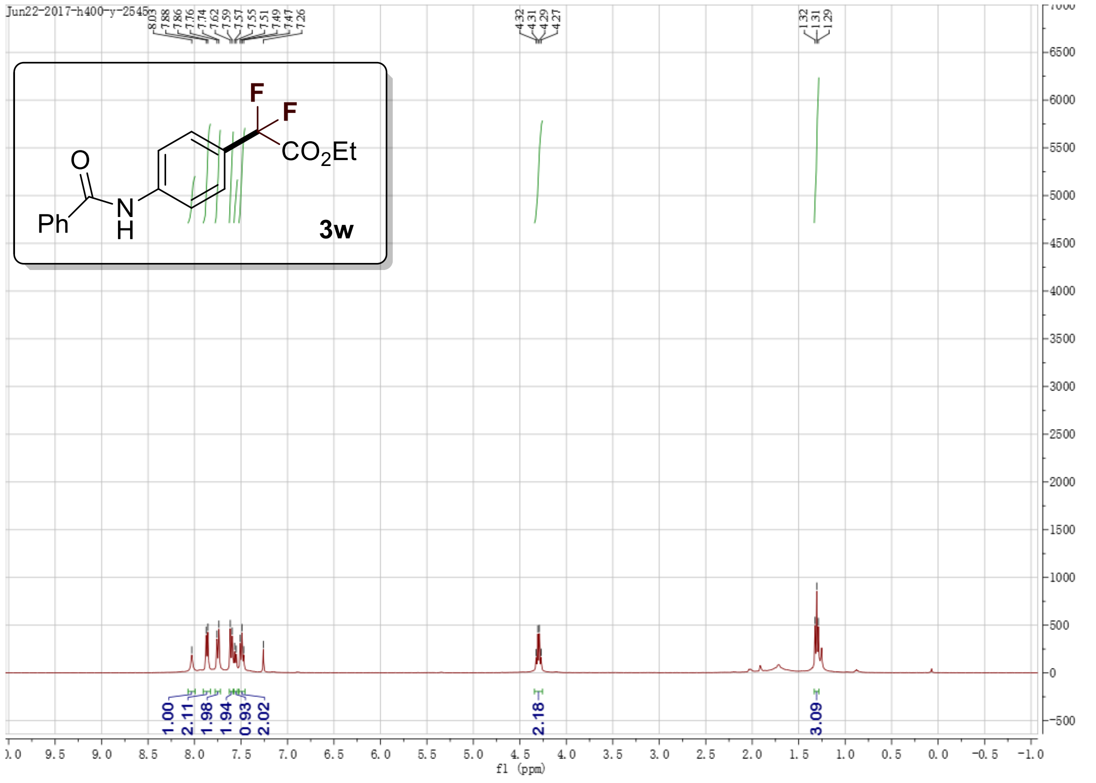

Supplementary Figure 126. ^{1}**H NMR spectra for 3w**

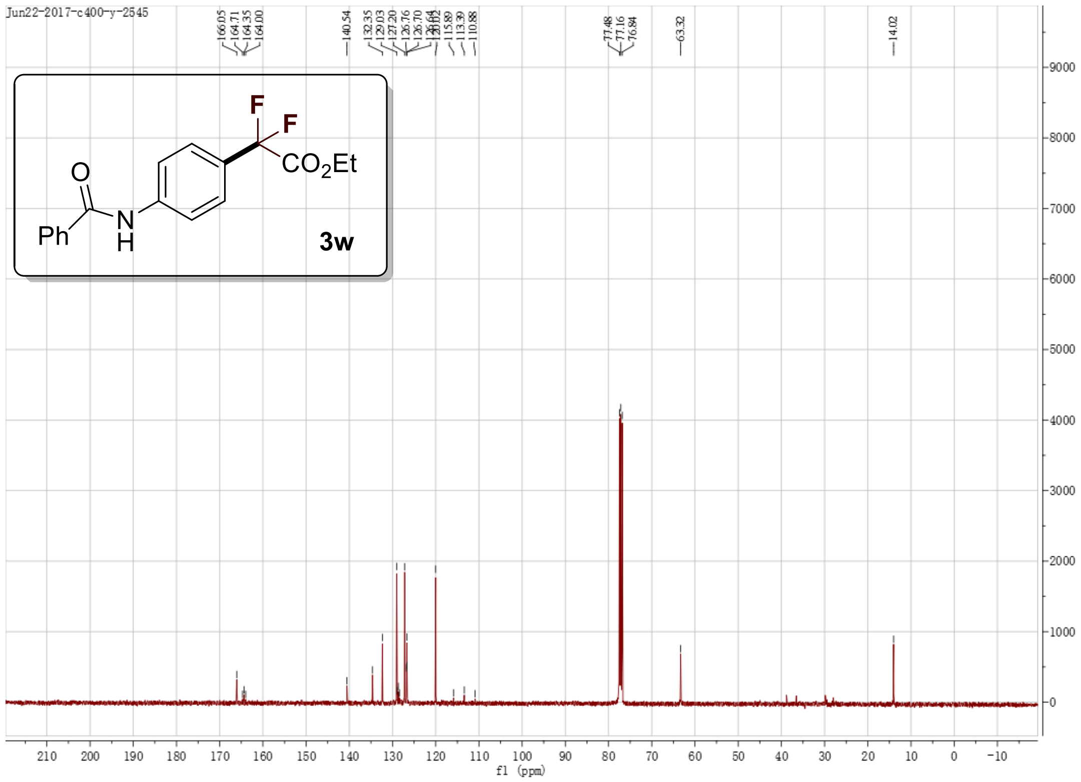

Supplementary Figure 127. ^{13}**C NMR spectra for 3w**

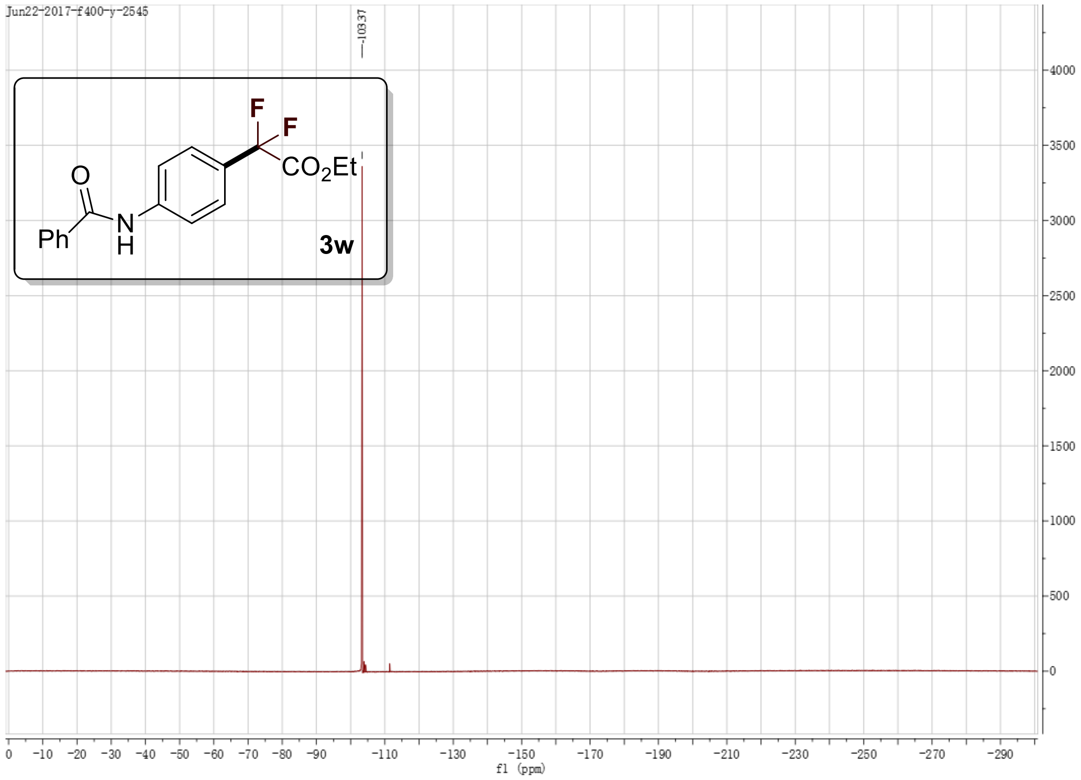

Supplementary Figure 128. ^{19}**F NMR spectra for 3w**

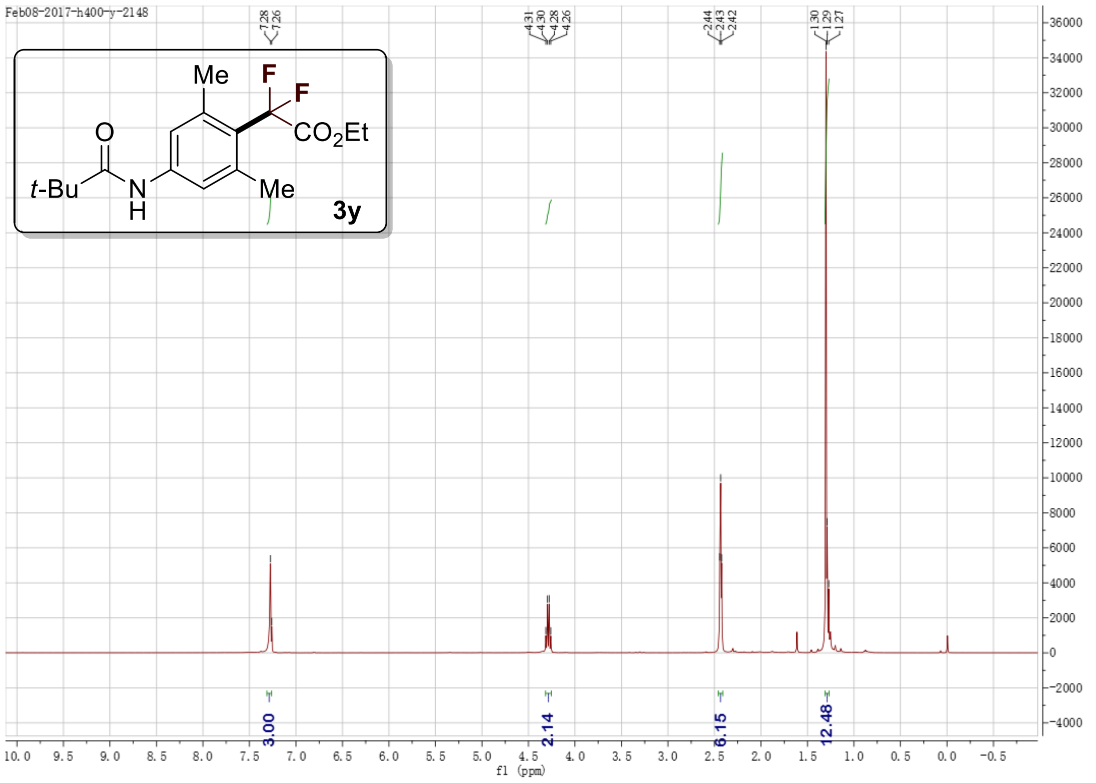

Supplementary Figure 129. ^{1}H NMR spectra for 3y

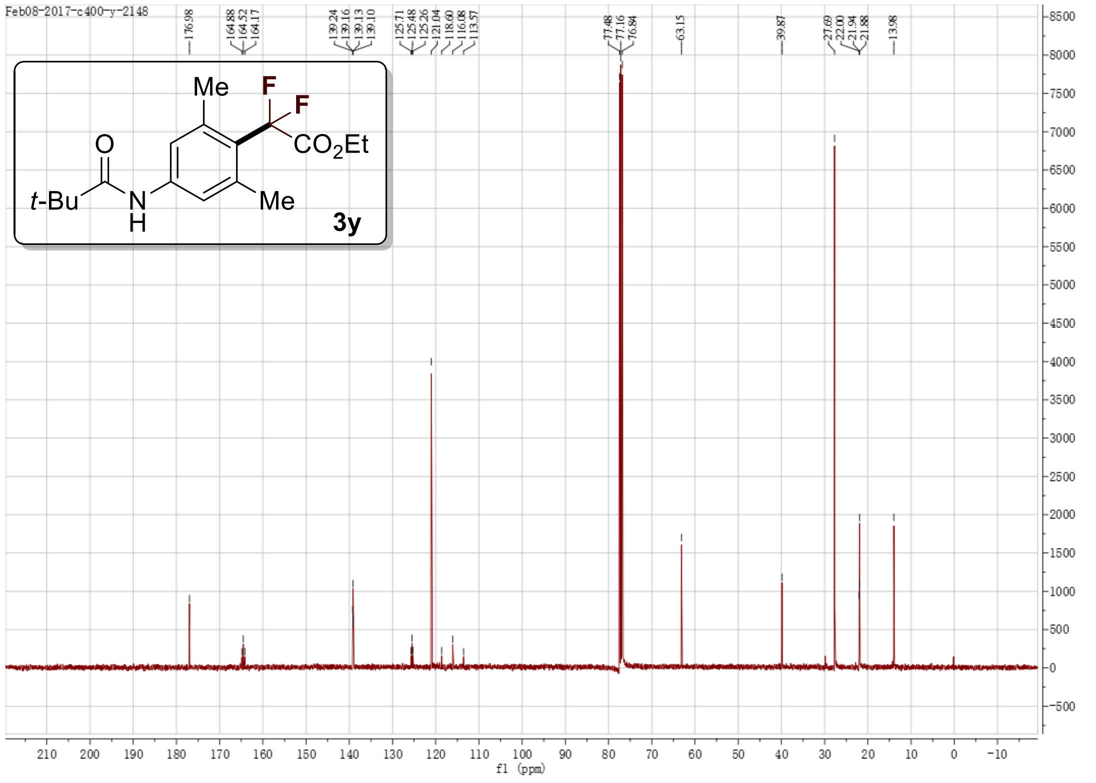

Supplementary Figure 130. ^{13}C NMR spectra for 3y

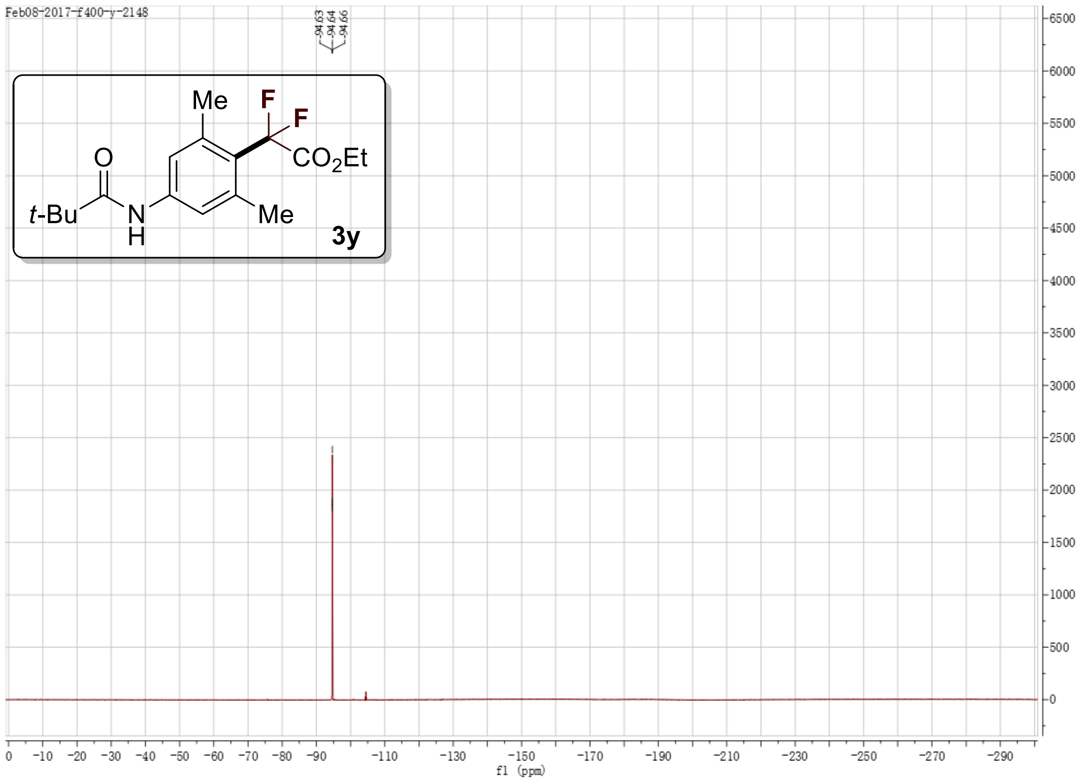

Supplementary Figure 131. ^{19}**F NMR spectra for 3y**

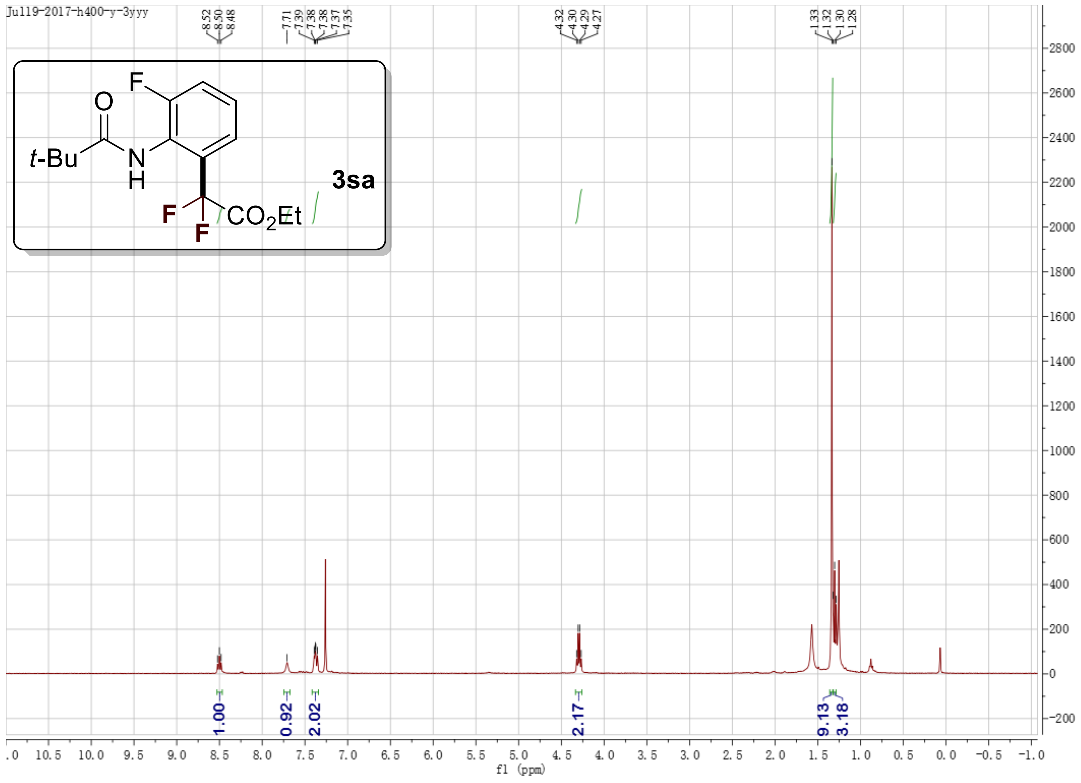

Supplementary Figure 132. ^{1}**H NMR spectra for 3sa**

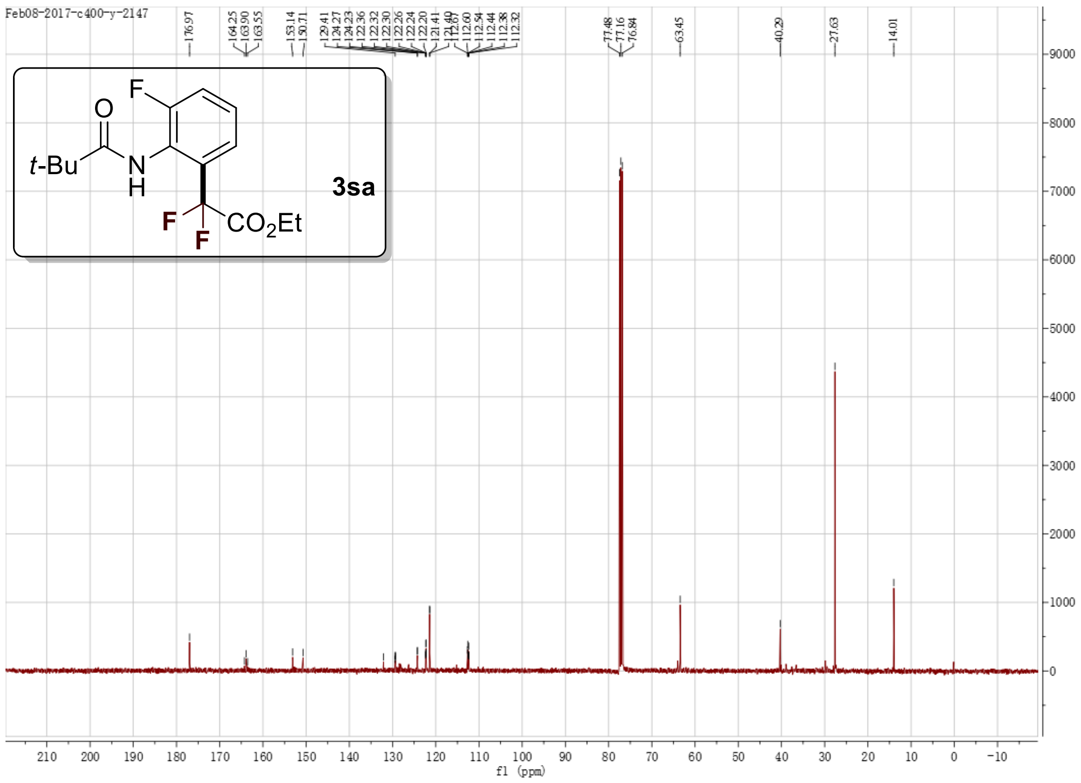

Supplementary Figure 133. ^{13}**C NMR spectra for 3sa**

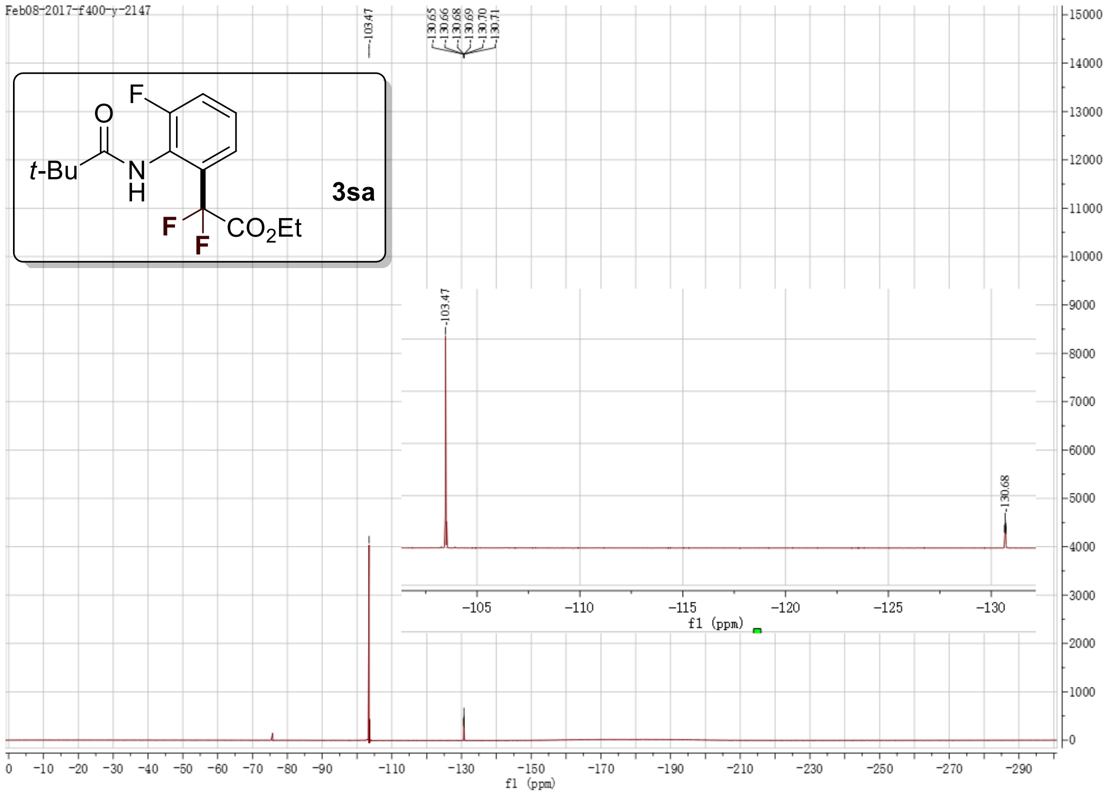

Supplementary Figure 134. ^{19}**F NMR spectra for 3sa**

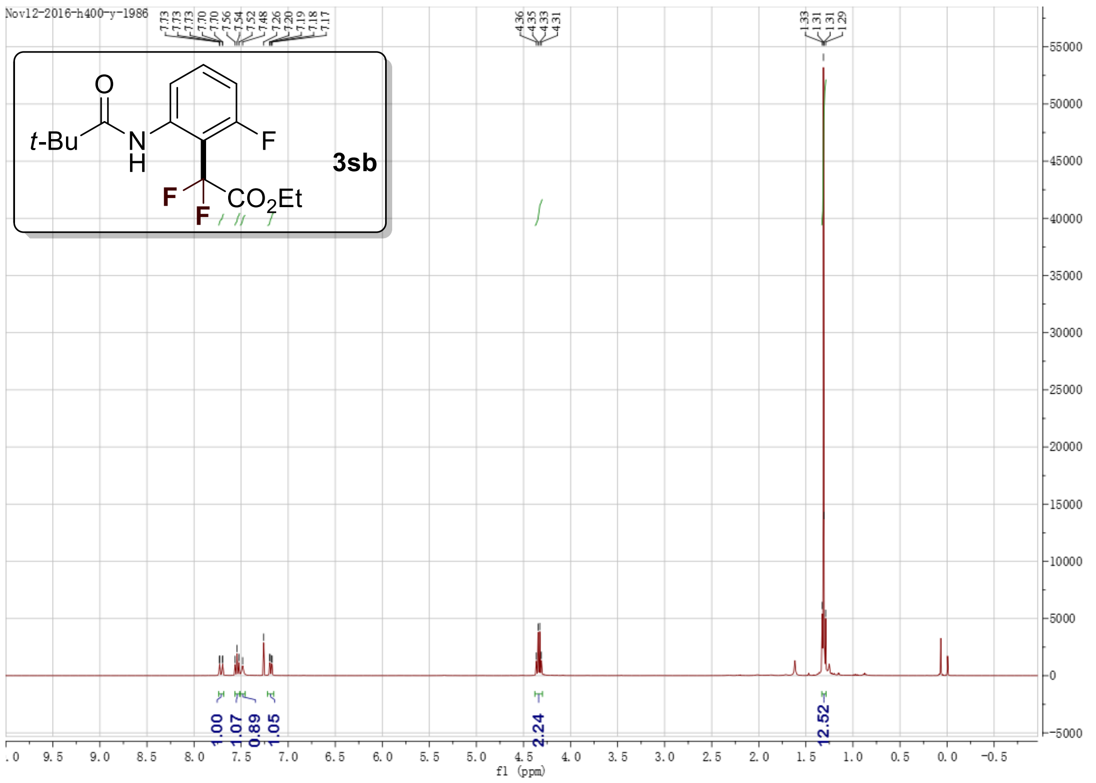

Supplementary Figure 135. ^{1}**H NMR spectra for 3sb**

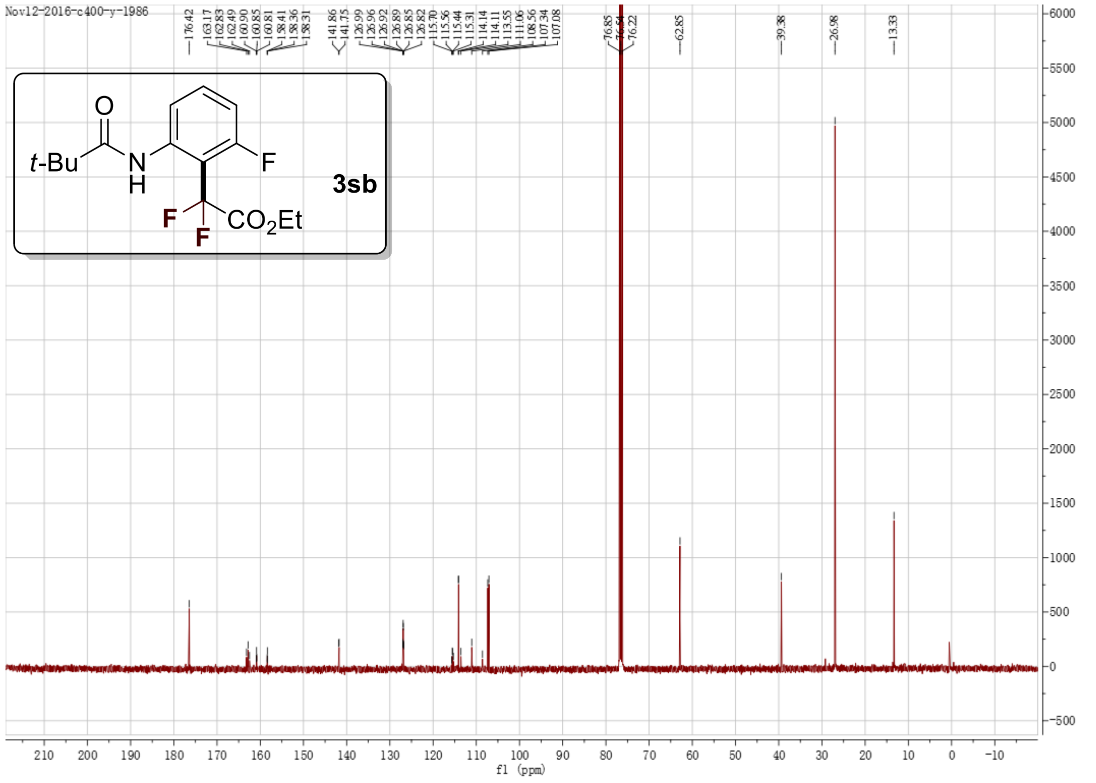

Supplementary Figure 136. ^{13}**C NMR spectra for 3sb**

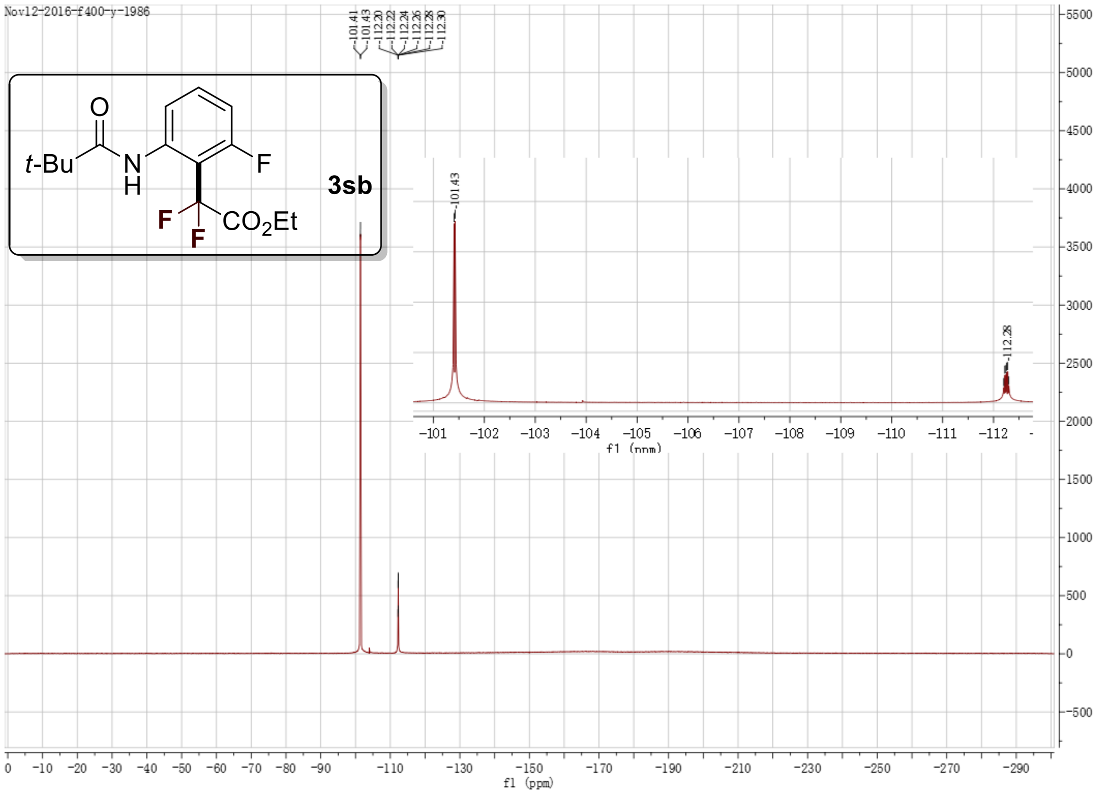

Supplementary Figure 137. ^{19}F NMR spectra for 3sb

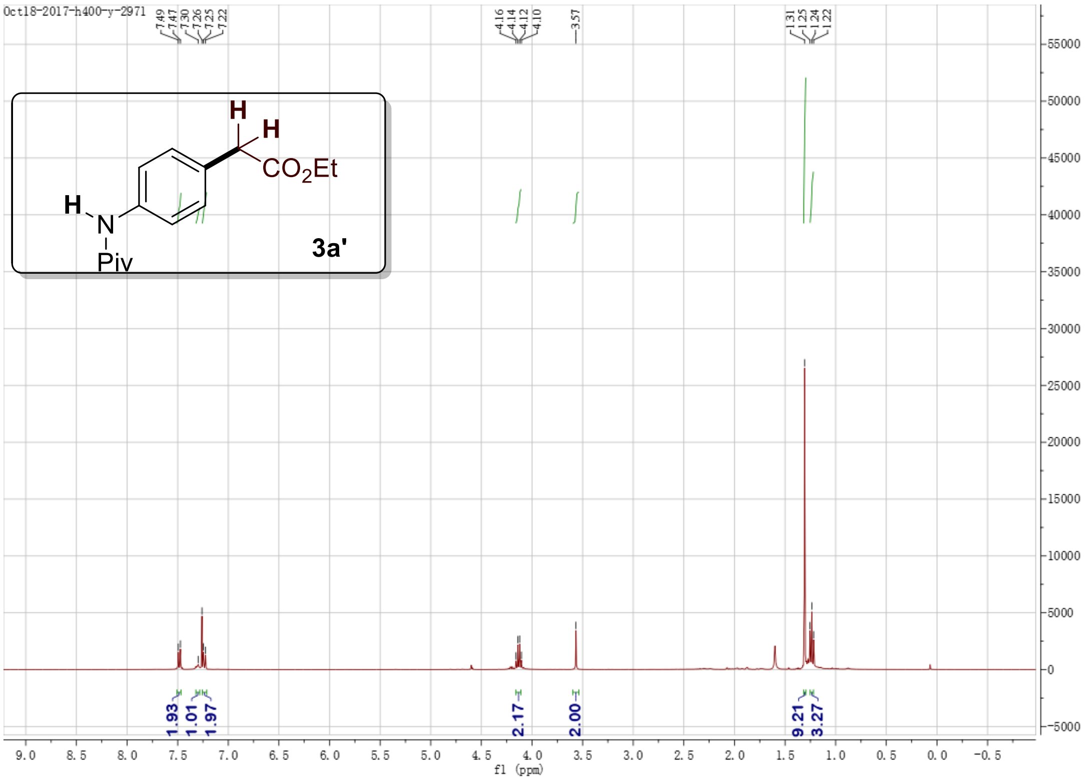

Supplementary Figure 138. ^{1}H NMR spectra for 3a'

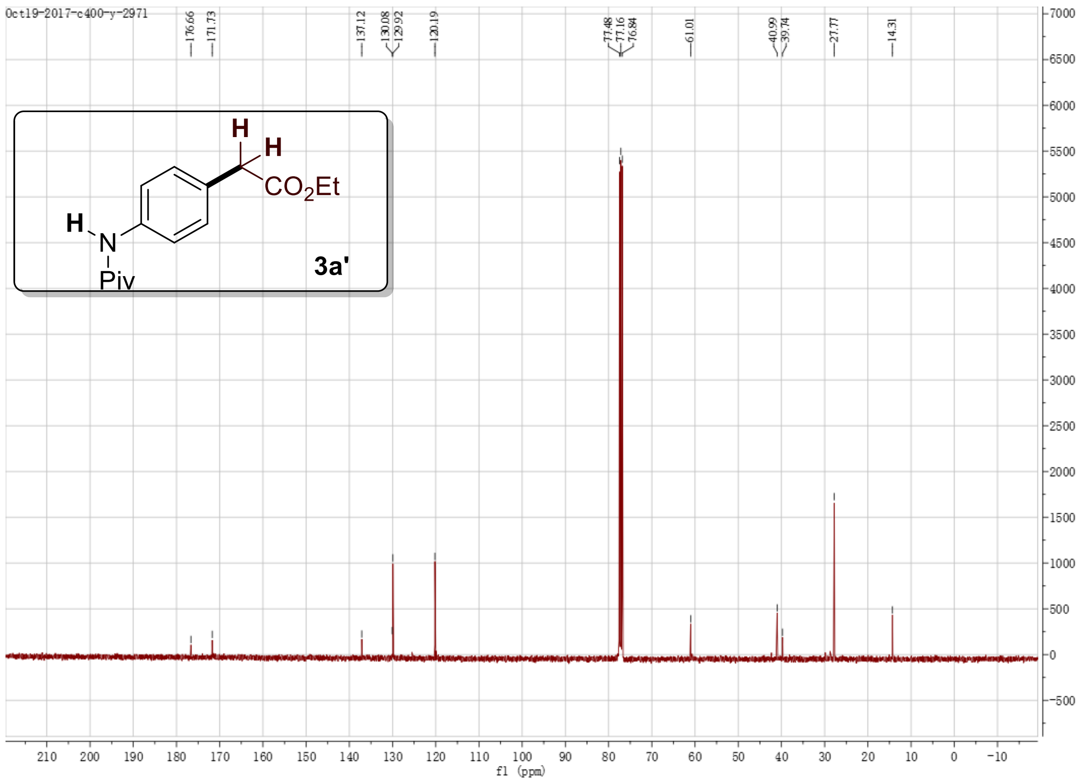

Supplementary Figure 139. ^{13}C NMR spectra for 3a'

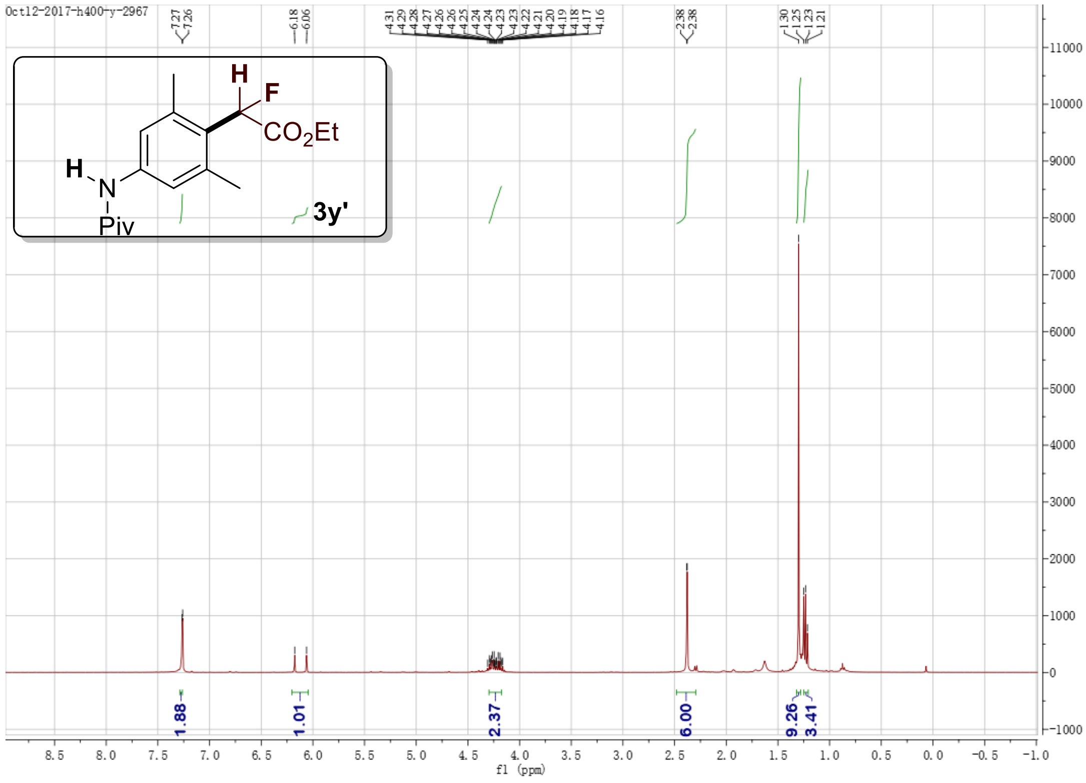

Supplementary Figure 140. ^{1}H NMR spectra for 3y'

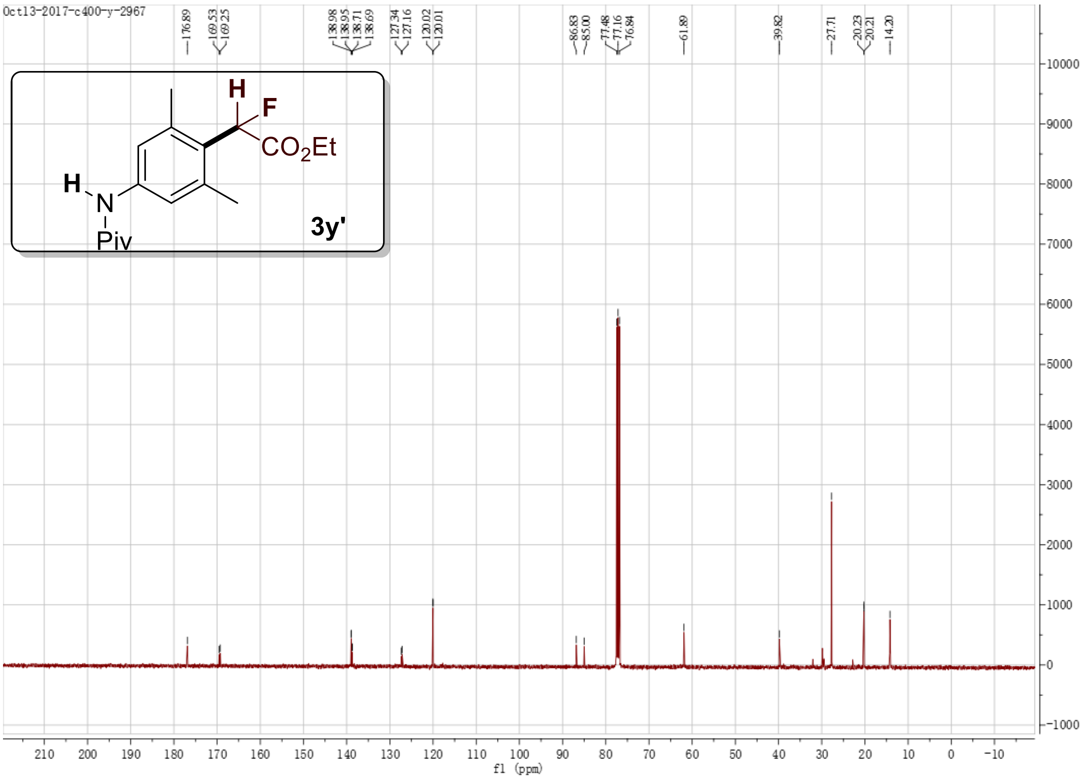

Supplementary Figure 141. ^{13}C NMR spectra for 3y'

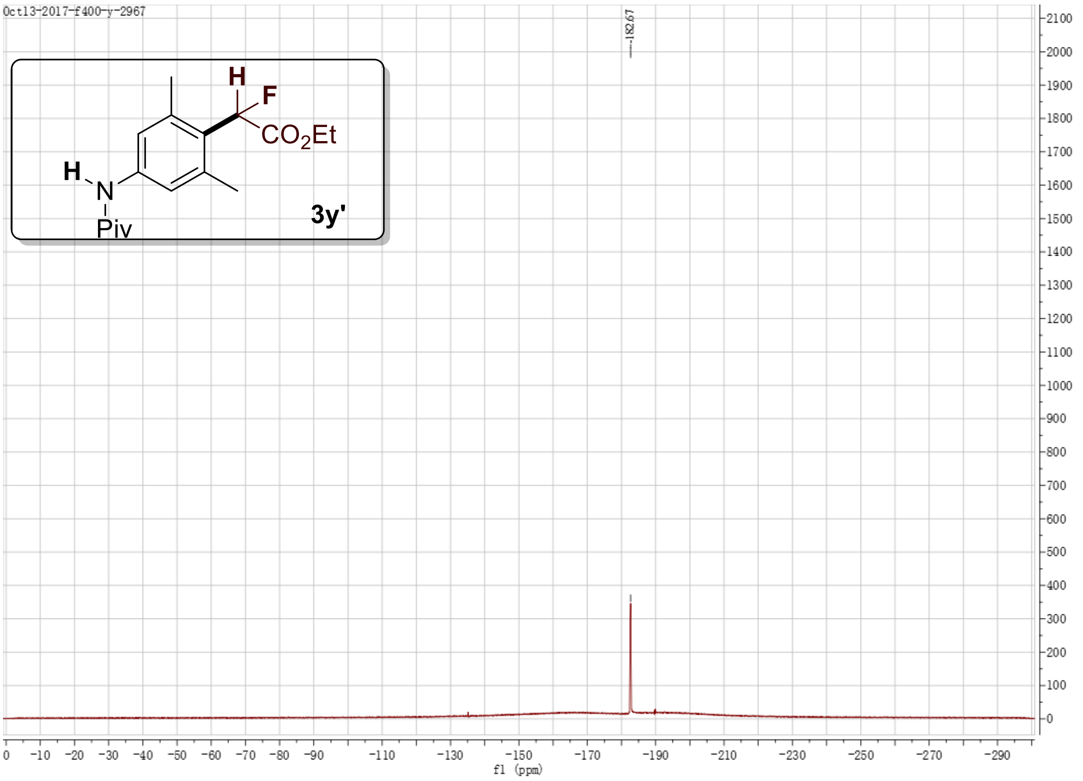

Supplementary Figure 142. ^{19}F NMR spectra for 3y'

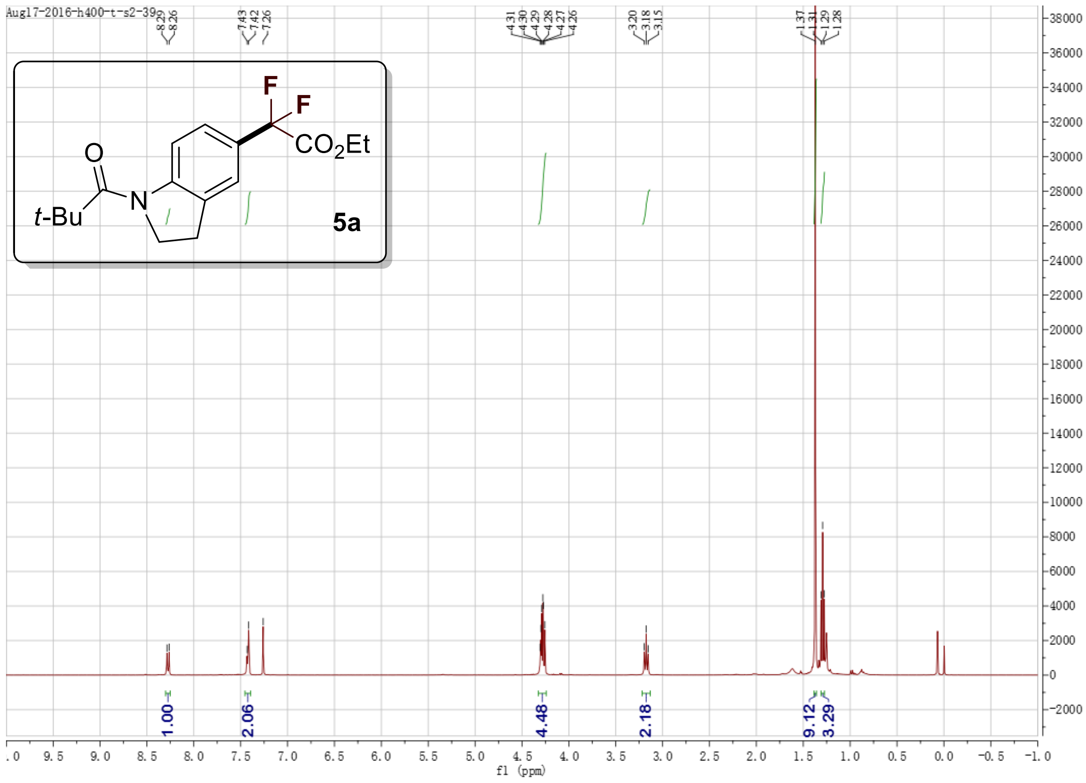

Supplementary Figure 143. ^{1}H NMR spectra for 5a

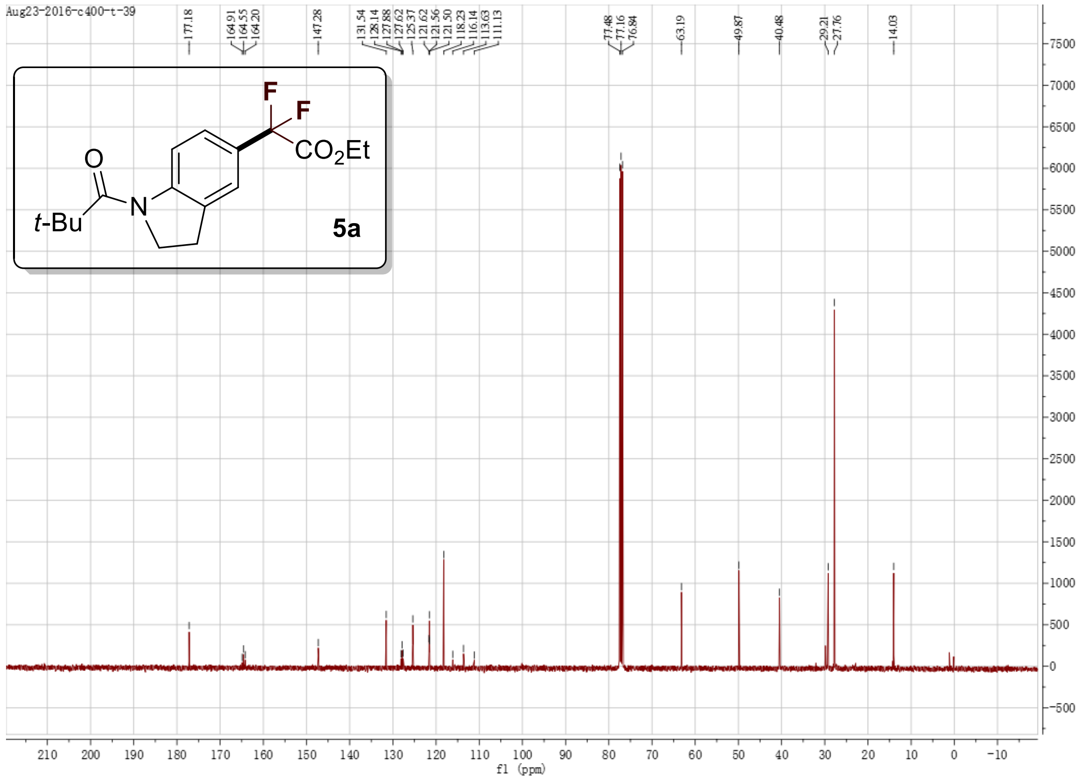

Supplementary Figure 144. ^{13}C NMR spectra for 5a

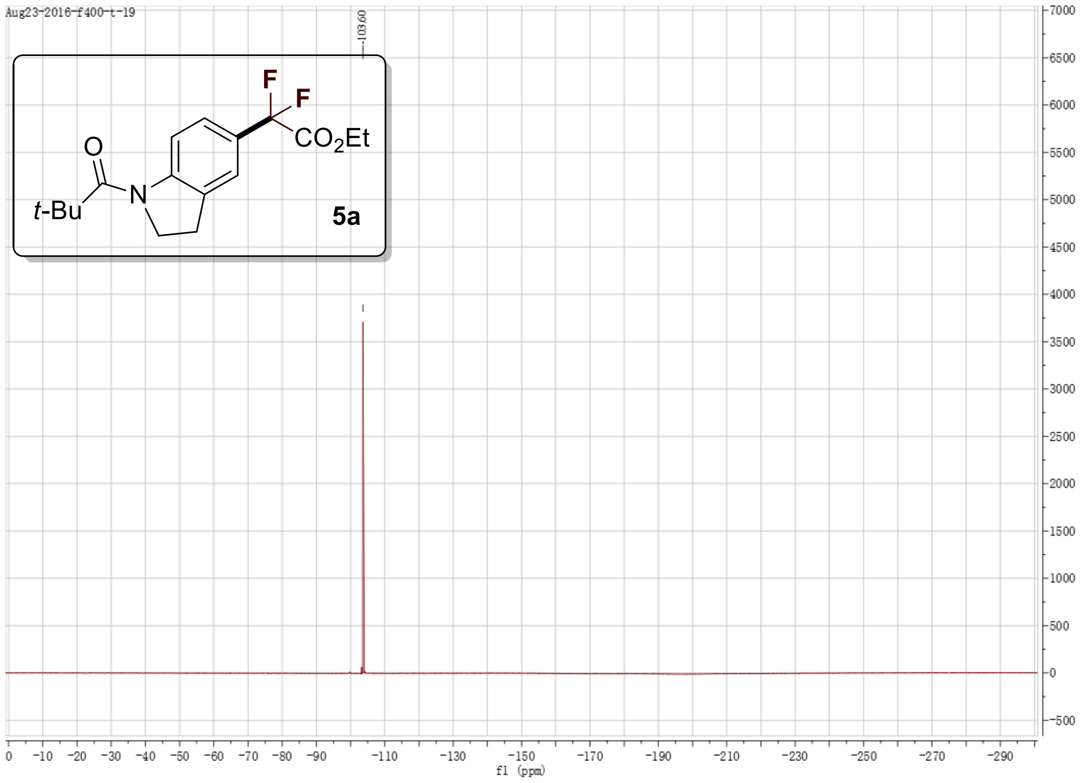

Supplementary Figure 145. ^{19}F NMR spectra for 5a

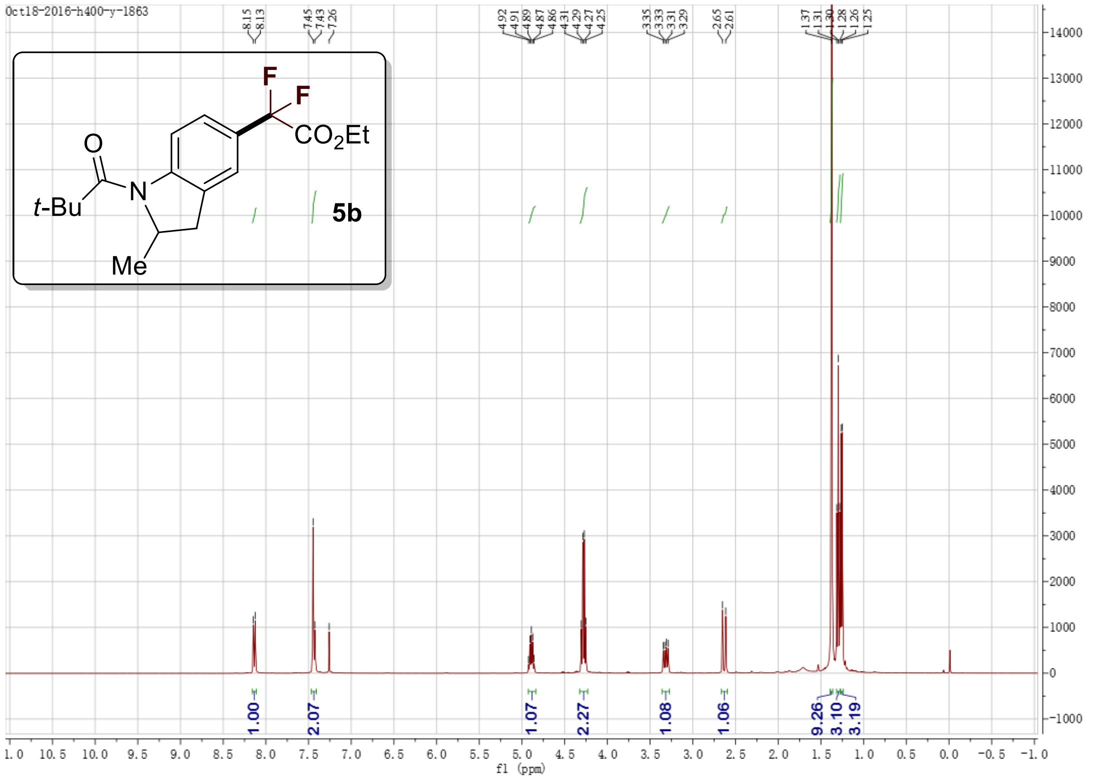

Supplementary Figure 146. ^{1}H NMR spectra for 5b

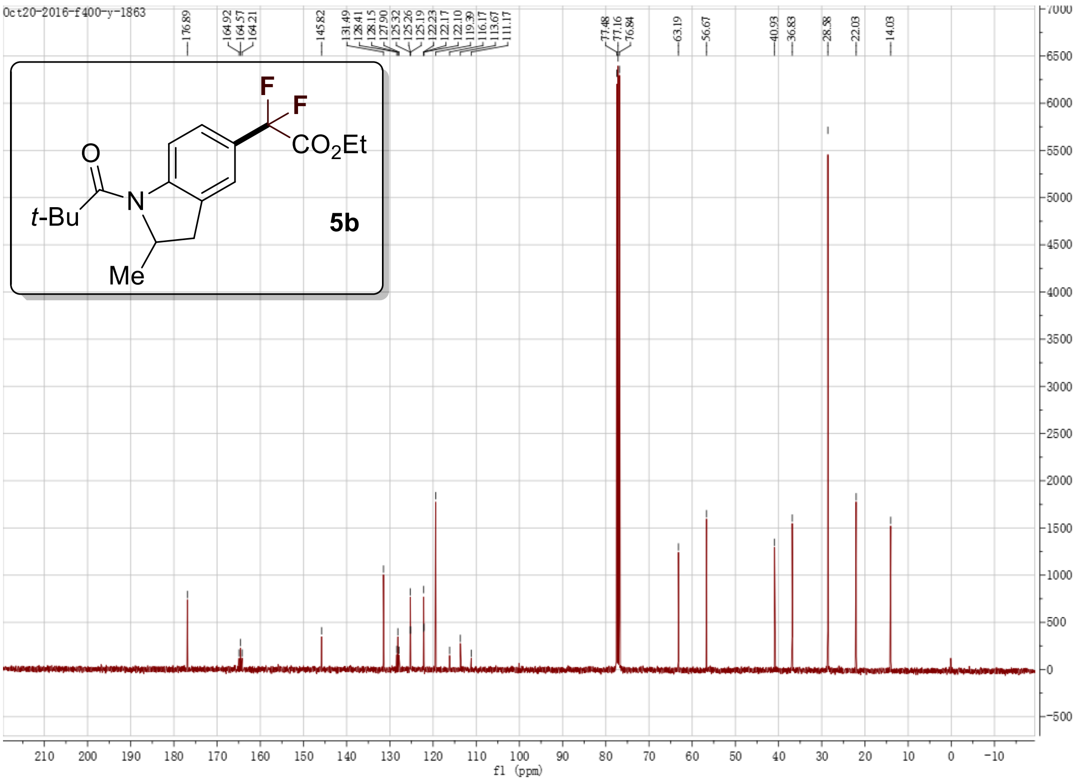

Supplementary Figure 147. ^{13}**C NMR spectra for 5b**

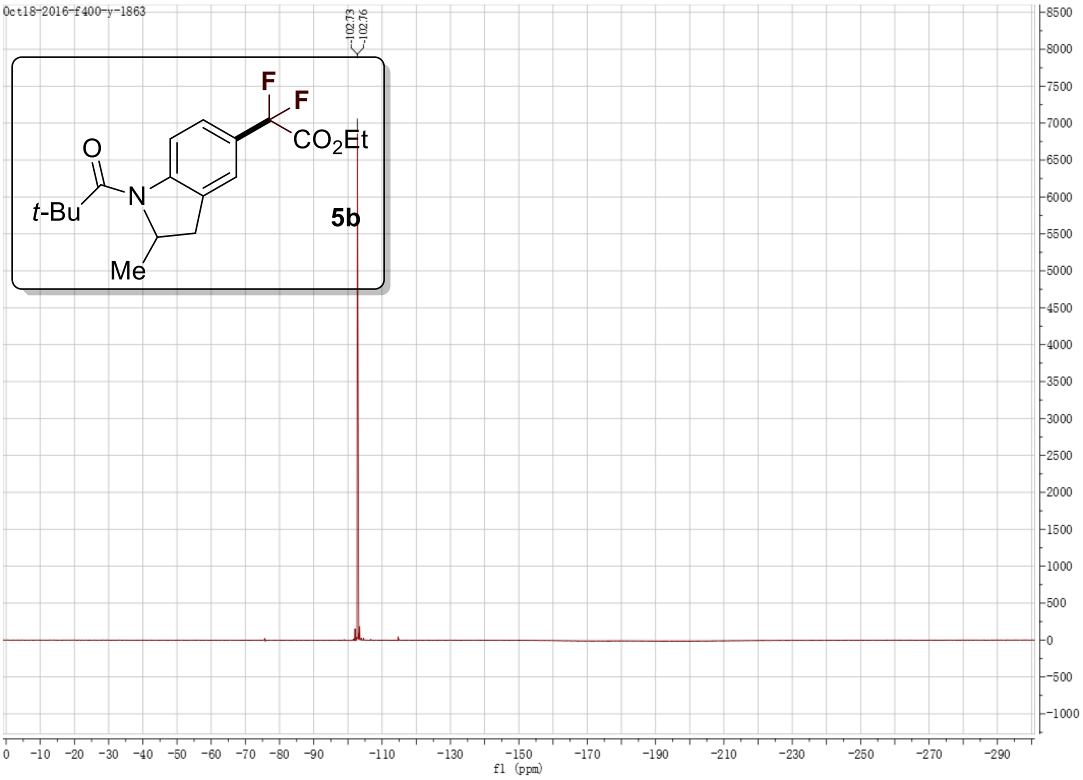

Supplementary Figure 148. ^{19}**F NMR spectra for 5b**

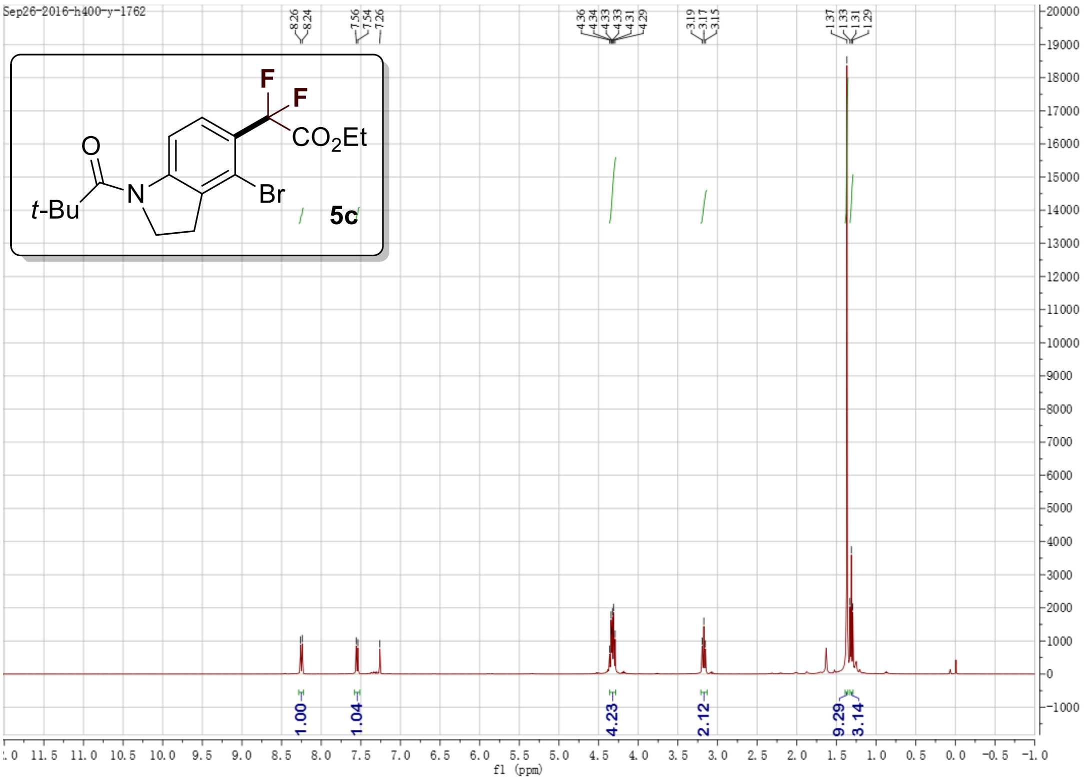

Supplementary Figure 149. ^{1}H NMR spectra for 5c

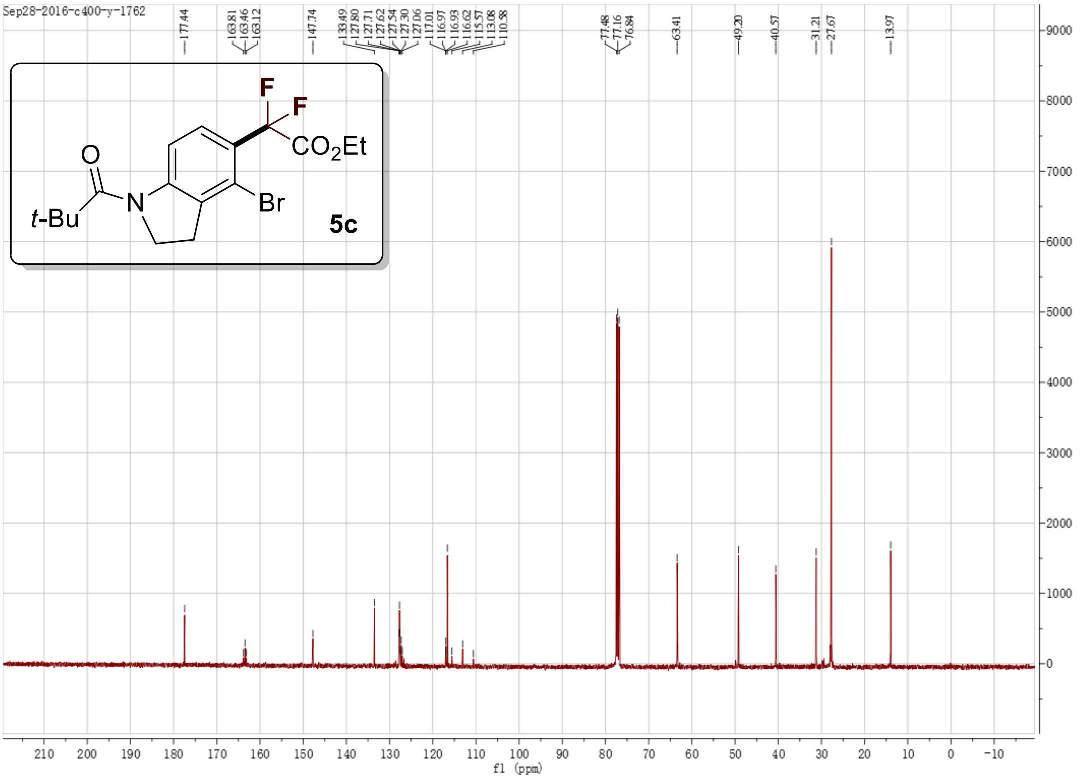

Supplementary Figure 150. ^{13}C NMR spectra for 5c

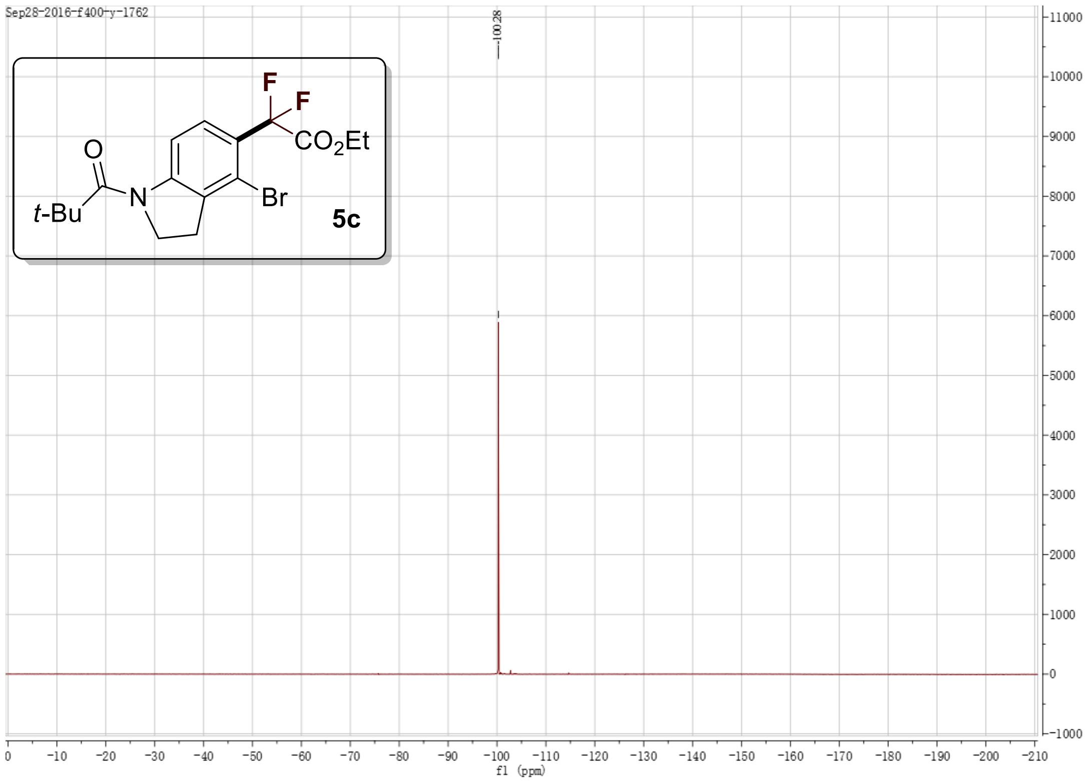

Supplementary Figure 151. ^{19}F NMR spectra for 5c

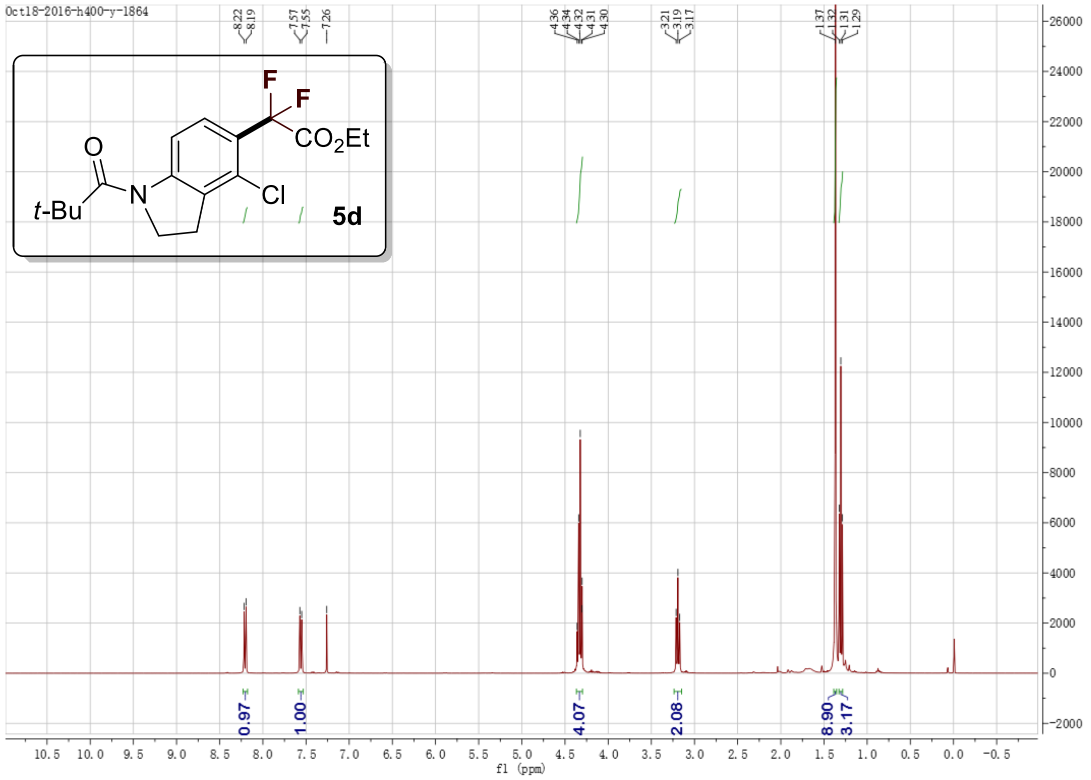

Supplementary Figure 152. ^{1}H NMR spectra for 5d

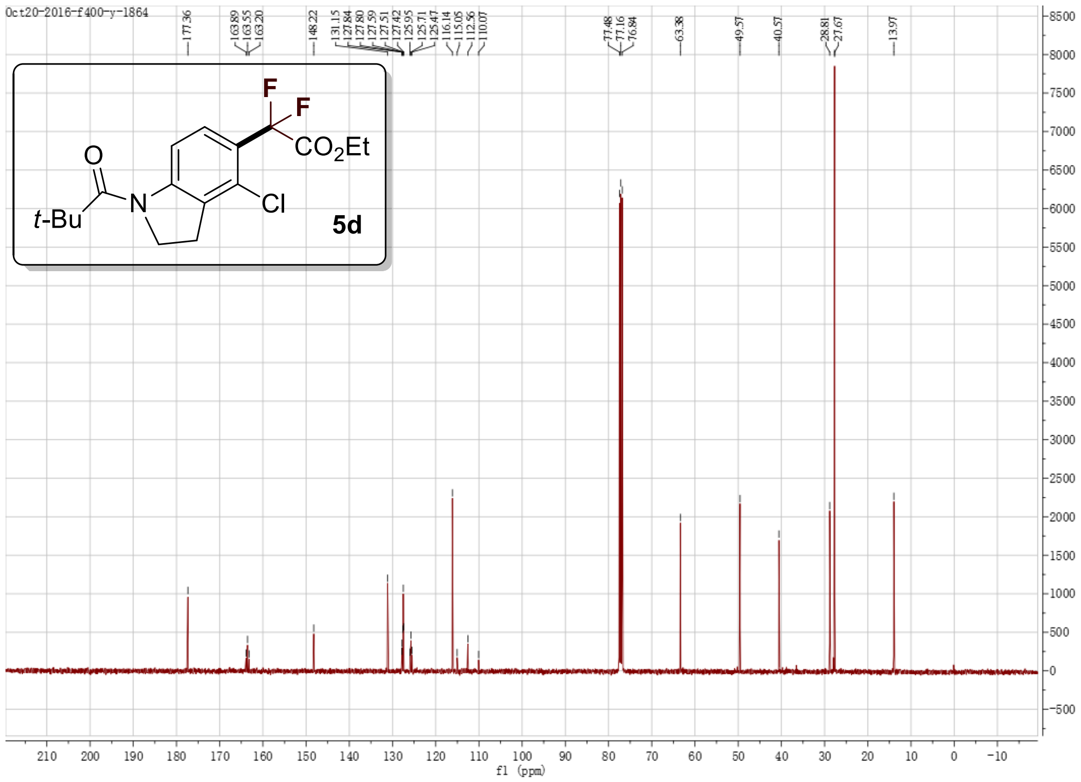

Supplementary Figure 153. ^{13}**C NMR spectra for 5d**

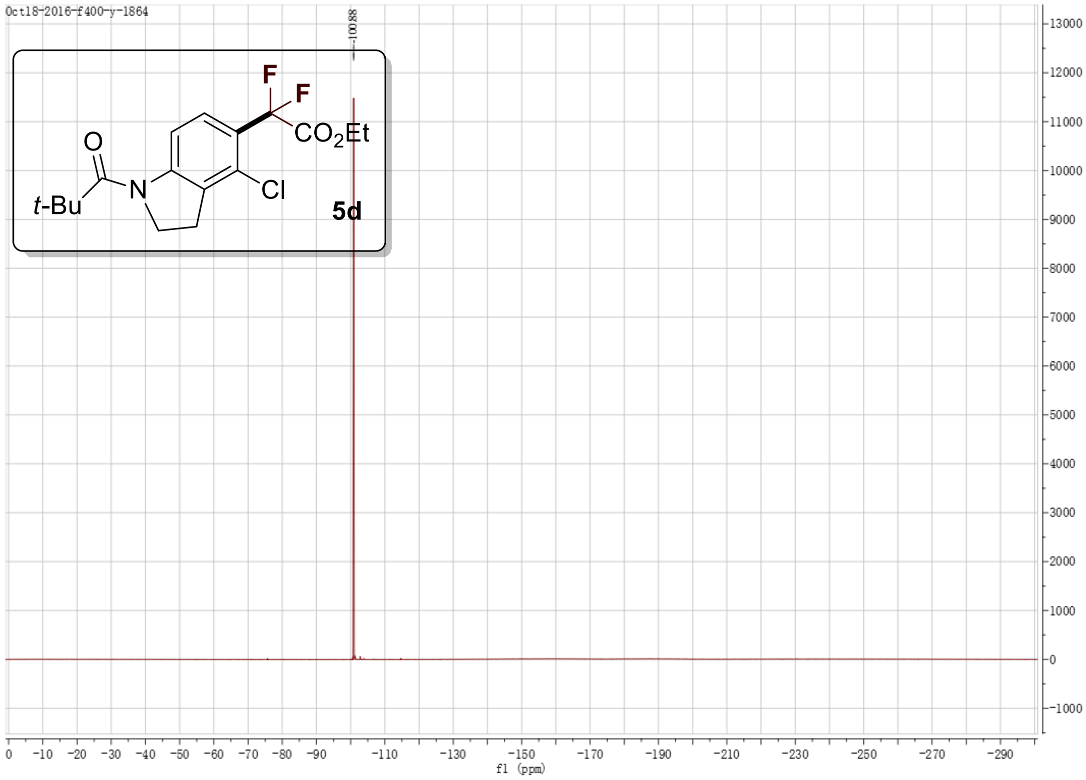

Supplementary Figure 154. ^{19}**F NMR spectra for 5d**

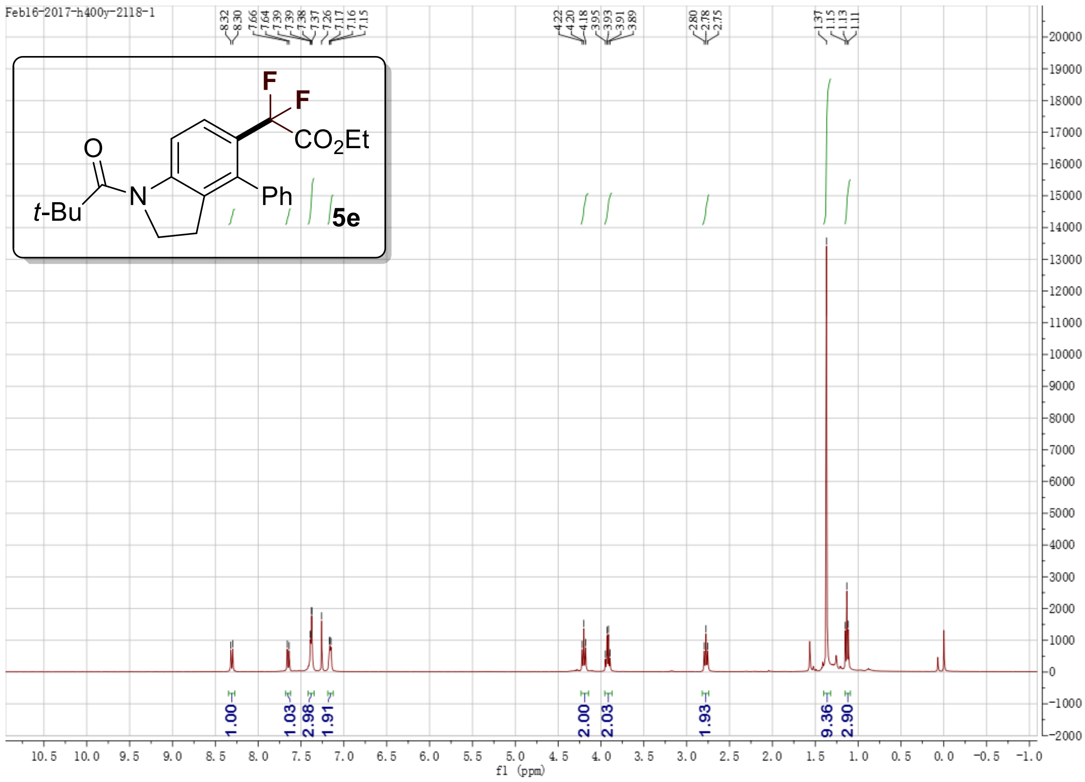

Supplementary Figure 155. ^{1}H NMR spectra for 5e

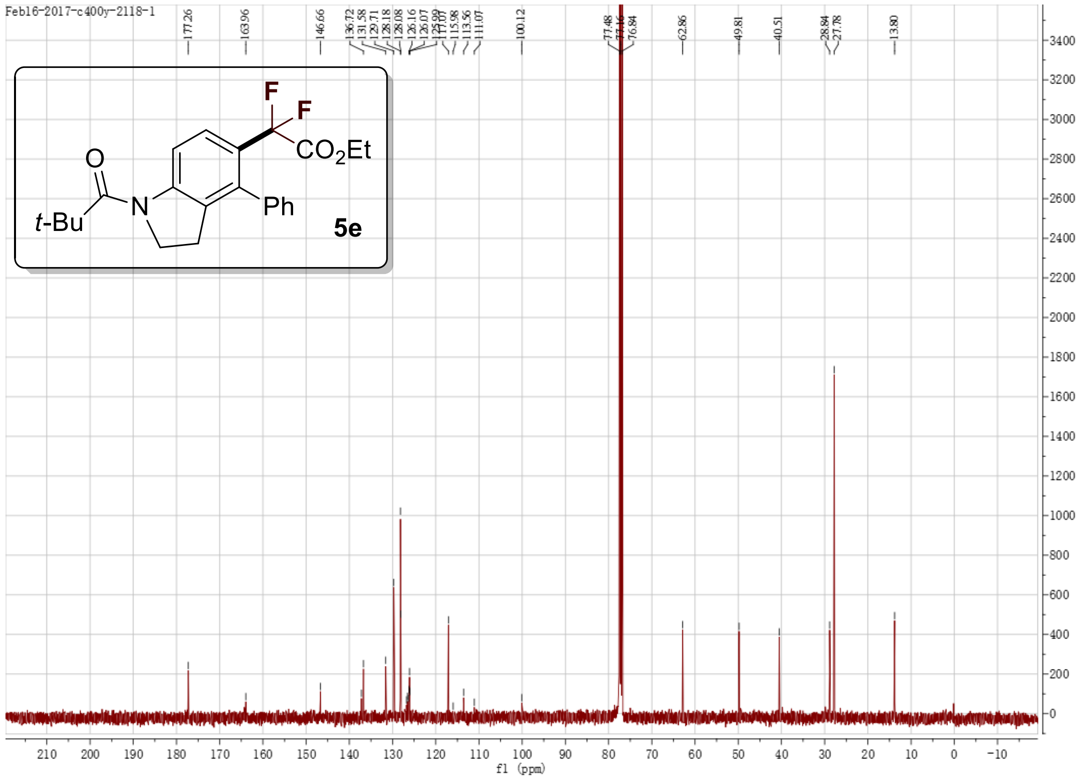

Supplementary Figure 156. ^{13}C NMR spectra for 5e

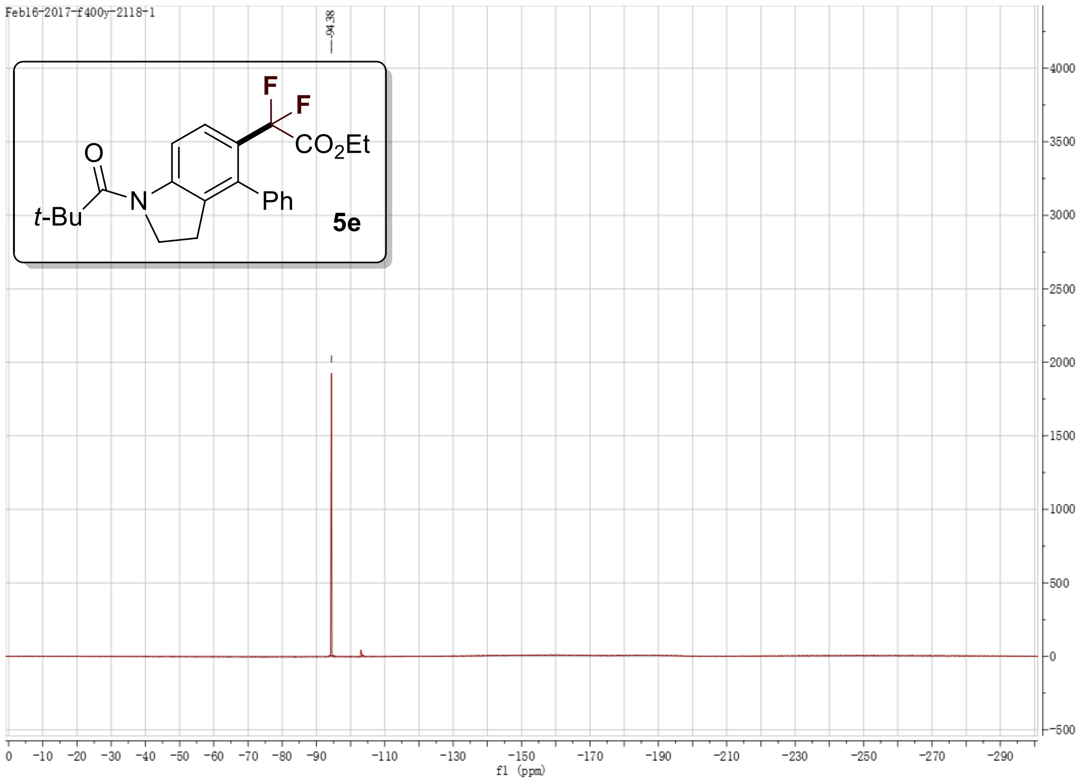

Supplementary Figure 157. ^{19}**F NMR spectra for 5e**

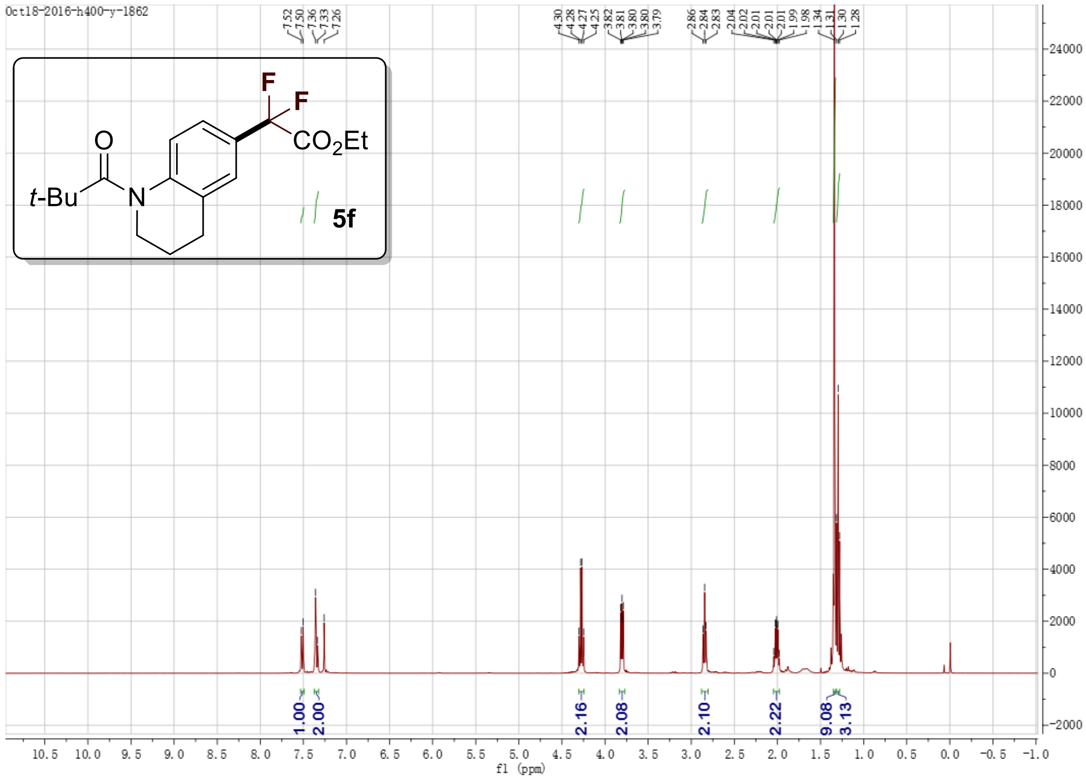

Supplementary Figure 158. ^{1}**H NMR spectra for 5f**

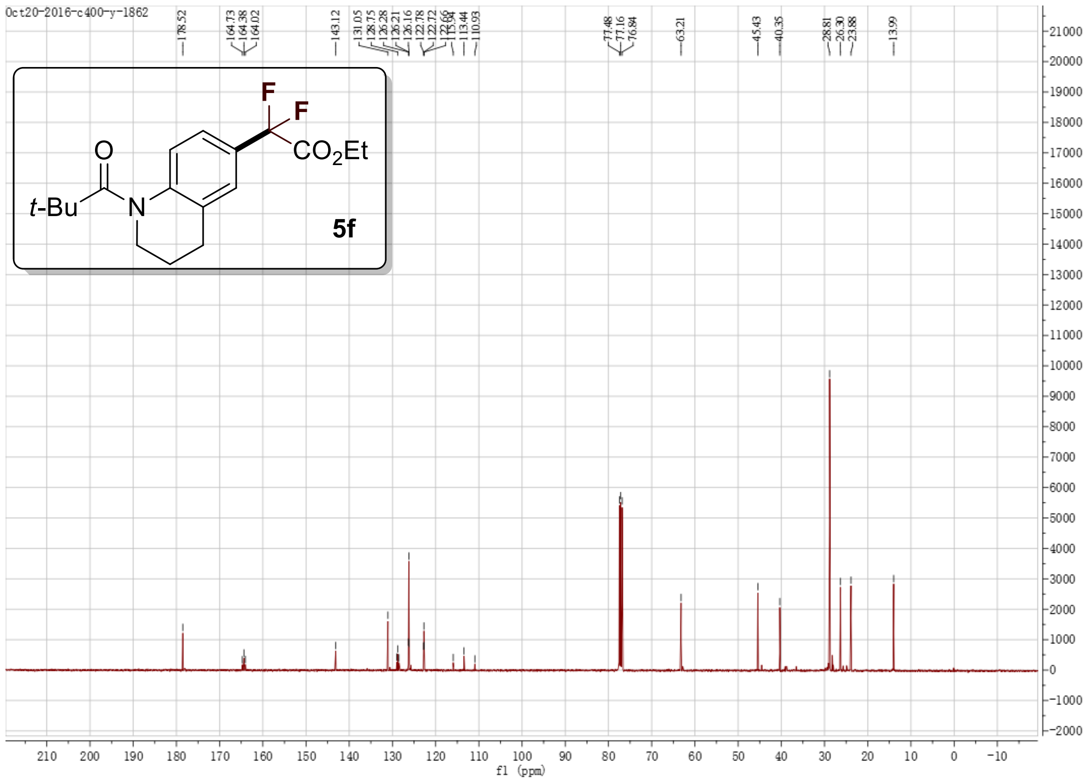

Supplementary Figure 159. ^{13}C NMR spectra for 5f

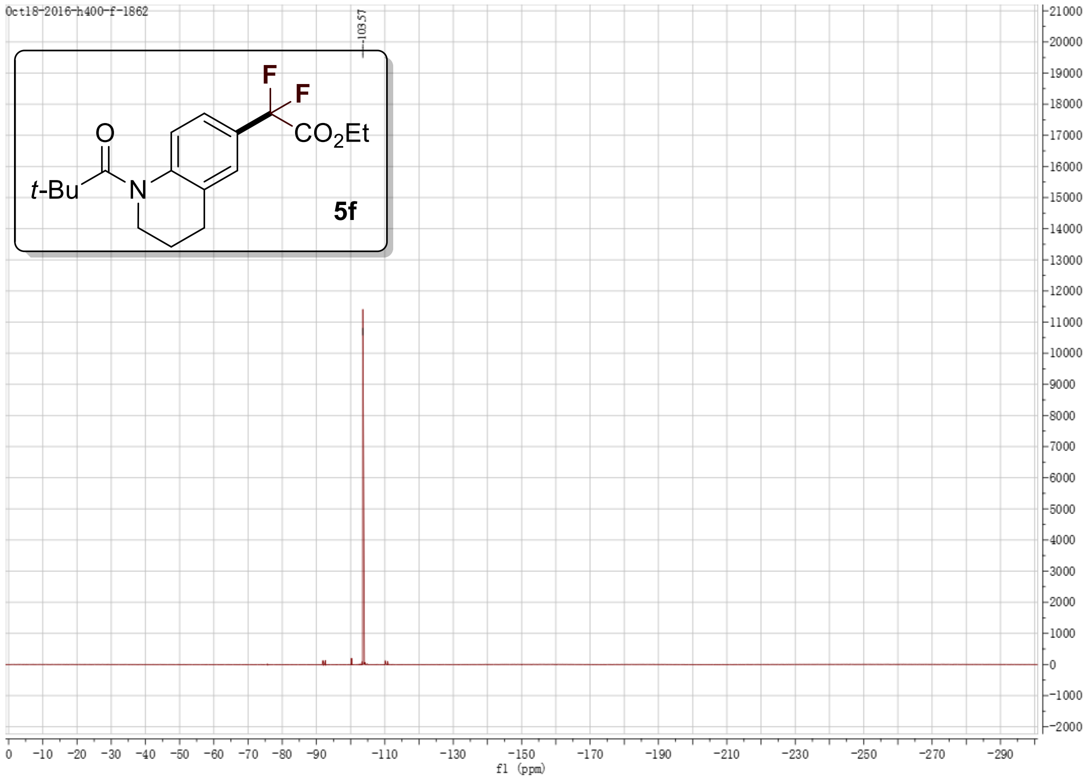

Supplementary Figure 160. ^{19}F NMR spectra for 5f

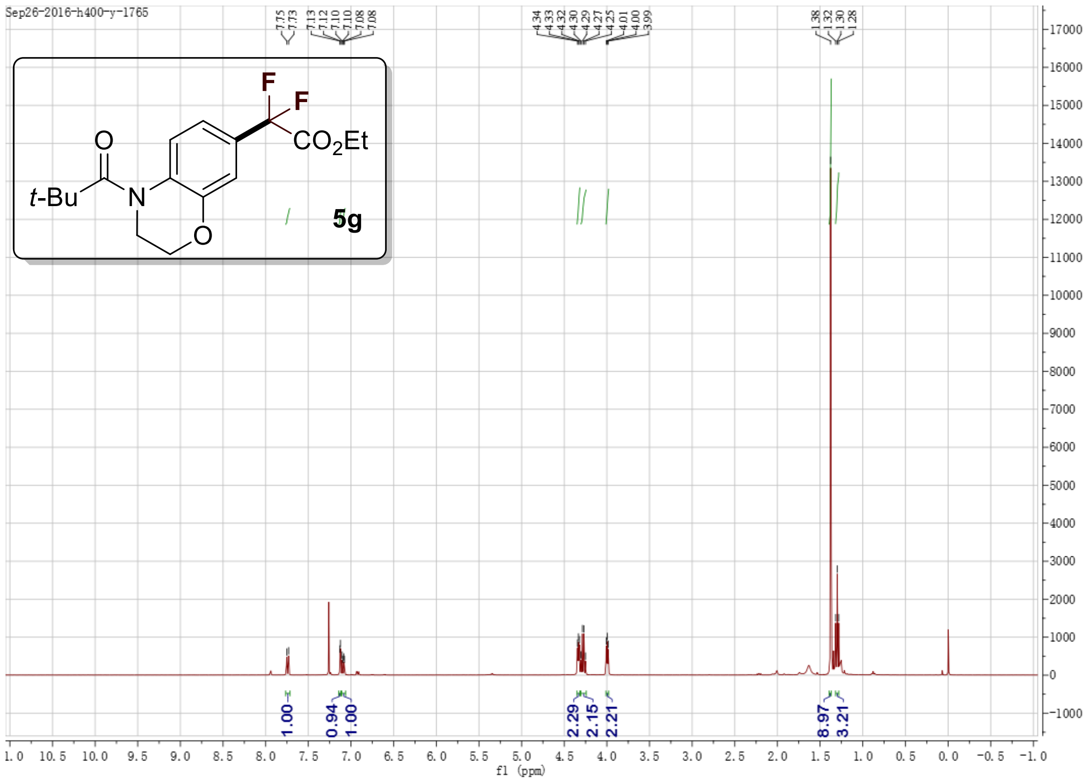

Supplementary Figure 161. ^{1}H NMR spectra for 5g

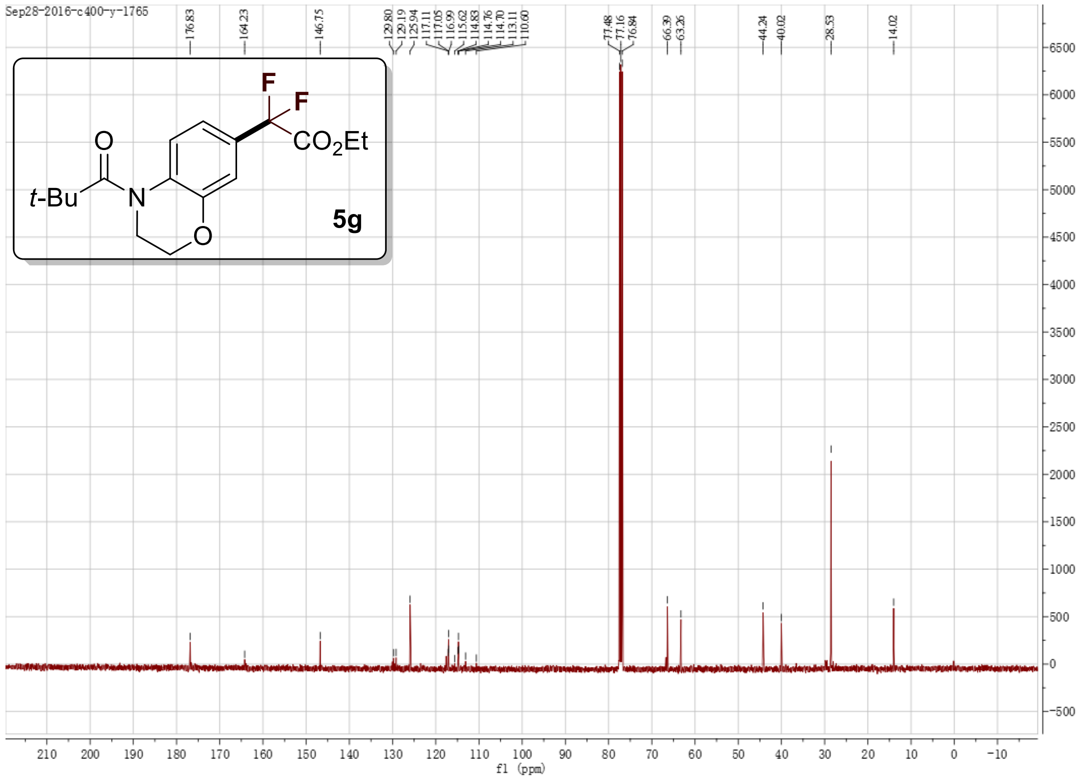

Supplementary Figure 162. ^{13}C NMR spectra for 5g

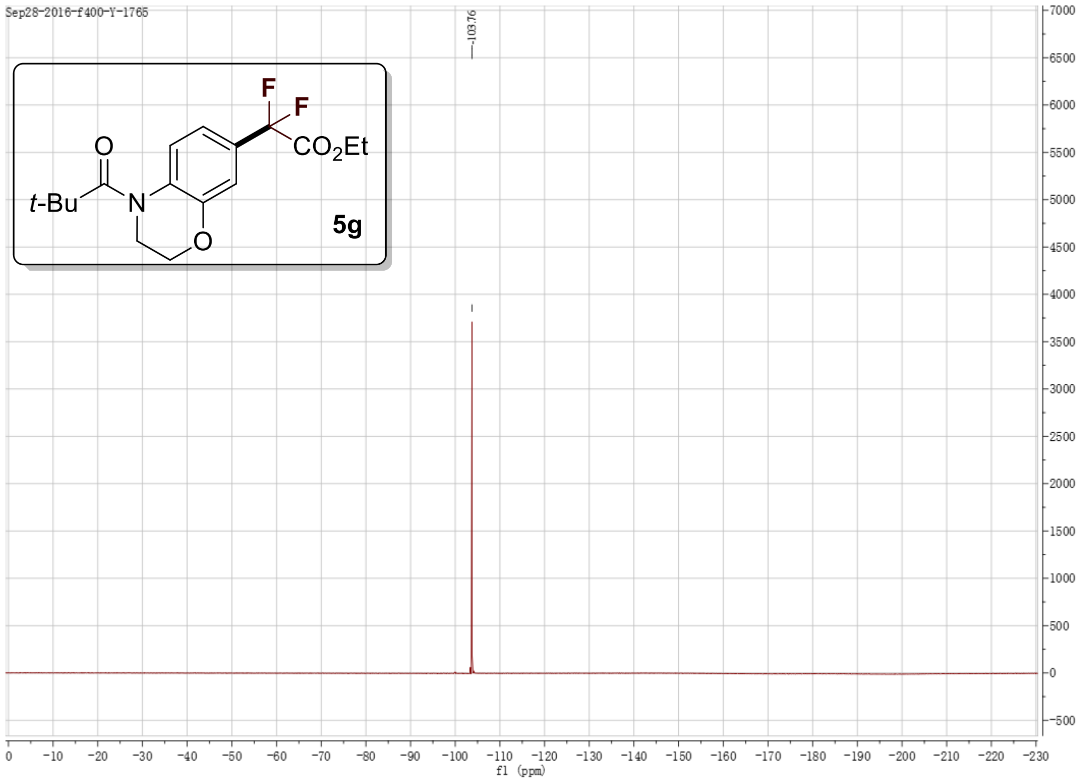

Supplementary Figure 163. ^{19}F NMR spectra for 5g

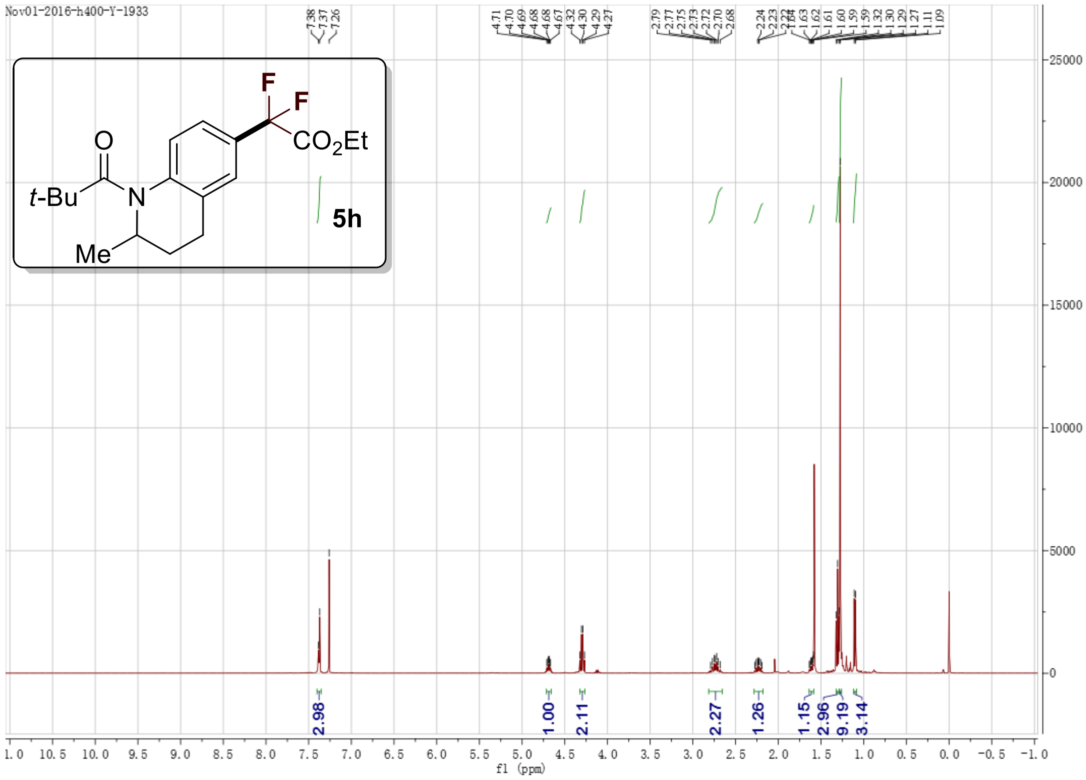

Supplementary Figure 164. ^{1}H NMR spectra for 5h

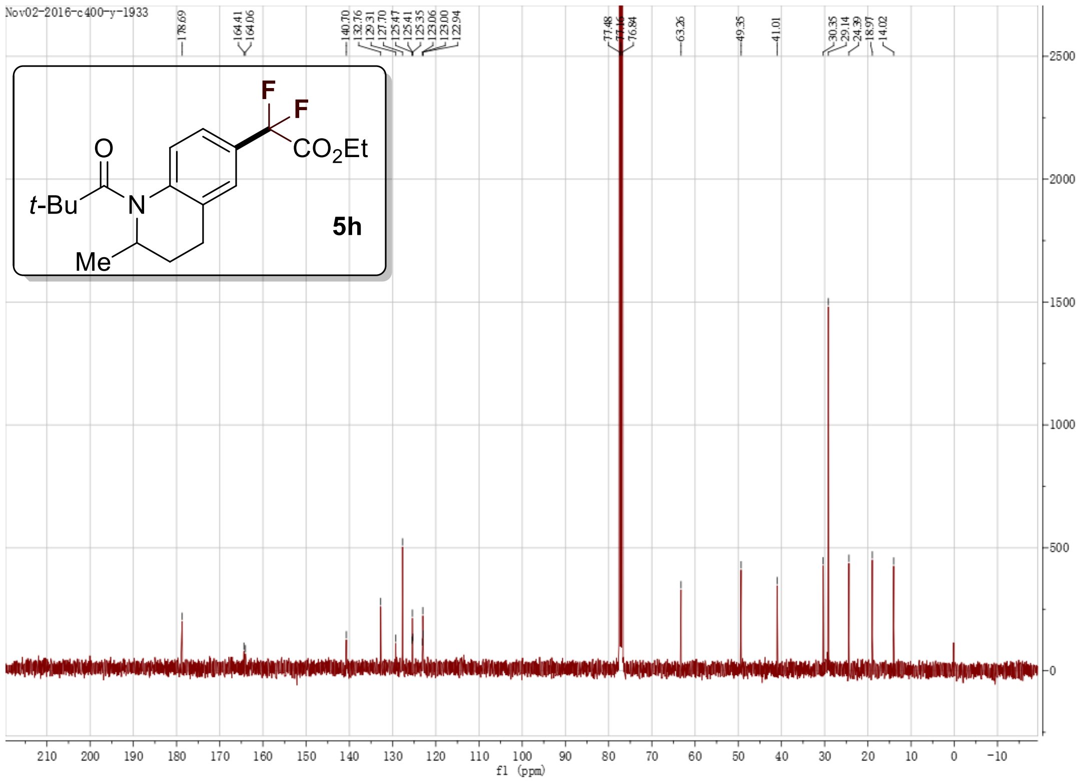

Supplementary Figure 165. ^{13}C NMR spectra for 5h

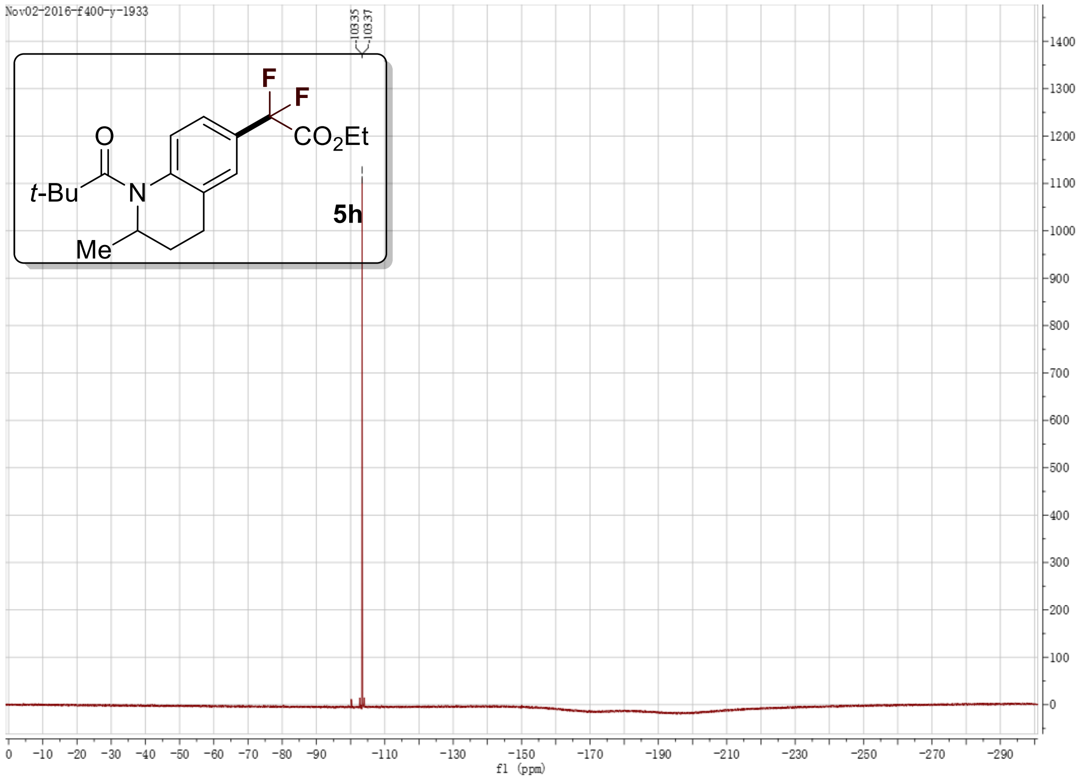

Supplementary Figure 166. ^{19}F NMR spectra for 5h

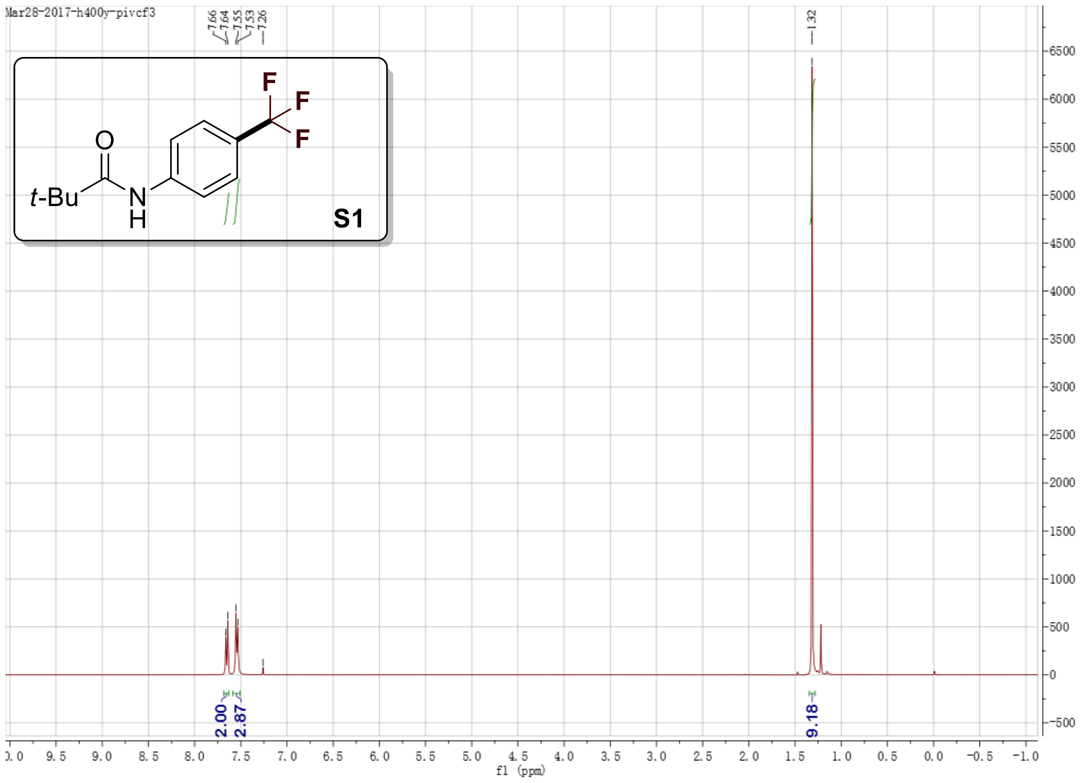

Supplementary Figure 167. ^{1}H NMR spectra for S1

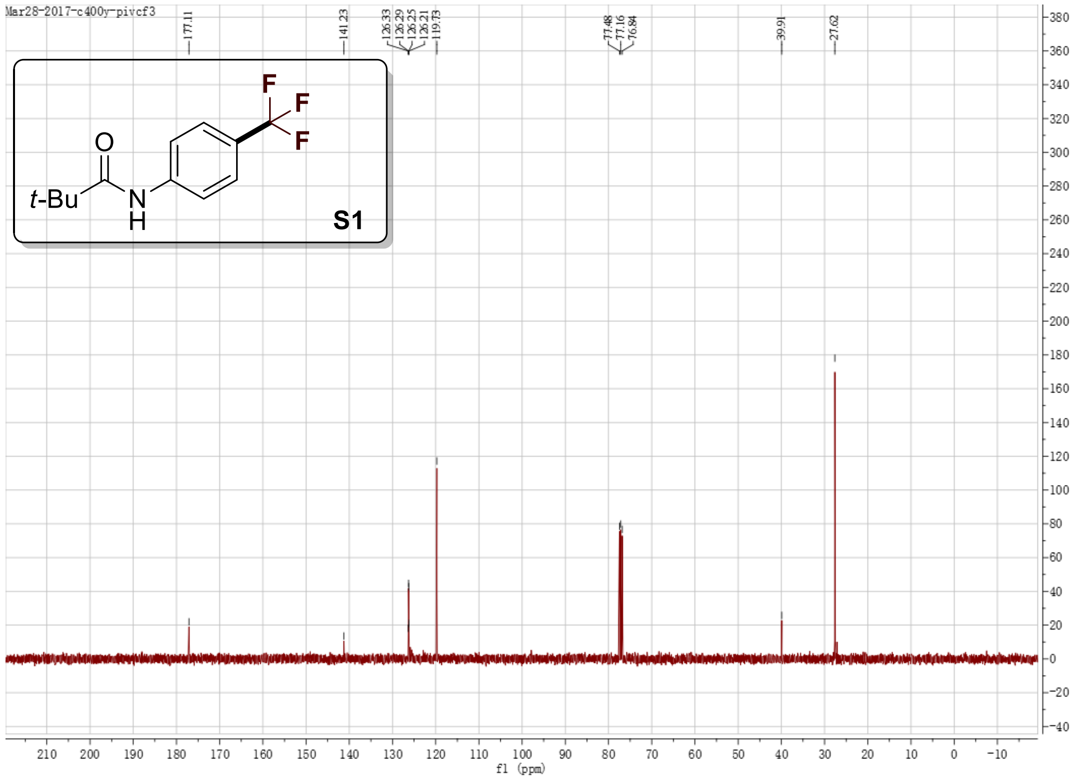

Supplementary Figure 168. ^{13}C NMR spectra for S1

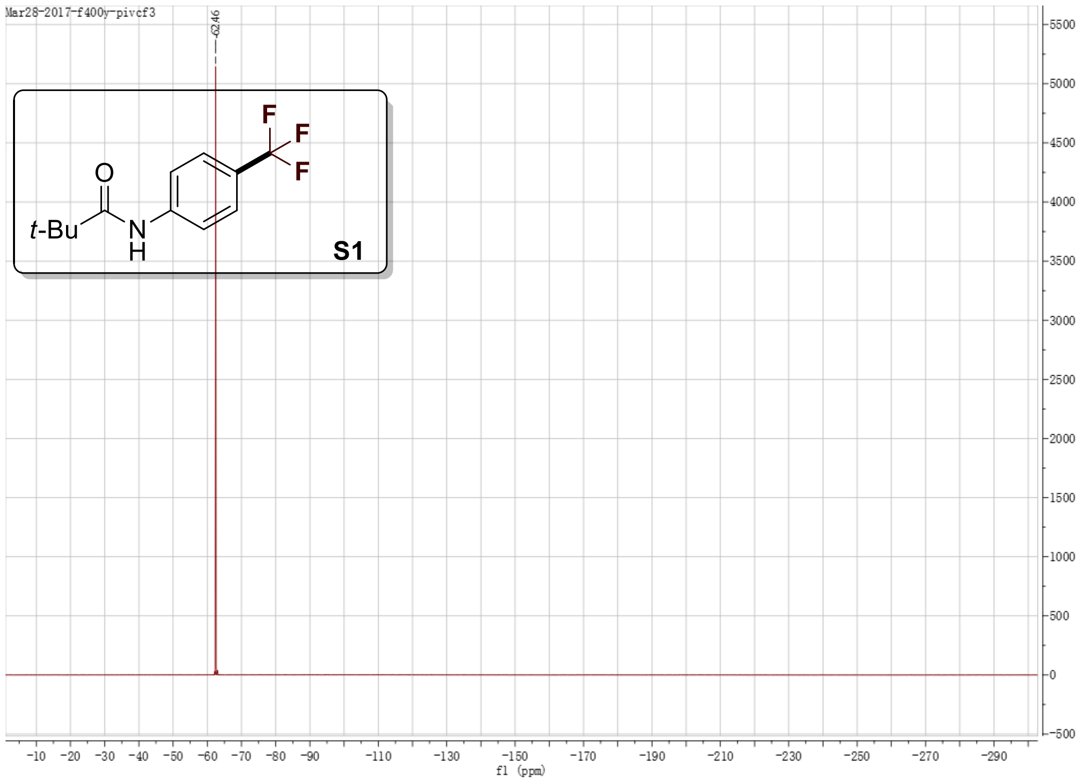

Supplementary Figure 169. ^{19}**F NMR spectra for S1**

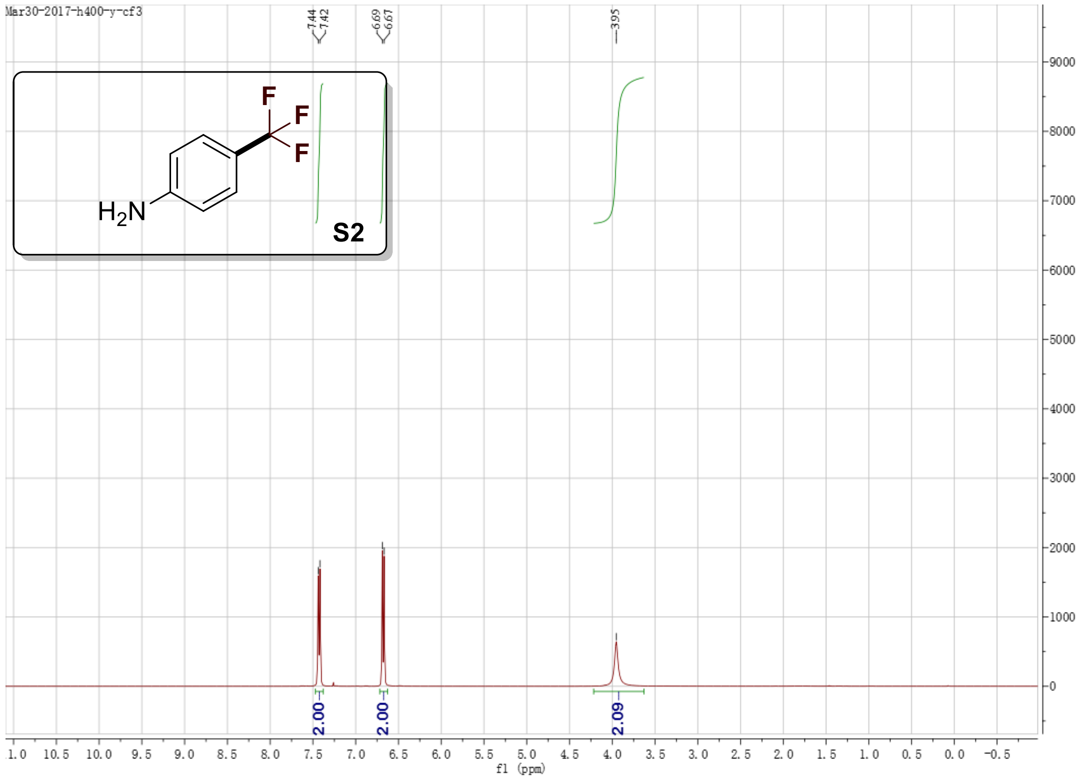

Supplementary Figure 170. ^{1}**H NMR spectra for S2**

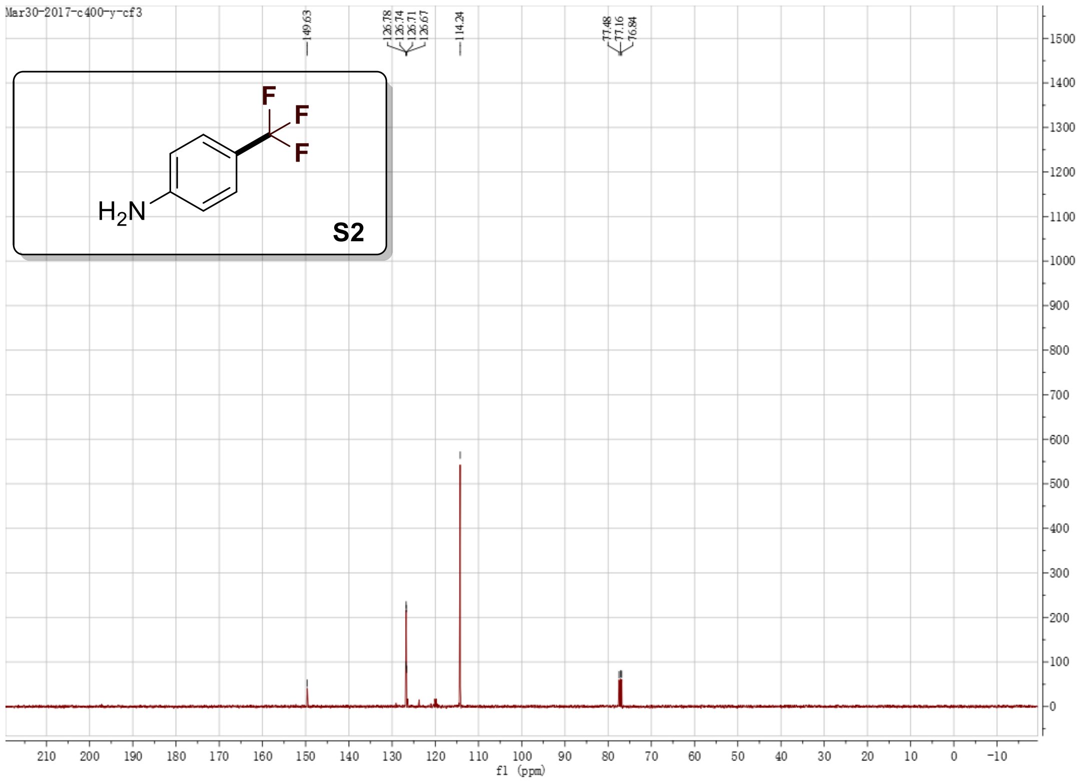

Supplementary Figure 171. ^{13}C NMR spectra for S2

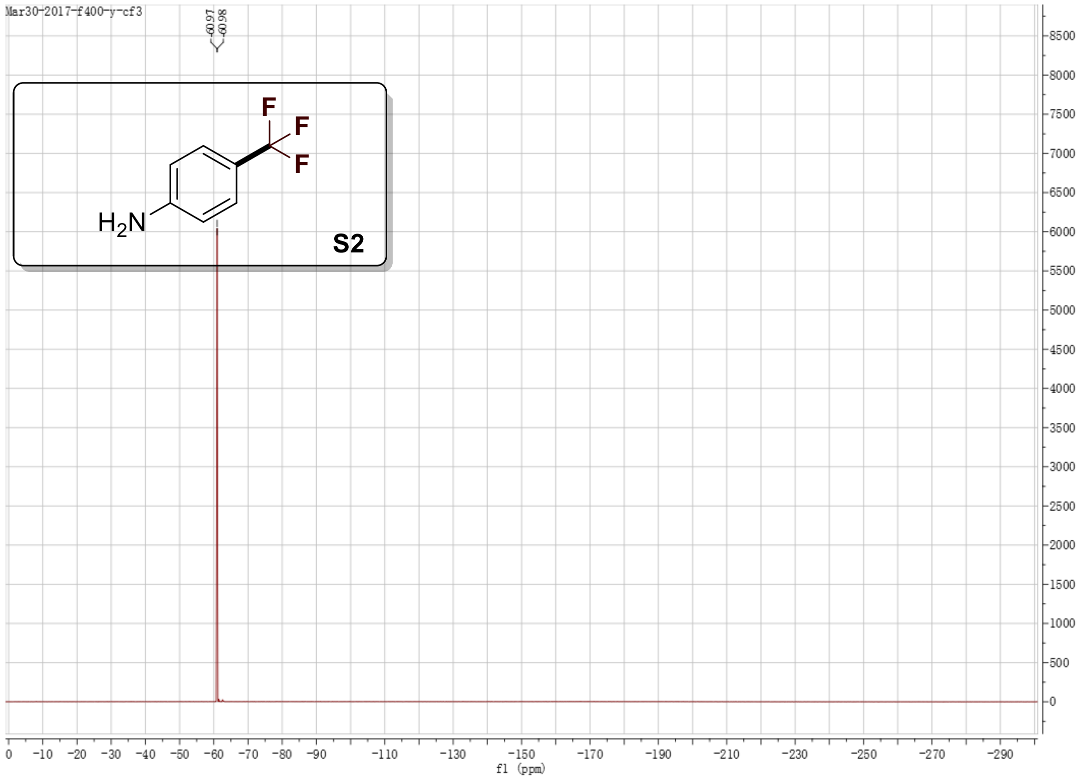

Supplementary Figure 172. ^{19}F NMR spectra for S2

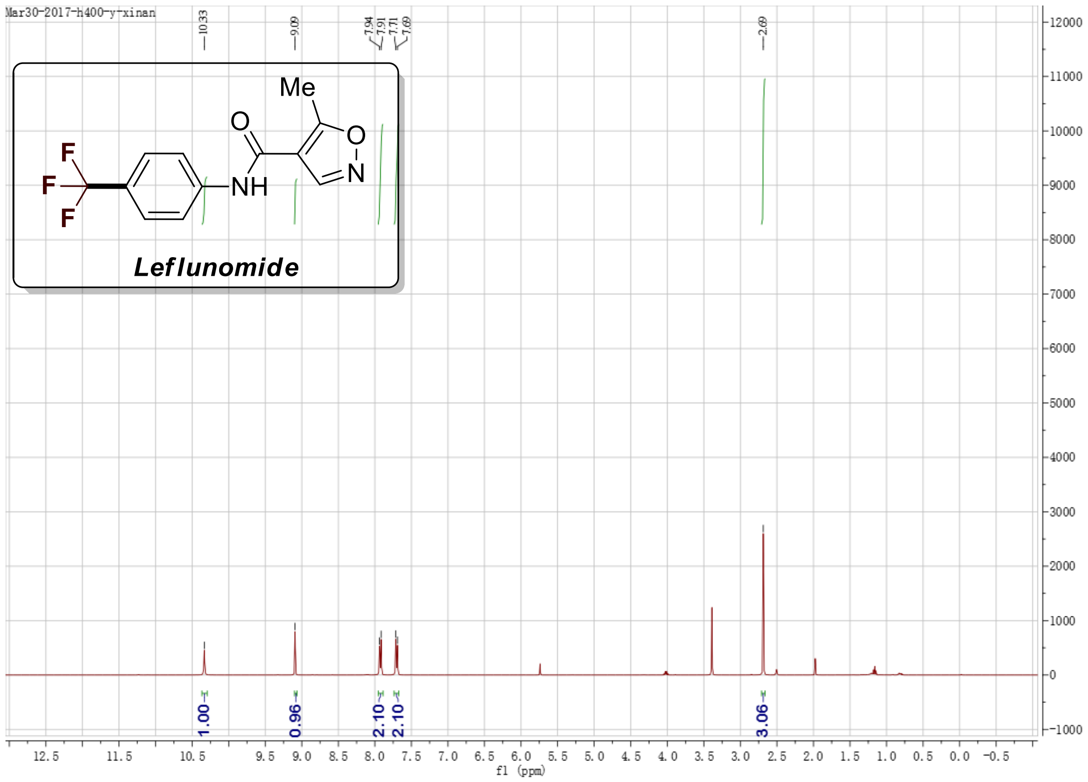

Supplementary Figure 173. ^{1}H NMR spectra for Leflunomide

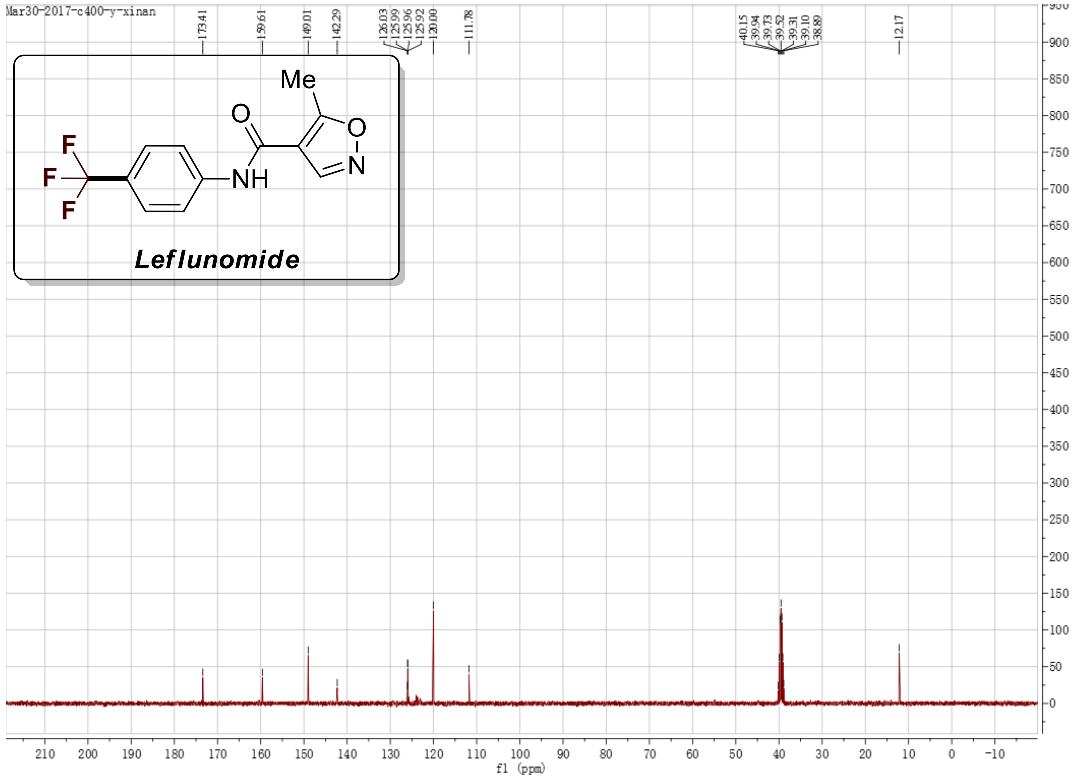

Supplementary Figure 174. ^{13}C NMR spectra for Leflunomide

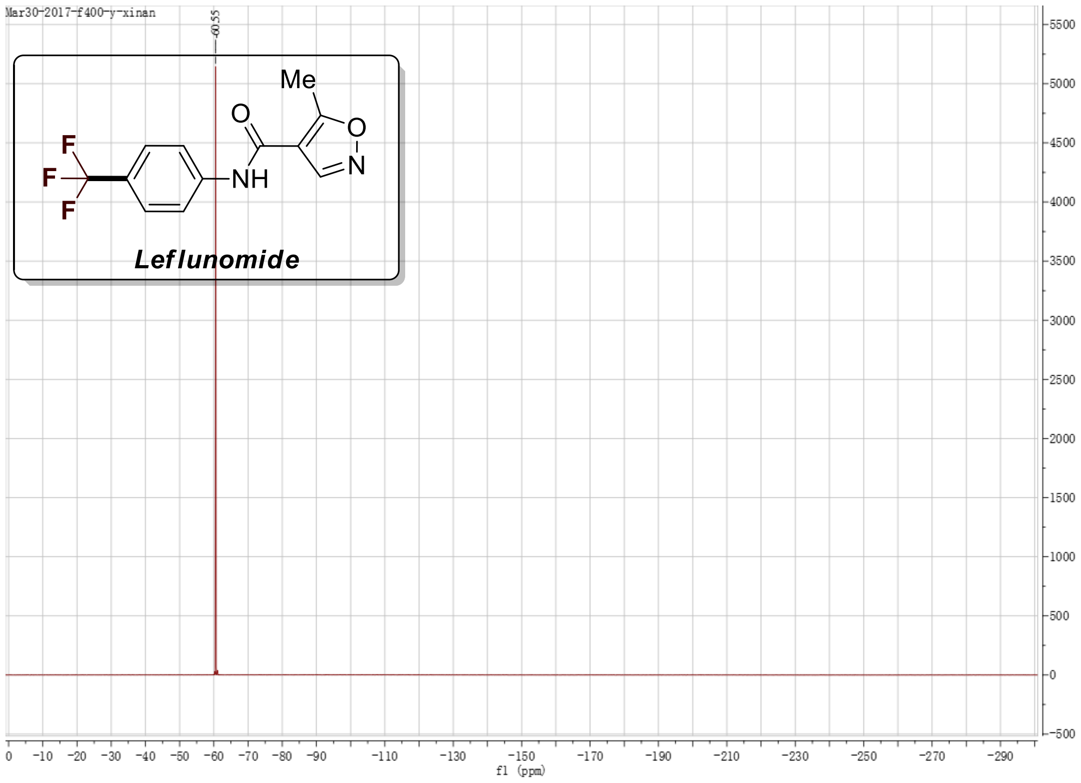

Supplementary Figure 175. ^{19}F NMR spectra for Leflunomide

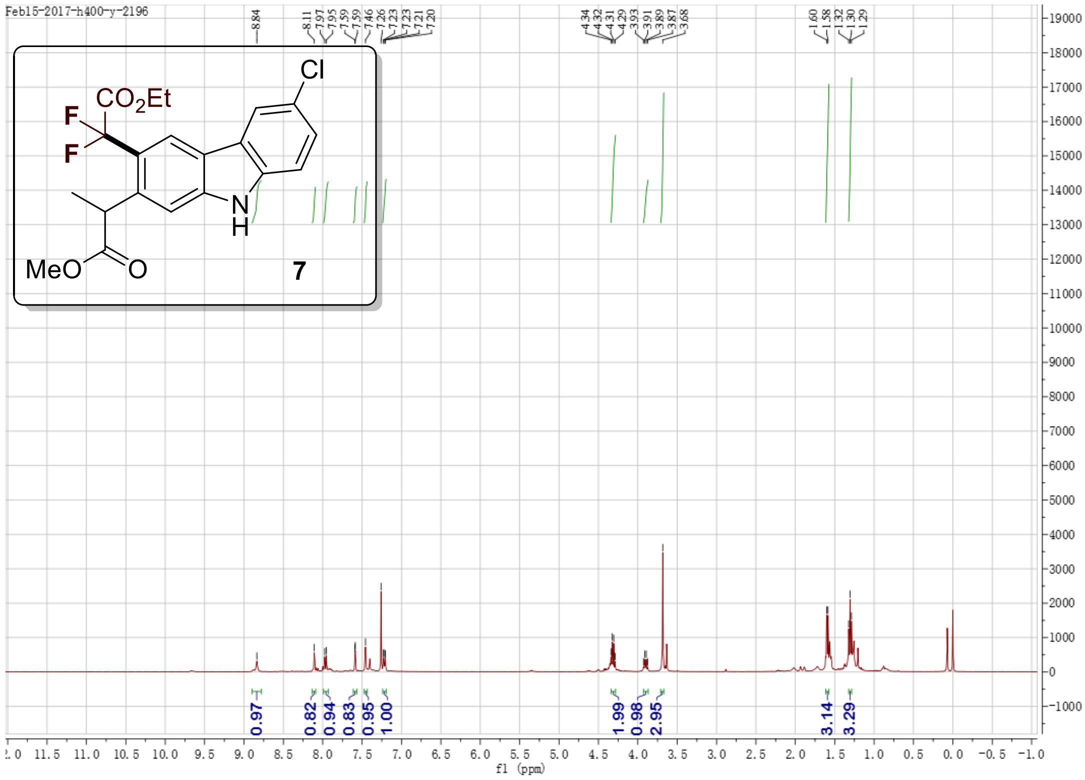

Supplementary Figure 176. ^{1}H NMR spectra for 7

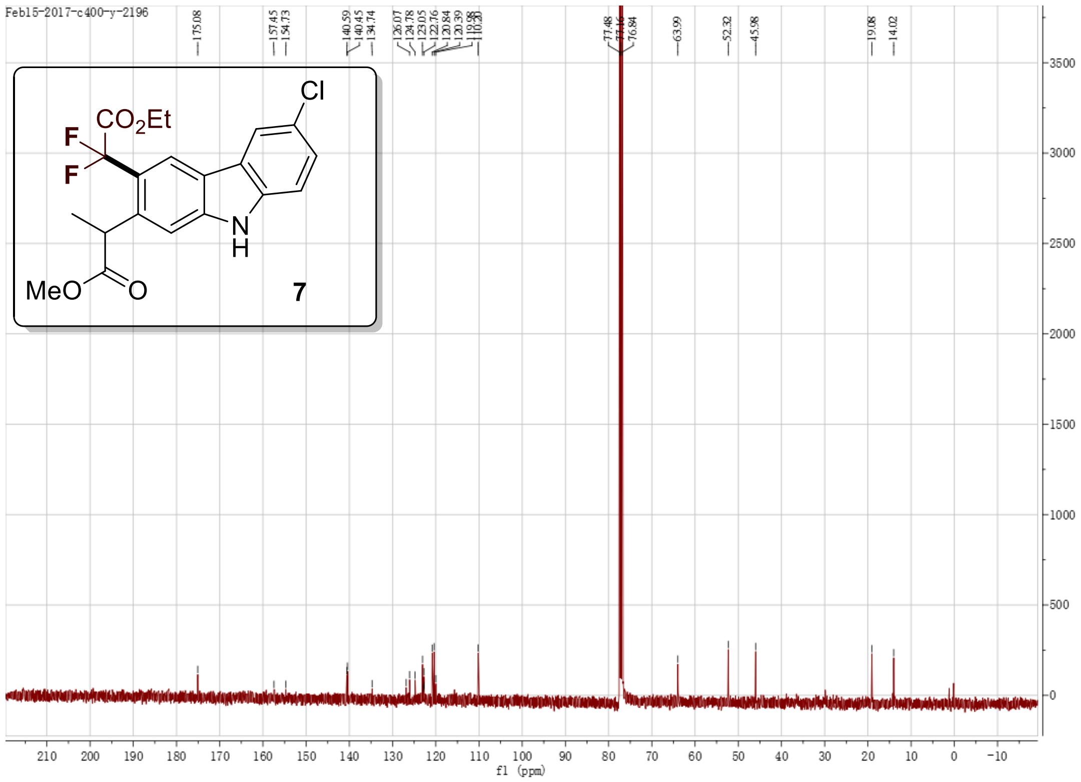

Supplementary Figure 177. ^{13}C NMR spectra for 7

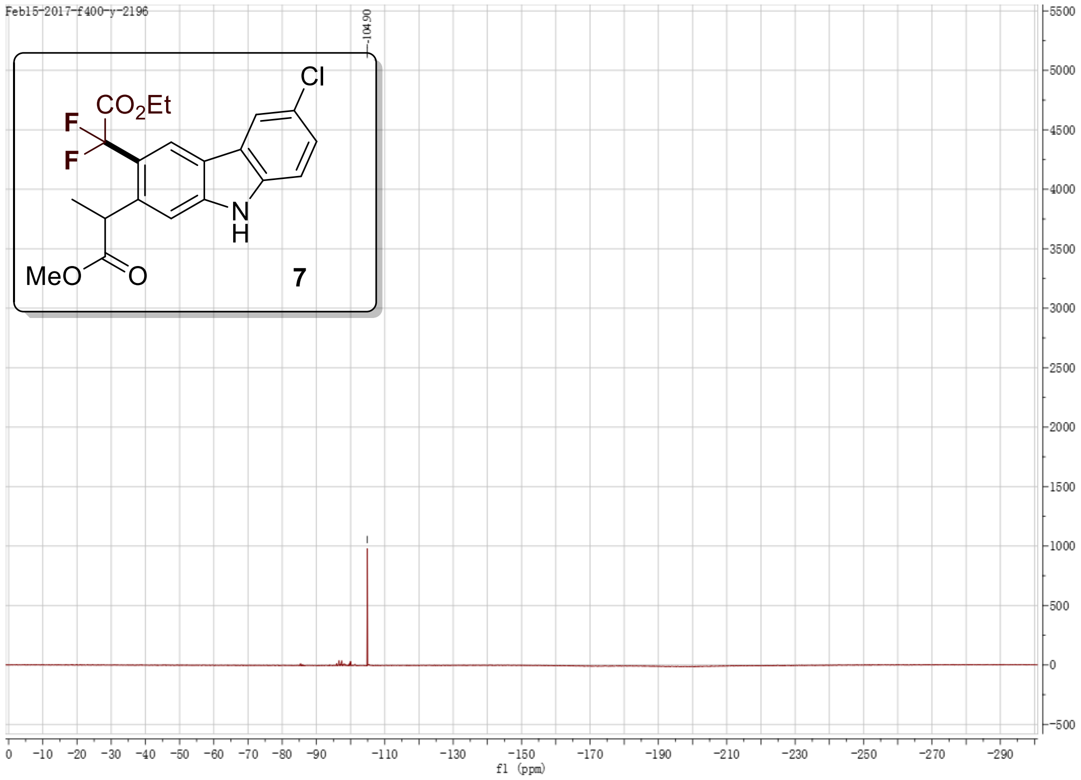

Supplementary Figure 178. ^{19}F NMR spectra for 7

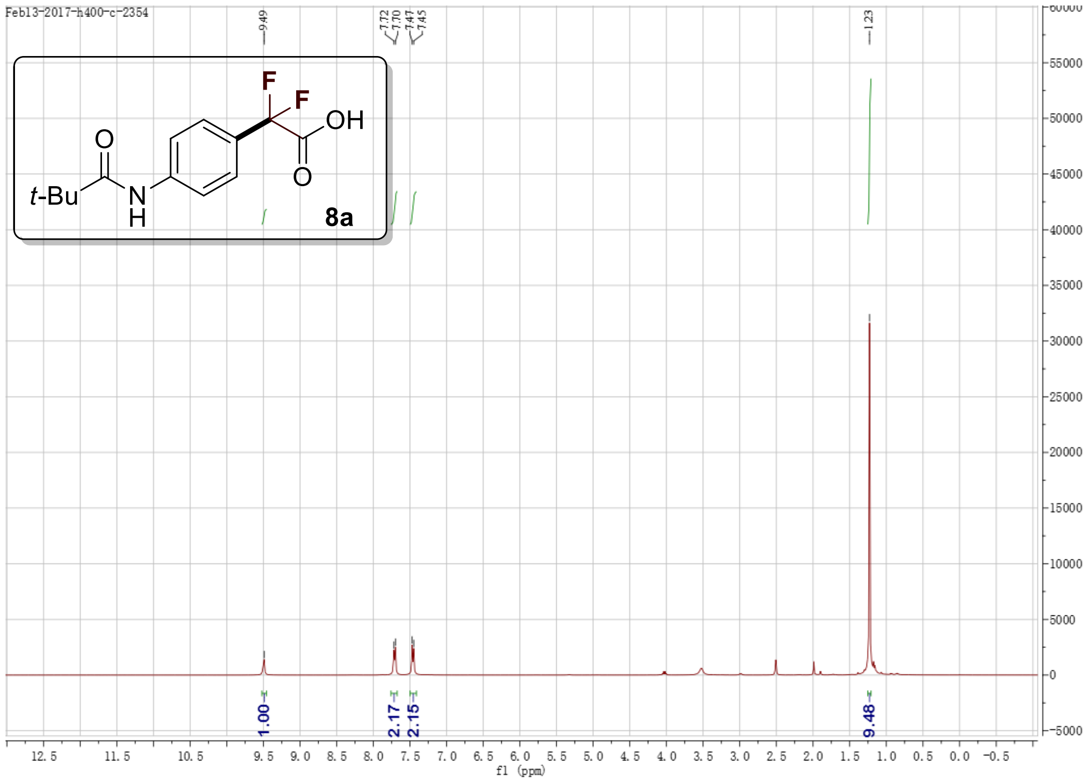

Supplementary Figure 179. ^{1}H NMR spectra for 8a

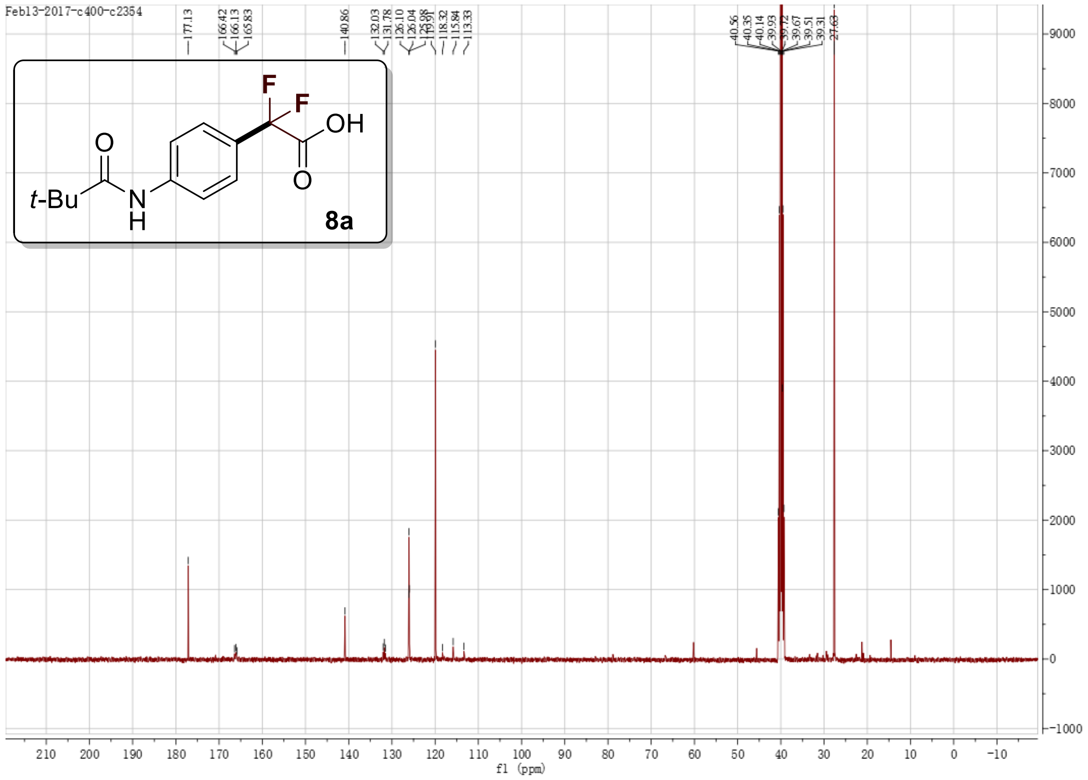

Supplementary Figure 180. ^{13}C NMR spectra for 8a

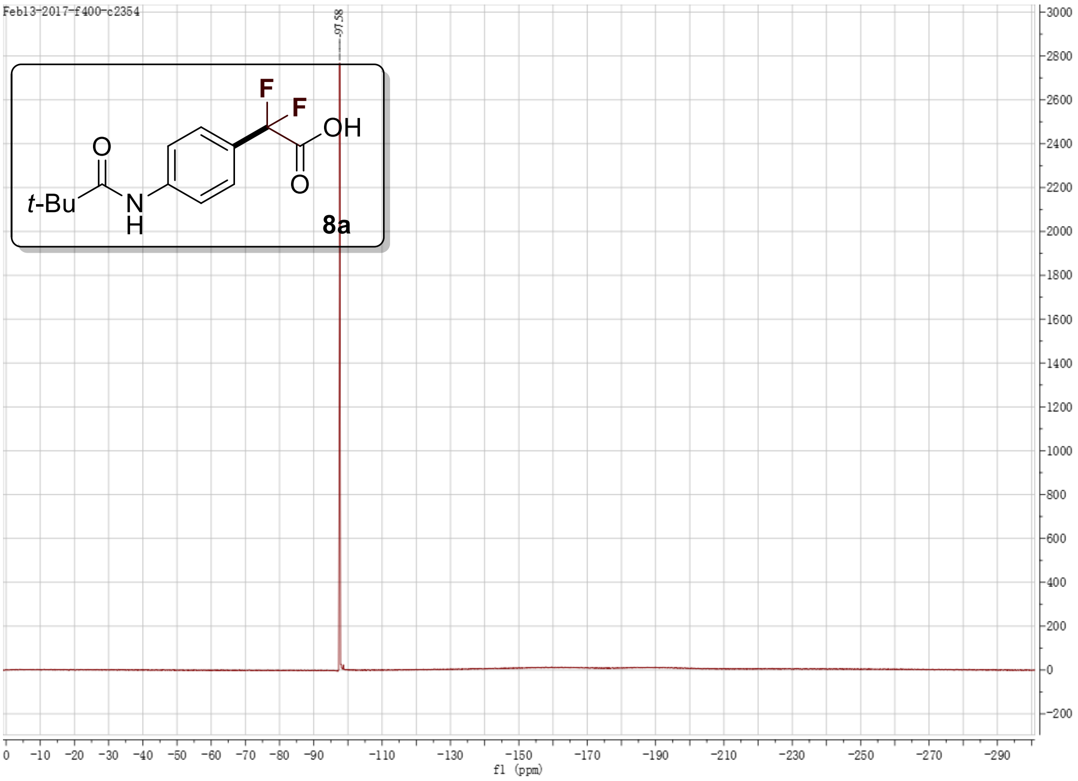

Supplementary Figure 181. ^{19}F **NMR spectra for 8a**

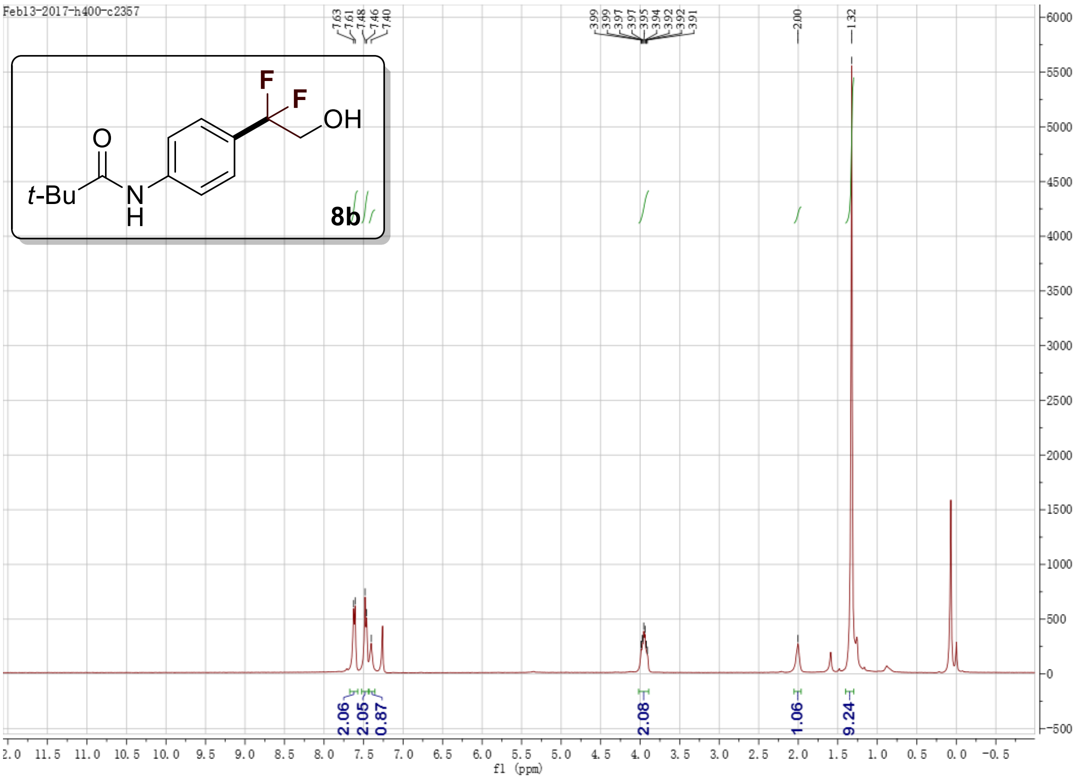

Supplementary Figure 182. ^{1}H **NMR spectra for 8b**

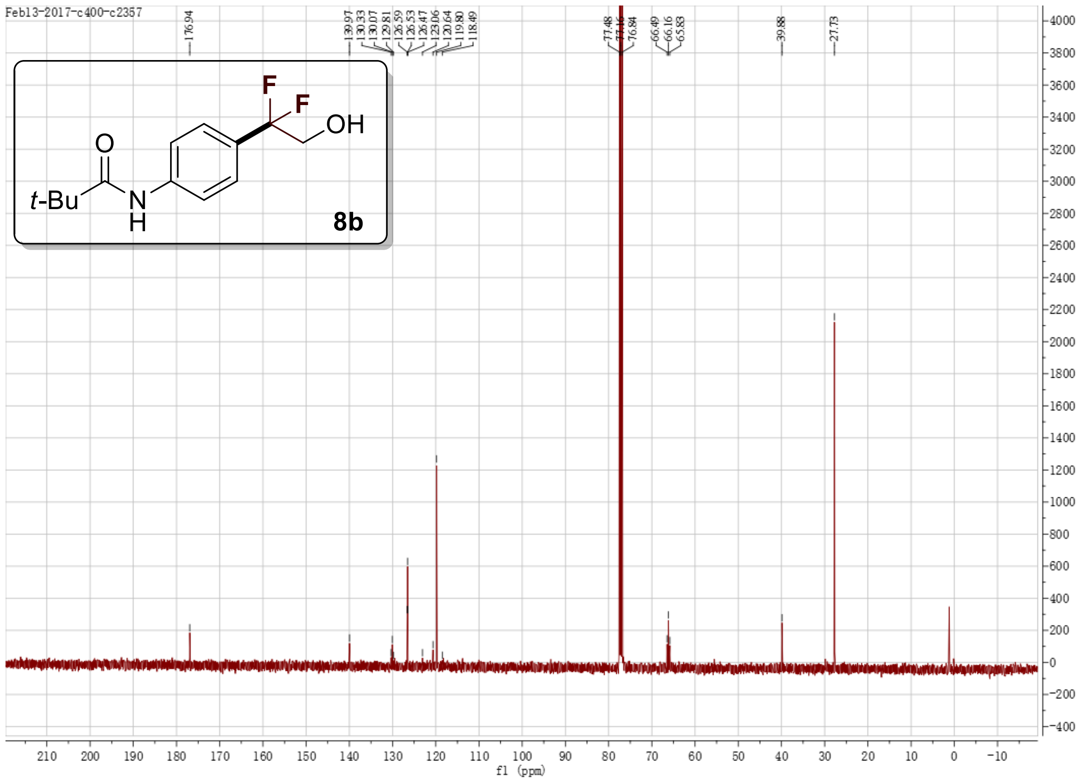

Supplementary Figure 183. ^{13}**C NMR spectra for 8b**

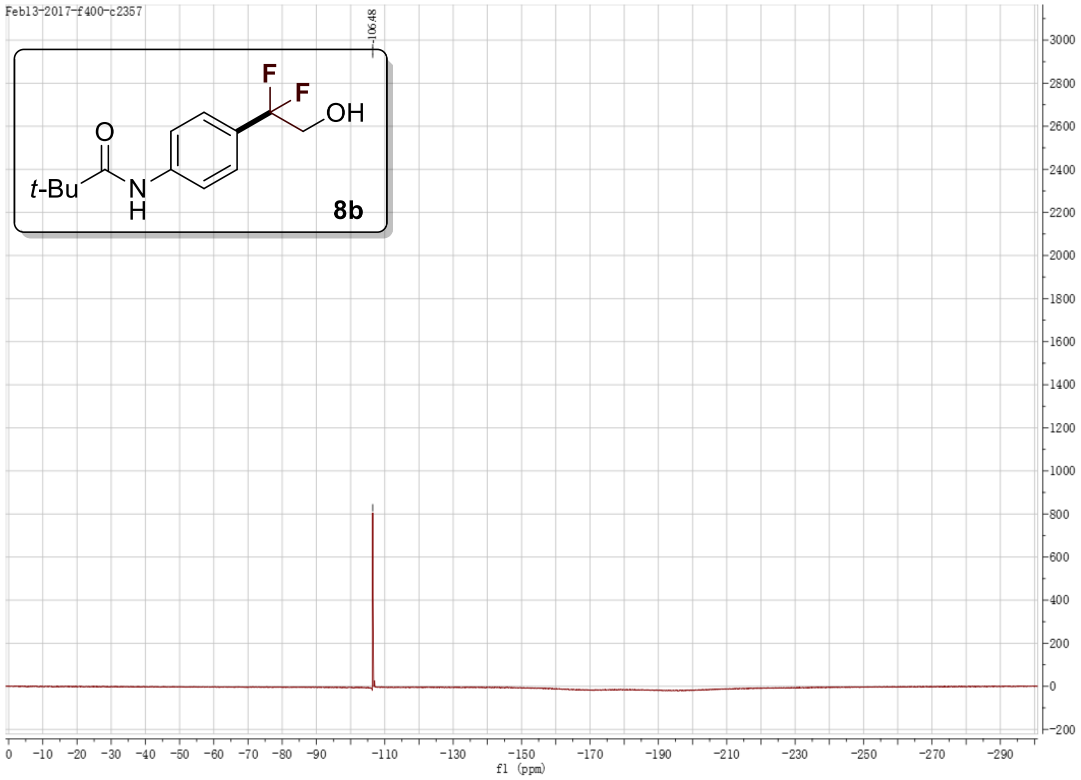

Supplementary Figure 184. ^{19}**F NMR spectra for 8b**

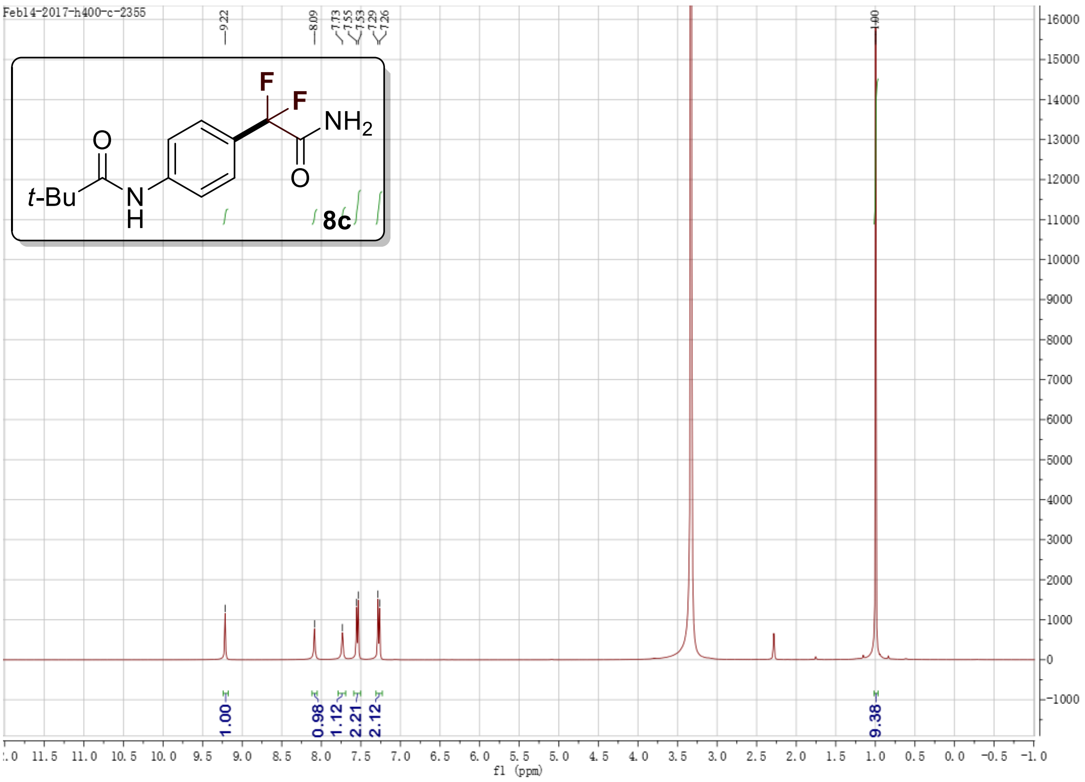

Supplementary Figure 185. ^{1}H NMR spectra for 8c

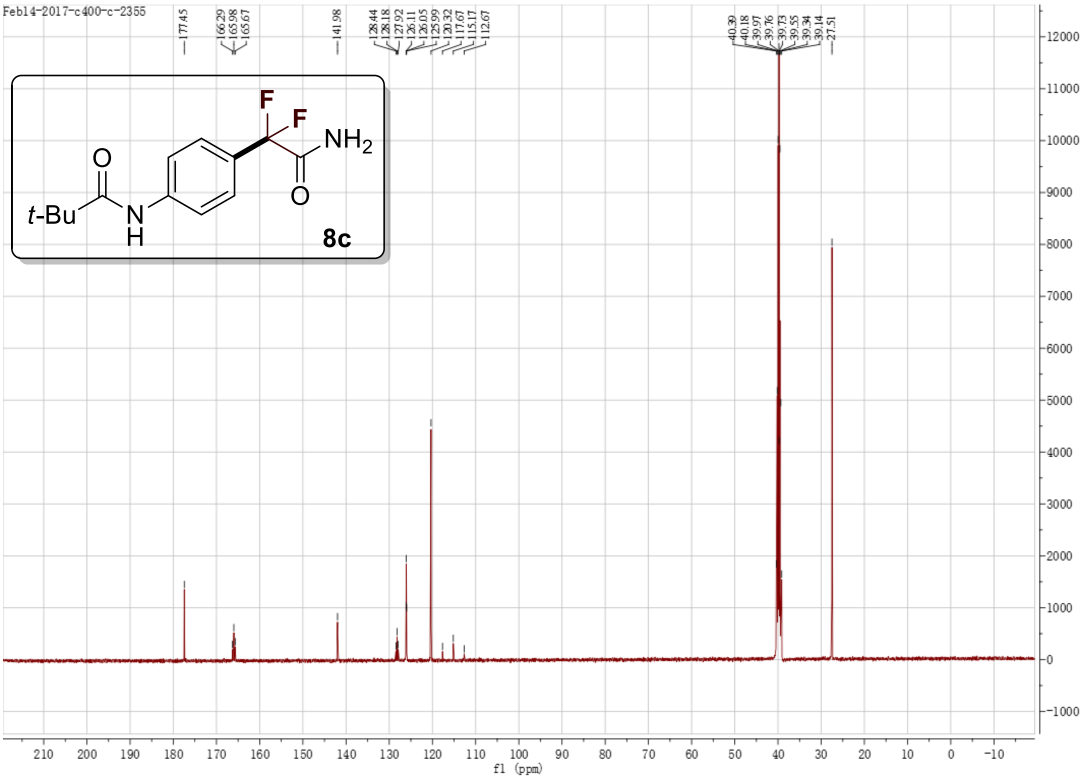

Supplementary Figure 186. ^{13}C NMR spectra for 8c

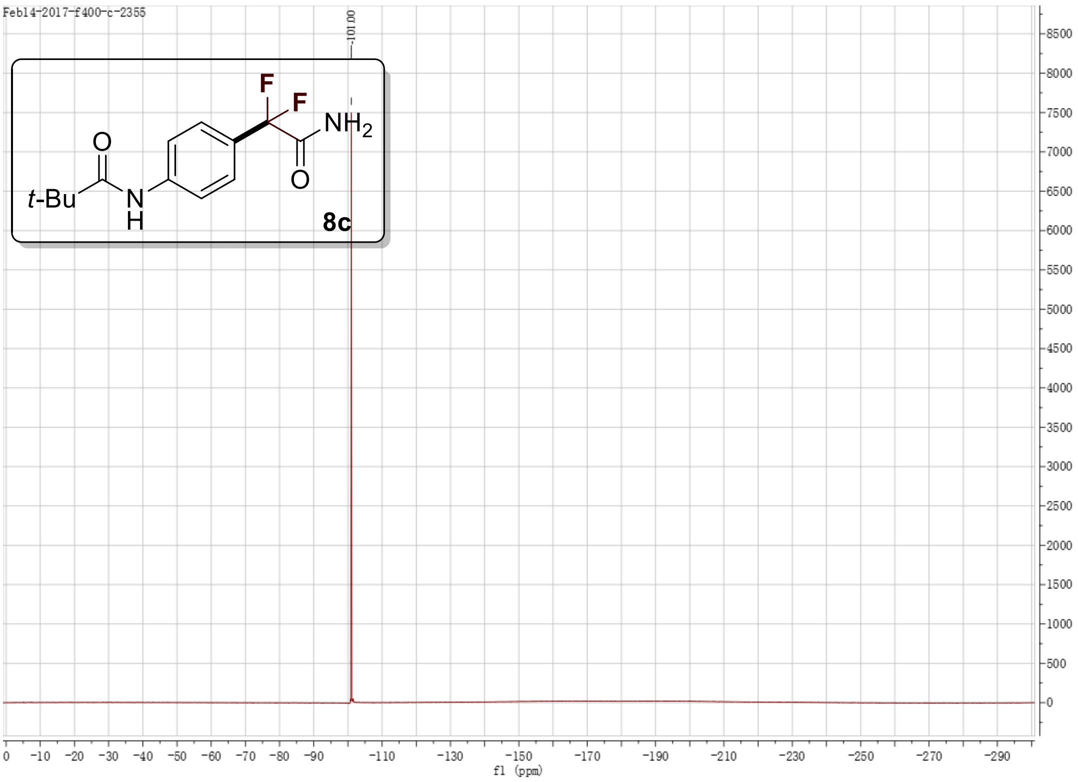

Supplementary Figure 187. ^{19}F NMR spectra for 8c

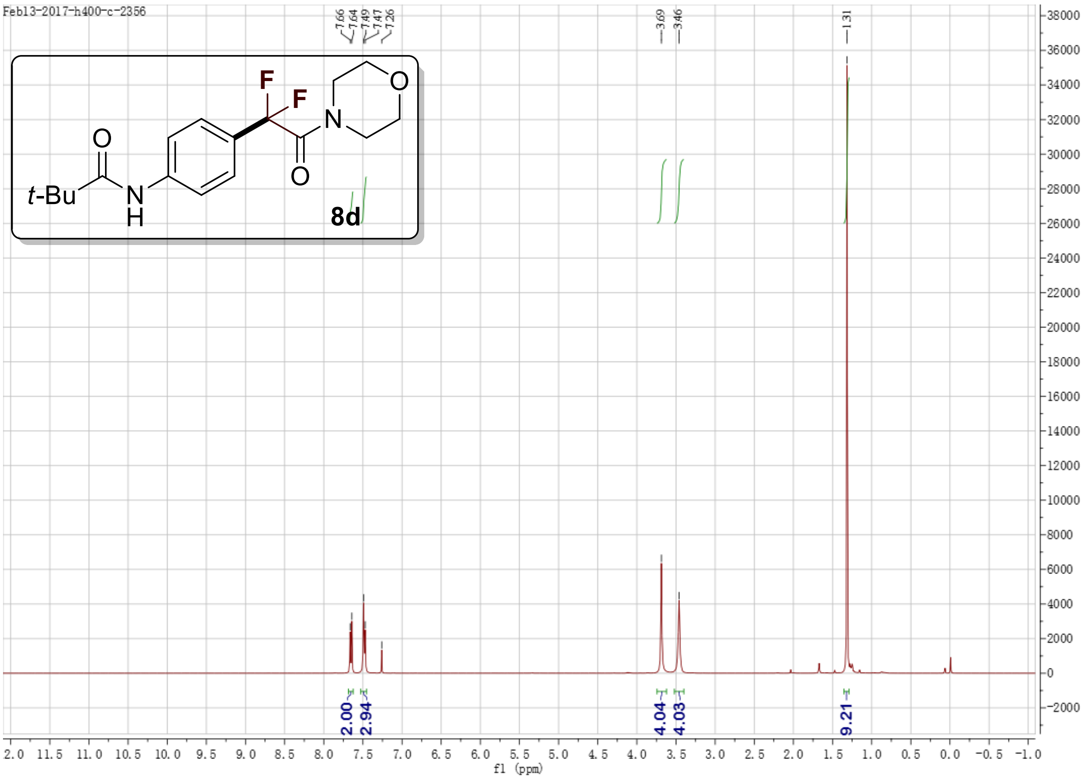

Supplementary Figure 188. ^{1}H NMR spectra for 8d

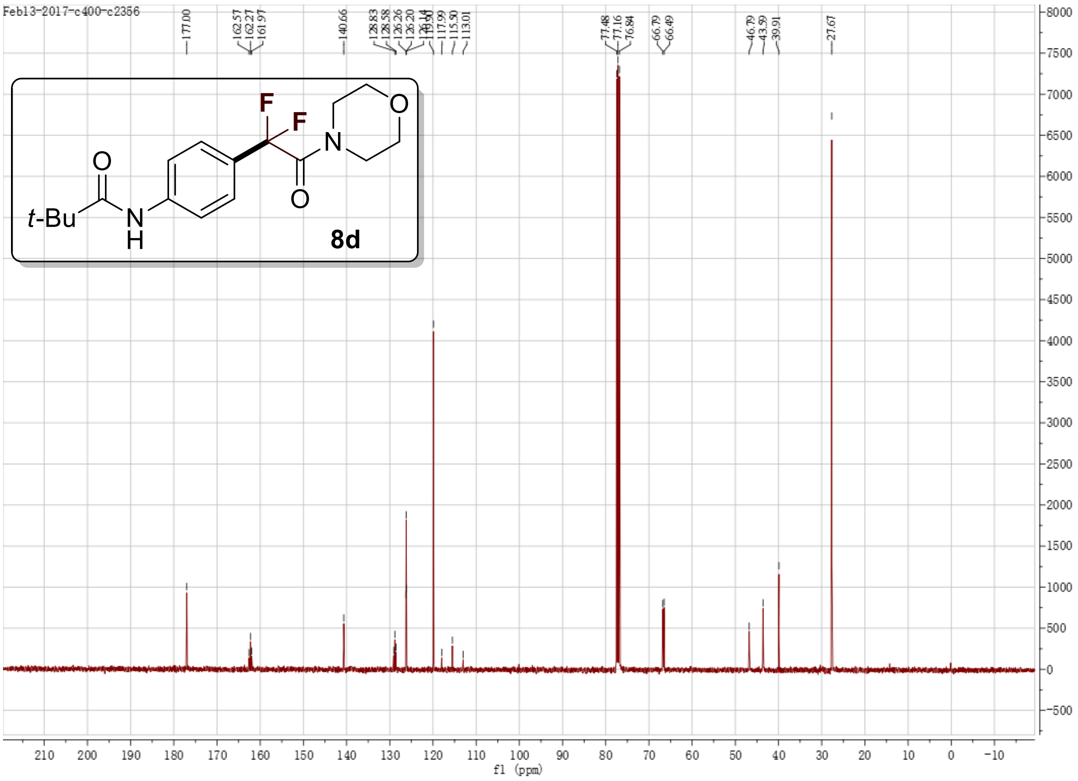

Supplementary Figure 189. ^{13}C NMR spectra for 8d

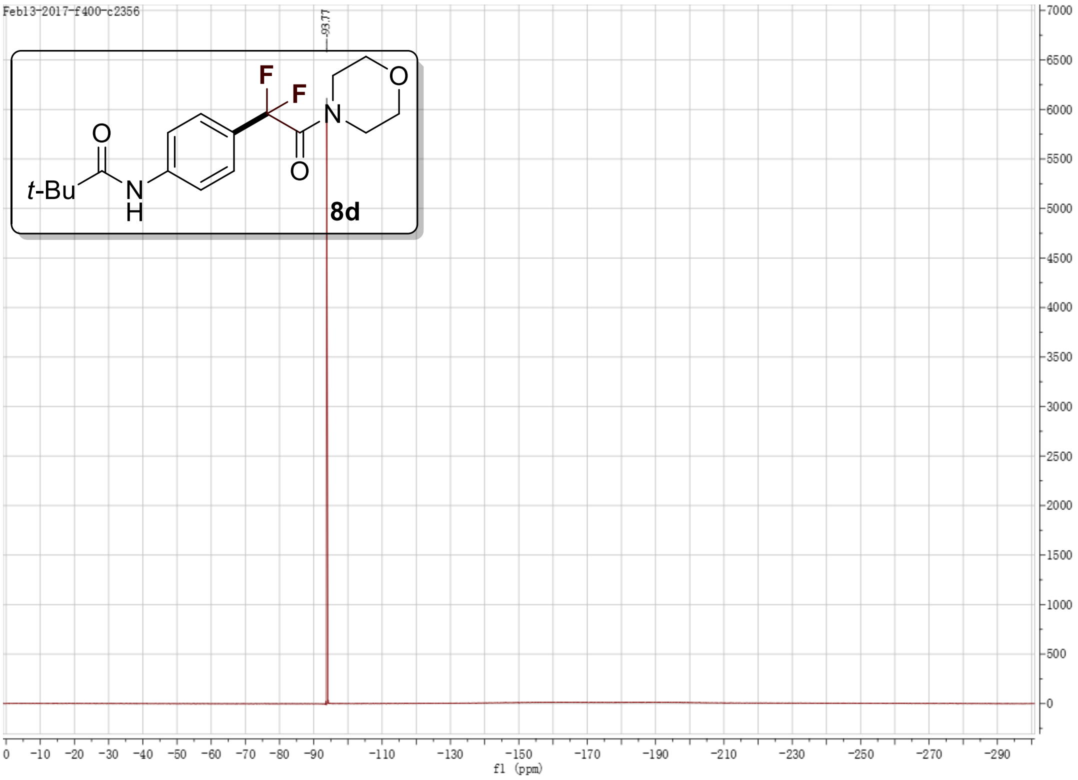

Supplementary Figure 190. ^{19}F NMR spectra for 8d

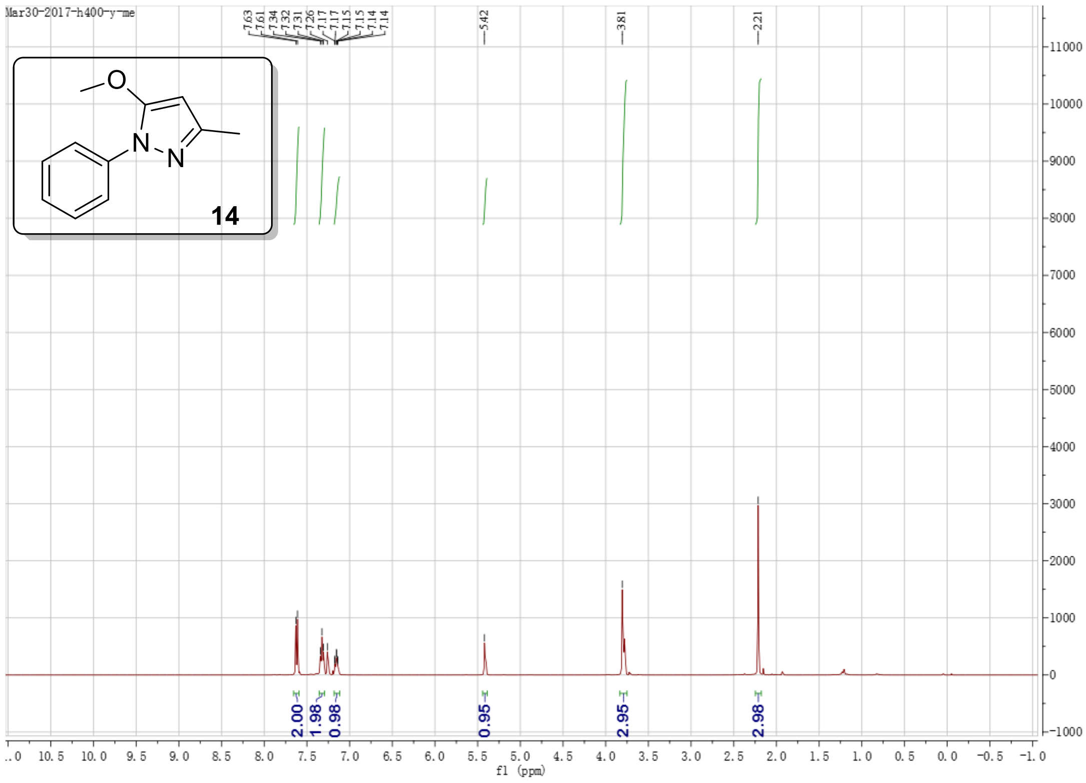

Supplementary Figure 194. ^{1}**H NMR spectra for 14**

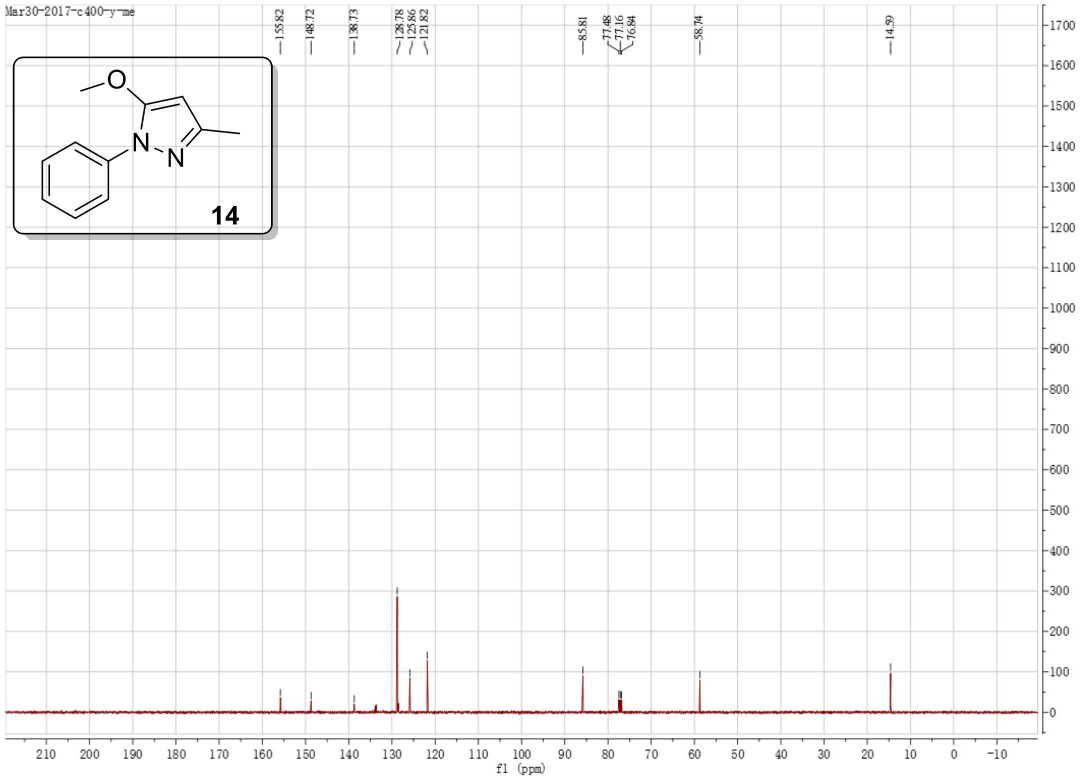

Supplementary Figure 195. ^{13}**C NMR spectra for 14**

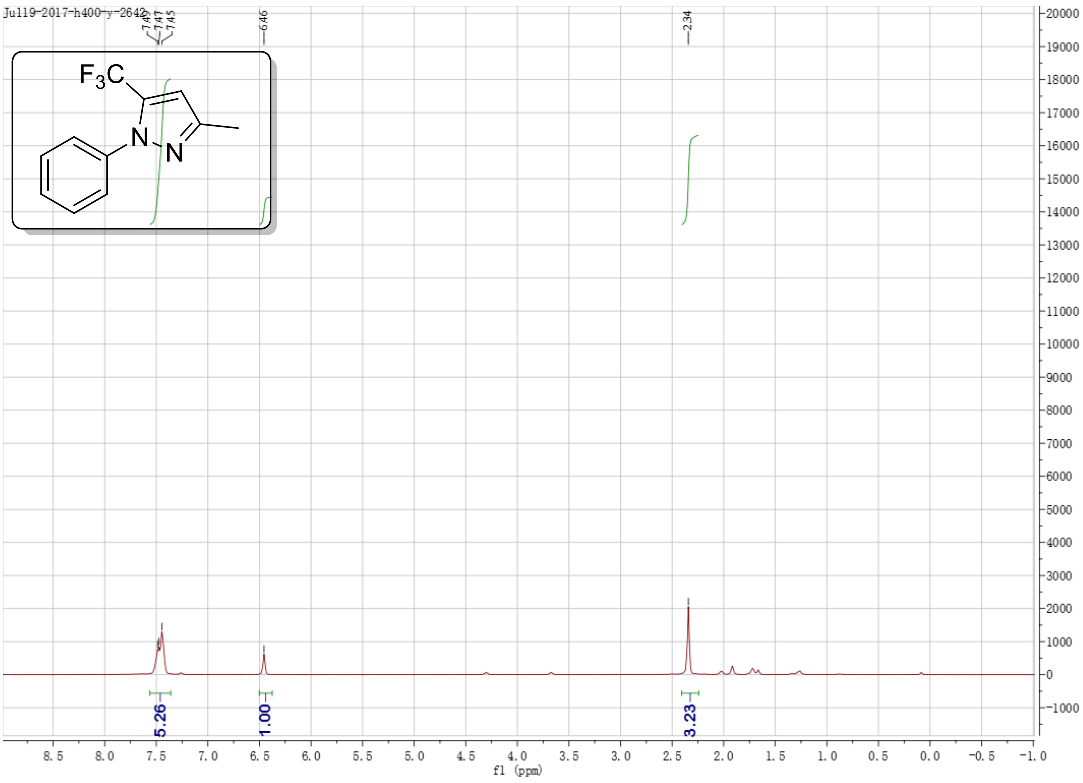

Supplementary Figure 196. **^{1}H NMR spectra for 3-methyl-5-trifluoromethylphenylpyrazole**

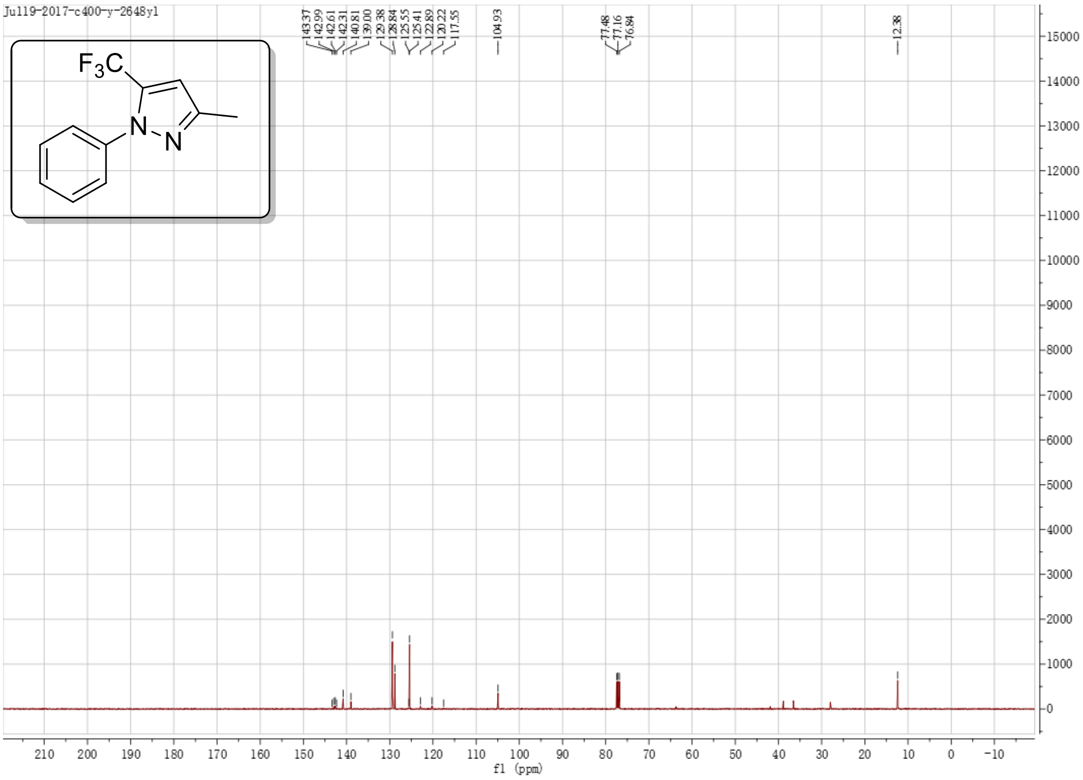

Supplementary Figure 197. **^{13}C NMR spectra for 3-methyl-5-trifluoromethylphenylpyrazole**

Supplementary References

[1] Zhang, G.-T. *et al.* External Oxidant-Free Oxidative Cross-Coupling: A Photoredox Cobalt-Catalyzed Aromatic C–H Thiolation for Constructing C–S Bonds *J. Am. Chem. Soc.***137(29)**, 9273-9280 (2015).

[2] Mizuta, S. *et al*. Catalytic Decarboxylative Fluorination for the Synthesis of Tri- and Difluoromethyl Arenes *Org. Lett*. **15**, 2648-2651 (2013).

[3] Kitson, S. L., Jones, S., Watters, W., Chen, F. & Madge, D. Carbon-14 radiosynthesis of 4-(5-chloro-2-hydroxyphenyl)-3-(2-hydroxyethyl)-6-(trifluoromethyl)-[4-14C]quinolin-2(1H)-one (XEN-D0401), A novel BK channel activator. *J. Label Compd. Radiopharm* **53**, 140-146 (2010).

[4] Dai, H., Yu, C., Lu, C. & Yan, H. Rh^{III}-Catalyzed C–H Allylation of Amides and Domino Cycling Synthesis of 3,4-Dihydroisoquinolin-1(2H)-ones with N-Bromosuccinimide*Eur. J. Org. Chem.* **2016**, 1255 (2016).

[5] Sun, X.-Y. & Yu, S.-Y. Visible-Light-Mediated Fluoroalkylation of Isocyanides with Ethyl Bromofluoroacetates: Unified Synthesis of Mono- and Difluoromethylated Phenanthridine Derivatives *Org. Lett*. **16**, 2938 (2014).

[6] Smith, G. R., Brenneman, D. E., Zhang, Y., Du, Y. & Retiz, A. B. Small-Molecule Anticonvulsant Agents with Potent In Vitro Neuroprotection and Favorable Drug-Like Properties *J. Mol. Neurosci.* **52**, 446 (2014).

[7] Kreutter, K. D. *et al*. Orally efficacious thrombin inhibitors with cyanofluorophenylacetamide as the P2 motif *Bioorg. Med. Chem. Lett*. **18**, 2865 (2008).

[8] Li, Z.-Y., Li. L., Li, Q.-L., Jing, K., Xu, H. & Wang, G.-W. Ruthenium-Catalyzed meta-Selective C−H Mono- and Difluoromethylation of Arenes through ortho-Metalation Strategy. Chem. Eur. J. **23**, 3285-3290 (2017).

[9] Ruan, Z.-X. *et al*. Ruthenium(II)-Catalyzed meta C−H Mono- and Difluoromethylations by Phosphine/Carboxylate Cooperation. *Angew. Chem. Int. Ed.* **56**, 2045-2049 (2017).

[10] Feng, Z., Min, Q.-Q., Xiao, Y.-L., Zhang, B. & Zhang, X. Palladium-Catalyzed Difluoroalkylation of Aryl Boronic Acids: A New Method for the Synthesis of Aryldifluoromethylated Phosphonates and Carboxylic Acid Derivatives *Angew. Chem., Int. Ed*. **53**, 1669-1673 (2014).

[11] Kondratov, L. S. *et al*. Radical Reactions of Alkyl 2-Bromo-2,2-difluoroacetates with Vinyl Ethers: “Omitted” Examples and Application for the Synthesis of 3,3-Difluoro-GABA *J. Org. Chem.* **80**, 12258-12264 (2015).

[12] Polkam, N. *et al*. Synthesis, molecular properties prediction and anticancer, antioxidant evaluation of new edaravone derivatives *Bioorg. Med. Chem. Lett*. **26**, 2562 (2016).

[13] Ackermann, L., Hofmann, N. & Vicente, R. Carboxylate-Assisted Ruthenium-Catalyzed Direct Alkylations of Ketimines. *Org. Lett.* **13**, 1875–1877 (2011).

[14] Ruan, Z.-X. *et al*. Ruthenium(II)-Catalyzed meta C−H Mono- and Difluoromethylations by Phosphine/Carboxylate Cooperation. *Angew. Chem. Int. Ed.* **56**, 2045–2049 (2017).

[15] Li, Z.-Y., Li. L., Li, Q.-L., Jing, K., Xu, H. & Wang, G.-W. Ruthenium-Catalyzed meta-Selective C−H Mono- and Difluoromethylation of Arenes through ortho-Metalation Strategy. *Chem. Eur. J.* **23**, 3285–3290 (2017).

[16] Dey, A., Maity, S. & Maiti, D. Reaching the south: metal-catalyzed transformation of the aromatic para-position. *Chem. Commun.* **52**, 12398–12414 (2016).

[17] Zhao, Y., Yan, H., Lu, H., Huang, Z. & Lei, A.-W. para-Selective C–H bond functionalization of iodobenzenes. *Chem. Commun.* **52**, 11366–11369 (2016).

[18] Ciana, C.-L., Phipps, R. J., Brandt, J. R., Meyer, F.-M. & Gaunt, M. J. A Highly Para-Selective Copper(II)-Catalyzed Direct Arylation of Aniline and Phenol Derivatives. *Angew. Chem., Int. Ed.* **50**, 458–462 (2011).

[19] Ball, L. T., Lloyd-Jones, G. C. & Russell, C. A. Gold-Catalyzed Oxidative Coupling of Arylsilanes and Arenes: Origin of Selectivity and Improved Precatalyst. *J. Am. Chem. Soc.* **136**, 254–264 (2014).

[20] Cambeiro, X. C., Boorman, T. C., Lu, P. & Larrosa, I. Redox-Controlled Selectivity of C−H Activation in the Oxidative Cross-Coupling of Arenes. *Angew. Chem., Int. Ed.* **52**, 1781–1784 (2013).

[21] Ball, L. T., Lloyd-Jones, G. C. & Russell, C. A. Gold-Catalyzed Direct Arylation. *Science* **337**, 1644–1648 (2012).

[22] Rosewall, C. F., Sibbald, Q. A., Liskin, D. V. & Michael, F. E. Palladium-Catalyzed Carboamination of Alkenes Promoted by N-Fluorobenzenesulfonimide via C−H Activation of Arenes. *J. Am. Chem. Soc.* **131**, 9488–9489 (2009).

[23] Xiao, T., Li, L., Xie, Y., Mao, Z. W., & Zhou, L. Synthesis of Gem-Difluorinated Fused Quinolines via Visible Light-Mediated Cascade Radical Cyclization. *Org. Lett.* **18**, 1004–1007 (2016).

[24] Lin, Q., Chu, L., & Qing, F.-L. Direct Introduction of Ethoxycarbonyldifluoromethyl-Group to Heteroarenes with Ethyl Bromodifluoroacetate via Visible-Light Photocatalysis. *Chin. J. Chem.* **31**, 885–891 (2013).

[25] Landelle, G., Panossian, A., Pazenok, S., Vors, J. P., & Leroux, F. R. Recent advances in transition metal-catalyzed Csp^2-monofluoro-, difluoro-, perfluoromethylation and trifluoromethylthiolation. *Beilstein J. Org. Chem.* **9**, 2476–2536 (2013).
