# Supplementary material for: Ruthenium(II)-enabled para-selective C–H difluoromethylation of anilides and their derivatives
Source: Nat Commun. 2018 Mar 22;9:1189. doi: 10.1038/s41467-018-03341-6 (PMC5864885; doi:10.1038/s41467-018-03341-6)
Supplement: Supplementary file 2 — Description of Additional Supplementary Files(PDF 49 kb) [file 41467_2018_3341_MOESM2_ESM.pdf]

Description of Additional Supplementary Files

File Name: Supplementary Data 1
Description: Cif file of 5c

File name: Supplementary Data 2
Description: Checkcif of 5c
