# Supplementary material for: Ruthenium(II)-enabled para-selective C–H difluoromethylation of anilides and their derivatives
Source: Nat Commun. 2018 Mar 22;9:1189. doi: 10.1038/s41467-018-03341-6 (PMC5864885; doi:10.1038/s41467-018-03341-6)

checkCIF/PLATON report

Structure factors have been supplied for datablock(s) a

THIS REPORT IS FOR GUIDANCE ONLY. IF USED AS PART OF A REVIEW PROCEDURE FOR PUBLICATION, IT SHOULD NOT REPLACE THE EXPERTISE OF AN EXPERIENCED CRYSTALLOGRAPHIC REFEREE.

No syntax errors found. CIF dictionary Interpreting this report

Datablock: a

```
Bond precision:  C-C = 0.0034 A          Wavelength=0.71073

Cell:           a=8.1010(6)    b=9.6695(8)        c=22.3350(19)
                alpha=90       beta=90.768(2)     gamma=90
Temperature:    296 K

                Calculated                  Reported
Volume          1749.4(2)                   1749.4(2)
Space group     P 21/n                      P 21/n
Hall group      -P 2yn                      -P 2yn
Moiety formula  C17 H20 Cl F2 N O3          C17 H20 Cl F2 N O3
Sum formula     C17 H20 Cl F2 N O3          C17 H20 Cl F2 N O3
Mr              359.79                      359.79
Dx,g cm-3       1.366                       1.366
Z               4                           4
Mu (mm-1)       0.253                       0.253
F000            752.0                       752.0
F000'           753.02
h,k,lmax        10,12,28                    10,12,28
Nref            4014                        4004
Tmin,Tmax       0.886,0.927                 0.886,0.927
Tmin'           0.881

Correction method= # Reported T Limits: Tmin=0.886 Tmax=0.927
AbsCorr = MULTI-SCAN

Data completeness= 0.998          Theta(max)= 27.459

R(reflections)= 0.0493( 2974)     wR2(reflections)= 0.1965( 4004)

S = 0.742                  Npar= 217
```

The following ALERTS were generated. Each ALERT has the format
test-name_ALERT_alert-type_alert-level.
Click on the hyperlinks for more details of the test.

Alert level B

```
PLAT939_ALERT_3_B Large Value of Not (SHELXL) Weight Optimized S .     469.86 Check
```

Alert level C

```
GOODF01_ALERT_2_C  The least squares goodness of fit parameter lies
            outside the range 0.80 <> 2.00
            Goodness of fit given =      0.742
PLAT242_ALERT_2_C Low    'MainMol' Ueq as Compared to Neighbors of        C14 Check
PLAT412_ALERT_2_C Short Intra XH3 .. XHn     H12A   ..  H17C    ..       1.87 Ang.
PLAT906_ALERT_3_C Large K value in the Analysis of Variance ......      6.186 Check
PLAT911_ALERT_3_C Missing # FCF Refl Between THmin & STh/L=  0.600          5 Report
PLAT913_ALERT_3_C Missing # of Very Strong Reflections in FCF ....          5 Note
PLAT918_ALERT_3_C Reflection(s) with I(obs) much Smaller I(calc) .          1 Check
```

Alert level G

```
PLAT066_ALERT_1_G Predicted and Reported Tmin&Tmax Range Identical          ? Check
PLAT072_ALERT_2_G SHELXL First  Parameter in WGHT  Unusually Large       0.17 Report
PLAT720_ALERT_4_G Number of Unusual/Non-Standard Labels ..........          1 Note
PLAT910_ALERT_3_G Missing # of FCF Reflection(s) Below Theta(Min)           2 Note
PLAT912_ALERT_4_G Missing # of FCF Reflections Above STh/L=  0.600          3 Note
PLAT978_ALERT_2_G Number C-C Bonds with Positive Residual Density           8 Note
```

0 **ALERT level A** = Most likely a serious problem - resolve or explain
1 **ALERT level B** = A potentially serious problem, consider carefully
7 **ALERT level C** = Check. Ensure it is not caused by an omission or oversight
6 **ALERT level G** = General information/check it is not something unexpected

1 ALERT type 1 CIF construction/syntax error, inconsistent or missing data
5 ALERT type 2 Indicator that the structure model may be wrong or deficient
6 ALERT type 3 Indicator that the structure quality may be low
2 ALERT type 4 Improvement, methodology, query or suggestion
0 ALERT type 5 Informative message, check

It is advisable to attempt to resolve as many as possible of the alerts in all categories. Often the minor alerts point to easily fixed oversights, errors and omissions in your CIF or refinement strategy, so attention to these fine details can be worthwhile. In order to resolve some of the more serious problems it may be necessary to carry out additional measurements or structure refinements. However, the purpose of your study may justify the reported deviations and the more serious of these should normally be commented upon in the discussion or experimental section of a paper or in the "special_details" fields of the CIF. checkCIF was carefully designed to identify outliers and unusual parameters, but every test has its limitations and alerts that are not important in a particular case may appear. Conversely, the absence of alerts does not guarantee there are no aspects of the results needing attention. It is up to the individual to critically assess their own results and, if necessary, seek expert advice.

Publication of your CIF in IUCr journals

A basic structural check has been run on your CIF. These basic checks will be run on all CIFs submitted for publication in IUCr journals (*Acta Crystallographica*, *Journal of Applied Crystallography*, *Journal of Synchrotron Radiation*); however, if you intend to submit to *Acta Crystallographica Section C* or *E* or *IUCrData*, you should make sure that full publication checks are run on the final version of your CIF prior to submission.

Publication of your CIF in other journals

Please refer to the *Notes for Authors* of the relevant journal for any special instructions relating to CIF submission.

PLATON version of 11/08/2016; check.def file version of 04/08/2016

Datablock a - ellipsoid plot

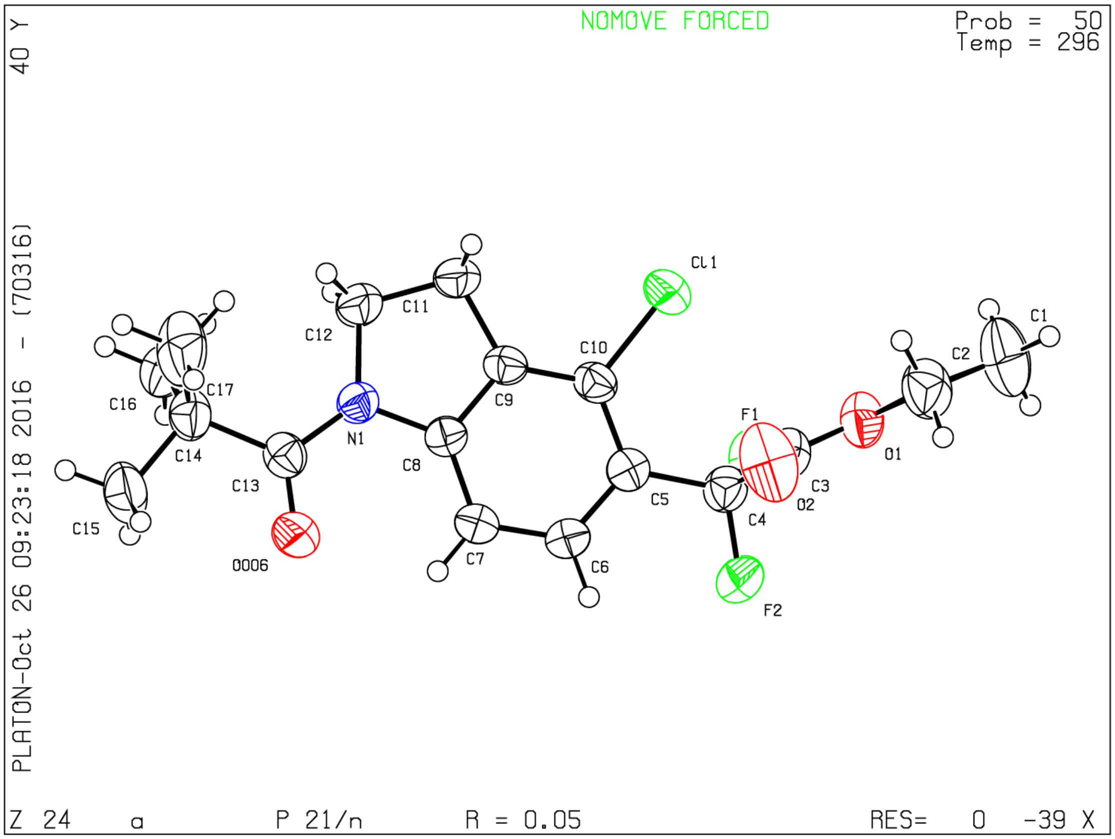

Supplement: Supplementary file 4 — Supplementary Data 2(PDF 106 kb) [file 41467_2018_3341_MOESM4_ESM.pdf]
